# Synthesis of Functionalized Indoles via Palladium-Catalyzed Cyclization of *N*-(2-allylphenyl) Benzamide: A Method for Synthesis of Indomethacin Precursor

**DOI:** 10.3390/molecules25051233

**Published:** 2020-03-09

**Authors:** Zhe Chang, Tong Ma, Yu Zhang, Zheng Dong, Heng Zhao, Depeng Zhao

**Affiliations:** School of Pharmaceutical Sciences, Sun Yat-sen University, Guangzhou 510006, China; changzh5@mail2.sysu.edu.cn (Z.C.); mt11072020@126.com (T.M.); zhangy637@mail2.sysu.edu.cn (Y.Z.); dongzh6@mail2.sysu.edu.cn (Z.D.); zhaoh5@mail2.sysu.edu.cn (H.Z.)

**Keywords:** palladium, indole, indomethacin, C-H functionalization

## Abstract

We developed an efficient method for synthesis of substituted *N*-benzoylindole via Pd(II)-catalyzed C–H functionalization of substituted *N*-(2-allylphenyl)benzamide. The reaction showed a broad substrate scope (including *N*-acetyl and *N*-Ts substrates) and substituted indoles were obtained in good to excellent yields. The most distinctive feature of this method lies in the high selectivity for *N*-benzoylindole over benzoxazine, and this is the first example of Pd(II)-catalyzed synthesis of substituted *N*-benzoylindole. Notably, this new method was applied for the synthesis of key intermediate of indomethacin.

## 1. Introduction

**Figure 1:**
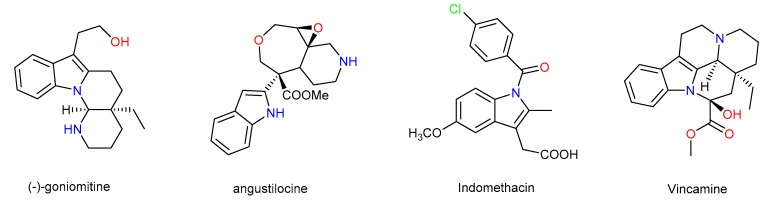
Representative molecules based on indole skeleton.

**Figure 2:**
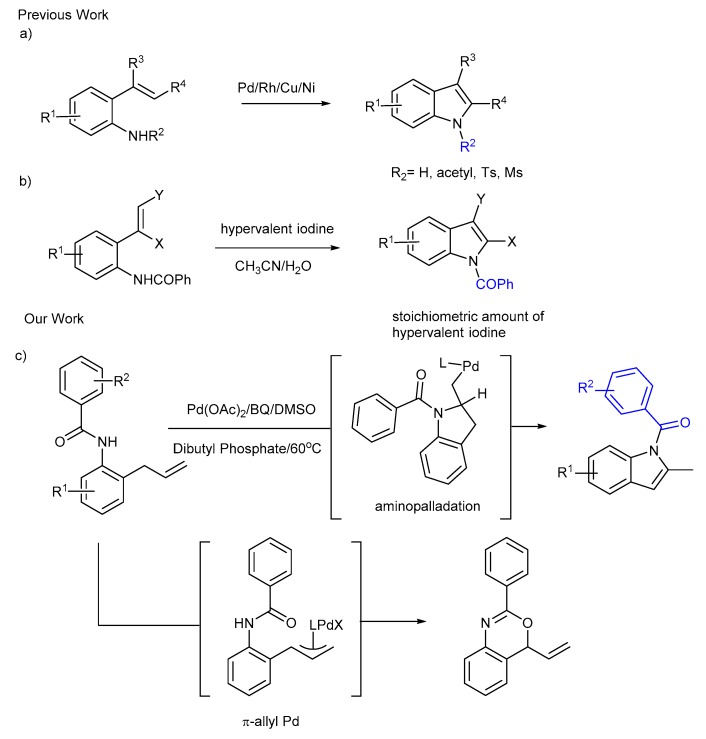
Methods for synthesis of substituted *N*-benzoylindole. (**a**) The synthesis of indoles by employing transition-metal-catalyzed. (**b**) The synthesis of *N*-benzoylindole by hypervalent iodine as the oxidant. (**c**) This work.

Indole skeletons are one of the most valuable heterocycles, due to their diverse biological activities and broad applications in functionalized materials and chemistry [1,2,3]. Substituted indoles exist extensively in nature and pharmaceuticals (Figure 1) [2,4,5]. As a result, many methods have been developed for the synthesis of indoles, including pioneering studies by Fischer, [6] Larock [7,8], Buchwald [9,10,11], and Hegedus [12,13,14,15]. Recently, an increasing number of approaches for the synthesis of indoles by employing transition-metal-catalyzed oxidative C-H bond functionalization has been reported (Figure 2a) [16,17,18,19,20,21,22,23,24,25,26]. These methods showed significant improvement with regard to the substrate scope and reaction conditions [27,28,29,30,31], but *N*-substituents in these reports are restricted to H, acetyl, Ts (tosyl), and Ms (mesyl). In addition, Rh(III)-catalyzed tandem C-H allylation and oxidative cyclization of anilides with allyl carbonates or acetates have been developed, albeit with costly Rh complexes [32,33,34]. Despite these achievements, it is still of great value to develop methods for synthesizing substituted indoles, especially *N*-benzoyl with low cost and wide variety of substituents. Substituted *N*-benzoylindole is one of the most attractive skeletons, since it is a privileged structure of many pharmacologically active compounds such as indomethacin. Besides, the only method reported to construct substituted *N*-benzoyl indole is the C-H-amination of styrenes using hypervalent iodine as the oxidant (Figure 2b) [35,36]. However, it should be noted that the oxidants of these two methods are not commercially available. A direct approach employing simple and readily available catalyst and oxidant remains a challenge for the synthesis of substituted *N*-benzoylindole. In this context, our strategy is to use commercially available and inexpensive catalyst Pd(OAc)_2_ and oxidant benzoquinone (BQ) for C–H functionalization to construct substituted *N*-benzoyl indoles (Figure 2c). It was reported that either aminopalladated or π-allyl Pd intermediates would be generated in palladium-catalyzed allylic C–H oxidation reaction with the usage of ambident O/N nucleophiles [37,38,39]. Our method can avoid the generation of the π-allyl Pd intermediates and obtain corresponding *N*-benzoyl indoles. More importantly, the synthetic utility of this method is further demonstrated by the synthesis of essential skeleton of indomethacin.

## 2. Results and Discussion

We began our study by the reaction of **1a** in the presence of 10 mol% of Pd (OAc)_2_ as the catalyst and BQ (1.5 equiv.) as the oxidant in MeCN at room temperature (Table 1, Entry 1). Gratifyingly, the desired product **2a** was obtained in 8% yield along with a by-product benzoxazine **3** formed via allylic C-H cleavage (**2a**/**3** = 1/1) [38,39]. Encouraged by this result, we further systematically optimized the reaction conditions to improve the conversion of the reaction and inhibit the formation of benzoxazine **3**. When the reaction was performed at elevated temperature of 60 °C, a slightly higher yield of **2a** was achieved and the ratio of **2a** to **3** was also enhanced to 4:1 (Table 1, Entry 3). Interestingly, addition of a stoichiometric amount of AcOH facilitated this reaction to give a higher selectivity (Table 1, Entry 4), improving the ratio to 10:1. Encouraged by this result, we evaluated several acids as additives of the reaction, as shown in Table 1 (Entries 5–8). We were excited to find that using dibutyl phosphate (DBP) as the acid led to a significantly higher yield and the ratio of **2a** to **3** was also improved to more than 20:1 (71% yield, Table 1, Entry 8). With dibutyl phosphate as the optimal additive, two other Pd catalysts were tested, but no satisfactory results were obtained (Table 1, Entries 9 and 10). Next, a survey of other solvents was then carried out (Table 1, Entries 11–14). To our delight, the yield could be further increased to 77% by using DMSO as the solvent (Table 1, Entry 11). Eventually, when 2 equiv. of BQ was used, the reaction gave the desired product in excellent yield (81%, Table 1, Entry 15).

With the optimized reaction conditions in hand, we turned to explore the substrate scope of this reaction. The reactions of *N*-(2-allylphenyl) benzamides with substituent (**R**) at the positions of benzamide aryl group were initially examined. As shown in Table 2, all substrates proceeded smoothly to afford the corresponding indole in moderate to good yields (62–90%). In general, better yields were found for substrates with electron-rich (**2g**, **2h**, **2i**, and **2u**) rather than electron-poor anilides (**2b**, **2c**, **2d**, **2e**, and **2f**). Prolonged reaction times were required for substrates with the latter substituents (**2b**, **2c**, and **2d**). Substrates **2b**, **2c**, and **2d** with Cl substituent at the *meta*-, *ortho*-, and *para*- position of the benzamide aryl group, respectivelu, were also studied. The results indicate that a relatively lower yield was observed for **2b** with *ortho*-Cl comparing with **2c** and **2d**. Additionally, products with substituents at the *meta*-position (**2d**, **2e**, and **2i**) can be obtained in higher yields than those with *para*-substituents (**2c**, **2f**, and **2h**). Similarly, indoles with two substituents on the phenyl ring (**2j** and **2u**) were also obtained in pretty good yield.

Subsequently, we investigated the effects of substituents (**R^1^**) residing on the aromatic moiety of the *N*-(2-allylphenyl) benzamide (Table 3). All the substrates **1k**–**t** gave the desired products **2k**–**t** in satisfactory yields (67–78%), but 48 h were required for the starting materials to be consumed completely in most cases. The reaction yields were not significantly influenced by the electronic properties of **R^1^** substituent. The effect of the same substituted group at different position was also studied. Indoles with methyl substituent at C5 position (**2k** and **2l**) were obtained in slightly lower yields than at the C7 position (**2s** and **2t**), due to the steric of the 1-position of the indoline. Besides, a gram-scale reaction of **1q** (2.9 g, 10 mmol) was performed affording the product **2q** in an identical yield with the small-scale reaction (71% vs. 77%). In addition, the structure of **2r** was determined by X-ray crystallography [40].

To further examine the propensity for the reaction, indoles with different *N*-substituents were investigated (Table 4). Under the standard reaction conditions, the reaction proceeded smoothly and indoles bearing *N*-acetyl (**2v**) and *N*-Ts (**2w**) were also obtained in 81% and 75% yields, respectively.

**Scheme 1:**
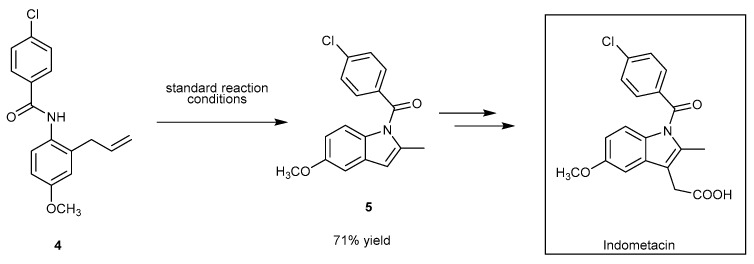
Synthesis of indomethacin.

To evaluate the synthetic utility of this novel method, we used it as the key step to build up the scaffold of the nonsteroidal anti-inflammatory drug molecule indomethacin (Scheme 1). When substrate **4** was subjected to the standard reaction conditions, the desired product **5** was obtained in 71% yield, which is the key intermediate of indomethacin. The following two steps to the final product indomethacin are described in a previous report [36]. Although indomethacin derivatives can be synthesized using Fisher indole synthesis and other cyclization methods, this methodology offers an alternative way to synthesize the key intermediate substituted *N*-benzoylindole **5** in one step, which is crucial to further diversity-oriented synthesis of analogs of indomethacin derivatives.

**Scheme 2:**
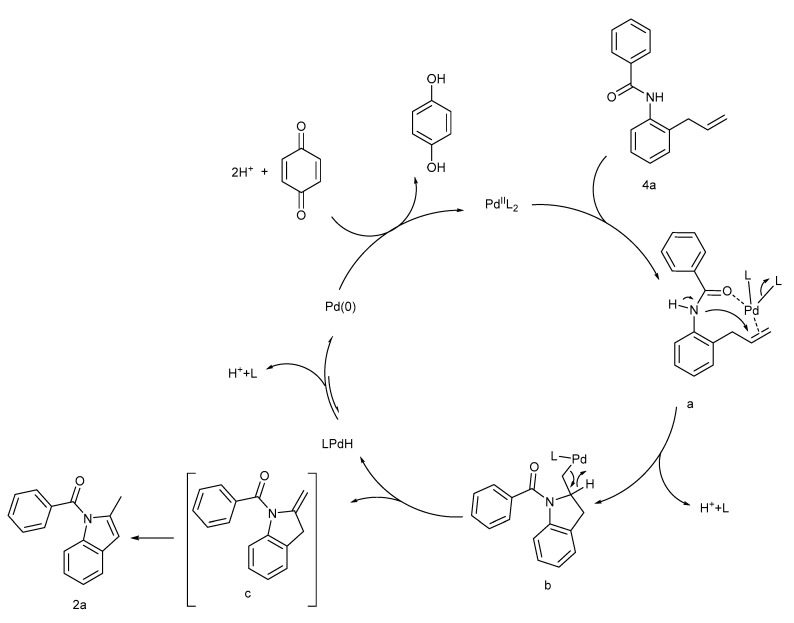
Proposed Mechanism.

On the basis of the previous studies, a plausible reaction mechanism is proposed (Scheme 2). Initially, the Pd^II^ catalyst first coordinates to the olefin to generate an intermediate **a**, followed by insertion of the alkene into the Pd^II^-N bond in an amidopalladation reaction to give an intermediate **b**. Subsequent β-hydride elimination from the resulting alkyl-Pd^II^ species affords the intermediate **c**, which undergoes spontaneous isomerization–aromatization to form product **2a** [12,31]. Pd(0), in equilibrium with LPdH, was then reoxidized by the action of BQ.

## 3. Materials and Methods

Column chromatography was performed on silica gel (Silica-P flash silica gel from Silicycle, size 40–63 μm). TLC was performed on silica gel 60/Kieselguhr F254. Mass spectra were recorded on an AEI-MS-902 mass spectrometer (EI+) or a LTQ Orbitrap XL (ESI+). ^1^H, ^13^C, and ^19^F NMR were recorded on a Varian AMX400 (400, 100.6, and 376 MHz, respectively) or a Varian Unity Plus Varian-500 (500, 125, and 471 MHz, respectively). Chemical shift values for ^1^H and ^13^C NMR are reported in ppm with the solvent resonance as the internal standard (CHCl_3_: δ 7.26 ppm for ^1^H, δ77.0 ppm for ^13^C). Data are reported as follows: chemical shifts, multiplicity (s = singlet, d = doublet, t = triplet, q = quartet, br = broad, m = multiplet), coupling constants (Hz), and integration. Melting points were determined on a Buchi B–545 melting point apparatus. All reactions were performed under anhydrous conditions and under N_2_ atmosphere. All chemicals used were of analytical grade and were used as received without any further purification. All anhydrous solvents used in reactions were purchased in SureSeal bottles or dried over molecular sieves. Flash column chromatography was performed on Biotage Isolelera One with prepacked columns.

## 4. Conclusions

In summary, we developed an effective method for the synthesis of substituted *N*-benzoylindoles. Pd(II)-catalyzed synthesis of substituted *N*-benzoylindole was realized for the first time via C–H activation, starting from readily available substituent *N*-(2-allylphenyl) benzamide. Using inexpensive BQ as the oxidant, a series of substituted indoles were prepared in good to excellent yields under mild reaction conditions, which overcome the formation of byproduct benzoxazine. It should be noted that dibutyl phosphate (DBP) is the key to obtaining high yield and chemoselectivity in the present reaction. The indoles can be readily converted to many useful skeletons. As an example, this method was successfully used for the synthesis of a key skeleton of indomethacin.

## Figures and Tables

**Table 1:**
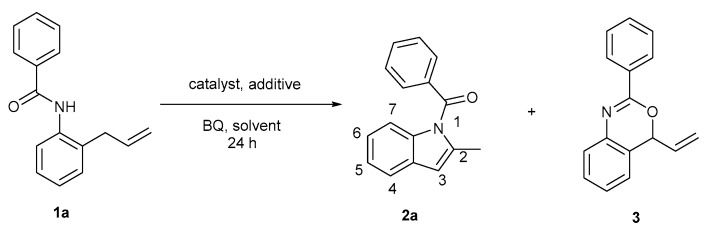
Optimization of the Reaction Conditions. ^a.^

Entry	Catalyst	Additive	Solvent	T [°C]	Yield ^b^ (2a:3) ^c^
**1**	Pd(OAc)_2_	-	CH_3_CN	RT	**8 (1:1)**
**2**	Pd(OAc)_2_	-	CH_3_CN	45	**26 (2:1)**
**3**	Pd(OAc)_2_	-	CH_3_CN	60	**33 (4:1)**
**4**	Pd(OAc)_2_	AcOH	CH_3_CN	60	**12 (10:1)**
**5**	Pd(OAc)_2_	PhCOOH	CH_3_CN	60	**22 (10:1)**
**6**	Pd(OAc)_2_	TFA	CH_3_CN	60	**26 (9:1)**
**7**	Pd(OAc)_2_	Ph_2_PO_2_H	CH_3_CN	60	**36 (15:1)**
**8**	Pd(OAc)_2_	(BuO)_2_PO_2_H	CH_3_CN	60	**71 (>20:1)**
**9**	PdCl_2_	(BuO)_2_PO_2_H	CH_3_CN	60	**23 (>20:1)**
**10**	White catalyst ^d^	(BuO)_2_PO_2_H	CH_3_CN	60	**9 (>20:1)**
**11**	Pd(OAc)_2_	(BuO)_2_PO_2_H	DMSO	60	**77 (>20:1)**
**12**	Pd(OAc)_2_	(BuO)_2_PO_2_H	dioxane	60	**52 (>20:1)**
**13**	Pd(OAc)_2_	(BuO)_2_PO_2_H	THF	60	**45 (>20:1)**
**14**	Pd(OAc)_2_	(BuO)_2_PO_2_H	DMF	60	**36 (>20:1)**
**15**	**Pd(OAc)_2_**	**(BuO)_2_PO_2_H**	**DMSO**	**60**	**81 (>20:1)**

^a^ Reaction conditions: **1a** (0.2 mmol), Pd(II) catalyst (10 mol%), additive (1.5 equiv.), BQ (Entries 1–14,1.5 equiv.; Entry 15, 2.0 equiv.), solvent (2.0 mL), 24 h; ^b^ Isolated yield of **2a**; ^c^ Determined by ^1^H NMR analysis of the crude residue. ^d^ 1,2-Bis(phenylsulfinyl)ethane palladium(II) acetate.

**Table 2:** Substrate scope of substrates with substituents at the positions (R) ^a,b^ Conditions: See Supplementary Materials for details.

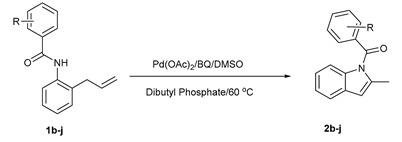
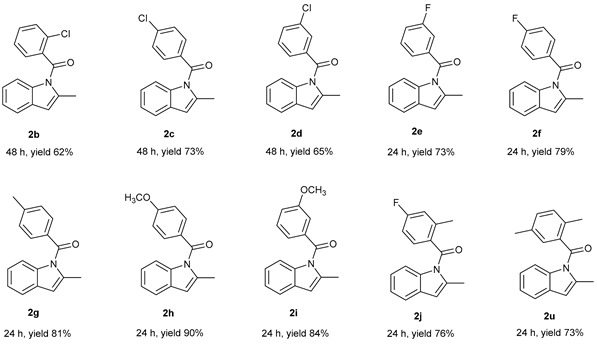

^a^ All reactions were carried out in 0.2 mmol scale. ^b^ Yields referred to here are isolated yields.

**Table 3:** Substrate scope of substrates with substituents at the positions (R^1^) ^a,b^ Conditions: See Supplementary Materials for details.

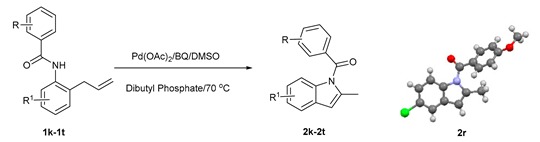
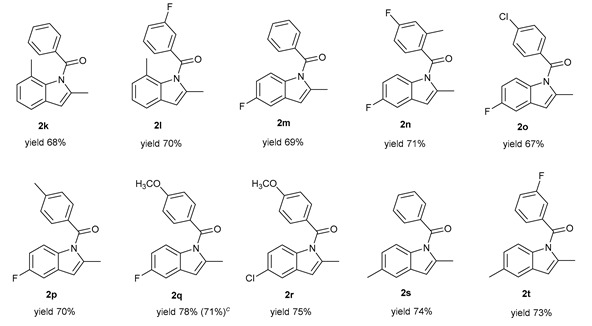

^a^ All reactions were carried out in 0.2 mmol scale. ^b^ Isolated yields. ^c^ Yield of the scale-up reaction (2.9 g, 10 mmol).

**Table 4:** Indoles with different *N*-substituents. ^a,b^ Conditions: See Supplementary Materials for details.

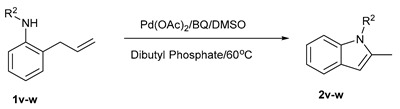
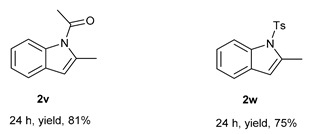

^a^ All reactions were carried out in 0.2 mmol scale. ^b^ Yields referred to here are isolated yields.

## Supplementary Materials

The following are available online, ^1^H, ^13^C, and ^19^F-NMR spectra of compounds **1a**–**1w**, **2a**–**2w**, **4** and **5**.

## References

[1] Somei M., Yamada F. (2004). Simple indole alkaloids and those with a nonrearranged monoterpenoid unit. Nat. Prod. Rep..

[2] Kawasaki T., Higuchi K. (2005). Simple indole alkaloids and those with a nonrearranged monoterpenoid unit. Nat. Prod. Rep..

[3] Kochanowska-Karamyan A.J., Hamann M.T. (2010). Marine indole alkaloids: Potential new drug leads for the control of depression and anxiety. Chem. Rev..

[4] Randriambola L., Quirion J.C., Kan-Fan C., Husson H.P. (1987). Structure of goniomitine, a new type of indole alkaloid. Tetrahedron Lett..

[5] Kaushik N.K., Kaushik N., Attri P., Kumar N., Kim C.H., Verma A.K., Choi E.H. (2013). Biomedical importance of indoles. Molecules.

[6] Fischer E., Jourdan F. (1883). Ueber die Hydrazine der Brenztraubensä ure. Ber. Dtsch. Chem. Ges..

[7] Larock R.C., Yum E.K. (1991). Synthesis of Indoles Via Palladium-Catalyzed Heteroannulation of Internal Alkynes. J. Am. Chem. Soc..

[8] Larock R.C., Yum E.K., Refvik M.D. (1998). Synthesis of 2,3-disubstituted indoles via palladium-catalyzed annulation of internal alkynes. J. Org. Chem..

[9] Wagaw S., Yang B.H., Buchwald S.L. (1998). A palladium-catalyzed strategy for the preparation of indoles: A novel entry into the Fischer indole synthesis. J. Am. Chem. Soc..

[10] Wagaw S., Yang B.H., Buchwald S.L. (1999). A palladium-catalyzed method for the preparation of indoles via the Fischer indole synthesis. J. Am. Chem. Soc..

[11] Rutherford J.L., Rainka M.P., Buchwald S.L. (2002). An annulative approach to highly substituted indoles: Unusual effect of phenolic additives on the success of the arylation of ketone enolates. J. Am. Chem. Soc..

[12] Hegedus L.S., Allen G.F., Waterman E.L. (1976). Palladium assisted intramolecular amination of olefins. A new synthesis of indoles. J. Am. Chem. Soc..

[13] Hegedus L.S., Allen G.F., Bozell J.J., Waterman E.L. (1978). Palladium-assisted intramolecular amination of olefins. Synthesis of nitrogen heterocycles. J. Am. Chem. Soc..

[14] Bozell J.J., Hegedus L.S. (1981). Palladium-Assisted Functionalization of Olefins-a New Amination of Electron-Deficient Olefins. J. Org. Chem..

[15] Louis S.H. (1988). Transition Metals in the Synthesis and Functionaiization of Indoles. Angew. Chem. Int. Ed..

[16] Stuart D.R., Bertrand-Laperle M., Burgess K.M., Fagnou K. (2008). Indole synthesis via rhodium catalyzed oxidative coupling of acetanilides and internal alkynes. J. Am. Chem. Soc..

[17] Bernini R., Fabrizi G., Sferrazza A., Cacchi S. (2009). Copper-catalyzed C-C bond formation through C-H functionalization: Synthesis of multisubstituted indoles from N-aryl enaminones. Angew. Chem. Int. Ed..

[18] Guan Z.H., Yan Z.Y., Ren Z.H., Liu X.Y., Liang Y.M. (2010). Preparation of indoles via iron catalyzed direct oxidative coupling. Chem. Commun..

[19] Stuart D.R., Alsabeh P., Kuhn M., Fagnou K. (2010). Rhodium(III)-catalyzed arene and alkene C-H bond functionalization leading to indoles and pyrroles. J. Am. Chem. Soc..

[20] Tan Y., Hartwig J.F. (2010). Palladium-catalyzed amination of aromatic C-H bonds with oxime esters. J. Am. Chem. Soc..

[21] Huestis M.P., Chan L., Stuart D.R., Fagnou K. (2011). The vinyl moiety as a handle for regiocontrol in the preparation of unsymmetrical 2,3-aliphatic-substituted indoles and pyrroles. Angew. Chem. Int. Ed..

[22] Neumann J.J., Rakshit S., Droge T., Wurtz S., Glorius F. (2011). Exploring the oxidative cyclization of substituted N-aryl enamines: Pd-catalyzed formation of indoles from anilines. Chem. Eur. J..

[23] Wang Y., Ye L., Zhang L. (2011). Au-catalyzed synthesis of 2-alkylindoles from N-arylhydroxylamines and terminal alkynes. Chem. Commun..

[24] Wei Y., Deb I., Yoshikai N. (2012). Palladium-catalyzed aerobic oxidative cyclization of N-aryl imines: Indole synthesis from anilines and ketones. J. Am. Chem. Soc..

[25] Song W., Ackermann L. (2013). Nickel-catalyzed alkyne annulation by anilines: Versatile indole synthesis by C-H/N-H functionalization. Chem. Commun..

[26] Wang C., Sun H., Fang Y., Huang Y. (2013). General and efficient synthesis of indoles through triazene-directed C-H annulation. Angew. Chem. Int. Ed..

[27] Tremont S.J., Rahman H.U. (1984). Ortho-Alkylation of Acetanilides Using Alkyl-Halides and Palladium Acetate. J. Am. Chem. Soc..

[28] Fix S.R., Brice J.L., Stahl S.S. (2002). Efficient intramolecular oxidative amination of olefins through direct dioxygen-coupled palladium catalysis. Angew. Chem. Int. Ed..

[29] Nallagonda R., Rehan M., Ghorai P. (2014). Synthesis of functionalized indoles via palladium-catalyzed aerobic oxidative cycloisomerization of o-allylanilines. Org. Lett..

[30] Ning X.S., Wang M.M., Qu J.P., Kang Y.B. (2018). Synthesis of Functionalized Indoles via Palladium-Catalyzed Aerobic Cycloisomerization of o-Allylanilines Using Organic Redox Cocatalyst. J. Org. Chem..

[31] Savvidou A., IoannisTzaras D., Koutoulogenis G.S., Theodorou A., Kokotos C.G. (2019). Synthesis of Benzofuran and Indole Derivatives Catalyzed by Palladium on Carbon. Eur. J. Org. Chem..

[32] Cajaraville A., Lopez S., Varela J.A., Saa C. (2013). Rh(III)-catalyzed tandem C-H allylation and oxidative cyclization of anilides: A new entry to indoles. Org. Lett..

[33] Kim M., Park J., Sharma S., Han S., Han S.H., Kwak J.H., Jung Y.H., Kim I.S. (2013). Synthesis and C2-functionalization of indoles with allylic acetates under rhodium catalysis. Org. Biomol. Chem..

[34] Gong T.-J., Cheng W.-M., Su W., Xiao B., Fu Y. (2014). Synthesis of indoles through Rh(III)-catalyzed C–H cross-coupling with allyl carbonates. Tetrahedron Lett..

[35] Andries-Ulmer A., Brunner C., Rehbein J., Gulder T. (2018). Fluorine as a Traceless Directing Group for the Regiodivergent Synthesis of Indoles and Tryptophans. J. Am. Chem. Soc..

[36] Xia H.D., Zhang Y.D., Wang Y.H., Zhang C. (2018). Water-Soluble Hypervalent Iodine(III) Having an I-N Bond. A Reagent for the Synthesis of Indoles. Org. Lett..

[37] McDonald R.I., Stahl S.S. (2010). Modular synthesis of 1,2-diamine derivatives by palladium-catalyzed aerobic oxidative cyclization of allylic sulfamides. Angew. Chem. Int. Ed..

[38] Strambeanu I.I., White M.C. (2013). Catalyst-controlled C-O versus C-N allylic functionalization of terminal olefins. J. Am. Chem. Soc..

[39] Osberger T.J., White M.C. (2014). N-Boc amines to oxazolidinones via Pd(II)/bis-sulfoxide/Bronsted acid co-catalyzed allylic C-H oxidation. J. Am. Chem. Soc..

[B40-molecules-25-01233] 40.CCDC 1894284 contains the supplementary crystallographic data for **2r**. These data can be obtained free of charge from The Cambridge Crystallographic Data Centre via www.ccdc.czm.ac.uk/datarequest/cif.

