# Supplementary material for: Synthesis of Functionalized Indoles via Palladium-Catalyzed Cyclization of N-(2-allylphenyl) Benzamide: A Method for Synthesis of Indomethacin Precursor
Source: Molecules. 2020 Mar 9;25(5):1233. doi: 10.3390/molecules25051233 (PMC7179414; doi:10.3390/molecules25051233)
Supplement: Supplementary file 1 [file molecules-25-01233-s001.pdf]

Supplementary information for

Synthesis of Functionalized Indoles via Palladium-Catalyzed Cyclization of *N*-(2-allylphenyl) Benzamide: A method for Synthesis of Indometacin Precursor

Zhe Chang [1], Tong Ma [1], Yu Zhang [1], Zheng Dong [1], Heng Zhao [1] and Depeng Zhao [1,*]

1 School of Pharmaceutical Sciences, Sun Yat-sen University, Guangzhou 510006, China；

Table of Contents

1. General remarks………………………………………………………………………. 2

2. General procedure for synthesis of substrates……………………………………… 3

3. Characterization of substrates………………………………………………………. 4-18

4. Typical experimental procedure …………………………………………………… 19

5. Characterization of the products……………………………………………………. 19-28

6. NMR Spectra ………………………………………………………………………… 29-88

1. General remarks

Column chromatography was performed on silica gel (Silica-P flash silica gel from Silicycle, size 40-63 μm). TLC was performed on silica gel 60/ Kieselguhr F254. Mass spectra were recorded on a AEI-MS-902 mass spectrometer (EI+) or a LTQ Orbitrap XL (ESI+). ^{1}H, ^{13}C, ^{19}F NMR were recorded on a Varian AMX400 (400, 100.6 and 376 MHz, respectively) or a Varian Unity Plus Varian-500 (500, 125 and 471 MHz, respectively). Chemical shift values for ^{1}H and ^{13}C NMR are reported in ppm with the solvent resonance as the internal standard ($CHCl_3$: δ 7.26 ppm for ^{1}H, δ77.0 ppm for ^{13}C). Data are reported as follows: chemical shifts, multiplicity (s = singlet, d = doublet, t = triplet, q = quartet, br = broad, m = multiplet), coupling constants (Hz), and integration. Melting points were determined on a Buchi B–545 melting point apparatus. All reactions were performed under anhydrous conditions and under N_2 atmosphere. All chemicals used were of analytical grade and were used as received without any further purification. All anhydrous solvents used in reactions were purchased in SureSeal bottles or dried over molecular sieves. Flash column chromatography was performed on Biotage Isolelera One with prepacked columns.

2. General procedure for synthesis of substrates

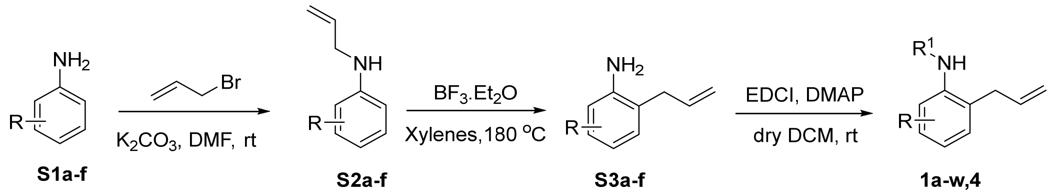

Typical procedure:

Allyl bromide (180 mmol) was added dropwise to a solution of commercially available **S1a-f** (200 mmol) and K_2CO_3 (300 mmol) in DMF (40 mL). The solution was stirred at room temperature overnight. The reaction mixture was then extracted with EtOAc (2x100 mL), washed with H_2O (3x50 mL) and brine (50 mL), dried over Na_2SO_4 and concentrated in vacuo. The crude product was purified by column chromatography (eluting with petroleum ether) to afford **S2a-f**.

$BF_3 \cdot OEt_2$ (76.8 mmol) was added to a solution of **S2a-f** (64 mmol) in xylene (20 mL) at -10 °C under N_2 atmosphere. The mixture was heated to 160 ºC in a thick-walled glass pressure tube and stirred at this temperature for 8 hours. After cooling, the reaction mixture was poured into 2M NaOH (100 mL) at 0 °C, and extracted with EtOAc (3x150 mL). The combined organic extracts were washed with brine (100 mL), dried over Na_2SO_4, and concentrated in vacuo. The crude product was purified by column chromatography (eluting with petroleum ether/ethyl acetate 3%) to yield **S3a-f**.

To a solutions of benzoic acid (10 mmol) in DCM (10 mL) were added the **S3a-f** (10 mmol), DMAP (20 mmol) and EDCI (20 mmol), and stirring was carried out overnight at room temperature. The reaction mixture was then extracted with DCM (2x30 mL), washed with saturated sodium bicarbonate (30 mL) and brine (30 mL), dried over Na_2SO_4 and concentrated in vacuo. The crude product was purified by column chromatography (eluting with petroleum ether/ethyl acetate 5-10%) to afford **1a-w, 4**.

3. Characterization of substrates

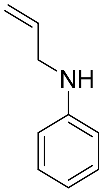

S2a: pale yellow liquid; yield 60%.

^{1}H NMR (400 MHz, $CDCl_3$) δ 7.19 (t, *J* = 7.8 Hz, 2H), 6.73 (t, *J* = 7.3 Hz, 1H), 6.64 (d, *J* = 8.2 Hz, 2H), 5.98 (m, 1H), 5.30 (dd, *J* = 17.2, 1.3 Hz, 1H), 5.18 (dd, *J* = 10.3, 0.9 Hz, 1H), 3.79 (d, *J* = 5.3 Hz, 3H).

^{13}C NMR (101 MHz, $CDCl_3$) δ 148.0, 135.4, 129.2, 117.5, 116.2, 112.9, 46.5.

HRMS (ESI+, *m/z*) calculated for $C_9H_{11}N$ $[M + H]^+$: 134.0964; found: 134.0965;.

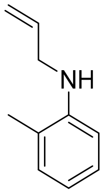

S2b: pale yellow liquid; yield 62%.

^{1}H NMR (400 MHz, $CDCl_3$) δ 7.23 (t, *J* = 7.7 Hz, 1H), 7.17 (d, *J* = 7.2 Hz, 1H), 6.78 (t, *J* = 7.4 Hz, 1H), 6.72 (d, *J* = 8.0 Hz, 1H), 6.10 (m, 1H), 5.40 (dd, *J* = 17.2, 1.5 Hz, 1H), 5.29 (dd, *J* = 10.3, 1.4 Hz, 1H), 3.92 (d, *J* = 5.3 Hz, 2H), 3.71 (s, 1H), 2.26 (s, 3H).

^{13}C NMR (101 MHz, $CDCl_3$) δ 145.9, 135.5, 130.0, 127.0, 121.8, 117.0, 116.1, 109.9, 46.4, 17.4.

HRMS (ESI+, *m/z*) calculated for $C_{10}H_{13}N$ $[M + H]^+$: 148.1121; found: 148.1124;.

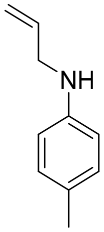

S2c: pale yellow liquid; yield 29%.

^{1}H NMR (400 MHz, $CDCl_3$) δ 7.02 (d, *J* = 8.2 Hz, 2H), 6.58 (d, *J* = 8.3 Hz, 2H), 5.98 (m, 1H), 5.30 (dd, *J* = 17.2, 1.3 Hz, 1H), 5.18 (d, *J* = 10.3 Hz, 1H), 3.77 (d, *J* = 5.3 Hz, 2H), 3.65 (s, 1H), 2.27 (s, 3H).

^{13}C NMR (126 MHz, $CDCl_3$) δ 145.7, 135.6, 129.6, 126.6, 115.9, 113.1, 46.8, 20.3.

HRMS (ESI+, *m/z*) calculated for $C_{10}H_{13}N$ $[M + H]^+$: 148.1121; found: 148.1121;.

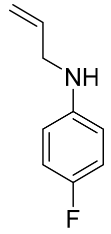

S2d: yellow liquid; yield 48%.

^{1}H NMR (400 MHz, $CDCl_3$) δ 6.98 – 6.86 (m, 2H), 6.61 – 6.52 (m, 2H), 5.97 (ddt, *J* = 17.0, 10.5, 5.3 Hz, 1H), 5.30 (dd, *J* = 17.2, 1.5 Hz, 1H), 5.20 (dd, *J* = 10.3, 1.3 Hz, 1H), 3.75 (d, *J* = 5.4 Hz, 2H), 3.69 (s, 1H).

^{13}C NMR (101 MHz, $CDCl_3$) δ155.8 (d, *J* = 235.3 Hz), 144.3, 135.3, 116.2, 115.5 (d, *J* = 22.2 Hz), 113.7 (d, *J* = 7.4 Hz), 47.0.

^{19}F NMR (376 MHz, $CDCl_3$) δ -128.05.

HRMS (ESI+, *m/z*) calculated for $C_9H_{10}FN$ $[M + H]^+$: 152.0870; found: 152.0868;.

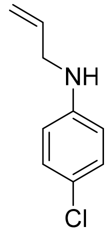

S2e: yellow liquid; yield 55%.

^{1}H NMR (500 MHz, $CDCl_3$) δ 7.18 – 6.99 (m, 2H), 6.60 – 6.50 (m, 2H), 5.93 (m, 1H), 5.28 (dd, *J* = 17.2, 1.2 Hz, 1H), 5.18 (dd, *J* = 10.3, 0.9 Hz, 1H), 3.81 (s, 1H), 3.75 (d, *J* = 5.3 Hz, 2H).

^{13}C NMR (126 MHz, $CDCl_3$) δ 146.5, 134.9, 129.0, 122.0, 116.4, 114.0, 46.5.

HRMS (ESI+, *m/z*) calculated for $C_9H_{10}C1N$ $[M + H]^+$: 168.0575; found: 168.0579;.

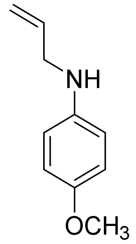

S2f: yellow liquid; yield 57%

^{1}H NMR (400 MHz, $CDCl_3$) δ 6.81 (d, *J* = 8.9 Hz, 2H), 6.62 (d, *J* = 8.9 Hz, 2H), 5.98 (m, 1H), 5.30 (dd, *J* = 17.2, 1.5 Hz, 1H), 5.18 (dd, *J* = 10.3, 1.2 Hz, 1H), 3.77 (s, 3H), 3.75 (d, *J* = 5.5 Hz, 2H), 3.46 (s, 1H).

^{13}C NMR (101 MHz, $CDCl_3$) δ 152.1, 142.2, 135.7, 116.0, 114.8, 114.2, 55.7, 47.4.

HRMS (ESI+, *m/z*) calculated for $C_{10}H_{13}NO$ [M + H]$^+$: 164.1070; found: 164.1067;.

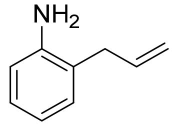

S3a: yellow liquid; yield 33%.

^{1}H NMR (500 MHz, $CDCl_3$) δ 7.17-7.12 (m, 2H), 6.84 (t, *J* = 7.4 Hz, 1H), 6.74 (d, *J* = 7.9 Hz, 1H), 6.03 (dq, *J* = 11.1, 6.2 Hz, 1H), 5.20 (t, *J* = 12.8 Hz, 2H), 3.72 (s, 2H), 3.38 (d, *J* = 6.2 Hz, 2H).

^{13}C NMR (126 MHz, $CDCl_3$) δ 144.7, 135.8, 130.0, 127.4, 123.8, 118.7, 115.9, 115.6, 36.3.

HRMS (ESI+, *m/z*) calculated for $C_9H_{11}N$ [M + H]$^+$: 134.0964; found: 134.0963;.

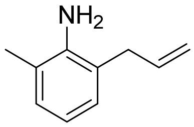

S3b: yellow liquid; yield 40%.

^{1}H NMR (400 MHz, $CDCl_3$) δ 7.06 (d, *J* = 7.4 Hz, 1H), 7.02 (d, *J* = 7.4 Hz, 1H), 6.76 (t, *J* = 7.5 Hz, 1H), 6.12 – 5.94 (m, 1H), 5.26 – 5.09 (m, 2H), 3.70 (s, 2H), 3.39 (d, *J* = 6.2 Hz, 2H), 2.25 (s, 3H).

^{13}C NMR (101 MHz, $CDCl_3$) δ 142.8, 136.0, 128.7, 127.9, 123.2, 122.2, 118.0, 115.9, 36.6, 17.5.

HRMS (ESI+, *m/z*) calculated for $C_{10}H_{13}N$ [M + H]$^+$: 148.1121; found: 148.1118;.

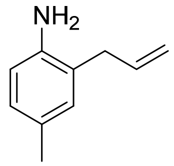

S3c: yellow liquid; yield 76%.

^{1}H NMR (500 MHz, $CDCl_3$) δ 6.98-6.96 (m, 2H), 6.67 (d, *J* = 7.7 Hz, 1H), 6.04 (dq, *J* = 11.8, 6.2 Hz, 1H), 5.25 – 5.16 (m, 2H), 3.60 (s, 2H), 3.36 (d, *J* = 6.2 Hz, 2H), 2.34 (s, 3H).

^{13}C NMR (126 MHz, $CDCl_3$) δ 142.1, 135.9, 130.6, 127.8, 127.8, 123.9, 115.8, 115.8, 36.3, 20.3.

HRMS (ESI+, *m/z*) calculated for $C_{10}H_{13}N$ [M + H]$^+$: 148.1121; found: 148.1121;.

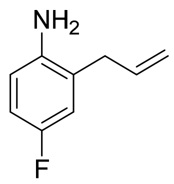

S3d: yellow liquid; yield 46%.

^{1}H NMR (400 MHz, $CDCl_3$) δ 6.80-6.75 (m, 2H), 6.61 (dd, *J* = 8.1, 4.9 Hz, 1H), 5.93 (ddt, *J* = 16.6, 10.2,

6.2 Hz, 1H), 5.17-5.08 (m, 2H), 3.53 (s, 2H), 3.27 (d, *J* = 6.1 Hz, 2H).

^{13}C NMR (101 MHz, $CDCl_3$) δ 156.4 (d, *J* = 236.3 Hz), 140.6 (d, *J* = 2.1 Hz), 135.0, 125.6 (d, *J* = 6.8 Hz), 116.6, 116.5 (d, *J* = 3.0 Hz), 116.3 (d, *J* = 18.2 Hz), 113.6 (d, *J* = 22.2 Hz), 36.2.

^{19}F NMR (376 MHz, $CDCl_3$) δ -126.49.

HRMS (ESI+, *m/z*) calculated for $C_9H_{10}FN$ $[M + H]^+$: 152.0870; found: 152.0871;.

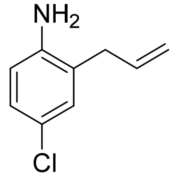

S3e: yellow liquid; yield 24%.

^{1}H NMR (400 MHz, $CDCl_3$) δ 7.03-7.01 (m, 2H), 6.64 – 6.51 (m, 1H), 5.92 (ddt, *J* = 16.3, 10.1, 6.2 Hz, 1H), 5.18-5.08 (m, 2H), 3.65 (s, 2H), 3.26 (d, *J* = 6.1 Hz, 2H).

^{13}C NMR (101 MHz, $CDCl_3$) δ 143.3, 134.9, 129.7, 127.2, 125.6, 123.2, 116.8, 116.7, 36.1.

HRMS (ESI+, *m/z*) calculated for $C_9H_{10}ClN$ $[M + H]^+$: 168.0575; found: 168.0571;.

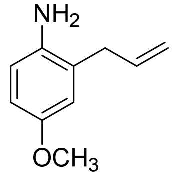

S3f: yellow liquid; yield 76%.

^{1}H NMR (400 MHz, $CDCl_3$) δ 6.69-6.62 (m, 3H), 5.96 (dq, *J* = 11.3, 6.2 Hz, 1H), 5.13 (t, *J* = 12.8 Hz, 2H), 3.76 (s, 3H), 3.36 (s, 2H), 3.30 (d, *J* = 6.0 Hz, 2H).

^{13}C NMR (101 MHz, $CDCl_3$) δ 152.7, 138.2, 135.6, 125.5, 116.8, 116.0, 115.8, 112.5, 55.5, 36.4.

HRMS (ESI+, *m/z*) calculated for $C_{10}H_{13}NO$ $[M + H]^+$: 164.1070; found: 164.1078;.

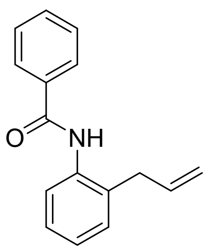

1a: white solid; yield 76%, m.p. = 107-109 °C.
^{1}H NMR (500 MHz, $CDCl_3$) δ 8.09 – 7.98 (m, 2H), 7.86 (d, *J* = 7.7 Hz, 2H), 7.55 (t, *J* = 7.2 Hz, 1H), 7.49 (t, *J* = 7.4 Hz, 2H), 7.32 (t, *J* = 7.7 Hz, 1H), 7.22 (t, *J* = 7.2 Hz, 1H), 7.16 (t, *J* = 7.4 Hz, 1H), 6.04 (ddt, *J* = 16.2, 10.2, 6.0 Hz, 1H), 5.24 (d, *J* = 10.1 Hz, 1H), 5.12 (d, *J* = 17.2 Hz, 1H), 3.46 (d, *J* = 5.7 Hz, 2H).

^{13}C NMR (126 MHz, $CDCl_3$) δ 165.4, 136.1, 134.8, 131.7, 130.3, 130.0, 128.7, 128.5, 127.5, 127.0, 125.3, 123.5, 116.8, 36.9.

HRMS (ESI+, *m/z*) calculated for $C_{16}H_{15}NO$ $[M + H]^+$: 238.1226; found: 238.1227;.

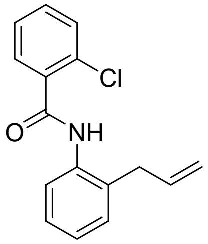

1b: white solid; yield 51%, m.p. = 97-99 °C.

^1H NMR (500 MHz, $CDCl_3$) δ 8.04 (d, *J* = 8.0 Hz, 1H), 7.89 (s, 1H), 7.74 (d, *J* = 7.3 Hz, 1H), 7.49 – 7.30 (m, 4H), 7.25 – 7.15 (m, 2H), 5.98 (ddt, *J* = 16.2, 10.2, 6.0 Hz, 1H), 5.11 (d, *J* = 10.1 Hz, 1H), 5.00 (d, *J* = 17.2 Hz, 1H), 3.46 (d, *J* = 5.6 Hz, 2H).

^{13}C NMR (126 MHz, $CDCl_3$) δ 164.6, 135.9, 135.6, 135.3, 131.6, 130.5, 130.5, 130.3, 130.3, 130.2, 127.5, 127.2, 125.8, 123.8, 116.7, 36.5.

HRMS (ESI+, *m/z*) calculated for $C_{16}H_{14}ClNO$ $[M + H]^+$: 277.0837; found: 272.0833;.

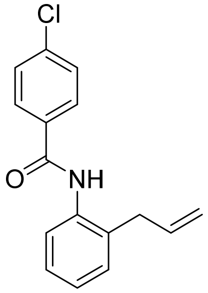

1c: white solid; yield 66%, m.p. = 120-122 °C.

^1H NMR (500 MHz, $CDCl_3$) δ 7.99 (s, 1H), 7.79 (d, *J* = 7.8 Hz, 1H), 7.64 (d, *J* = 8.2 Hz, 2H), 7.29 (d, *J* = 8.3 Hz, 2H), 7.17 (t, *J* = 7.6 Hz, 1H), 7.10 (d, *J* = 7.4 Hz, 1H), 7.05 (t, *J* = 7.4 Hz, 1H), 5.89 (ddt, *J* = 16.2, 10.2, 6.0 Hz, 1H), 5.09 (d, *J* = 10.1 Hz, 1H), 4.97 (d, *J* = 17.2 Hz, 1H), 3.31 (d, *J* = 5.8 Hz, 2H).

^{13}C NMR (126 MHz, $CDCl_3$) δ 164.5, 137.9, 136.2, 135.9, 133.1, 130.5, 130.3, 128.8, 128.4, 127.4, 125.6, 123.8, 116.7, 36.8.

HRMS (ESI+, *m/z*) calculated for $C_{16}H_{14}ClNO$ $[M + H]^+$: 272.0837; found: 272.0835;.

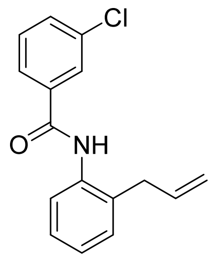

1d: white solid; yield 82%, m.p. = 68-70 °C.

^{1}H NMR (500 MHz, $CDCl_3$) δ 8.08 (s, 1H), 7.95 (d, *J* = 7.9 Hz, 1H), 7.85-7.84 (m, 1H), 7.70 (d, *J* = 7.7 Hz, 1H), 7.51-7.49 (m, 1H), 7.39 (t, *J* = 7.9 Hz, 1H), 7.32 – 7.28 (m, 1H), 7.23-7.21 (m, 1H), 7.18-7.15(m, 1H), 6.02 (dd, *J* = 17.2, 10.2 Hz, 1H), 5.24 (ddd, *J* = 10.1, 3.0, 1.5 Hz, 1H), 5.12 (ddd, *J* = 17.2, 3.4, 1.7 Hz, 1H), 3.44 (d, *J* = 6.0 Hz, 2H).

^{13}C NMR (126 MHz, $CDCl_3$) δ 164.1, 136.5, 136.2, 135.8, 134.8, 131.7, 130.3, 130.3, 129.9, 127.5, 127.5, 125.6, 124.9, 123.6, 116.9, 37.0.

HRMS (ESI+, *m/z*) calculated for $C_{16}H_{14}ClNO$ $[M + H]^+$: 272.0837; found: 272.0833;

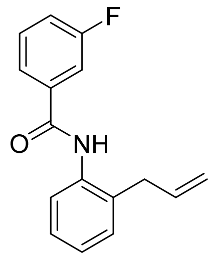

1e: white solid; yield 64%, m.p. = 91-93 °C.

^{1}H NMR (500 MHz, $CDCl_3$) δ 8.03 (d, *J* = 7.8 Hz, 1H), 7.97 (s, 1H), 7.59 (t, *J* = 8.9 Hz, 2H), 7.48-7.44 (m, 1H), 7.33 (t, *J* = 7.7 Hz, 1H), 7.26-7.23 (m, 2H), 7.17 (t, *J* = 7.4 Hz, 1H), 6.05 (ddt, *J* = 16.2, 10.2, 6.0 Hz, 1H), 5.26 (d, *J* = 10.1 Hz, 1H), 5.12 (d, *J* = 17.2 Hz, 1H), 3.46 (d, *J* = 5.8 Hz, 2H).

^{13}C NMR (126 MHz, $CDCl_3$) δ 164.1, 162.9 (d, *J* = 249.48 Hz), 137.2 (d, *J* = 6.8 Hz), 136.2, 136.0, 130.5, 130.4 (d, *J* = 7.6 Hz), 129.8, 127.7, 125.6, 123.4, 122.3 (d, *J* = 2.5 Hz), 118.9 (d, *J* = 21.4 Hz), 117.0, 114.5 (d, *J* = 22.7 Hz), 37.1.

^{19}F NMR (471 MHz, $CDCl_3$) δ -111.33.

HRMS (ESI+, *m/z*) calculated for $C_{16}H_{14}FNO$ $[M + H]^+$: 256.1132; found: 256.1135;.

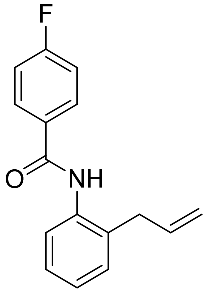

1f: white solid; yield 78%, m.p. = 98-100 °C.

^{1}H NMR (400 MHz, $CDCl_3$) δ 7.98 (d, *J* = 8.2 Hz, 1H), 7.96 (s, 1H), 7.89 – 7.82 (m, 2H), 7.35 – 7.28 (m, 1H), 7.24-7.21 (m, 1H), 7.18-7.13 (m, 3H), 6.04 (ddt, *J* = 16.2, 10.2, 6.0 Hz, 1H), 5.23 (dd, *J* = 10.1, 1.6 Hz, 1H), 5.10 (dd, *J* = 17.2, 1.7 Hz, 1H), 3.45 (d, *J* = 5.9 Hz, 2H).

^{13}C NMR (101 MHz, $CDCl_3$) δ 164.9 (d, *J* = 253.5 Hz), 164.4, 136.3, 136.1, 131.0 (d, *J* = 3.0 Hz), 130.4, 130.0, 129.3 (d, *J* = 9.0 Hz), 127.6, 125.5, 123.6, 116.8, 115.8 (d, *J* =22.2 Hz), 37.0.

^{19}F NMR (471 MHz, $CDCl_3$) δ -107.54.

HRMS (ESI+, *m/z*) calculated for $C_{16}H_{14}FNO$ $[M + H]^+$: 256.1132; found: 256.1130;.

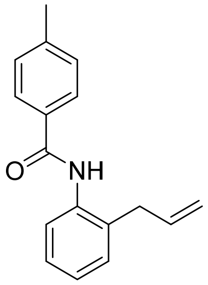

1g: white solid; yield 69%, m.p. = 110-112 °C.

^{1}H NMR (500 MHz, $CDCl_3$) δ 8.05 (d, *J* = 8.0 Hz, 1H), 7.98 (s, 1H), 7.75 (d, *J* = 7.9 Hz, 2H), 7.33-7.27 (m, 3H), 7.22 (d, *J* = 7.4 Hz, 1H), 7.14 (t, *J* = 7.4 Hz, 1H), 6.04 (ddt, *J* = 16.2, 10.2, 6.0 Hz, 1H), 5.24 (d, *J* = 10.1 Hz, 1H), 5.12 (d, *J* = 17.2 Hz, 1H), 3.45 (d, *J* = 5.8 Hz, 2H), 2.43 (s, 3H).

^{13}C NMR (126 MHz, $CDCl_3$) δ 165.4, 142.3, 136.4, 136.2, 132.0, 130.3, 129.8, 129.4, 127.5, 127.0, 125.1, 123.4, 116.8, 37.0, 21.4.

HRMS (ESI+, *m/z*) calculated for $C_{17}H_{17}NO$ $[M + H]^+$: 252.1383; found: 252.1381;.

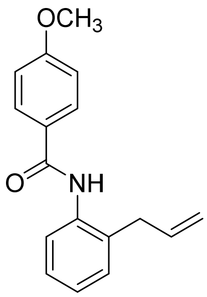

1h: white solid; yield 83%, m.p. = 123-125 °C.

^{1}H NMR (400 MHz, $CDCl_3$) δ 8.04 (d, *J* = 8.0 Hz, 1H), 7.90 (s, 1H), 7.86 – 7.76 (m, 2H), 7.36 – 7.28 (m, 1H), 7.22 (dd, *J* = 7.5, 1.4 Hz, 1H), 7.14 (td, *J* = 7.5, 1.1 Hz, 1H), 7.03 – 6.92 (m, 2H), 6.05 (ddt, *J* = 16.1, 10.2, 5.9 Hz, 1H), 5.29 – 5.20 (m, 1H), 5.12 (ddt, *J* = 16.2, 10.2, 6.0 Hz, 1H), 3.87 (d, *J* = 6.1 Hz, 3H), 3.46 (d, *J* = 5.9 Hz, 2H).

^{13}C NMR (126 MHz, $CDCl_3$) δ 165.0, 162.4, 136.5, 136.4, 130.3, 129.67, 128.8, 127.6, 127.1, 125.1, 123.4, 116.8, 113.9, 55.4, 37.1.

HRMS (ESI+, *m/z*) calculated for $C_{17}H_{17}NO_2$ $[M + H]^+$: 268.1332; found: 268.1331;.

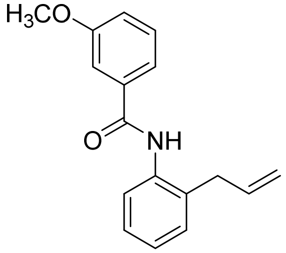

1i: pale yellow solid; yield 74%, m.p. = 64-66 °C.

1H NMR (500 MHz, $CDCl_3$) δ 8.05 (d, *J* = 8.0 Hz, 1H), 7.99 (s, 1H), 7.44 (s, 1H), 7.40 – 7.29 (m, 3H), 7.22 (d, *J* = 7.5 Hz, 1H), 7.15 (t, *J* = 7.4 Hz, 1H), 7.09 (d, *J* = 7.3 Hz, 1H), 6.10 – 5.97 (m, 1H), 5.24 (d, *J* = 10.1 Hz, 1H), 5.12 (d, *J* = 17.2 Hz, 1H), 3.87 (s, 3H), 3.46 (d, *J* = 5.7 Hz, 2H).

^{13}C NMR (126 MHz, $CDCl_3$) δ 165.3, 159.6, 136.0, 136.0, 135.9, 130.6, 130.0, 129.4, 127.1, 125.2, 123.7, 118.6, 117.7, 116.5, 112.2, 55.1, 36.5.

HRMS (ESI+, *m/z*) calculated for $C_{17}H_{17}NO_2$ $[M + H]^+$: 268.1332; found: 268.1330;.

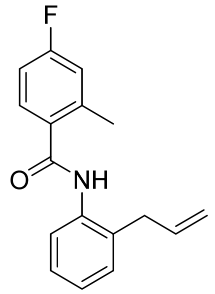

1j: white solid; yield 66%, m.p. = 132-133 °C.

^{1}H NMR (500 MHz, $CDCl_3$) δ 8.04 (d, *J* = 5.0 Hz, 1H), 7.55 (s, 1H), 7.46 (s, 1H), 7.32 (d, *J* = 6.8 Hz, 1H), 7.22 (d, *J* = 7.5 Hz, 1H), 7.16 (t, *J* = 7.4 Hz, 1H), 7.01 – 6.88 (m, 2H), 5.97 (ddt, *J* = 16.1, 10.2, 5.9 Hz, 1H), 5.14 (d, *J* = 10.1 Hz, 1H), 5.00 (d, *J* = 17.2 Hz, 1H), 3.42 (d, *J* = 5.7 Hz, 2H), 2.52 (s, 3H).

^{13}C NMR (126 MHz, $CDCl_3$) δ 167.1, 163.5 (d, *J* = 250.7 Hz), 140.1 (d, *J* = 8.2 Hz), 136.1, 136.0, 132.5, 130.4, 129.9, 128.7 (d, *J* = 8.8 Hz), 127.62, 125.6, 123.4, 118.2 (d, *J* = 21.4 Hz), 116.8, 112.8 (d, *J* = 21.4 Hz), 36.9, 20.1.

^{19}F NMR (471 MHz, $CDCl_3$) δ -110.27.

HRMS (ESI+, *m/z*) calculated for $C_{17}H_{16}FNO$ $[M + H]^+$: 270.1289; found: 270.1288;.

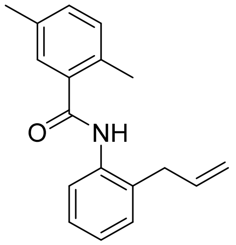

1u: white solid; yield 55%, m.p. = 140-142 °C.

^{1}H NMR (400 MHz, $CDCl_3$) δ 8.06 (d, *J* = 7.0 Hz, 1H), 7.59 (s, 1H), 7.35 – 7.27 (m, 2H), 7.24 – 7.20 (m, 1H), 7.15 (m, 3H), 5.98 (ddt, *J* = 16.3, 10.2, 6.0 Hz, 1H), 5.15 (dd, *J* = 10.1, 1.4 Hz, 1H), 5.02 (dd, *J* = 17.2, 1.6 Hz, 1H), 3.42 (d, *J* = 6.0 Hz, 2H), 2.48 (s, 3H), 2.36 (s, 3H).

^{13}C NMR (101 MHz, $CDCl_3$) δ 168.1, 136.1, 136.0, 135.4, 133.3, 131.2, 130.9, 130.2, 129.9, 127.5, 127.2, 125.3, 123.3, 116.6, 36.7, 20.8, 19.4.

HRMS (ESI+, *m/z*) calculated for $C_{18}H_{19}NO$ $[M + H]^+$: 266.1539; found: 266.1532;.

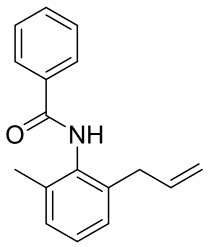

1k: white solid; yield 59%, m.p. = 137-139 °C.

^{1}H NMR (400 MHz, $CDCl_3$) δ 7.90 (d, *J* = 7.4 Hz, 2H), 7.68 (s, 1H), 7.57 (d, *J* = 7.3 Hz, 1H), 7.49-7.46 (m, 2H), 7.23 – 7.06 (m, 3H), 5.96 (ddt, *J* = 16.6, 10.1, 6.2 Hz, 1H), 5.09 (dd, *J* = 10.1, 1.1 Hz, 1H), 4.96 (dd, *J* = 17.1, 1.5 Hz, 1H), 3.38 (d, *J* = 6.2 Hz, 2H), 2.28 (s, 3H).

^{13}C NMR (101 MHz, $CDCl_3$) δ 165.7, 136.9, 136.3, 134.3, 134.0, 131.7, 129.1, 128.7, 127.7, 127.5, 127.2, 115.9, 37.0, 18.5.

HRMS (ESI+, *m/z*) calculated for $C_{17}H_{17}NO$ $[M + H]^+$: 252.1383; found: 252.1379;.

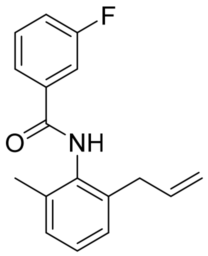

1l: white solid; yield 71%, m.p. = 74-77 °C.

^{1}H NMR (400 MHz, $CDCl_3$) δ 7.75 (s, 1H), 7.66 – 7.57 (m, 2H), 7.45-7.40 (m, 1H), 7.25 – 7.06 (m, 4H), 5.94 (ddt, *J* = 16.5, 10.2, 6.2 Hz, 1H), 5.08 (dd, *J* = 10.1, 1.0 Hz, 1H), 4.95 (dd, *J* = 17.1, 1.4 Hz, 1H), 3.35 (d, *J* = 6.2 Hz, 2H), 2.24 (s, 3H).

^{13}C NMR (101 MHz, $CDCl_3$) δ 164.5, 162.7 (d, *J* = 249.5 Hz), 136.7, 136.5, 136.4, 136.3 (d, *J* = 4.0 Hz), 136.2, 133.7, 130.3 (d, *J* = 7.9 Hz), 129.0, 127.6, 122.6 (d, *J* = 3.0 Hz), 118.7 (d, *J* =21.2 Hz), 115.9, 114.6 (d, *J* = 22.2 Hz), 36.9, 18.4.

^{19}F NMR (471 MHz, $CDCl_3$) δ -111.40.

HRMS (ESI+, *m/z*) calculated for $C_{17}H_{16}FNO$ $[M + H]^+$: 270.1289; found: 270.1292;.

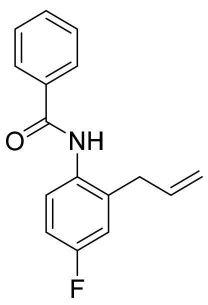

1m: white solid; yield 55%, m.p. = 117-119 °C.

^{1}H NMR (400 MHz, $CDCl_3$) δ 7.93 (s, 1H), 7.87-7.83 (m, 3H), 7.55 (t, *J* = 7.2 Hz, 1H), 7.47 (t, *J* = 7.6 Hz, 2H), 7.01-6.93 (m, 2H), 6.09-5.92 (m, 1H), 5.24 (d, *J* = 10.1 Hz, 1H), 5.10 (d, *J* = 17.2 Hz, 1H), 3.40 (d, *J* = 5.9 Hz, 2H).

^{13}C NMR (101 MHz, $CDCl_3$) δ 165.7, 160.1 (d, *J* = 245.4 Hz), 135.4, 134.5, 133.5 (d, *J* = 7.0 Hz), 131.9, 131.9, 128.8, 127.0, 125.9 (d, *J* = 8.3 Hz), 117.3, 116.8 (d, *J* = 22.2 Hz), 114.0 (d, *J* = 22.2 Hz), 36.7.

^{19}F NMR (471 MHz, $CDCl_3$) δ -116.84.

HRMS (ESI+, *m/z*) calculated for $C_{16}H_{14}FNO$ [M + H]$^+$: 256.1132; found: 256.1127;.

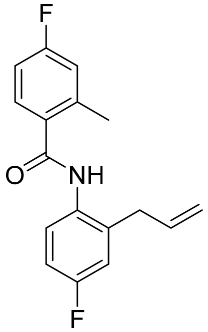

1n: white solid; yield 85%, m.p. = 130-132 °C.

^{1}H NMR (500 MHz, $CDCl_3$) δ 7.93 – 7.82 (m, 1H), 7.47-7.44 (m, 2H), 7.06 – 6.82 (m, 4H), 5.94 (ddt, *J* = 16.5, 10.4, 6.0 Hz, 1H), 5.17 (d, *J* = 10.1 Hz, 1H), 5.01 (d, *J* = 17.2 Hz, 1H), 3.38 (d, *J* = 5.9 Hz, 2H), 2.51 (s, 3H).

^{13}C NMR (126 MHz, $CDCl_3$) δ 167.2, 163.5 (d, *J* = 250.7 Hz), 160.2 (d, *J* = 245.7 Hz), 140.1 (d, *J* = 8.8 Hz), 135.1, 133.2 (d, *J* = 6.3 Hz), 132.1, 131.7 (d, *J* = 2.5 Hz), 128.6 (d, *J* = 9.1 Hz), 125.7 (d, *J* = 7.5 Hz), 118.2 (d, *J* = 21.4 Hz), 117.3, 116.9 (d, *J* = 22.7 Hz), 114.1 (d, *J* = 22.7 Hz), 112.8 (d, *J* = 21.4 Hz), 36.6, 20.1.

^{19}F NMR (471 MHz, $CDCl_3$) δ -110.03, -116.49.

HRMS (ESI+, *m/z*) calculated for $C_{17}H_{15}F_2NO$ [M + H]$^+$: 288.1194; found: 288.1192;.

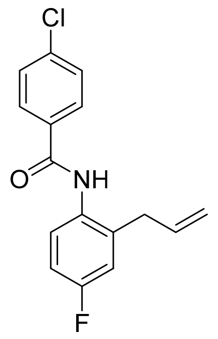

1o: white solid; yield 83%, m.p. = 133-135 °C.

^{1}H NMR (400 MHz, $CDCl_3$) δ 7.91 (s, 1H), 7.82 – 7.72 (m, 3H), 7.44-7.41 (m, 2H), 7.02 – 6.88 (m, 2H), 6.05 – 5.90 (m, 1H), 5.23 (dd, *J* = 10.1, 1.4 Hz, 1H), 5.08 (dd, *J* = 17.2, 1.6 Hz, 1H), 3.39 (d, *J* = 5.9 Hz, 2H).

^{13}C NMR (101 MHz, $CDCl_3$) δ 164.7, 160.2 (d, *J* = 246.44 Hz), 138.2, 135.4, 133.6, 132.9, 131.7 (d, *J* = 2.1 Hz), 129.0, 128.4, 126.0 (d, *J* = 8.3 Hz), 117.3, 116.9 (d, *J* = 8.3 Hz), 114.1 (d, *J* = 22.22 Hz), 36.7.

^{19}F NMR (376 MHz, $CDCl_3$) δ -116.39.

HRMS (ESI+, *m/z*) calculated for $C_{16}H_{13}FClNO$ [M + H]$^+$: 290.0742; found: 290.0742;.

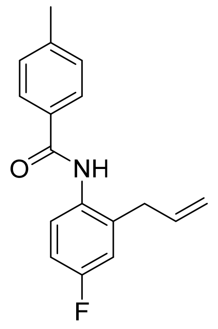

1p: white solid; yield 70%, m.p. = 130-133 °C.

^{1}H NMR (500 MHz, $CDCl_3$) δ 7.95 – 7.88 (m, 1H), 7.82 (s, 1H), 7.74 (d, *J* = 7.5 Hz, 2H), 7.29 (d, *J* = 7.6 Hz, 2H), 7.00 (t, *J* = 8.4 Hz, 1H), 6.95 (d, *J* = 9.1 Hz, 1H), 6.01 (ddt, *J* = 16.5, 10.4, 6.0 Hz, 1H), 5.25 (d, *J* = 10.1 Hz, 1H), 5.11 (d, *J* = 17.2 Hz, 1H), 3.41 (d, *J* = 5.7 Hz, 2H), 2.43 (s, 3H).

^{13}C NMR (126 MHz, $CDCl_3$) δ 165.6, 160.0 (d, *J* = 244.4 Hz), 142.4, 135.4, 133.2 (d, *J* = 7.4 Hz), 132.0 (d, *J* = 2.6 Hz), 131.6, 129.4, 127.0, 125.8 (d, *J* = 8.1 Hz), 117.2, 116.7 (d, *J* = 22.7 Hz), 113.9 (d, *J* = 22.7 Hz), 36.7, 21.4.

^{19}F NMR (471 MHz, $CDCl_3$) δ -117.05.

HRMS (ESI+, *m/z*) calculated for $C_{17}H_{16}FNO$ [M + H]$^+$: 270.1289; found: 270.1288;.

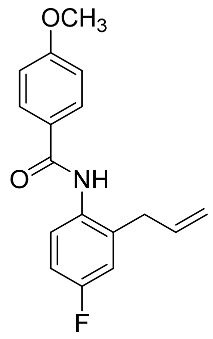

1q: white solid; yield 91%, m.p. = 94-95 °C.

^{1}H NMR (500 MHz, $CDCl_3$) δ 7.89 – 7.77 (m, 4H), 7.04 – 6.86 (m, 4H), 6.00 (ddt, *J* = 16.4, 10.2, 6.0 Hz, 1H), 5.24 (dd, *J* = 10.1, 1.2 Hz, 1H), 5.10 (dd, *J* = 17.2, 1.4 Hz, 1H), 3.87 (s, 3H), 3.40 (d, *J* = 6.0 Hz, 2H).

^{13}C NMR (126 MHz, $CDCl_3$) δ 165.2, 162.5, 160.0 (d, *J* = 244.4 Hz), 135.5, 133.2 (d, *J* = 7.5 Hz), 132.1 (d, *J* = 2.8 Hz), 128.9, 126.7, 125.9 (d, *J* = 8.3 Hz), 117.2, 116.7 (d, *J* = 22.7 Hz), 114.0 (d, *J* = 22.7 Hz), 114.0, 55.4, 36.7.

^{19}F NMR (471 MHz, $CDCl_3$) δ -117.14.

HRMS (ESI+, *m/z*) calculated for $C_{17}H_{16}FNO_2$ $[M + H]^+$: 286.1238; found: 286.1238;.

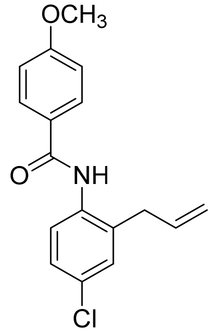

1r: white solid; yield 45%, m.p. = 133-135 °C.

^{1}H NMR (400 MHz, $CDCl_3$) δ 7.99 (d, *J* = 8.7 Hz, 1H), 7.84 (s, 1H), 7.79 (d, *J* = 8.8 Hz, 2H), 7.29 – 7.24 (m, 1H), 7.20 (d, *J* = 2.4 Hz, 1H), 6.96 (d, *J* = 8.8 Hz, 2H), 6.01 (ddt, *J* = 16.2, 10.3, 5.9 Hz, 1H), 5.27 (dd, *J* = 10.1, 1.4 Hz, 1H), 5.13 (dd, *J* = 17.2, 1.5 Hz, 1H), 3.87 (s, 3H), 3.41 (d, *J* = 5.9 Hz, 2H).

^{13}C NMR (101 MHz, $CDCl_3$) δ 164.9, 162.6, 135.4, 135.0, 131.5, 130.0, 130.0, 128.8, 127.5, 126.8, 124.6, 117.5, 114.0, 55.5, 36.7.

HRMS (ESI+, *m/z*) calculated for $C_{17}H_{16}ClNO_2$ $[M + H]^+$: 302.0942; found: 302.0938;.

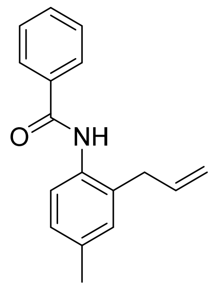

1s: white solid; yield 59%, m.p. = 162-164 °C.

^{1}H NMR (500 MHz, $CDCl_3$) δ 7.89-7.84 (m, 4H), 7.55 (t, *J* = 7.3 Hz, 1H), 7.48 (t, *J* = 7.5 Hz, 2H), 7.13 (d, *J* = 8.1 Hz, 1H), 7.04 (s, 1H), 6.03 (ddt, *J* = 16.2, 10.3, 5.9 Hz, 1H), 5.22 (d, *J* = 10.1 Hz, 1H), 5.11 (d, *J* = 17.2 Hz, 1H), 3.42 (d, *J* = 5.8 Hz, 2H), 2.34 (s, 3H).

^{13}C NMR (126 MHz, $CDCl_3$) δ 165.5, 136.4, 135.1, 135.0, 133.6, 131.7, 130.9, 130.1, 128.7, 128.1, 127.0, 123.7, 116.6, 37.0, 20.9.

HRMS (ESI+, *m/z*) calculated for $C_{17}H_{17}NO$ $[M + H]^+$: 252.1383; found: 252.1380;.

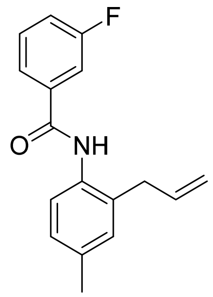

1t: white solid; yield 82%, m.p. = 107-109 °C.

^{1}H NMR (500 MHz, $CDCl_3$) δ 7.98 (s, 1H), 7.79 (d, *J* = 7.5 Hz, 1H), 7.59-7.55 (m, 2H), 7.45-7.42 (m, 1H), 7.24-7.21 (m, 1H), 7.10 (d, *J* = 8.1 Hz, 1H), 7.03 (s, 1H), 6.01 (ddt, *J* = 16.2, 10.3, 5.9 Hz, 1H), 5.22 (d, *J* = 10.1 Hz, 1H), 5.10 (d, *J* = 17.2 Hz, 1H), 3.40 (d, *J* = 5.9 Hz, 2H), 2.34 (s, 3H).

^{13}C NMR (126 MHz, $CDCl_3$) δ 164.2, 162.8(d, *J* = 248.2 Hz), 137.1 (d, *J* = 6.8 Hz), 136.4, 135.4, 133.2, 130.9, 130.5, 130.3 (d, *J* = 7.9 Hz), 128.0, 123.8, 122.3 (d, *J* = 2.6 Hz), 118.6 (d, *J* = 21.4 Hz), 116.6, 114.5 (d, *J* = 22.7 Hz), 36.9, 20.9.

^{19}F NMR (471 MHz, $CDCl_3$) δ -111.43.

HRMS (ESI+, *m/z*) calculated for $C_{17}H_{16}FNO$ $[M + H]^+$: 270.1289; found: 270.1288;.

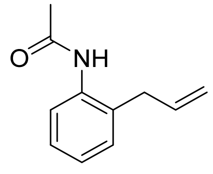

1v: white solid; yield 82%, m.p. = 95-97 °C.

^{1}H NMR (400 MHz, $CDCl_3$) δ 7.84 (d, *J* = 7.9 Hz, 1H), 7.30 – 7.10 (m, 4H), 5.98 (ddd, *J* = 16.3, 11.5, 6.0 Hz, 1H), 5.18 (d, *J* = 10.0 Hz, 1H), 5.10 (d, *J* = 17.0 Hz, 1H), 3.39 (d, *J* = 6.0 Hz, 2H), 2.15 (s, 3H).

^{13}C NMR (101 MHz, $CDCl_3$) δ 168.2, 136.3, 136.0, 130.1, 130.0, 127.4, 125.3, 123.8, 116.5, 36.9, 24.2.

HRMS (ESI+, *m/z*) calculated for $C_{11}H_{13}NO$ $[M + H]^+$: 176.1070; found: 176.1082;

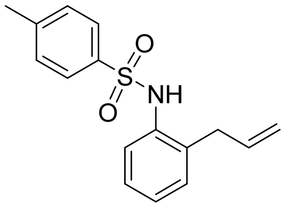

1w: white solid; yield 82%, m.p. = 67-69 °C.

^{1}H NMR (400 MHz, $CDCl_3$) δ 7.60 (d, *J* = 8.2 Hz, 2H), 7.40 (d, *J* = 7.9 Hz, 1H), 7.23-7.18 (m, 3H), 7.14-7.03 (m, 2H), 6.53 (s, 1H), 5.78 (ddt, *J* = 16.4, 10.3, 6.0 Hz, 1H), 5.11 (d, *J* = 10.1 Hz, 1H), 4.94 (dd, *J* = 17.2, 1.2 Hz, 1H), 3.02 (d, *J* = 5.9 Hz, 2H), 2.39 (s, 3H).

^{13}C NMR (101 MHz, $CDCl_3$) δ 143.8, 136.8, 135.5, 135.0, 131.9, 130.4, 129.6, 127.7, 127.1, 126.2, 124.4, 117.0, 36.2, 21.5.

HRMS (ESI+, *m/z*) calculated for $C_{16}H_{17}NO_2S$ $[M + H]^+$: 288.1053; found: 288.1026;

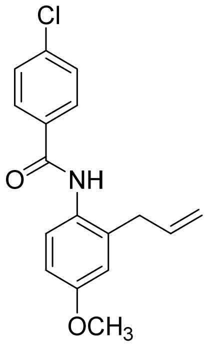

4: white solid; yield 89%, m.p. = 135-137 °C.

^{1}H NMR (400 MHz, $CDCl_3$) δ 7.99 (s, 1H), 7.73 (d, *J* = 7.8 Hz, 2H), 7.60 (d, *J* = 8.4 Hz, 1H), 7.37 (d, *J* = 7.6 Hz, 2H), 6.78-6.75 (m, 2H), 5.95(ddt, *J* = 16.2, 10.2, 6.0 Hz, 1H), 5.15 (d, *J* = 10.1 Hz, 1H), 5.05 (d, *J* = 17.2 Hz, 1H), 3.79 (s, 3H), 3.34 (d, *J* = 5.7 Hz, 2H).

^{13}C NMR (101 MHz, $CDCl_3$) δ 164.7, 157.5, 137.7, 136.0, 133.7, 133.0, 128.7, 128.5, 128.4, 126.1, 116.6, 115.6, 112.1, 55.32, 36.8.

HRMS (ESI+, *m/z*) calculated for $C_{17}H_{16}ClNO_2$ $[M + H]^+$: 302.0942; found: 302.0933;

4. Typical experimental procedure

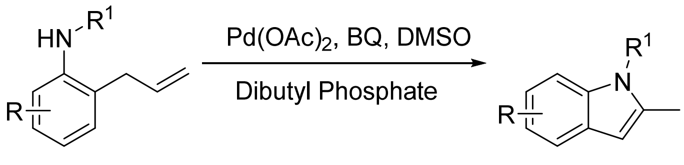

To a solution of **1a-w, 4** (0.2 mmol) in DMSO (2 mL) were added the $Pd(OAc)_2$ (0.02 mmol), BQ (0.4 mmol) and dibutyl phosphate (0.3 mmol), and the mixture was heated to 60-70 ºC and stirred at this temperature for 24-48 hours. The reaction mixture was then extracted with EtOAc (3x15 mL), washed with brine (30 mL), dried over Na_2SO_4 and concentrated in vacuo. The crude product was purified by column chromatography (eluting with petroleum ether/ethyl acetate 0-2%) to afford **2a-w, 5**.

5. Characterization of the Products

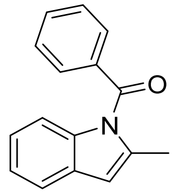

2a: pale yellow oil; yield 81%.

^{1}H NMR (500 MHz, $CDCl_3$) δ 7.73 (d, J = 7.8 Hz, 2H), 7.63 (t, J = 7.4 Hz, 1H), 7.52-7.47 (m, 3H), 7.16-7.13 (m, 1H), 7.06-6.98 (m, 2H), 6.44 (s, 1H), 2.42 (s, 3H).

^{13}C NMR (126 MHz, $CDCl_3$) δ 169.8, 137.9, 137.1, 135.5, 132.8, 129.7, 129.5, 128.7, 122.6, 122.5 119.8, 114.3, 108.5, 15.7.

HRMS (ESI+, *m/z*) calculated for $C_{16}H_{13}NO$ $[M + H]^+$: 236.1070; found: 236.1035;.

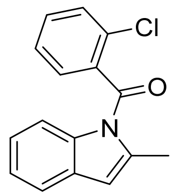

2b: pale yellow oil; yield 62%.

^{1}H NMR (400 MHz, $CDCl_3$) δ 7.52 – 7.47 (m, 3H), 7.46 – 7.39 (m, 2H), 7.35 (d, J = 8.3 Hz, 1H), 7.20 (t, J = 7.3 Hz, 1H), 7.10 (t, J = 7.8 Hz, 1H), 6.41 (s, 1H), 2.26 (s, 3H).

^{13}C NMR (101 MHz, $CDCl_3$) δ166.9, 137.2, 136.8, 136.4, 132.0, 131.7, 130.3, 129.9, 129.2, 127.3, 123.7,

123.6, 119.8, 114.8, 110.3, 16.1.

HRMS (ESI+, *m/z*) calculated for $C_{16}H_{12}C1NO$ [M + H]$^+$: 270.0680; found: 270.0677;.

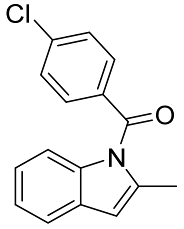

2c: pale yellow oil; yield 73%.

^{1}H NMR (400 MHz, $CDCl_3$) δ 7.67 (d, *J* = 8.5 Hz, 2H), 7.49-7.47 (m, 3H), 7.15 (t, *J* = 7.4 Hz, 1H), 7.08 – 6.95 (m, 2H), 6.44 (s, 1H), 2.42 (s, 3H).

^{13}C NMR (126 MHz, $CDCl_3$) δ 168.6, 139.3, 137.8, 137.0, 133.8, 131.2, 129.5, 129.1, 122.8, 122.7, 119.9, 114.1, 108.8, 15.7.

HRMS (ESI+, *m/z*) calculated for $C_{16}H_{12}C1NO$ [M + H]$^+$: 270.0680; found: 270.0678;.

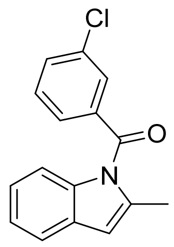

2d: yellow oil; yield 65%.

^{1}H NMR (500 MHz, $CDCl_3$) δ 7.73 (t, *J* = 1.8 Hz, 1H), 7.64 – 7.56 (m, 2H), 7.48 (d, *J* = 7.7 Hz, 1H), 7.43 (t, *J* = 7.9 Hz, 1H), 7.18-7.16 (m, 1H), 7.08 – 7.00 (m, 2H), 6.45 (d, *J* = 0.5 Hz, 1H), 2.41 (d, *J* = 1.1 Hz, 3H).

^{13}C NMR (126 MHz, $CDCl_3$) δ 168.3, 137.6, 137.2, 134.9, 134.9, 132.7, 130.0, 129.5, 129.5, 127.7, 122.9, 122.9, 120.0, 114.2, 109.1, 15.8.

HRMS (ESI+, *m/z*) calculated for $C_{16}H_{12}C1NO$ [M + H]$^+$: 270.0680; found: 270.0678;.

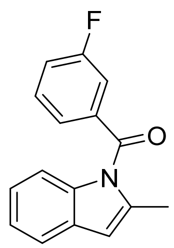

2e: pale yellow oil; yield 73%.

^{1}H NMR (500 MHz, $CDCl_3$) δ 7.51 – 7.41 (m, 4H), 7.35-7.31 (m, 1H), 7.16 (t, *J* = 7.2 Hz, 1H), 7.10 – 6.97 (m, 2H), 6.44 (s, 1H), 2.41 (s, 3H).

^{13}C NMR (126 MHz, $CDCl_3$) δ 168.4, 162.6 (d, *J* = 249.5 Hz), 137.7, 137.5 (d, *J* = 7.6 Hz), 137.0, 130.5 (d, *J* = 7.8 Hz), 129.6, 125.4, 125.4, 122.8 (d, *J* = 3.6 Hz), 119.9, 119.8 (d, *J* = 21.4 Hz), 116.5 (d, *J* = 22.7 Hz), 114.2, 109.0, 15.8.

^{19}F NMR (376 MHz, $CDCl_3$) δ -111.11.

HRMS (ESI+, *m/z*) calculated for $C_{16}H_{12}FNO$ $[M + H]^+$: 254.0976; found: 254.0976;.

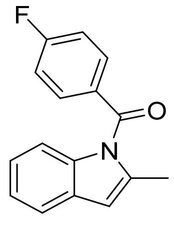

2f: pale yellow oil; yield 79%.

^{1}H NMR (500 MHz, $CDCl_3$) δ 7.76 (dd, *J* = 8.5, 5.5 Hz, 2H), 7.48 (d, *J* = 7.8 Hz, 1H), 7.21 – 7.12 (m, 3H), 7.04 (t, *J* = 7.7 Hz, 1H), 6.97 (d, *J* = 8.3 Hz, 1H), 6.44 (s, 1H), 2.44 (s, 3H).

^{13}C NMR (126 MHz, $CDCl_3$) δ 168.6, 165.6 (d, *J* = 255.78 Hz), 137.9, 137.0, 132.4 (d, *J* = 9.2 Hz), 131.5 (d, *J* = 3.2 Hz), 129.5, 122.7, 122.6, 119.9, 116.1 (d, *J* = 22.7 Hz), 114.1, 108.6, 15.6.

^{19}F NMR (471 MHz, $CDCl_3$) δ -121.16.

HRMS (ESI+, *m/z*) calculated for $C_{16}H_{12}FNO$ $[M + H]^+$: 254.0976; found: 254.0975;.

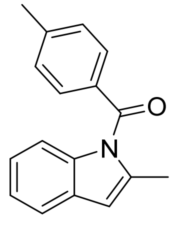

2g: pale yellow oil; yield 81%.

^{1}H NMR (500 MHz, $CDCl_3$) δ 7.64 (d, *J* = 7.8 Hz, 2H), 7.48 (d, *J* = 7.7 Hz, 1H), 7.30 (d, *J* = 7.8 Hz, 2H), 7.17 – 7.11 (m, 1H), 7.04-7.03 (m, 2H), 6.43 (s, 1H), 2.47 (s, 3H), 2.44 (s, 3H).

^{13}C NMR (126 MHz, $CDCl_3$) δ 169.8, 143.8, 137.9, 137.1, 132.6, 130.0, 129.4, 129.4, 122.4, 122.3, 119.7, 114.2, 108.1, 21.7, 15.6.

HRMS (ESI+, *m/z*) calculated for $C_{17}H_{15}NO$ $[M + Na]^+$: 272.1046; found: 272.1047;.

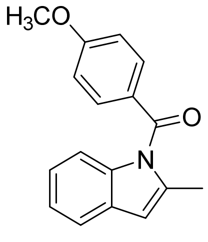

2h: pale yellow oil; yield 90%.

^{1}H NMR (500 MHz, $CDCl_3$) δ 7.73 (d, *J* = 8.4 Hz, 2H), 7.48 (d, *J* = 7.8 Hz, 1H), 7.14 (dt, *J* = 7.8, 4.0 Hz, 1H), 7.03 (d, *J* = 4.0 Hz, 2H), 6.97 (d, *J* = 8.4 Hz, 2H), 6.43 (s, 1H), 3.90 (s, 3H), 2.45 (s, 3H).

^{13}C NMR (126 MHz, $CDCl_3$) δ 169.1, 163.6, 138.0, 137.2, 132.4, 129.3, 127.4, 122.3, 122.2, 119.7, 114.0, 107.8, 55.5, 15.4.

HRMS (ESI+, *m/z*) calculated for $C_{17}H_{15}NO_2$ $[M + H]^+$: 266.1176; found: 266.1173;.

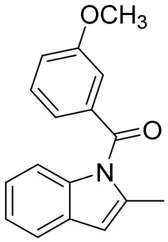

2i: pale yellow oil; yield 84%.

^{1}H NMR (500 MHz, $CDCl_3$) δ 7.48 (d, *J* = 7.7 Hz, 1H), 7.39 (t, *J* = 7.9 Hz, 1H), 7.31 – 7.27 (m, 2H), 7.19-7.14 (m, 2H), 7.10 – 7.02 (m, 2H), 6.44 (s, 1H), 3.85 (s, 3H), 2.43 (s, 3H).

^{13}C NMR (126 MHz, $CDCl_3$) δ 169.6, 159.8, 137.9, 137.1, 136.8, 129.7, 129.5, 122.7, 122.6, 122.1, 119.7, 119.2, 114.3, 114.0, 108.6, 55.5, 15.7.

HRMS (ESI+, *m/z*) calculated for $C_{17}H_{15}NO_2$ $[M + H]^+$: 266.1176; found: 266.1171;.

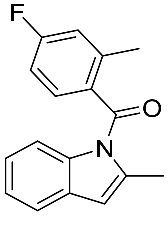

2j: pale yellow oil; yield 76%.

^{1}H NMR (400 MHz, $CDCl_3$) δ 7.45 (d, *J* = 7.7 Hz, 1H), 7.39 (dd, *J* = 8.4, 5.8 Hz, 1H), 7.19 – 7.13 (m, 1H), 7.13 – 6.94 (m, 4H), 6.41 (s, 1H), 2.32 (s, 3H), 2.31 (s, 3H).

^{13}C NMR (126 MHz, $CDCl_3$) δ 169.1, 165.5 (d, *J* = 255.78 Hz), 140.0 (d, *J* = 8.6 Hz), 137.5, 136.7, 132.4 (d, *J* = 3.2 Hz), 130.7 (d, *J* = 9.1 Hz), 129.7, 123.3, 123.1, 119.8, 118.1 (d, *J* = 21.4 Hz), 114.3, 113.3 (d,

J = 21.4 Hz), 109.6, 19.4, 16.1.

^{19}F NMR (471 MHz, $CDCl_3$) δ -108.27.

HRMS (ESI+, *m/z*) calculated for $C_{17}H_{14}FNO$ [M + H]$^+$: 268.1132; found: 268.1130;.

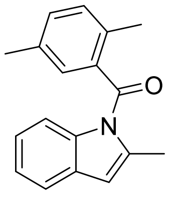

2u: white solid; yield 73%.

^{1}H NMR (500 MHz, $CDCl_3$) δ 7.44 (d, *J* = 7.7 Hz, 1H), 7.24 (s, 1H), 7.22 – 7.12 (m, 4H), 7.05 (t, *J* = 7.8 Hz, 1H), 6.39 (s, 1H), 2.33 (s, 3H), 2.30 (s, 3H), 2.22 (s, 3H).

^{13}C NMR (126 MHz, $CDCl_3$) δ 170.3, 137.6, 136.8, 136.3, 135.9, 133.1, 131.8, 131.0, 129.7, 128.5, 123.2, 123.0, 119.6, 114.5, 109.4, 20.8, 18.7, 16.2.

HRMS (ESI+, *m/z*) calculated for $C_{18}H_{17}NO$ [M + H]$^+$: 264.1383; found: 264.1384;.

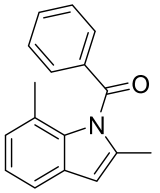

2k: pale yellow oil; yield 68%.

^{1}H NMR (500 MHz, $CDCl_3$) δ 7.74 (d, *J* = 7.7 Hz, 2H), 7.62 (t, *J* = 7.4 Hz, 1H), 7.46 (t, *J* = 7.7 Hz, 2H), 7.40 (d, *J* = 7.8 Hz, 1H), 7.13 (t, *J* = 7.5 Hz, 1H), 6.95 (d, *J* = 7.3 Hz, 1H), 6.39 (s, 1H), 2.23 (s, 3H), 2.07 (s, 3H).

^{13}C NMR (126 MHz, $CDCl_3$) δ 171.0, 136.9, 136.9, 135.6, 133.8, 130.3, 129.9, 128.9, 125.5, 123.0, 122.4, 117.6, 106.1, 20.3, 15.0.

HRMS (ESI+, *m/z*) calculated for $C_{17}H_{15}NO$ [M + H]$^+$: 250.1226; found: 250.1224;.

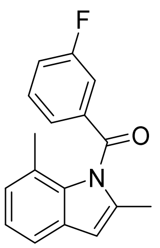

2l: pale yellow oil; yield 70%.

^{1}H NMR (400 MHz, $CDCl_3$) δ 7.51 – 7.36 (m, 4H), 7.32 (t, *J* = 7.9 Hz, 1H), 7.13 (t, *J* = 7.5 Hz, 1H),

6.96 (d, *J* = 7.2 Hz, 1H), 6.39 (s, 1H), 2.22 (s, 3H), 2.06 (s, 3H).

^{13}C NMR (126 MHz, $CDCl_3$) δ 169.7, 162.7 (d, *J* = 249.5 Hz), 137.8 (d, *J* = 6.8 Hz), 136.9, 136.8, 130.5 (d, *J* = 7.8 Hz), 130.0, 126.1 (d, *J* = 3.1 Hz), 125.8, 123.0, 122.7, 120.9 (d, *J* = 21.4 Hz), 117.8, 117.0 (d, *J* =22.7 Hz), 106.7, 20.4, 15.1.

^{19}F NMR (471 MHz, $CDCl_3$) δ -111.03.

HRMS (ESI+, *m/z*) calculated for $C_{17}H_{14}FNO$ $[M + H]^+$: 268.1132; found: 268.1130;.

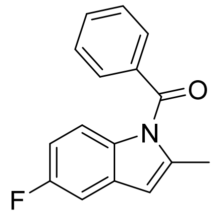

2m: pale yellow oil; yield 69%.

^{1}H NMR (400 MHz, $CDCl_3$) δ 7.70 (d, *J* = 7.5 Hz, 2H), 7.64 (t, *J* = 7.4 Hz, 1H), 7.52-7.48 (m, 2H), 7.12 (dd, *J* = 8.8, 2.2 Hz, 1H), 7.01-6.98 (m, 1H), 6.76 (td, *J* = 9.1, 2.3 Hz, 1H), 6.38 (s, 1H), 2.37 (s, 3H).

^{13}C NMR (101 MHz, $CDCl_3$) δ 169.6, 159.2 (d, *J* =240.4 Hz), 139.6, 135.3, 133.5, 132.9, 130.4, 129.6, 128.8, 115.1 (d, *J* = 9.1 Hz), 110.4 (d, *J* = 25.2 Hz), 108.3 (d, *J* = 3.8 Hz), 105.2 (d, *J* = 24.2 Hz), 15.9.

^{19}F NMR (471 MHz, $CDCl_3$) δ -121.16.

HRMS (ESI+, *m/z*) calculated for $C_{16}H_{12}FNO$ $[M + H]^+$: 254.0976; found: 254.0973;.

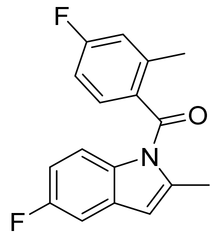

2n: pale yellow oil; yield 71%.

^{1}H NMR (500 MHz, $CDCl_3$) δ 7.37 (dd, *J* = 8.4, 5.7 Hz, 1H), 7.15 (dd, *J* = 9.1, 4.5 Hz, 1H), 7.10 (dd, *J* = 8.7, 2.6 Hz, 1H), 7.06 – 6.96 (m, 2H), 6.80 (td, *J* = 9.1, 2.6 Hz, 1H), 6.36 (s, 1H), 2.30 (s, 3H), 2.25 (s, 3H).

^{13}C NMR (126 MHz, $CDCl_3$) δ 168.8, 164.1 (d, *J* = 253.3 Hz), 159.5 (d, *J* = 239.4 Hz), 140.0 (d, *J* = 8.6 Hz), 139.0, 133.1, 132.2 (d, *J* = 3.2 Hz), 130.7 (d, *J* = 9.1 Hz), 118.3, 118.1, 115.4 (d, *J* = 9.1 Hz), 113. 4 (d, *J* = 21.4 Hz), 110.9 (d, *J* = 25.2 Hz), 109.37 (d, *J* = 3.7 Hz), 105.4(d, *J* = 23.9 Hz), 19.4, 16.2.

^{19}F NMR (471 MHz, $CDCl_3$) δ -107.90, -120.08.

HRMS (ESI+, *m/z*) calculated for $C_{17}H_{13}F_2NO$ $[M + H]^+$: 286.1038; found: 286.1036;.

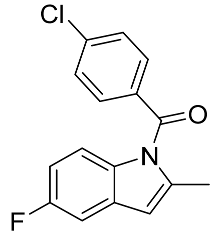

2o: pale yellow oil; yield 67%.

^1H NMR (500 MHz, $CDCl_3$) δ 7.71 – 7.60 (m, 2H), 7.57 – 7.41 (m, 2H), 7.12 (dd, *J* = 8.8, 2.6 Hz, 1H), 6.99 (dd, *J* = 9.0, 4.4 Hz, 1H), 6.78 (td, *J* = 9.1, 2.6 Hz, 1H), 6.39 (s, 1H), 2.38 (s, 3H).

^{13}C NMR (126 MHz, $CDCl_3$) δ 168.4, 159.3 (d, *J* = 239.4 Hz), 139.5, 139.4, 133.5, 133.3, 131.1, 130.4 (d, *J* = 10.0 Hz), 129.2, 115.0 (d, *J* = 9.2 Hz), 110.4 (d, *J* = 25.2 Hz), 108.6 (d, *J* = 3.7 Hz), 105.4 (d, *J* = 23.9 Hz), 15.9.

^{19}F NMR (376 MHz, $CDCl_3$) δ -120.81.

HRMS (ESI+, *m/z*) calculated for $C_{16}H_{11}FClNO$ $[M + H]^+$: 288.0586; found: 288.0583;.

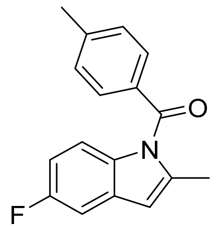

2p: pale yellow oil; yield 70%.

^1H NMR (400 MHz, $CDCl_3$) δ 7.61 (d, *J* = 7.8 Hz, 2H), 7.29 (d, *J* = 7.8 Hz, 2H), 7.12 (d, *J* = 8.9 Hz, 1H), 7.02-6.98 (m, 1H), 6.76 (t, *J* = 9.1 Hz, 1H), 6.37 (s, 1H), 2.47 (s, 3H), 2.39 (s, 3H).

^{13}C NMR (101 MHz, $CDCl_3$) δ 169.5, 159.0 (d, *J* = 239.4 Hz), 144.0, 139.6, 133.5, 132.3, 130.3 (d, *J* = 10.1 Hz), 129.9, 129.4, 114.9 (d, *J* = 9.2 Hz), 110.1 (d, *J* = 25.2 Hz), 107.9 (d, *J* = 3.8 Hz), 105.1 (d, *J* = 23.2 Hz), 21.7, 15.7.

^{19}F NMR (376 MHz, $CDCl_3$) δ -121.47.

HRMS (ESI+, *m/z*) calculated for $C_{17}H_{14}FNO$ $[M + H]^+$: 268.1132; found: 268.1130;.

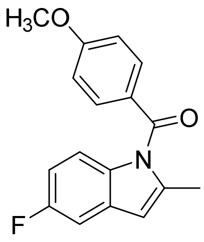

2q: pale yellow oil; yield 78%.

^{1}H NMR (500 MHz, $CDCl_3$) δ 7.70 (d, J = 8.5 Hz, 2H), 7.12 (dd, J = 8.9, 2.5 Hz, 1H), 6.99-6.96 (m, 3H), 6.75 (td, J = 9.1, 2.4 Hz, 1H), 6.37 (s, 1H), 3.90 (s, 3H), 2.41 (s, 3H).

^{13}C NMR (101 MHz, $CDCl_3$) δ 168.9, 163.7, 159.0 (d, J = 239.4 Hz), 139.7, 133.6, 132.4, 130.2 (d, J = 10.0 Hz), 127.1, 113.7 (d, J = 9.1 Hz), 114.0, 110.1 (d, J = 25.2 Hz), 107.6 (d, J = 3.9 Hz), 105.1 (d, J = 23.2 Hz), 55.6, 15.5.

^{19}F NMR (376 MHz, $CDCl_3$) δ -121.77.

HRMS (ESI+, *m/z*) calculated for $C_{17}H_{14}FNO_2$ $[M + H]^+$: 284.1081; found: 284.1079;.

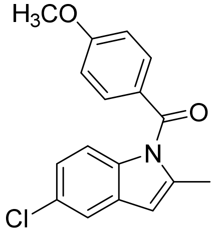

2r: white solid; yield 75%.

^{1}H NMR (500 MHz, $CDCl_3$) δ 7.77 – 7.62 (m, 2H), 7.44 (d, J = 2.0 Hz, 1H), 7.02 – 6.87 (m, 4H), 6.36 (s, 1H), 3.90 (s, 3H), 2.43 (s, 3H).

^{13}C NMR (126 MHz, $CDCl_3$) δ 168.8, 163.8, 139.4, 135.5, 132.4, 130.5, 127.7, 126.9, 122.4, 119.3, 114.9, 114.1, 107.0, 55.6, 15.4.

HRMS (ESI+, *m/z*) calculated for $C_{17}H_{14}C1NO_2$ $[M + H]^+$: 300.0786; found: 300.0786;.

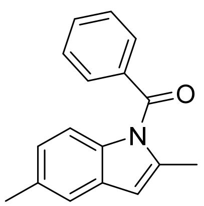

2s: pale yellow oil; yield 74%.

^{1}H NMR (500 MHz, $CDCl_3$) δ 7.71 (d, J = 7.6 Hz, 2H), 7.62 (t, J = 7.4 Hz, 1H), 7.49 (t, J = 7.5 Hz, 2H),

7.26 (s, 1H), 6.87-6.82 (m, 2H), 6.36 (s, 1H), 2.41 (s, 3H), 2.39 (s, 3H).

^{13}C NMR (126 MHz, $CDCl_3$) δ 169.8, 138.0, 135.6, 135.3, 132.7, 132.1, 129.8, 129.6, 128.7, 123.9, 119.7, 114.0, 108.4, 21.2, 15.8.

HRMS (ESI+, *m/z*) calculated for $C_{17}H_{15}NO$ $[M + H]^+$: 250.1226; found: 250.1226;.

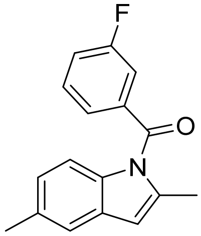

2t: pale yellow oil; yield 73%.

^{1}H NMR (400 MHz, $CDCl_3$) δ 7.42-7.37 (m, 3H), 7.30 – 7.22 (m, 1H), 7.21 (s, 1H), 6.85-6.79 (m, 2H), 6.31 (s, 1H), 2.35 (s, 3H), 2.34 (s, 3H).

^{13}C NMR (101 MHz, $CDCl_3$) δ 168.3, 162.5 (d, J = 250.5 Hz), 137.8, 137.7 (d, J = 7.8 Hz), 135.2, 132.4, 130.4 (d, J = 7.8 Hz), 129.8, 125.2 (d, J = 3.1 Hz), 124.1, 119.8, 119.6 (d, J = 21.2 Hz), 116.4 (d, J = 23.2 Hz), 113.9, 108.9, 21.1, 15.8.

^{19}F NMR (471 MHz, $CDCl_3$) δ -111.15.

HRMS (ESI+, *m/z*) calculated for $C_{17}H_{14}FNO$ $[M + H]^+$: 268.1132; found: 268.1133;.

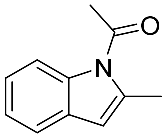

2v: pale yellow oil; yield 81%.

^{1}H NMR (500 MHz, $CDCl_3$) δ 7.91 (d, J = 8.0 Hz, 1H), 7.37 (d, J = 7.3 Hz, 1H), 7.21 – 7.11 (m, 2H), 6.29 (s, 1H), 2.64 (s, 3H), 2.55 (s, 3H).

^{13}C NMR (101 MHz, $CDCl_3$) δ 170.3, 137.3, 136.5, 129.8, 123.5, 123.1, 119.8, 115.2, 109.7, 27.4, 17.6.

HRMS (ESI+, *m/z*) calculated for $C_{11}H_{11}NO$ $[M + H]^+$: 174.0913; found: 174.0872;

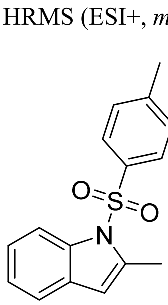

2w: pale yellow oil; yield 75%.

^{1}H NMR (400 MHz, $CDCl_3$) δ 8.18 (d, *J* = 8.3 Hz, 1H), 7.69 (d, *J* = 8.2 Hz, 2H), 7.42 (d, *J* = 7.6 Hz, 1H), 7.29 (d, *J* = 6.2 Hz, 1H), 7.24 – 7.20 (m, 3H), 6.36 (s, 1H), 2.63 (s, 3H), 2.36 (s, 3H).

^{13}C NMR (101 MHz, $CDCl_3$) δ 144.6, 137.3, 137.0, 136.3, 129.8, 129.6, 126.3, 123.6, 123.3, 119.9, 114.4, 109.5, 21.5, 15.7.

HRMS (ESI+, *m/z*) calculated for $C_{16}H_{15}NO_2S$ $[M + H]^+$: 286.0896; found: 286.0889;

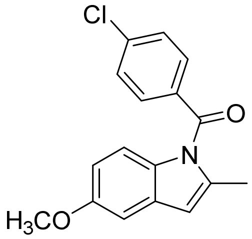

5: pale yellow oil; yield 71%.

^{1}H NMR (400 MHz, $CDCl_3$) δ 7.65 (d, *J* = 8.4 Hz, 2H), 7.47 (d, *J* = 8.4 Hz, 2H), 6.94 (d, *J* = 2.2 Hz, 1H), 6.90 (d, *J* = 9.0 Hz, 1H), 6.66 (dd, *J* = 9.0, 2.4 Hz, 1H), 6.36 (s, 1H), 3.82 (s, 3H), 2.40 (s, 3H).

^{13}C NMR (101 MHz, $CDCl_3$) δ 168.4, 155.9, 139.1, 138.6, 133.9, 131.6, 131.0, 130.5, 129.1, 115.0, 111.2, 108.9, 102.7, 55.6, 15.9.

HRMS (ESI+, *m/z*) calculated for $C_{17}H_{14}C1NO_2$ $[M + H]^+$: 300.0786; found: 300.0778;.

6. NMR Spectra

S2a

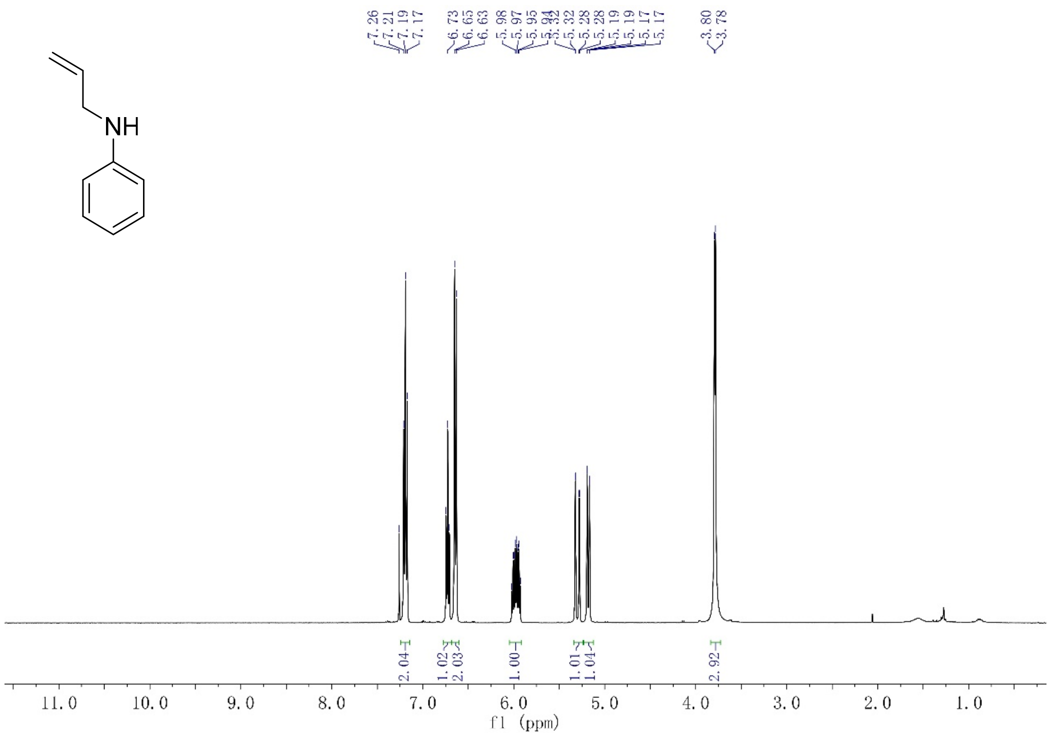

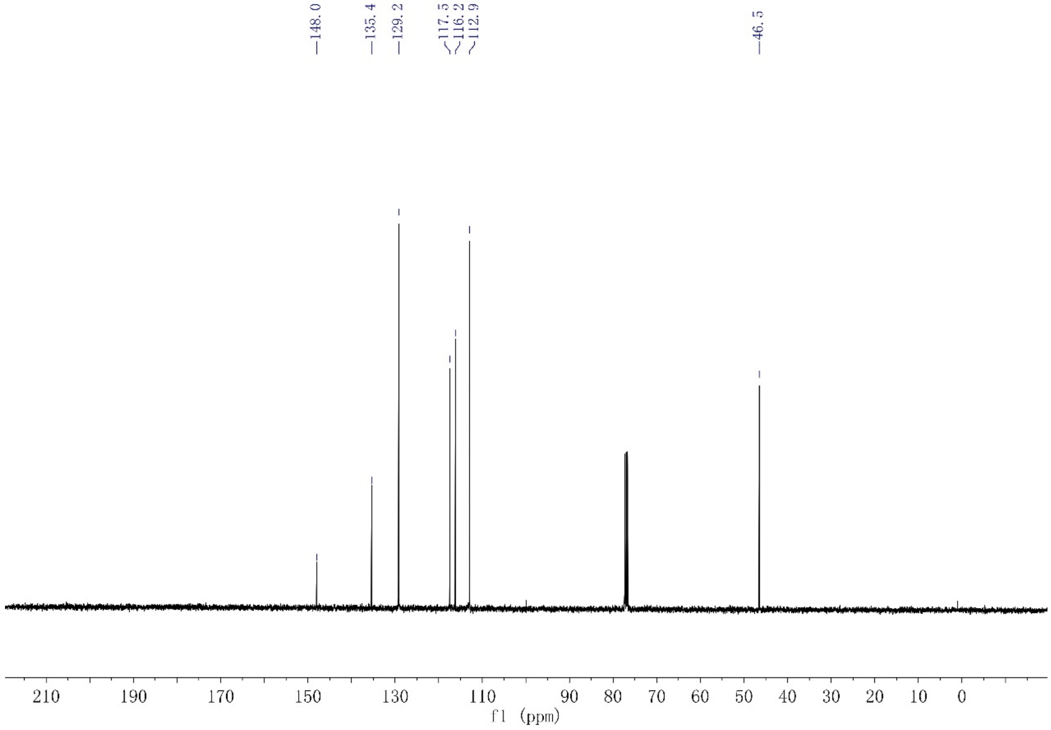

S2b

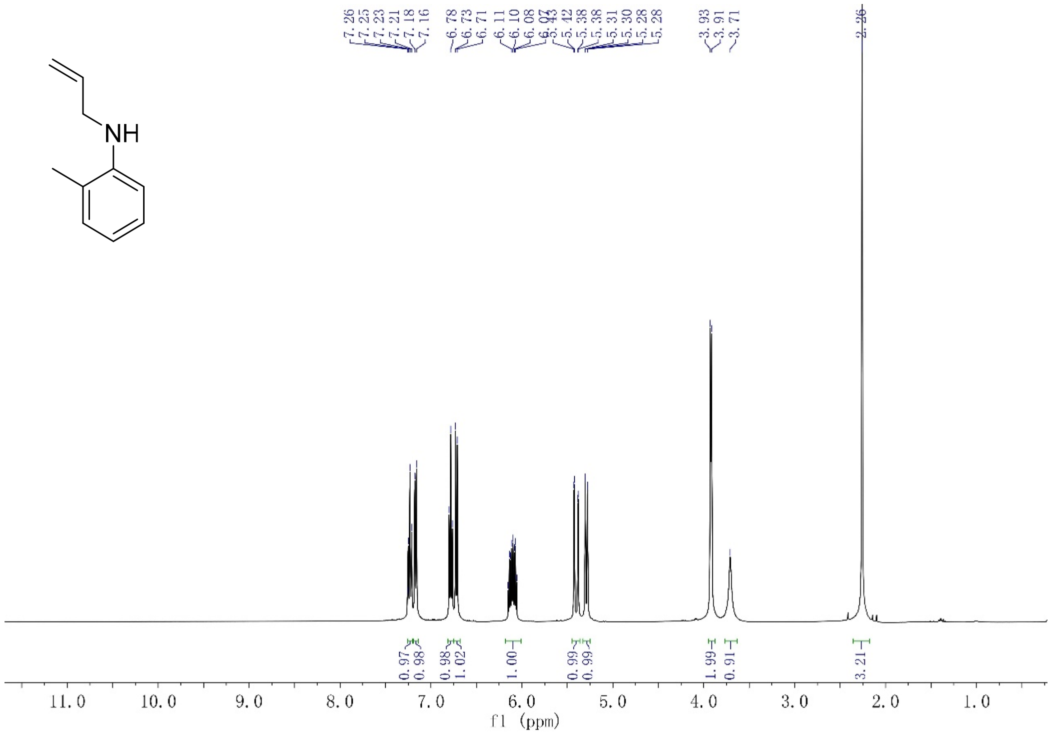

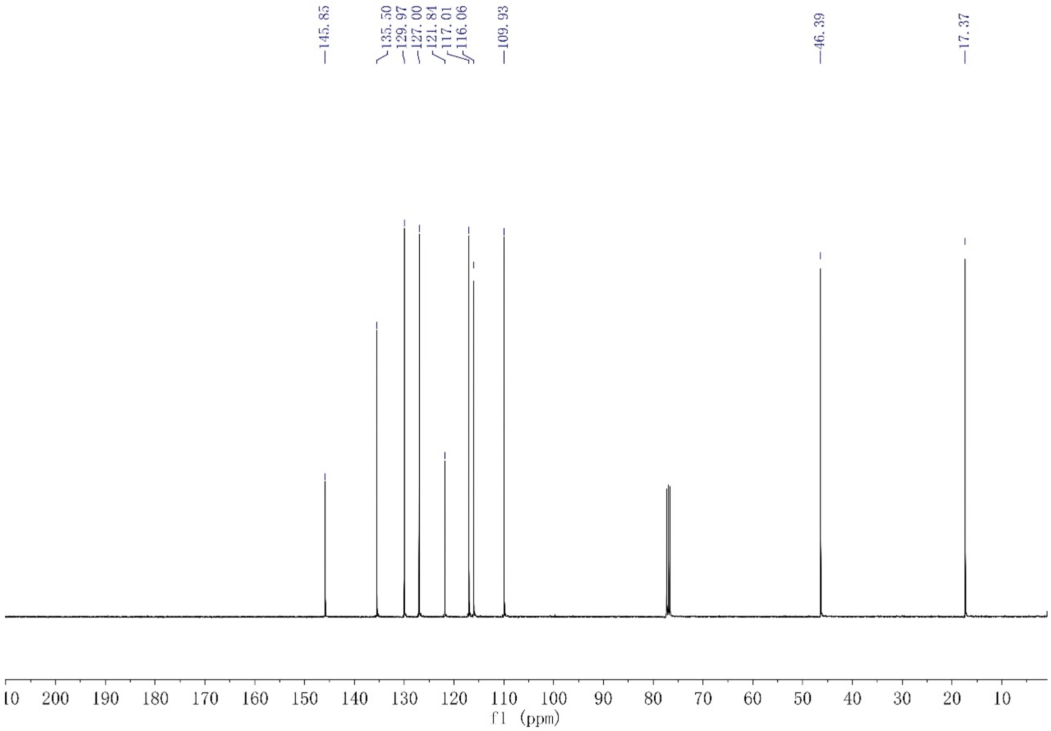

S2c

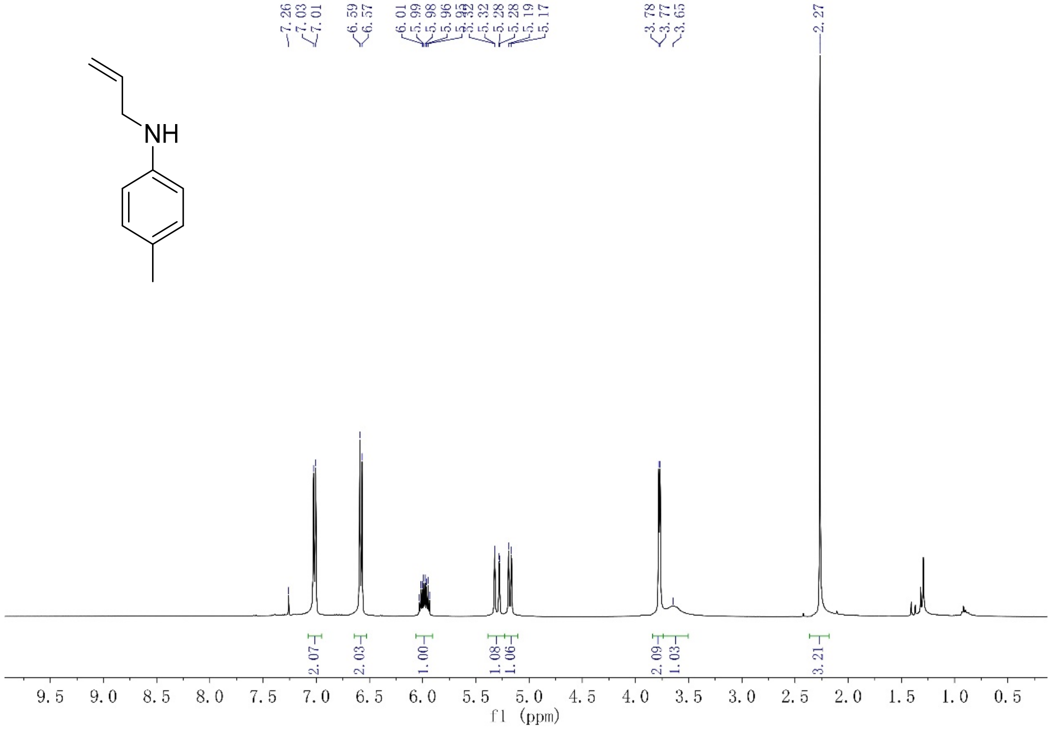
NH
7.26
7.03
7.01
6.59
6.57
6.01
5.99
5.98
5.96
5.32
5.28
5.28
5.19
5.17
3.78
3.77
3.65
2.27
2.07
2.03
1.00
1.08
1.06
2.09
1.03
3.21
9.5 9.0 8.5 8.0 7.5 7.0 6.5 6.0 5.5 5.0 4.5 4.0 3.5 3.0 2.5 2.0 1.5 1.0 0.5
f1 (ppm)

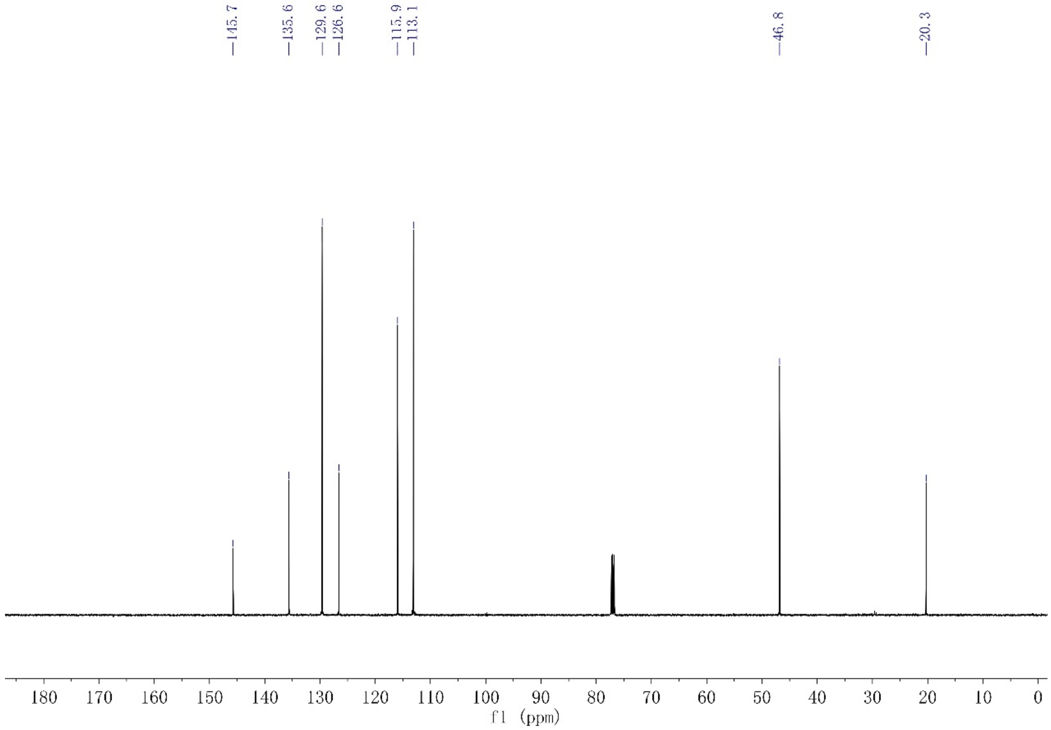
145.7
135.6
129.6
126.6
115.9
113.1
46.8
20.3
180 170 160 150 140 130 120 110 100 90 80 70 60 50 40 30 20 10 0
f1 (ppm)

S2d

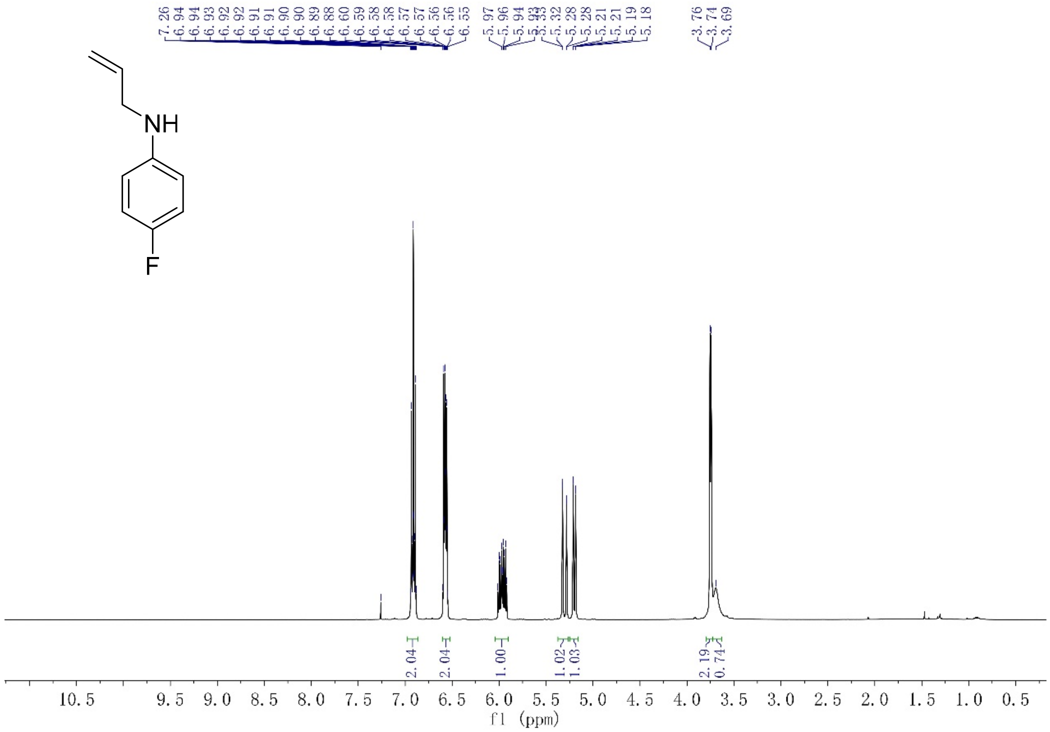
NH
F
7.26 6.94 6.93 6.92 6.91 6.90 6.89 6.88 6.60 6.59 6.58 6.58 6.57 6.56 6.55
5.97 5.96 5.94 5.93 5.33 5.32 5.28 5.28 5.21 5.19 5.18
3.76 3.74 3.69
2.04 2.04 1.00 1.02 1.03 2.19 0.74
10.5 9.5 9.0 8.5 8.0 7.5 7.0 6.5 6.0 5.5 5.0 4.5 4.0 3.5 3.0 2.5 2.0 1.5 1.0 0.5
f1 (ppm)

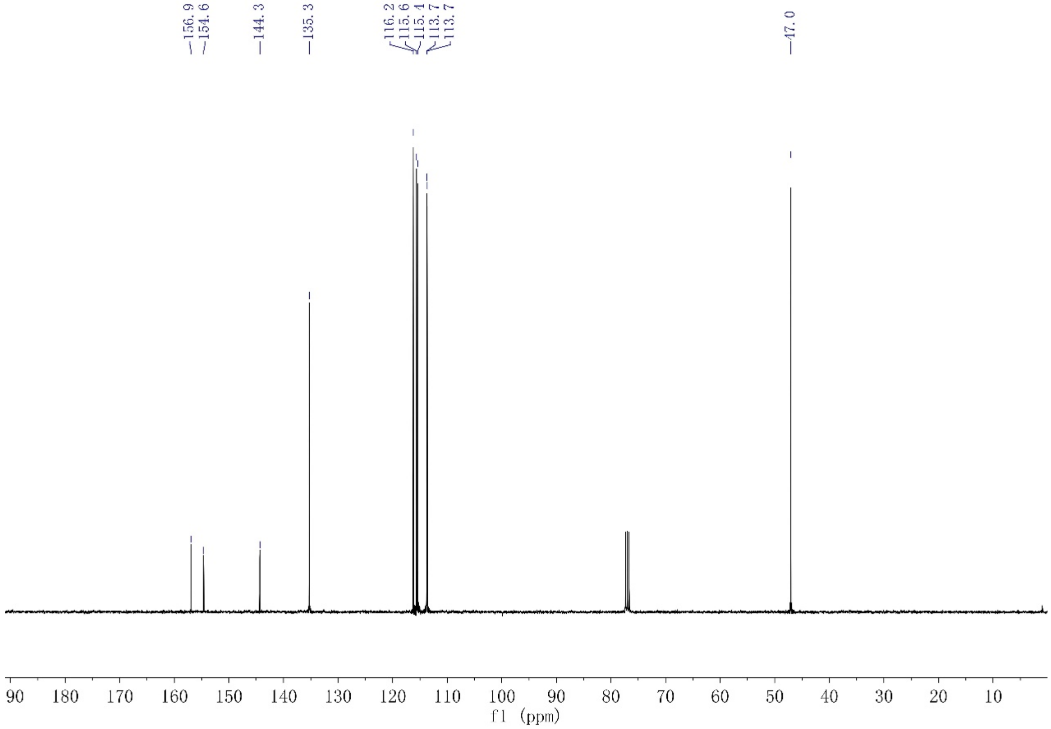
156.9 154.6
144.3
135.3
116.2 115.6 115.4 113.7 113.7
47.0
90 180 170 160 150 140 130 120 110 100 90 80 70 60 50 40 30 20 10
f1 (ppm)

S2e

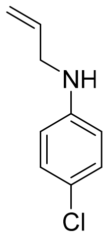
NH
Cl

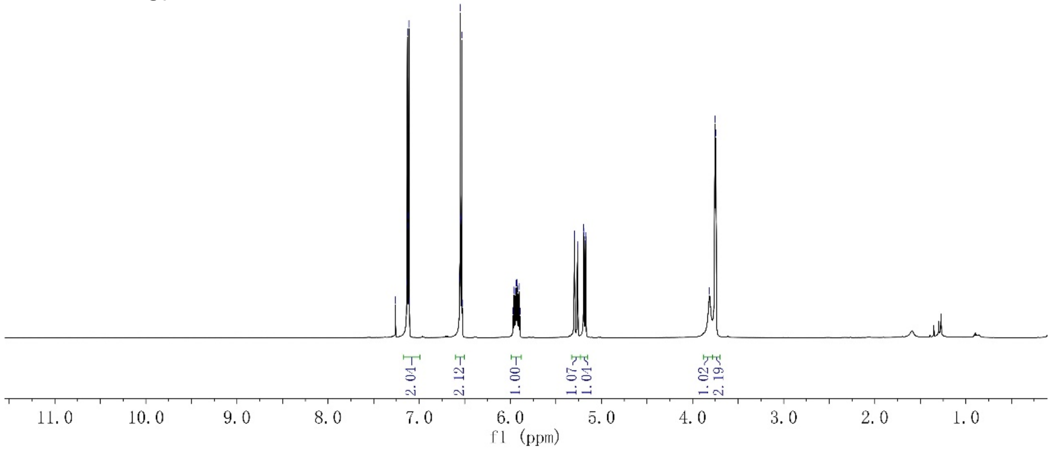
7.26
7.14
7.13
7.13
7.12
7.11
7.11
6.55
6.54
6.53
5.94
5.93
5.90
5.30
5.29
5.26
5.26
5.19
5.19
5.17
5.17
3.81
3.75
3.74
2.04
2.12
1.00
1.07
1.04
1.02
2.19
11.0 10.0 9.0 8.0 7.0 6.0 5.0 4.0 3.0 2.0 1.0
f1 (ppm)

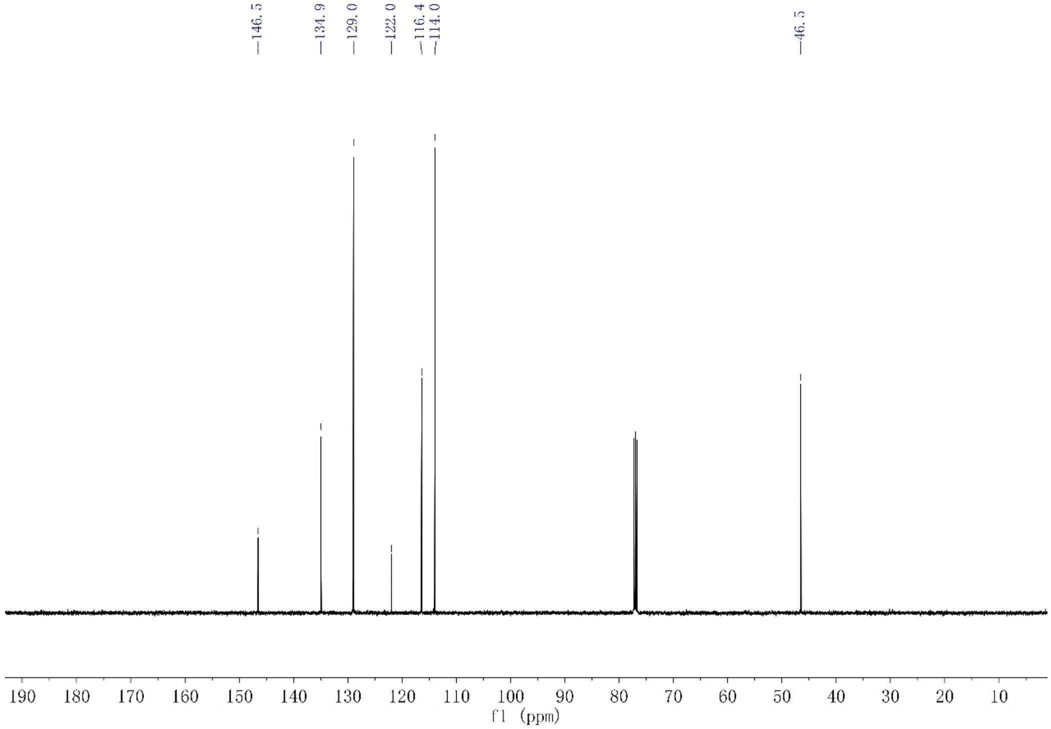
146.5
134.9
129.0
122.0
116.4
114.0
46.5
190 180 170 160 150 140 130 120 110 100 90 80 70 60 50 40 30 20 10
f1 (ppm)

S2f

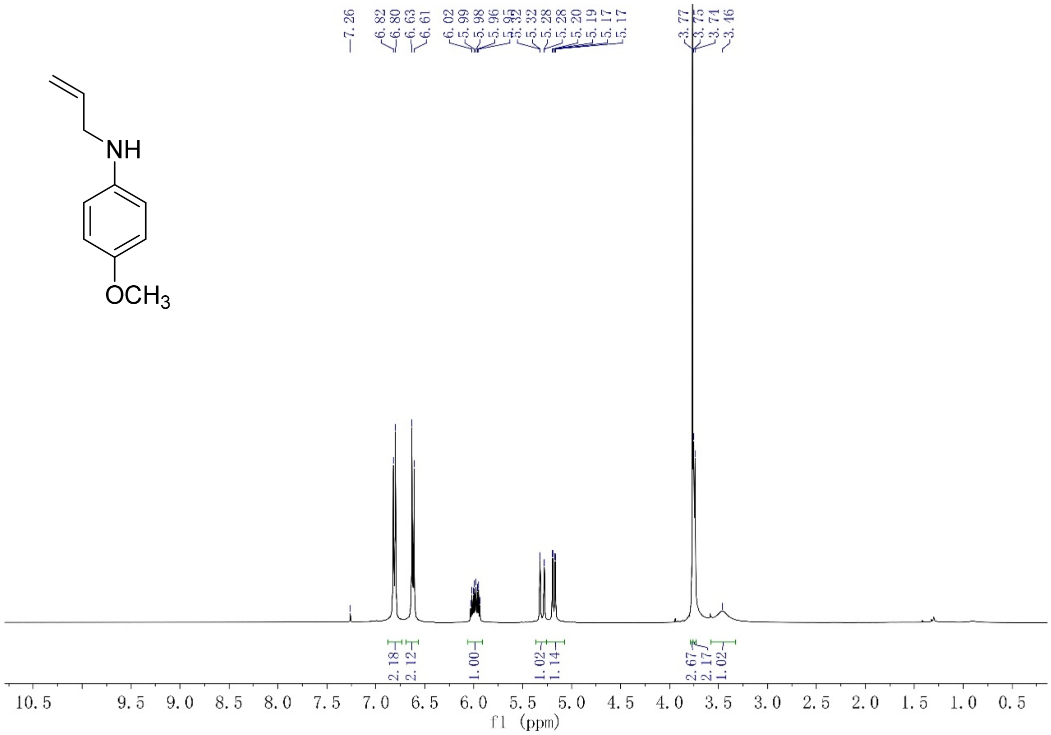
NH
OCH3
7.26
6.82
6.80
6.63
6.61
6.02
5.99
5.98
5.96
5.32
5.28
5.20
5.19
5.17
5.17
3.77
3.71
3.46
2.18
2.12
1.00
1.02
1.14
2.67
2.17
1.02
10.5 9.5 9.0 8.5 8.0 7.5 7.0 6.5 6.0 5.5 5.0 4.5 4.0 3.5 3.0 2.5 2.0 1.5 1.0 0.5
f1 (ppm)

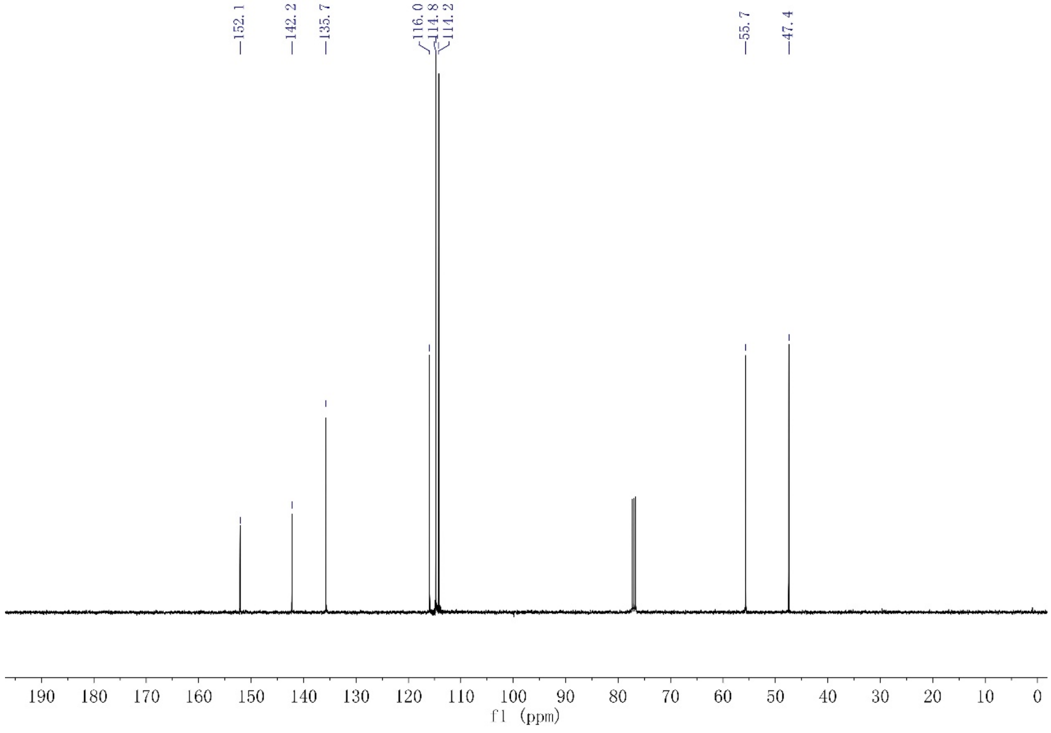
152.1
142.2
135.7
116.0
114.8
114.2
55.7
47.4
190 180 170 160 150 140 130 120 110 100 90 80 70 60 50 40 30 20 10 0
f1 (ppm)

S3a

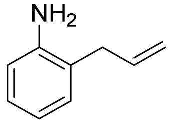

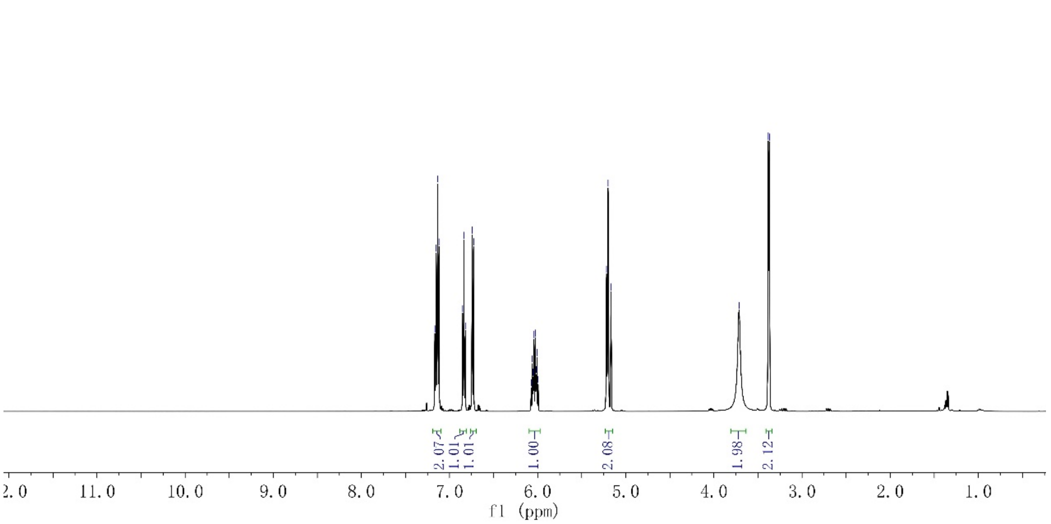

7.17
7.15
7.14
7.12
6.85
6.84
6.82
6.74
6.73
6.06
6.01
6.03
6.02
6.01
5.22
5.20
5.17
3.72
3.38
3.37
2.07
1.01
1.01
1.00
2.08
1.98
2.12
f1 (ppm)

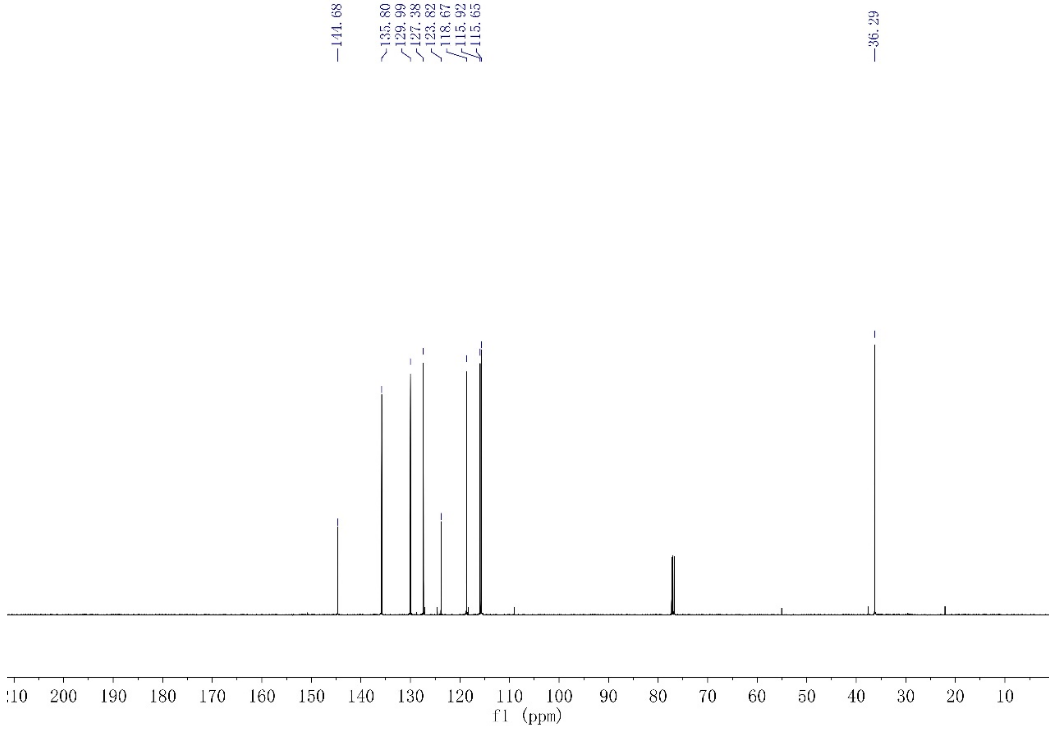

144.68
135.80
129.99
127.38
123.82
118.67
115.92
115.65
36.29
f1 (ppm)

S3b

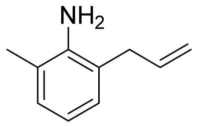

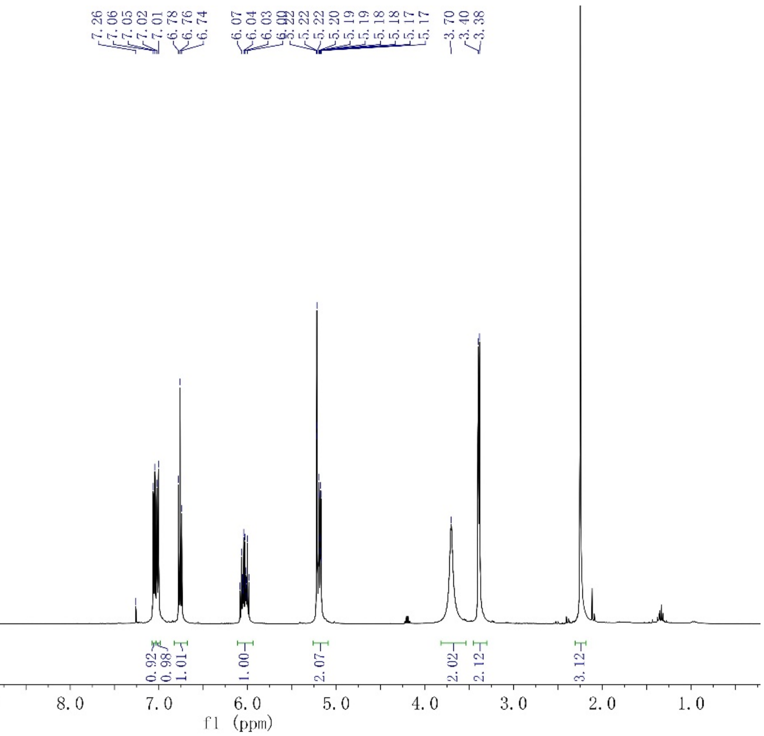
7.26
7.06
7.05
7.02
7.01
6.78
6.76
6.74
6.07
6.04
6.03
5.22
5.22
5.20
5.19
5.19
5.18
5.17
5.17
3.70
3.40
3.38
0.92
0.98
1.01
1.00
2.07
2.02
2.12
3.12
f1 (ppm)

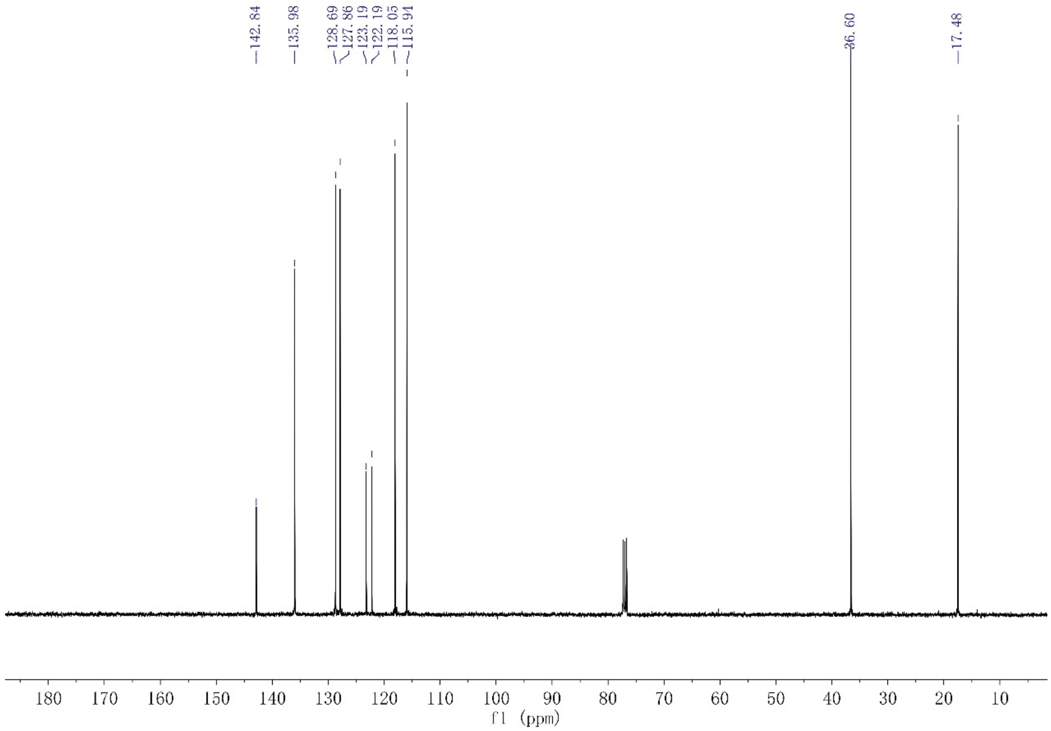
142.84
135.98
128.69
127.86
123.19
122.19
118.05
115.94
36.60
17.48
f1 (ppm)

S3c

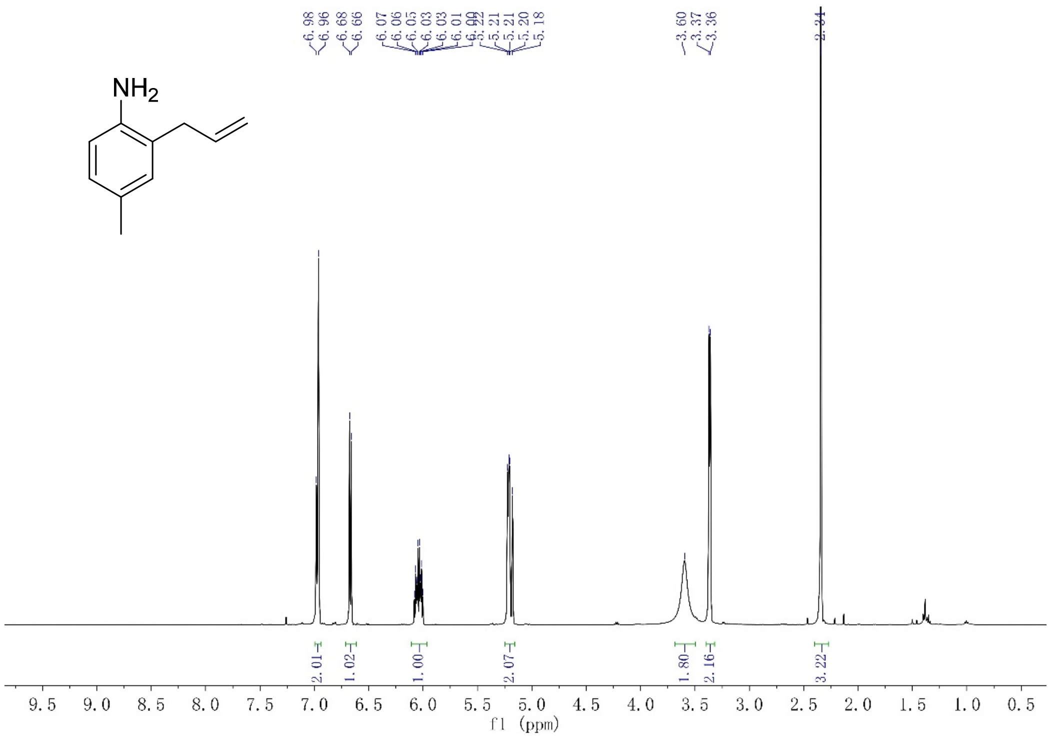

NH2
6.98
6.96
6.68
6.66
6.07
6.06
6.05
6.03
6.03
6.01
5.21
5.21
5.20
5.18
3.60
3.37
3.36
2.01
1.02
1.00
2.07
1.80
2.16
3.22
9.5 9.0 8.5 8.0 7.5 7.0 6.5 6.0 5.5 5.0 4.5 4.0 3.5 3.0 2.5 2.0 1.5 1.0 0.5
f1 (ppm)

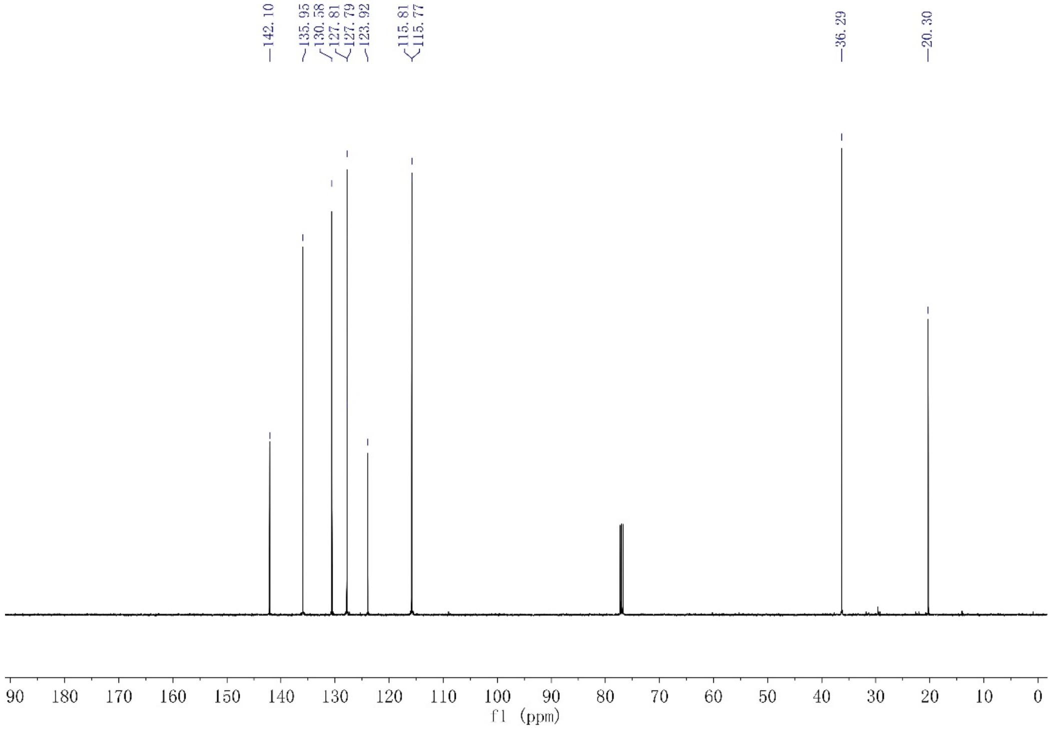

142.10
135.95
130.58
127.81
127.79
123.92
115.81
115.77
36.29
20.30
90 180 170 160 150 140 130 120 110 100 90 80 70 60 50 40 30 20 10 0
f1 (ppm)

S3d

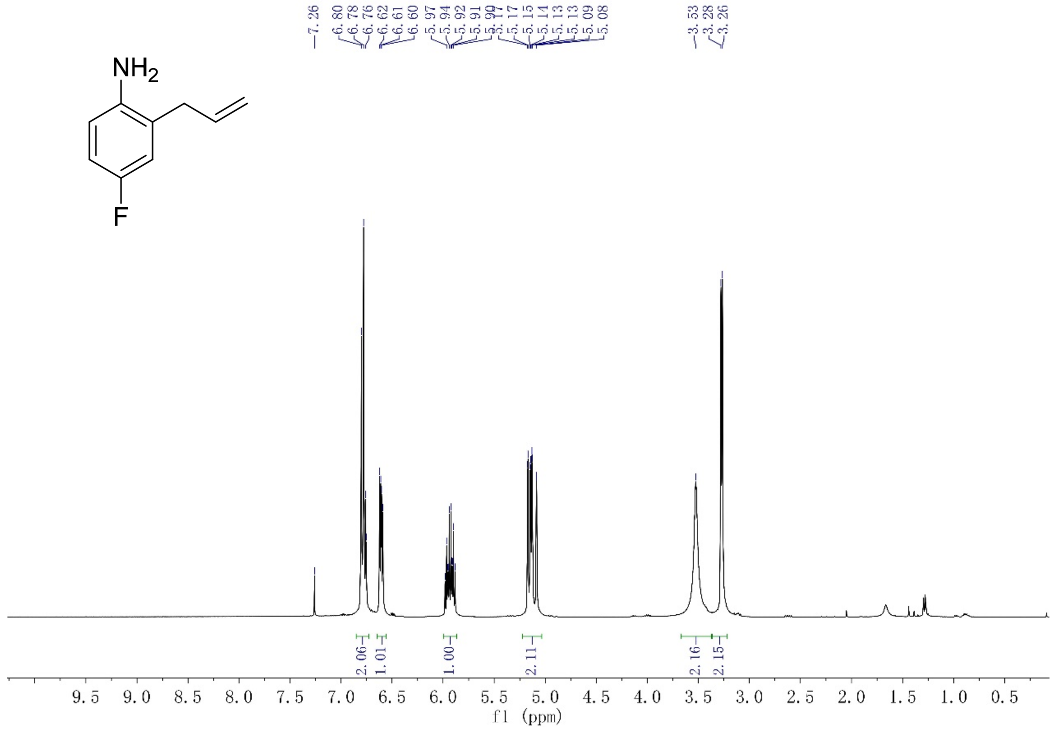

7.26
6.80
6.78
6.76
6.62
6.61
6.60
5.97
5.94
5.92
5.91
5.90
5.17
5.15
5.14
5.13
5.13
5.09
5.08
3.53
3.28
3.26
2.06
1.01
1.00
2.11
2.16
2.15
9.5 9.0 8.5 8.0 7.5 7.0 6.5 6.0 5.5 5.0 4.5 4.0 3.5 3.0 2.5 2.0 1.5 1.0 0.5
f1 (ppm)

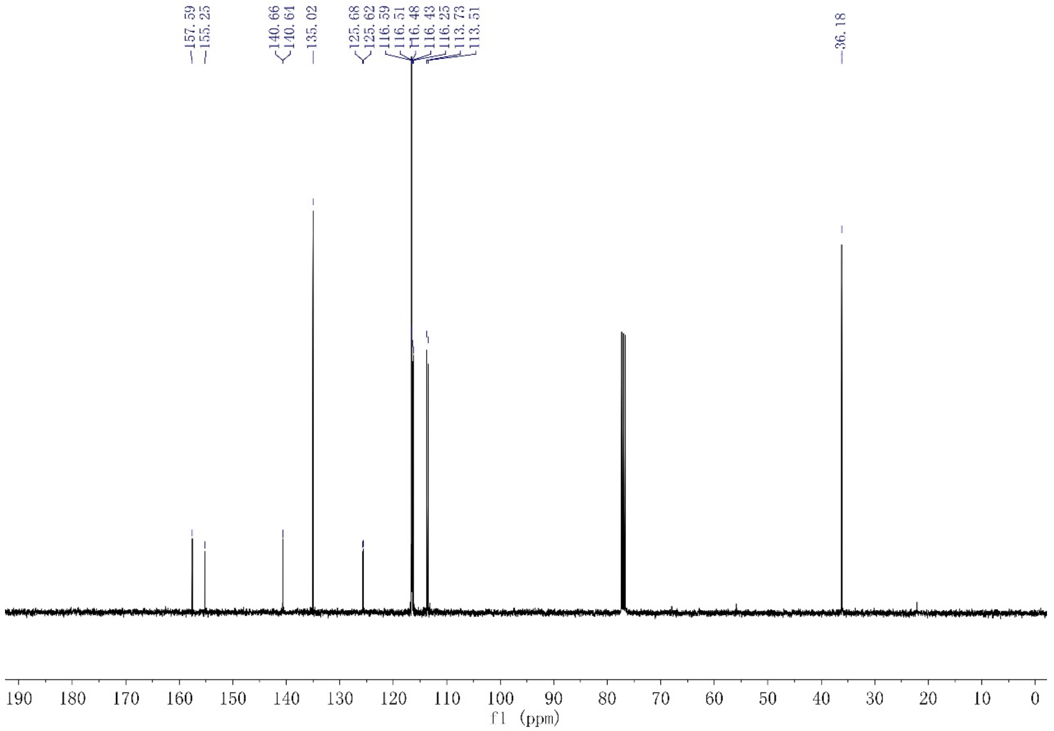

157.59
155.25
140.66
140.61
135.02
125.68
125.62
116.59
116.51
116.48
116.43
116.25
113.73
113.51
36.18
190 180 170 160 150 140 130 120 110 100 90 80 70 60 50 40 30 20 10 0
f1 (ppm)

S3e

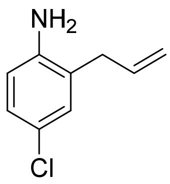

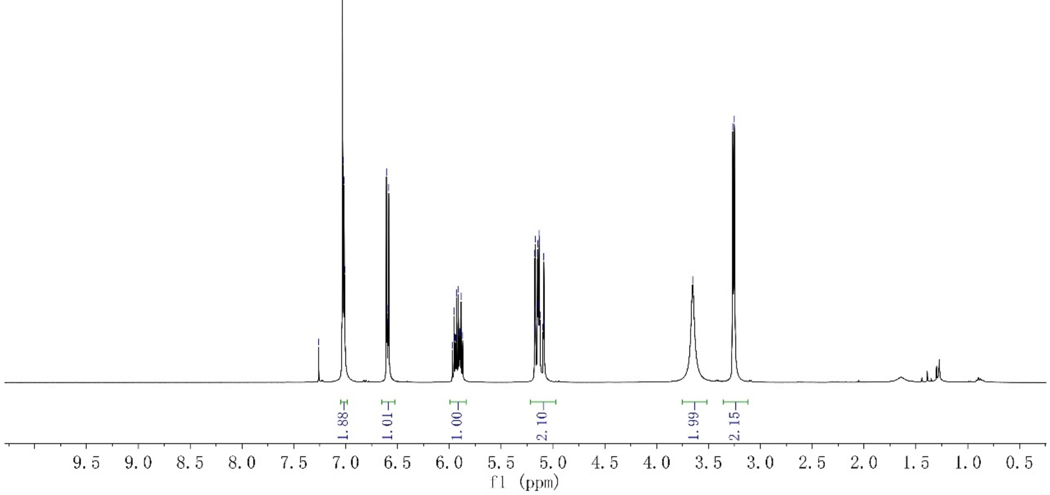

7.26
7.03
7.02
7.01
7.01
6.61
6.60
6.58
5.96
5.93
5.91
5.90
5.89
5.18
5.17
5.17
5.15
5.15
5.14
5.14
5.13
5.13
5.13
5.09
5.09
3.65
3.27
3.25
1.88
1.01
1.00
2.10
1.99
2.15
9.5 9.0 8.5 8.0 7.5 7.0 6.5 6.0 5.5 5.0 4.5 4.0 3.5 3.0 2.5 2.0 1.5 1.0 0.5
f1 (ppm)

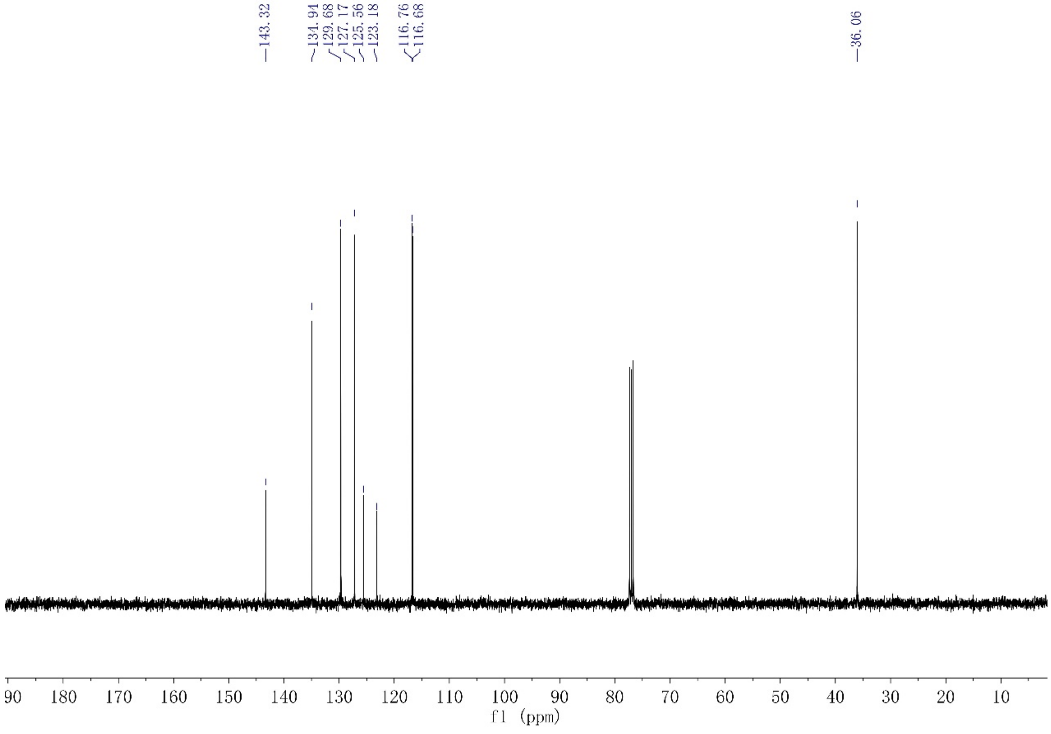

143.32
134.91
129.68
127.17
125.56
123.18
116.76
116.68
36.06
90 180 170 160 150 140 130 120 110 100 90 80 70 60 50 40 30 20 10
f1 (ppm)

S3f

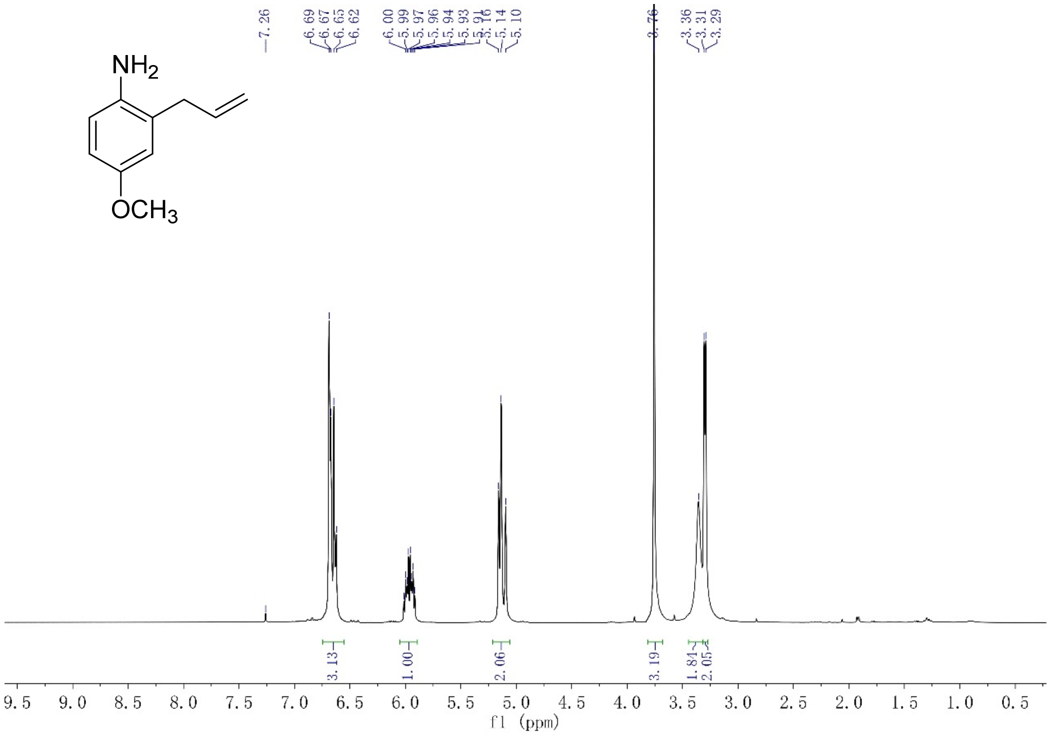

NH2
OCH3
7.26
6.69
6.67
6.65
6.62
6.00
5.99
5.97
5.96
5.94
5.93
5.91
5.16
5.14
5.10
3.76
3.36
3.31
3.29
3.13
1.00
2.06
3.19
1.84
2.05
9.5 9.0 8.5 8.0 7.5 7.0 6.5 6.0 5.5 5.0 4.5 4.0 3.5 3.0 2.5 2.0 1.5 1.0 0.5
f1 (ppm)

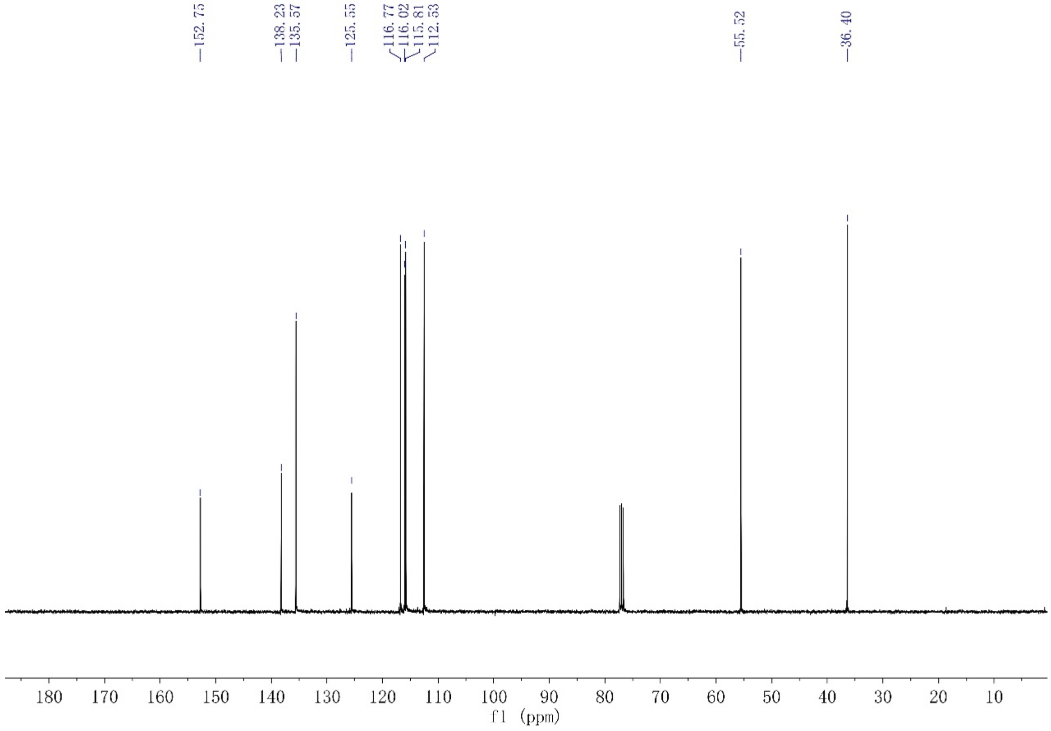

152.75
138.23
135.57
125.55
116.77
116.02
115.81
112.53
55.52
36.40
180 170 160 150 140 130 120 110 100 90 80 70 60 50 40 30 20 10
f1 (ppm)

1a

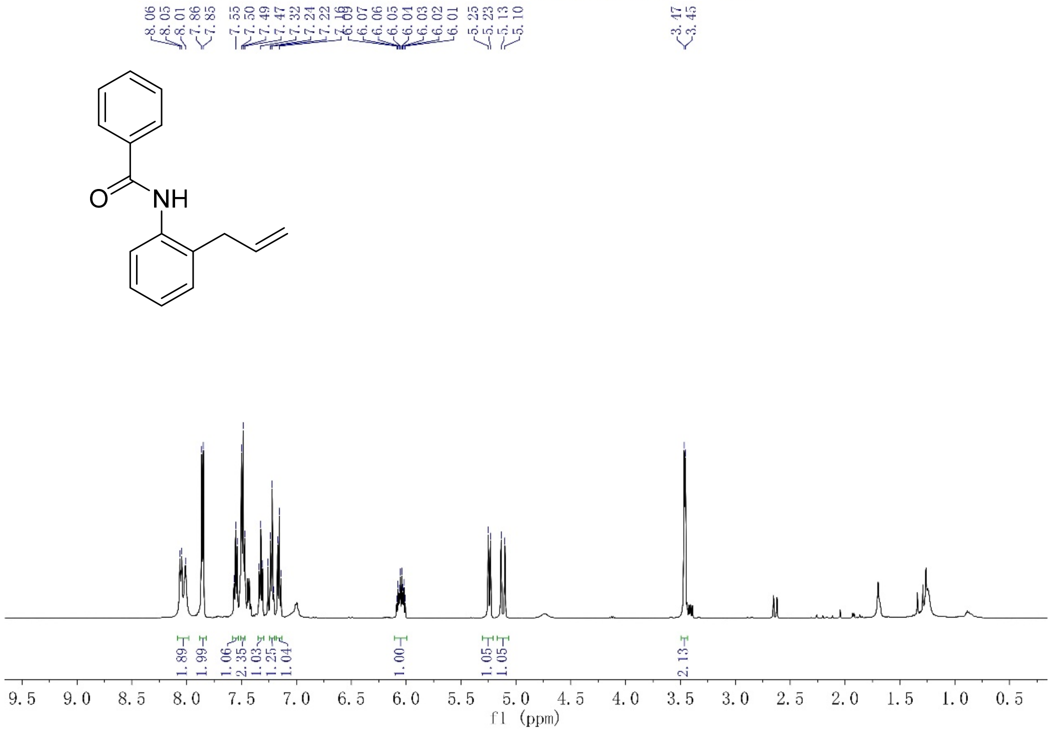

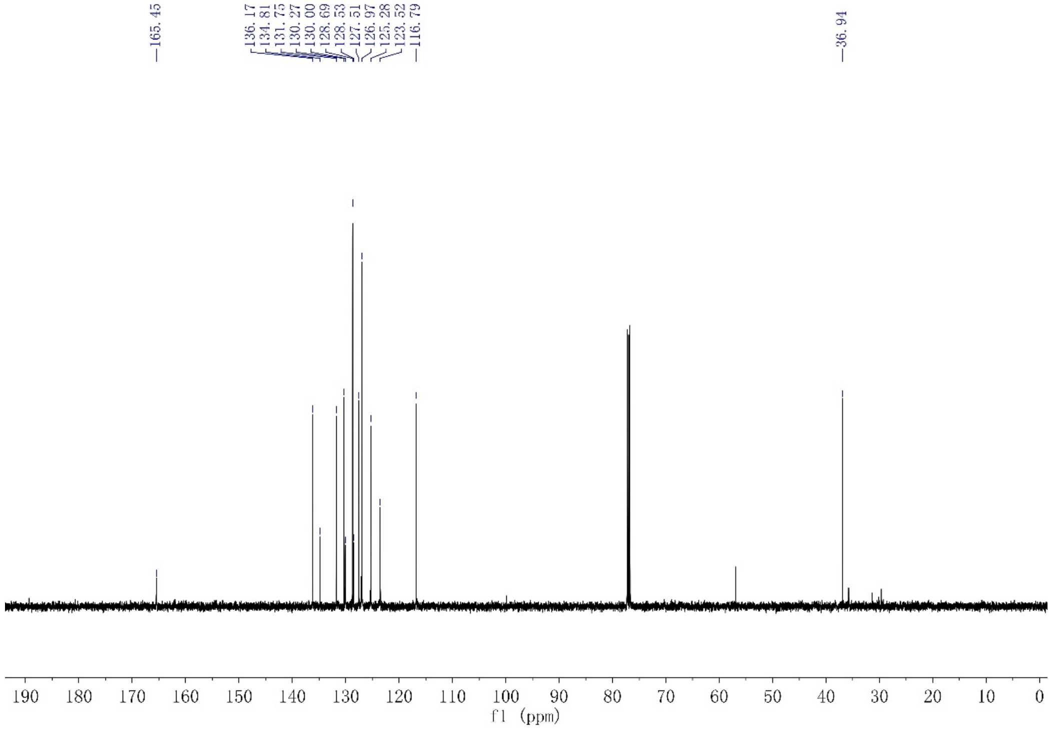

1b

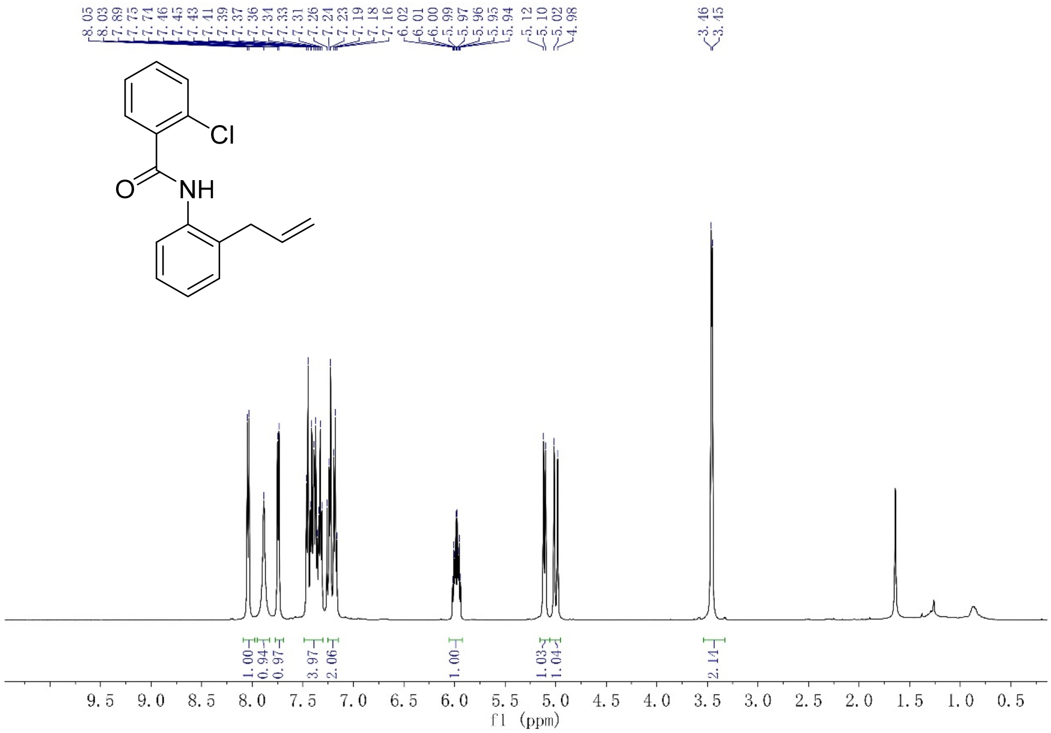
8.05
8.03
7.89
7.75
7.74
7.46
7.45
7.43
7.41
7.39
7.37
7.36
7.34
7.33
7.31
7.26
7.24
7.23
7.19
7.18
7.16
6.02
6.01
6.00
5.99
5.97
5.96
5.95
5.94
5.12
5.10
5.02
4.98
3.46
3.45
Cl
O
NH
1.00
0.94
0.97
3.97
2.06
1.00
1.03
1.04
2.14
9.5 9.0 8.5 8.0 7.5 7.0 6.5 6.0 5.5 5.0 4.5 4.0 3.5 3.0 2.5 2.0 1.5 1.0 0.5
f1 (ppm)

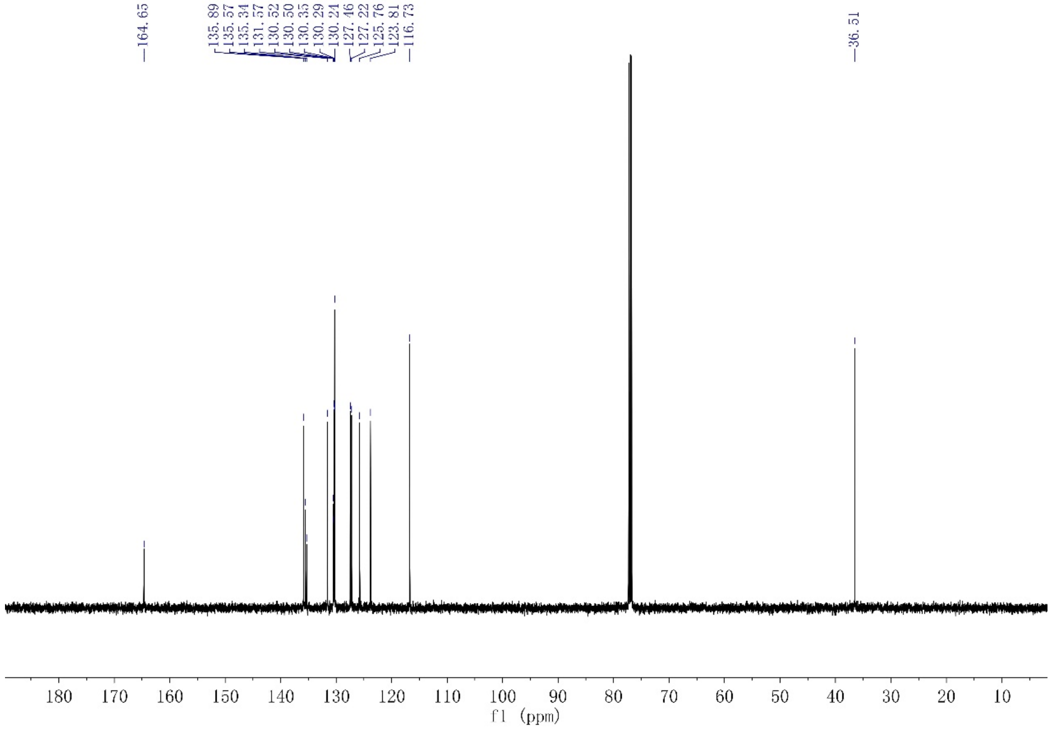
164.65
135.89
135.57
135.34
131.57
130.52
130.50
130.35
130.29
130.21
127.46
127.22
125.76
123.81
116.73
36.51
180 170 160 150 140 130 120 110 100 90 80 70 60 50 40 30 20 10
f1 (ppm)

1c

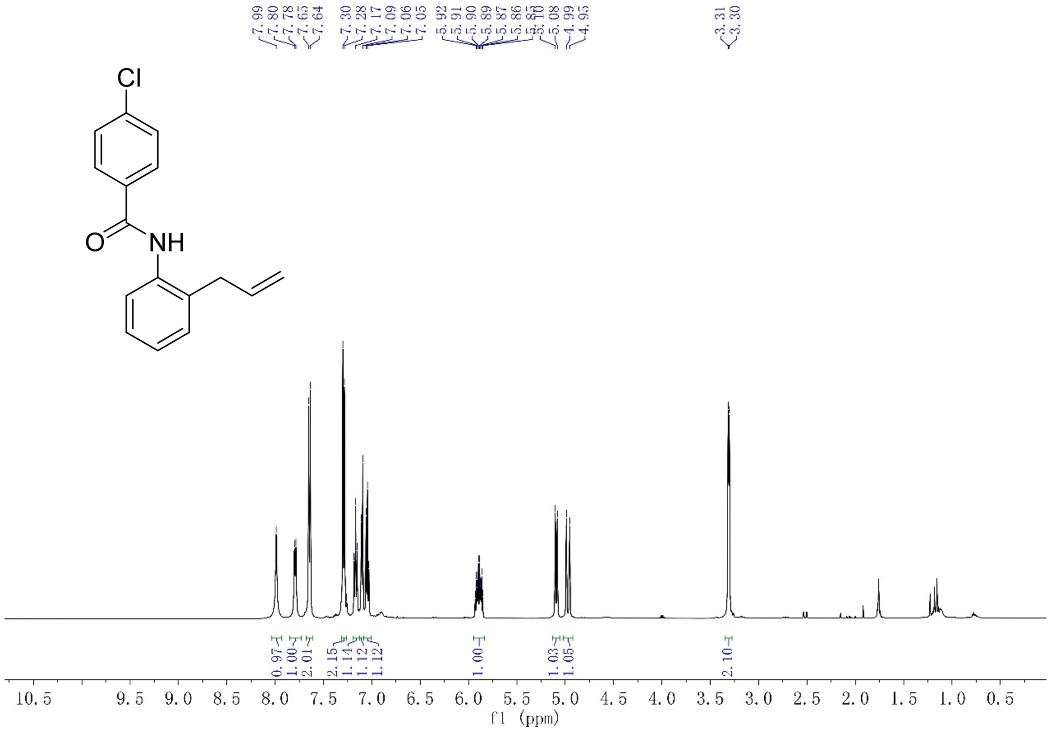

Cl
O
NH
7.99
7.80
7.78
7.65
7.64
7.30
7.28
7.17
7.09
7.06
7.05
5.92
5.91
5.90
5.89
5.87
5.86
5.85
5.10
5.08
4.99
4.95
3.31
3.30
0.97
1.00
2.01
2.15
1.14
1.12
1.12
1.00
1.03
1.05
2.10
10.5 9.5 9.0 8.5 8.0 7.5 7.0 6.5 6.0 5.5 5.0 4.5 4.0 3.5 3.0 2.5 2.0 1.5 1.0 0.5
f1 (ppm)

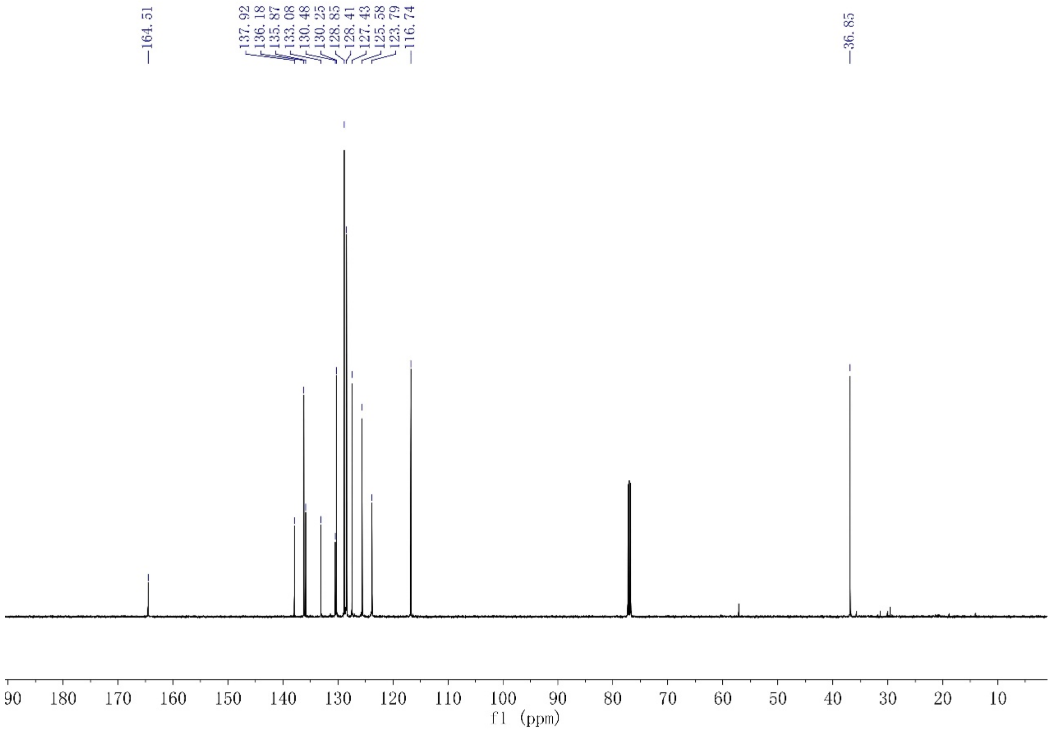

164.51
137.92
136.18
135.87
133.08
130.48
130.25
128.85
128.41
127.43
125.58
123.79
116.74
36.85
90 180 170 160 150 140 130 120 110 100 90 80 70 60 50 40 30 20 10
f1 (ppm)

1d

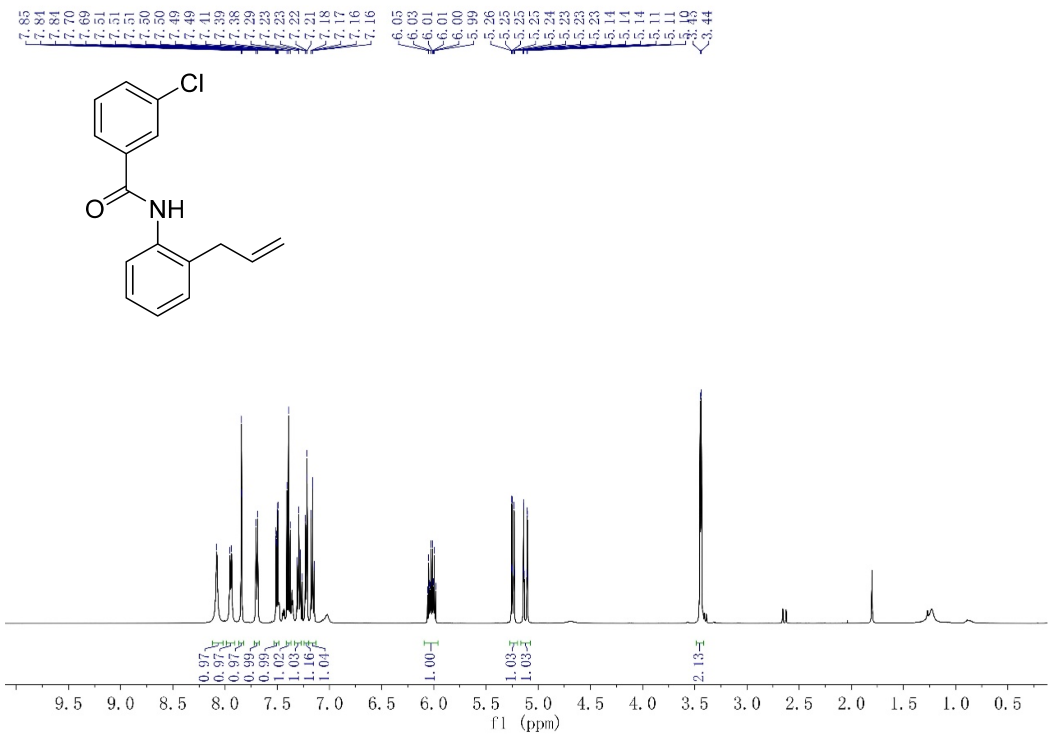

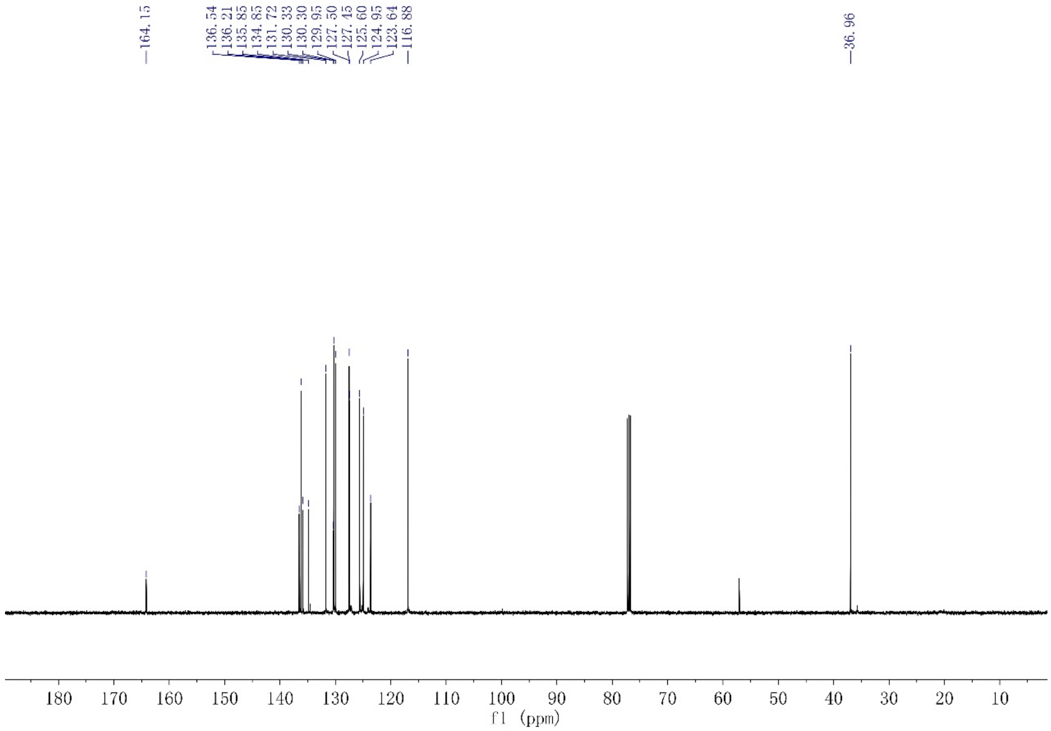

1e

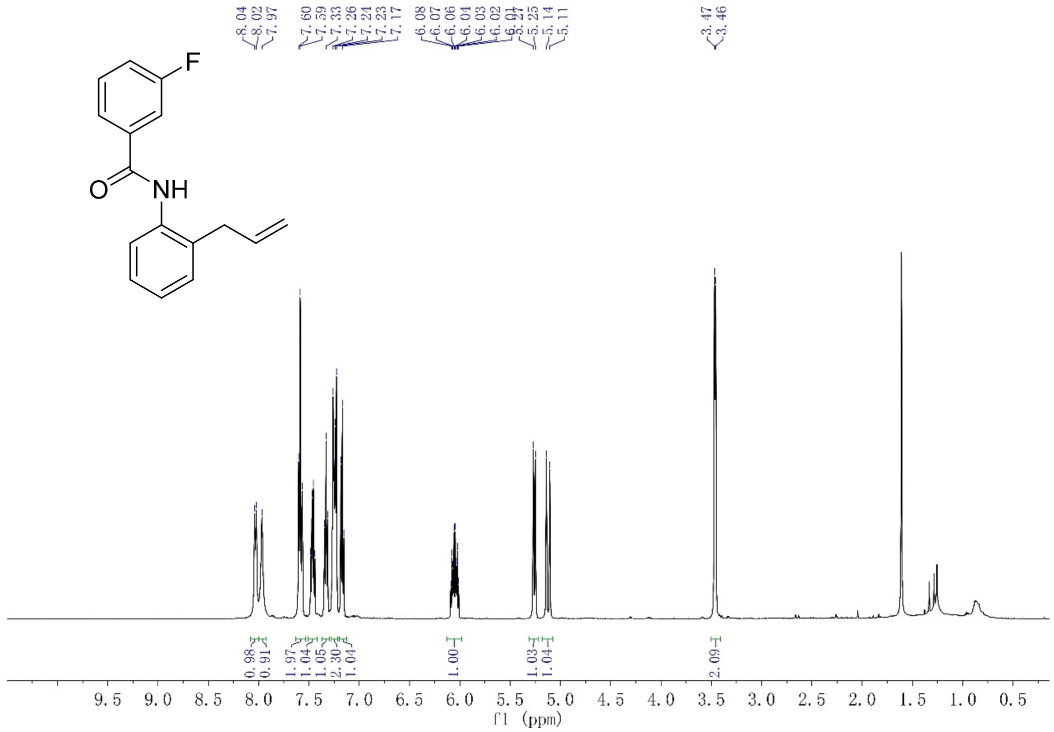
F
O
NH
8.04
8.02
7.97
7.60
7.59
7.33
7.26
7.21
7.23
7.17
6.08
6.07
6.06
6.04
6.03
6.02
5.25
5.14
5.11
3.47
3.46
0.98
0.91
1.97
1.04
1.05
2.30
1.04
1.00
1.03
1.04
2.09
9.5 9.0 8.5 8.0 7.5 7.0 6.5 6.0 5.5 5.0 4.5 4.0 3.5 3.0 2.5 2.0 1.5 1.0 0.5
f1 (ppm)

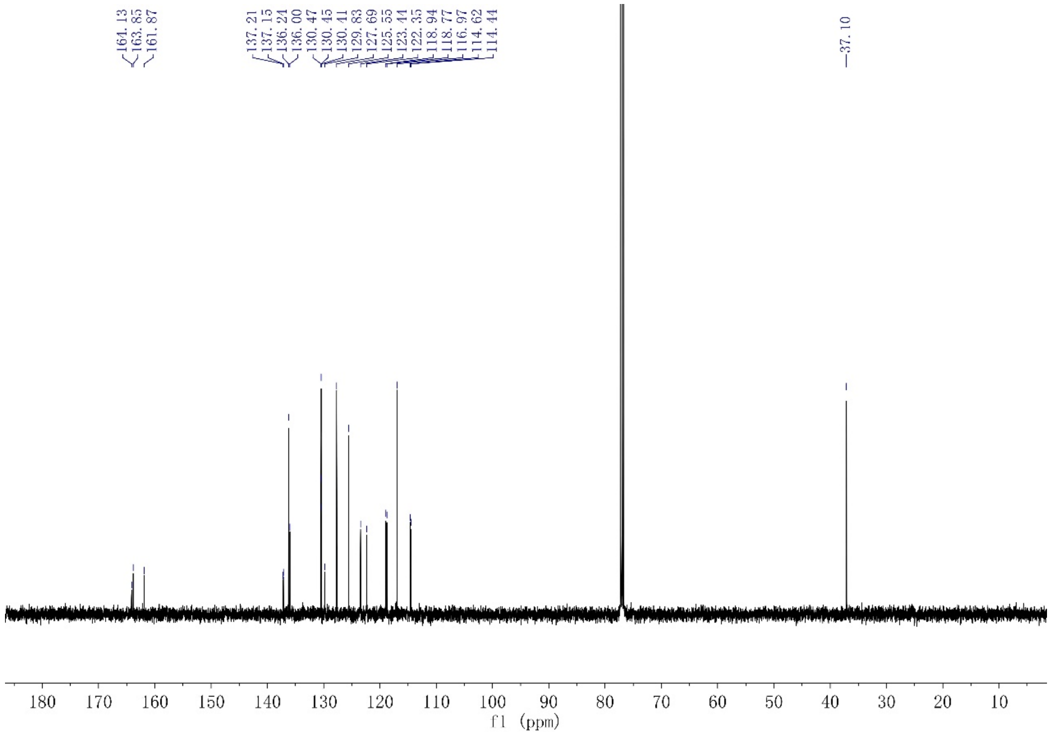
164.13
163.85
161.87
137.21
137.15
136.21
136.00
130.47
130.45
130.41
129.83
127.69
125.55
123.41
122.35
118.94
118.77
116.97
114.62
114.44
37.10
180 170 160 150 140 130 120 110 100 90 80 70 60 50 40 30 20 10
f1 (ppm)

1f

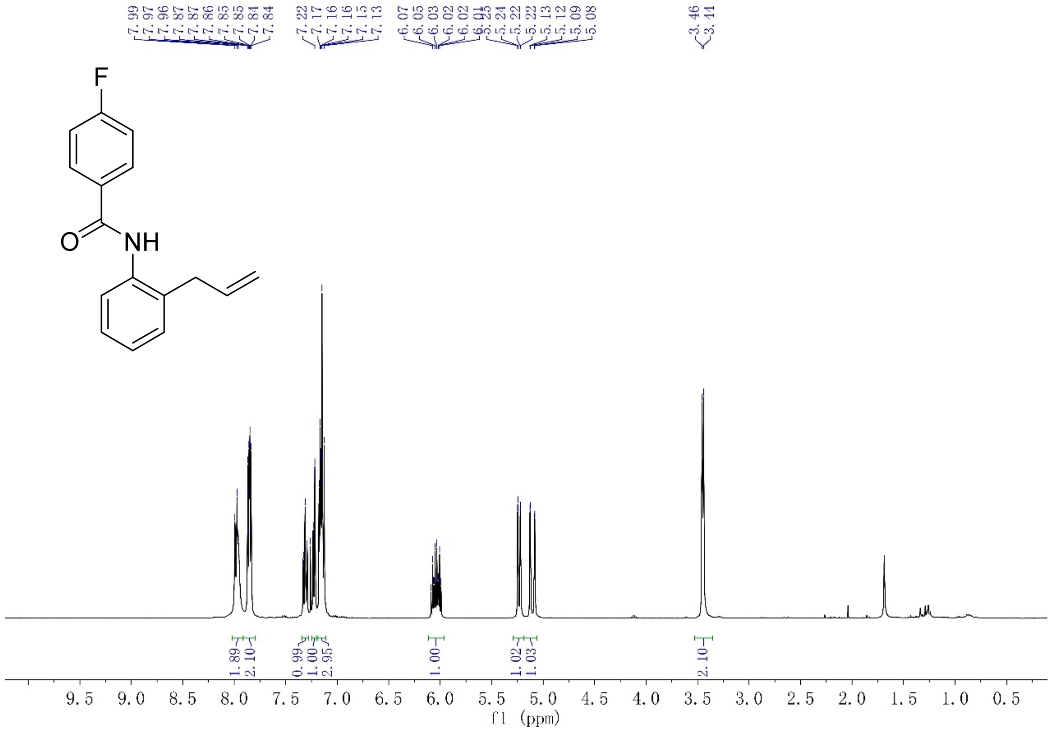

F
O
NH
7.99
7.97
7.96
7.87
7.87
7.86
7.85
7.85
7.84
7.84
7.22
7.17
7.16
7.16
7.15
7.13
6.07
6.05
6.03
6.02
6.02
6.01
5.25
5.24
5.22
5.22
5.13
5.12
5.09
5.08
3.46
3.41
1.89
2.10
0.99
1.00
2.95
1.00
1.02
1.03
2.10
9.5 9.0 8.5 8.0 7.5 7.0 6.5 6.0 5.5 5.0 4.5 4.0 3.5 3.0 2.5 2.0 1.5 1.0 0.5
f1 (ppm)

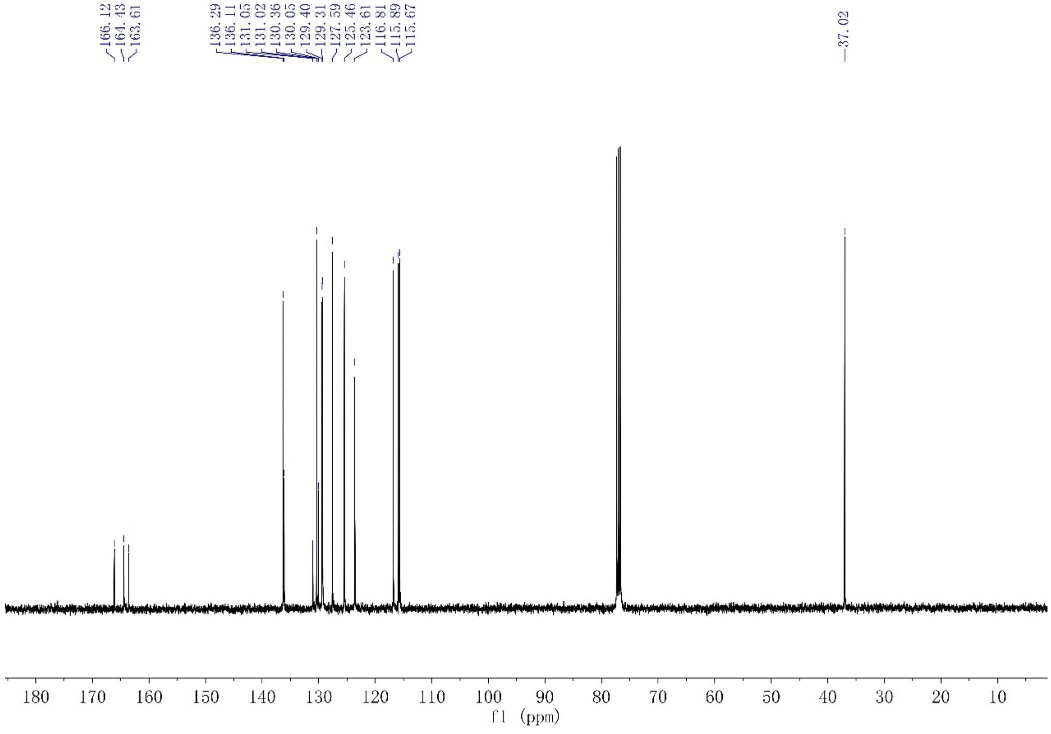

166.12
164.43
163.61
136.29
136.11
131.05
131.02
130.36
130.05
129.40
129.31
127.59
125.46
123.61
116.81
115.89
115.67
37.02
180 170 160 150 140 130 120 110 100 90 80 70 60 50 40 30 20 10
f1 (ppm)

1g

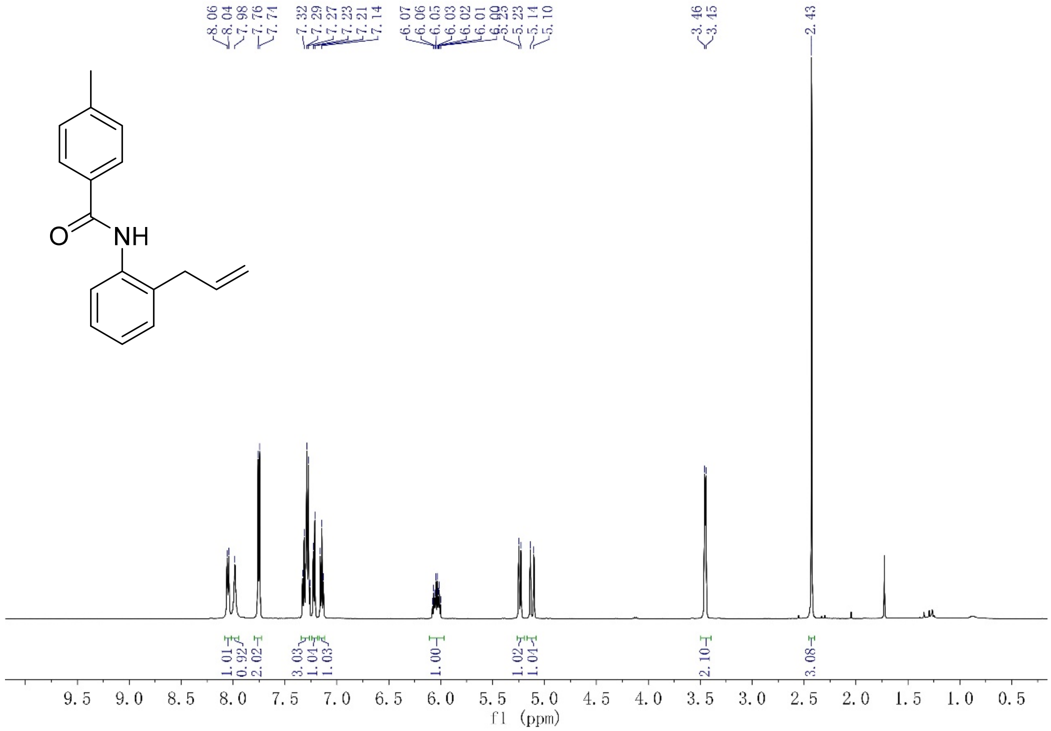

8.06
8.04
7.98
7.76
7.74
7.32
7.29
7.27
7.23
7.21
7.14
6.07
6.06
6.05
6.03
6.02
6.01
5.23
5.14
5.10
3.46
3.45
2.43
NH
O
1.01
0.92
2.02
3.03
1.04
1.03
1.00
1.02
1.04
2.10
3.08
f1 (ppm)

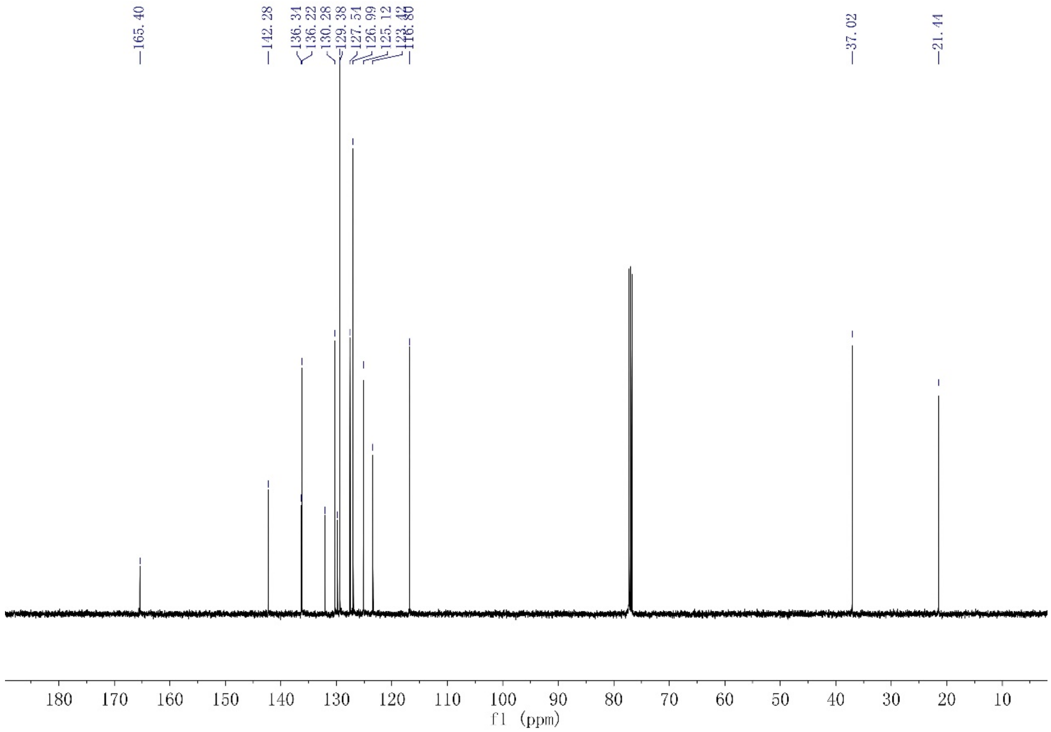

165.40
142.28
136.31
136.22
130.28
129.38
127.51
126.99
125.12
37.02
21.44
f1 (ppm)

1h

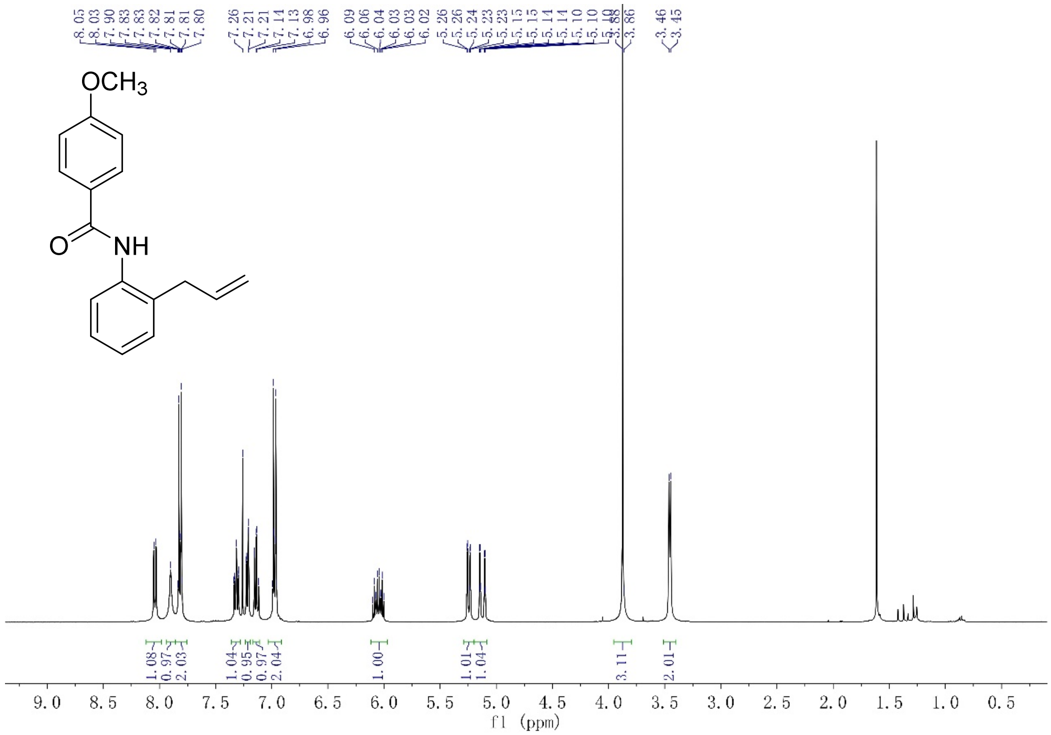
OCH3
O
NH
f1 (ppm)

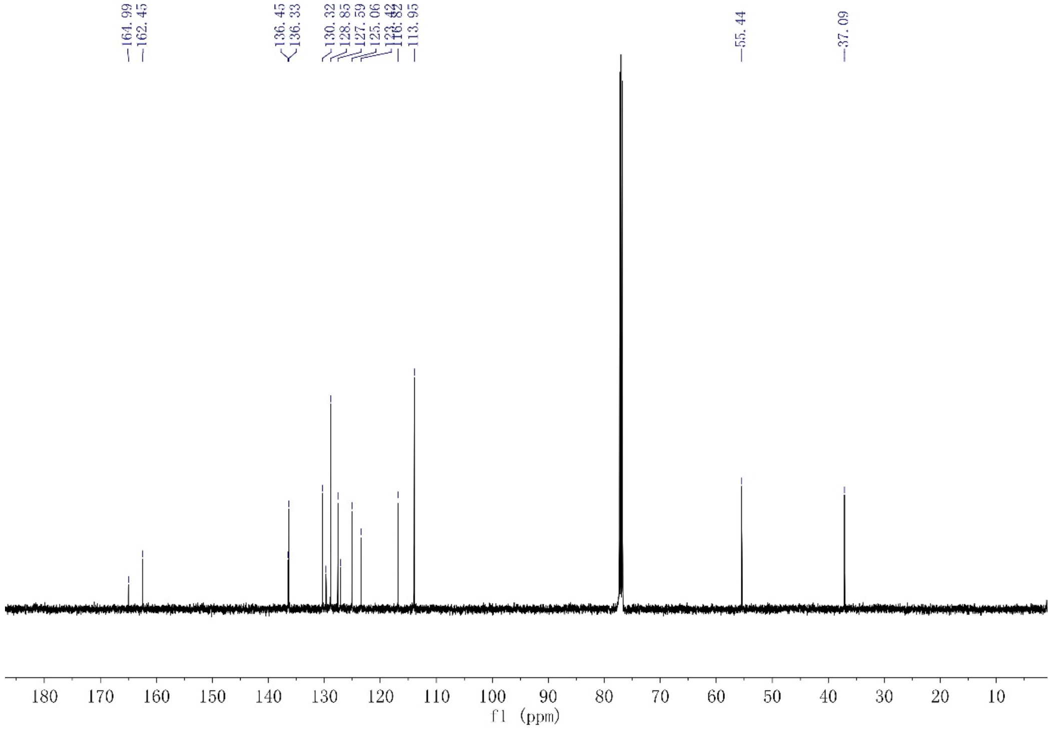
164.99
162.45
136.45
136.33
130.32
128.85
127.59
125.06
113.95
55.44
37.09
f1 (ppm)

1i

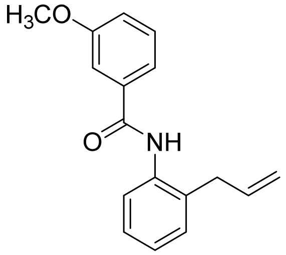

H_3CO
O
NH

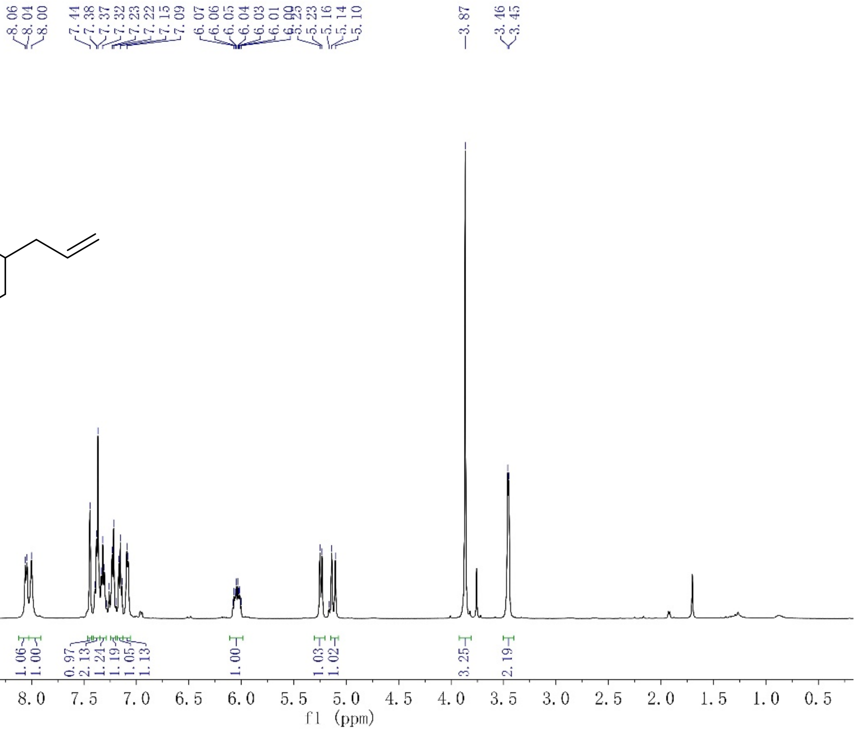

8.06
8.01
8.00
7.44
7.38
7.37
7.32
7.23
7.22
7.15
7.09
6.07
6.06
6.05
6.04
6.03
6.01
5.23
5.16
5.14
5.10
3.87
3.46
3.45
1.06
1.00
0.97
2.13
1.24
1.19
1.05
1.13
1.00
1.03
1.02
3.25
2.19
f1 (ppm)

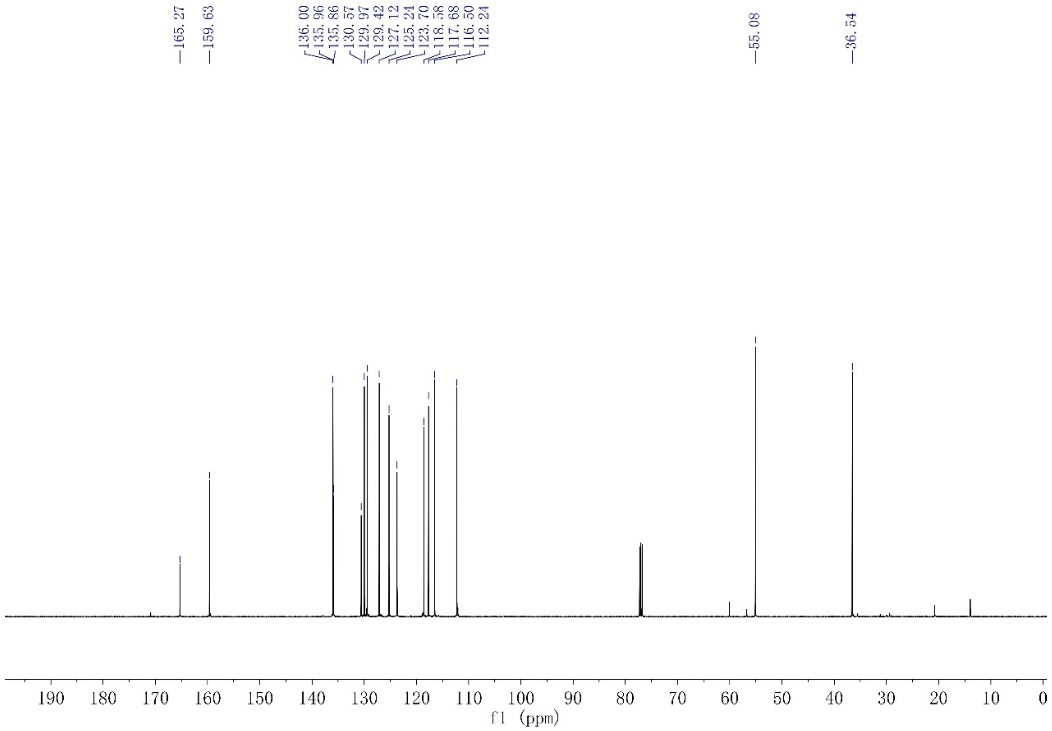

165.27
159.63
136.00
135.96
135.86
130.57
129.97
129.42
127.12
125.21
123.70
118.58
117.68
116.50
112.24
55.08
36.54
f1 (ppm)

1j

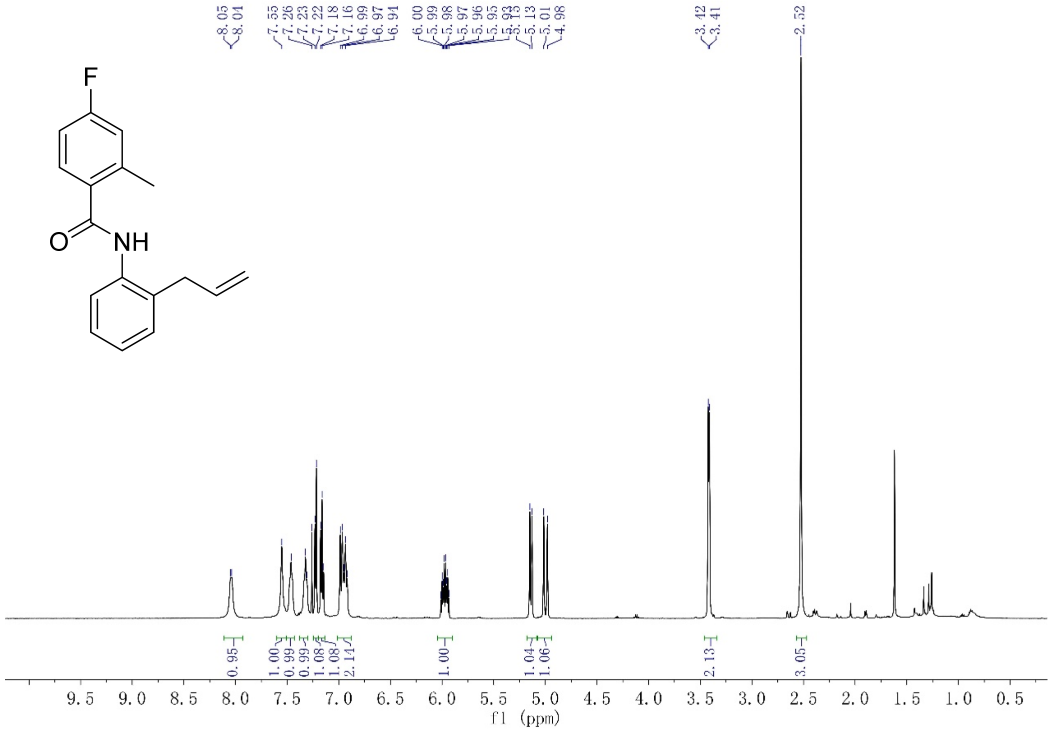
F
O
NH
8.05
8.01
7.55
7.26
7.23
7.22
7.18
7.16
6.99
6.97
6.94
6.00
5.99
5.98
5.97
5.96
5.95
5.93
5.13
5.13
5.01
4.98
3.42
3.41
2.52
0.95
1.00
0.99
0.99
1.08
1.08
2.14
1.00
1.04
1.06
2.13
3.05
9.5
9.0
8.5
8.0
7.5
7.0
6.5
6.0
5.5
5.0
4.5
4.0
3.5
3.0
2.5
2.0
1.5
1.0
0.5
f1 (ppm)

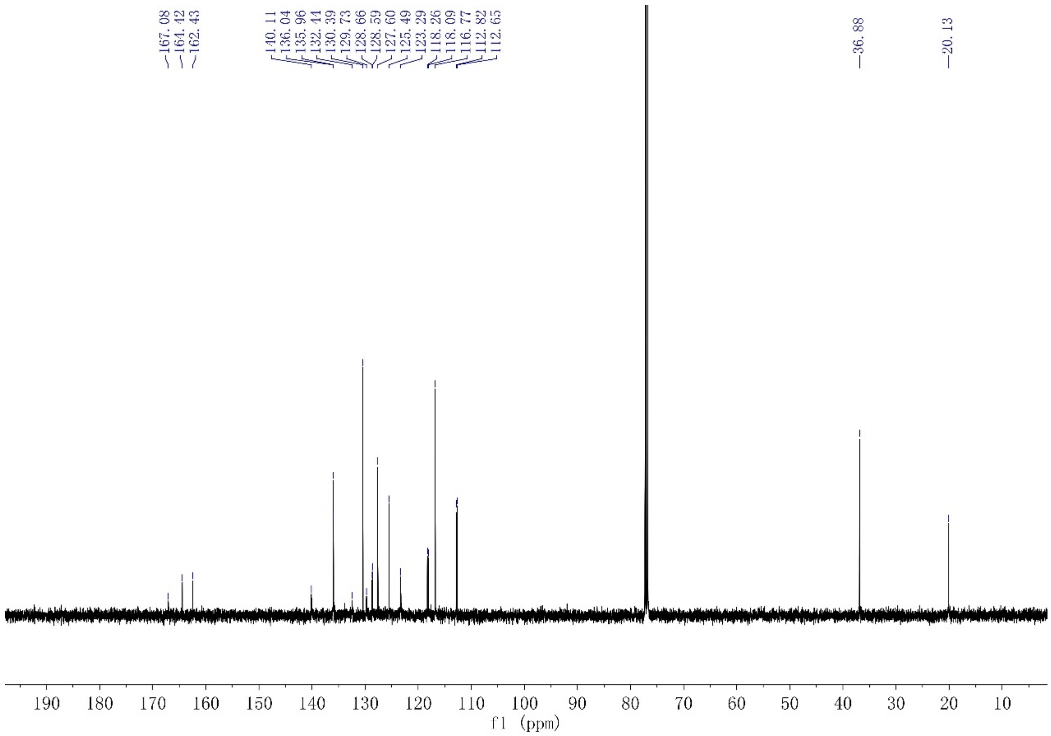
167.08
164.42
162.43
140.11
136.04
135.96
132.41
130.39
129.73
128.66
128.59
127.60
125.49
123.29
118.26
118.09
116.77
112.82
112.65
36.88
20.13
190
180
170
160
150
140
130
120
110
100
90
80
70
60
50
40
30
20
10
f1 (ppm)

1u

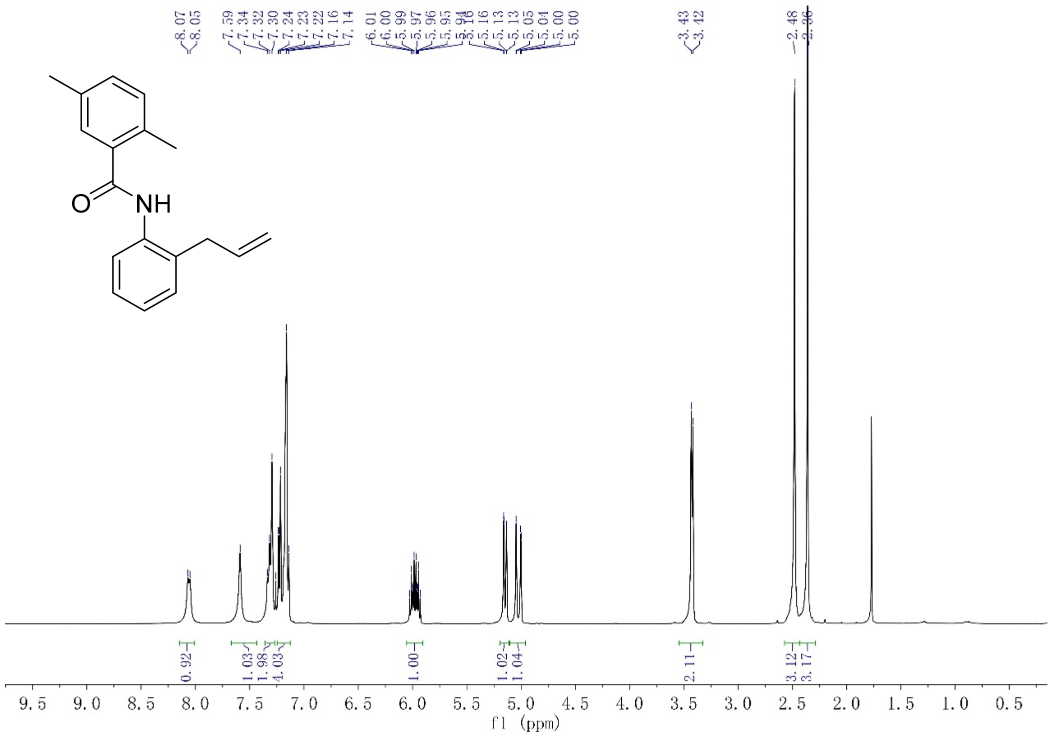

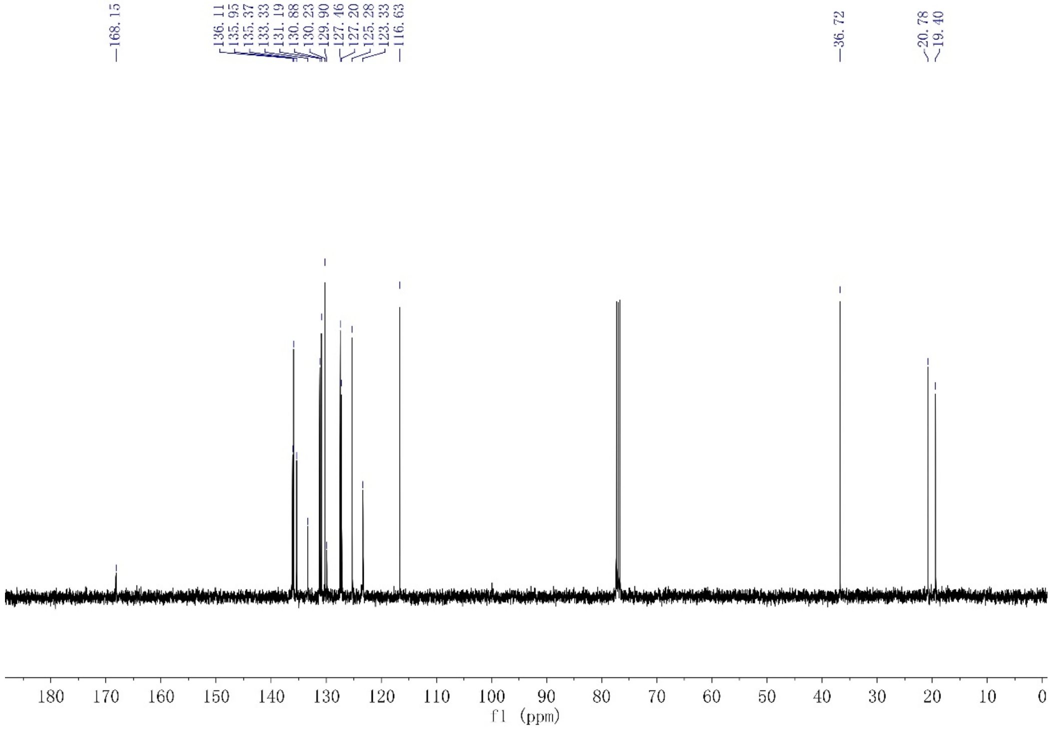

1k

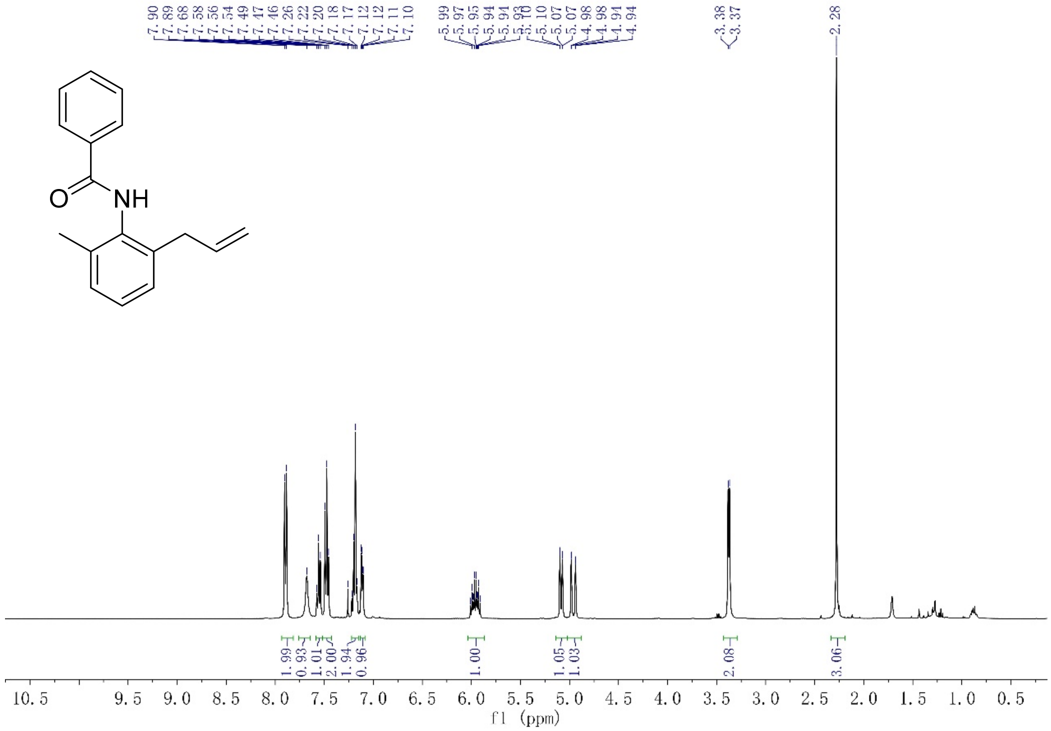

O
NH
7.90
7.89
7.68
7.58
7.56
7.54
7.49
7.47
7.46
7.26
7.22
7.20
7.18
7.17
7.12
7.12
7.11
7.10
5.99
5.97
5.95
5.94
5.91
5.93
5.10
5.10
5.07
5.07
4.98
4.91
4.94
3.38
3.37
2.28
1.99
0.93
1.01
2.00
1.94
0.96
1.00
1.05
1.03
2.08
3.06
10.5 9.5 9.0 8.5 8.0 7.5 7.0 6.5 6.0 5.5 5.0 4.5 4.0 3.5 3.0 2.5 2.0 1.5 1.0 0.5
f1 (ppm)

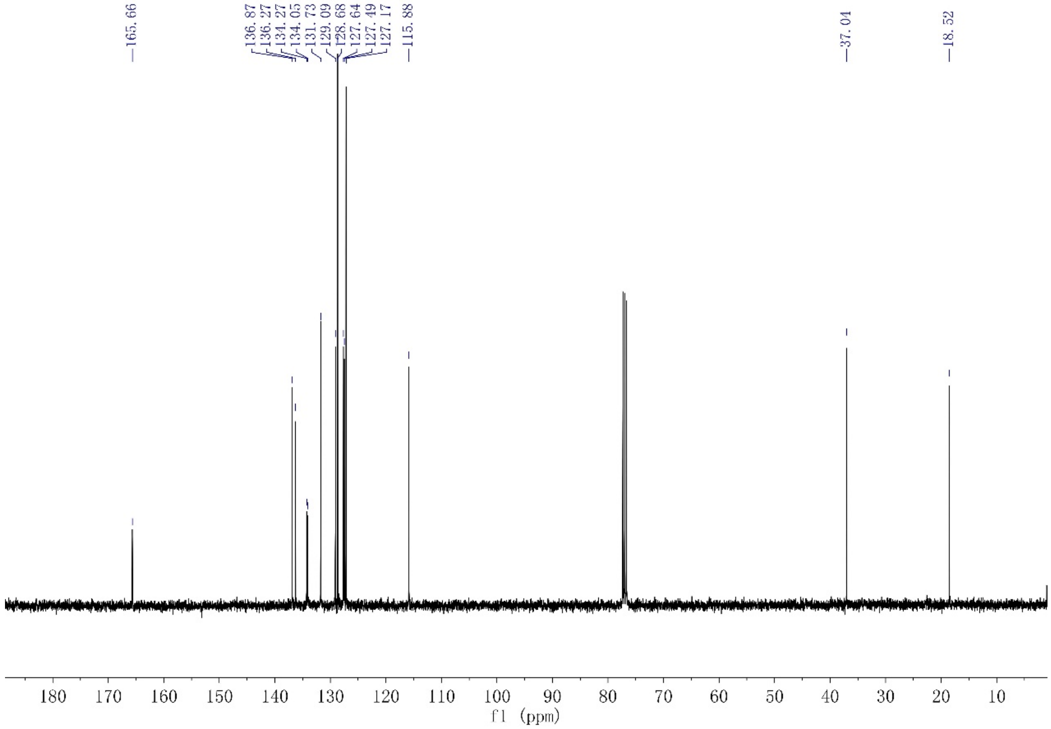

165.66
136.87
136.27
134.27
134.05
131.73
129.09
128.68
127.64
127.49
127.17
115.88
37.04
18.52
180 170 160 150 140 130 120 110 100 90 80 70 60 50 40 30 20 10
f1 (ppm)

1l

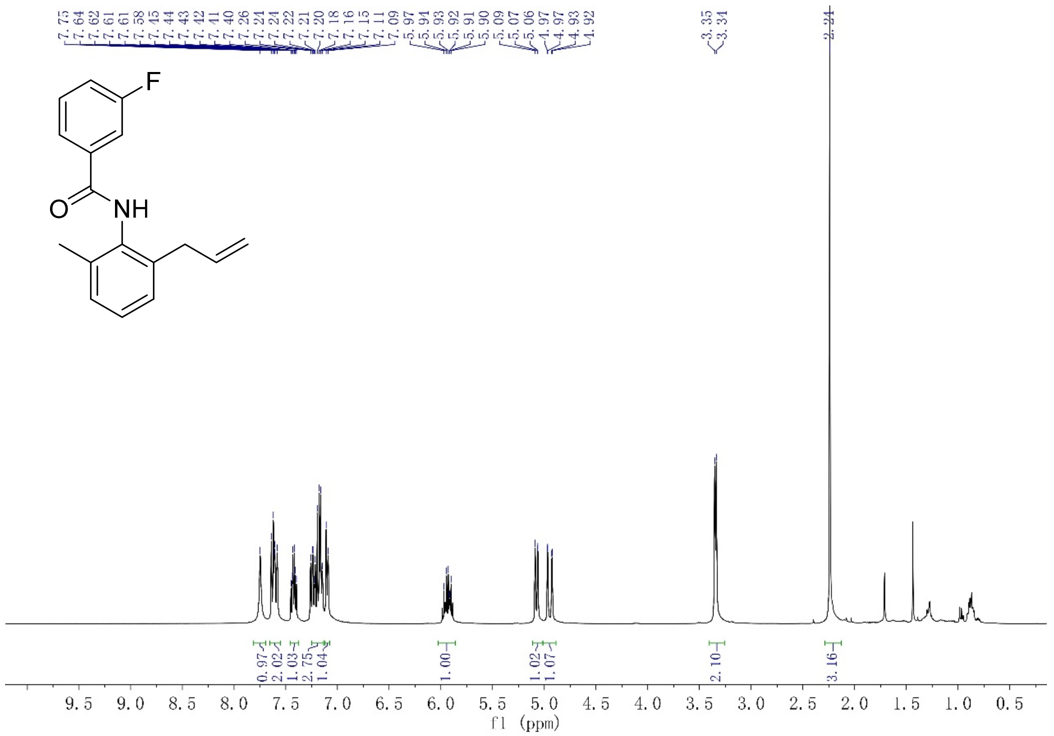

7.75 7.64 7.62 7.61 7.61 7.58 7.45 7.44 7.43 7.42 7.41 7.40 7.26 7.21 7.21 7.22 7.21 7.20 7.18 7.16 7.15 7.11 7.09 5.97 5.94 5.93 5.92 5.91 5.90 5.09 5.07 5.06 4.97 4.97 4.93 4.92
3.35 3.31
2.24
F
O
NH
0.97 2.02 1.03 2.75 1.04 1.00 1.02 1.07 2.10 3.16
f1 (ppm)

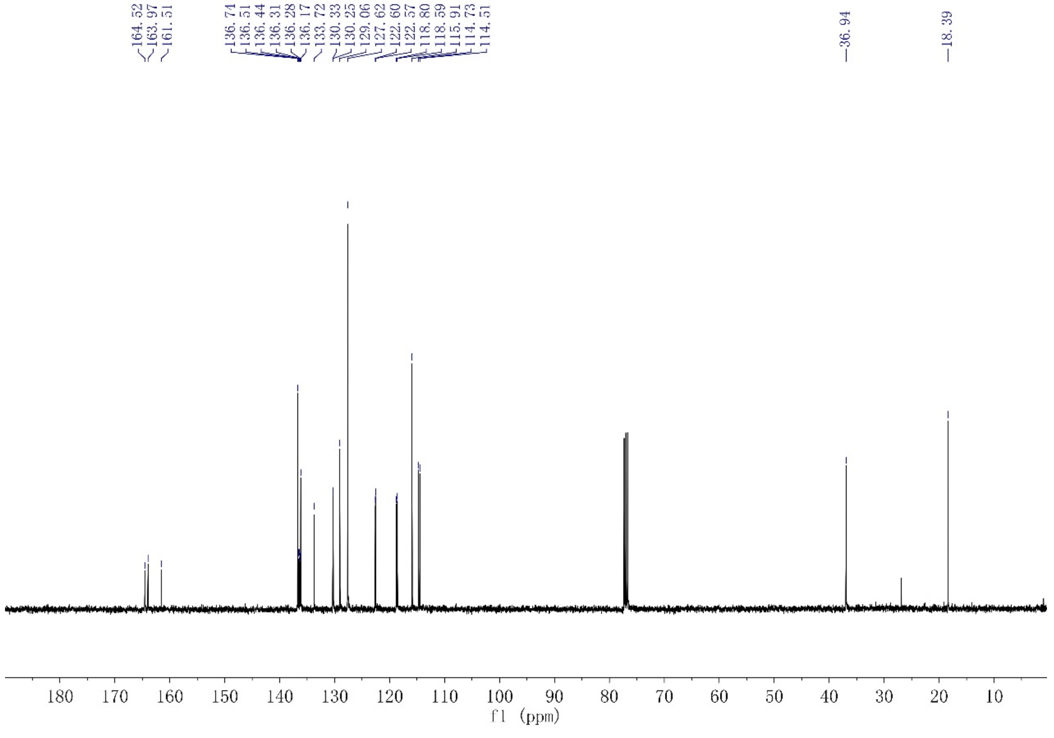

164.52 163.97 161.51
136.74 136.51 136.44 136.31 136.28 136.17 133.72 130.33 130.25 129.06 127.62 122.60 122.57 118.80 118.59 115.91 114.73 114.51
36.94
18.39
f1 (ppm)

1m

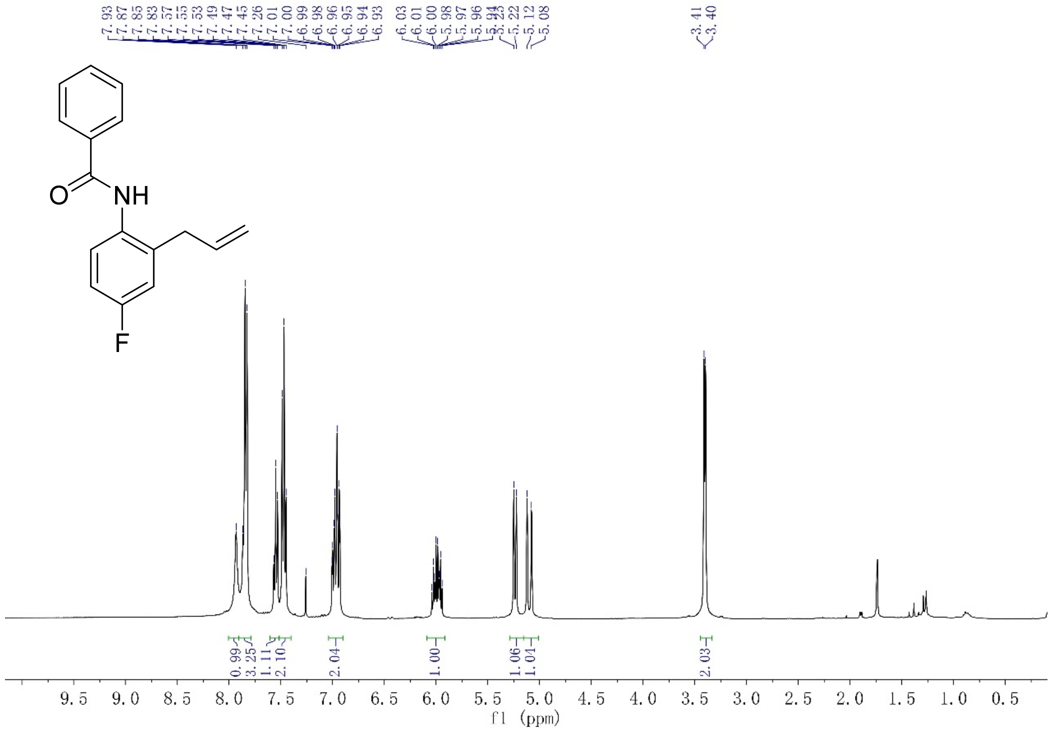

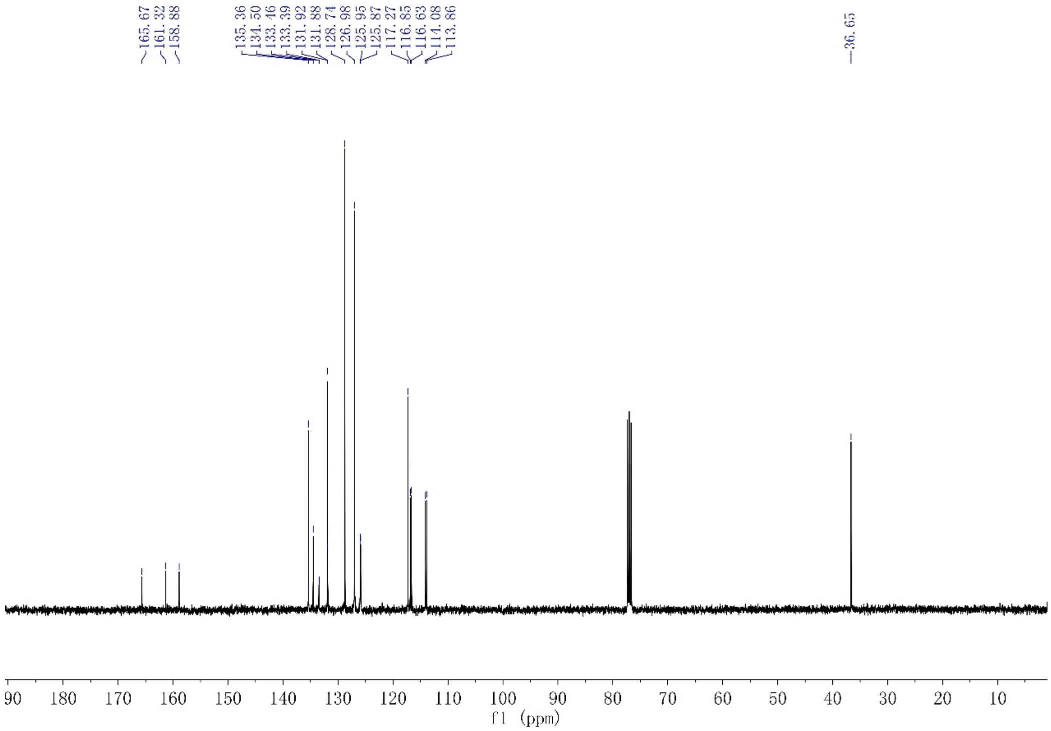

1n

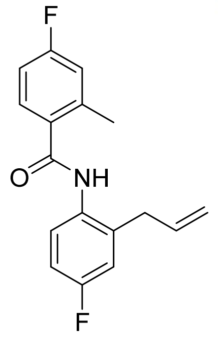

F
O
NH
F

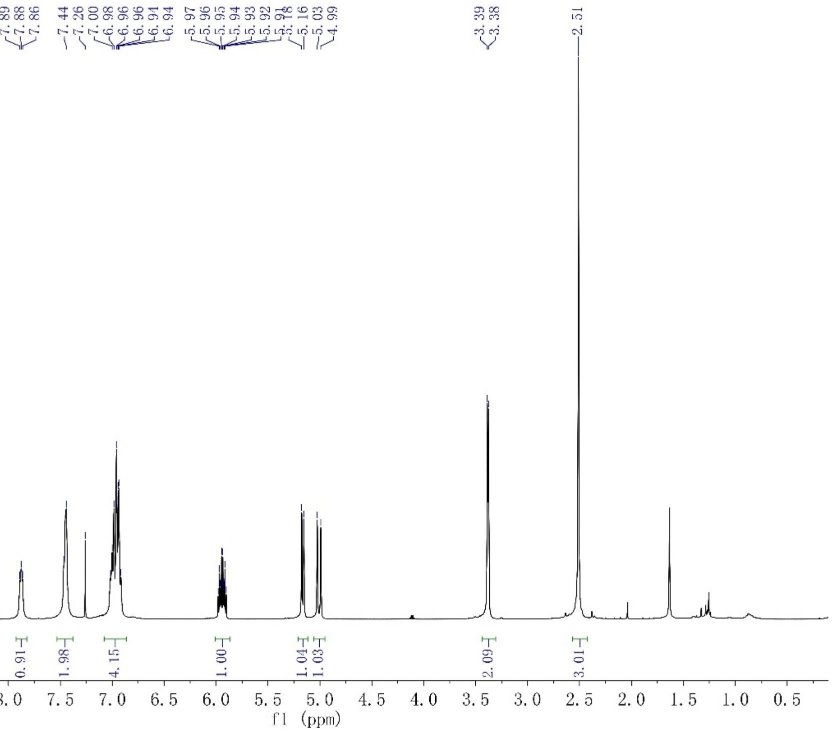

7.89
7.88
7.86
7.44
7.26
7.00
6.98
6.96
6.96
6.94
6.94
5.97
5.96
5.95
5.94
5.93
5.92
5.91
5.18
5.16
5.03
4.99
3.39
3.38
2.51
0.91
1.98
4.15
1.00
1.04
1.03
2.09
3.01
9.5 9.0 8.5 8.0 7.5 7.0 6.5 6.0 5.5 5.0 4.5 4.0 3.5 3.0 2.5 2.0 1.5 1.0 0.5
f1 (ppm)

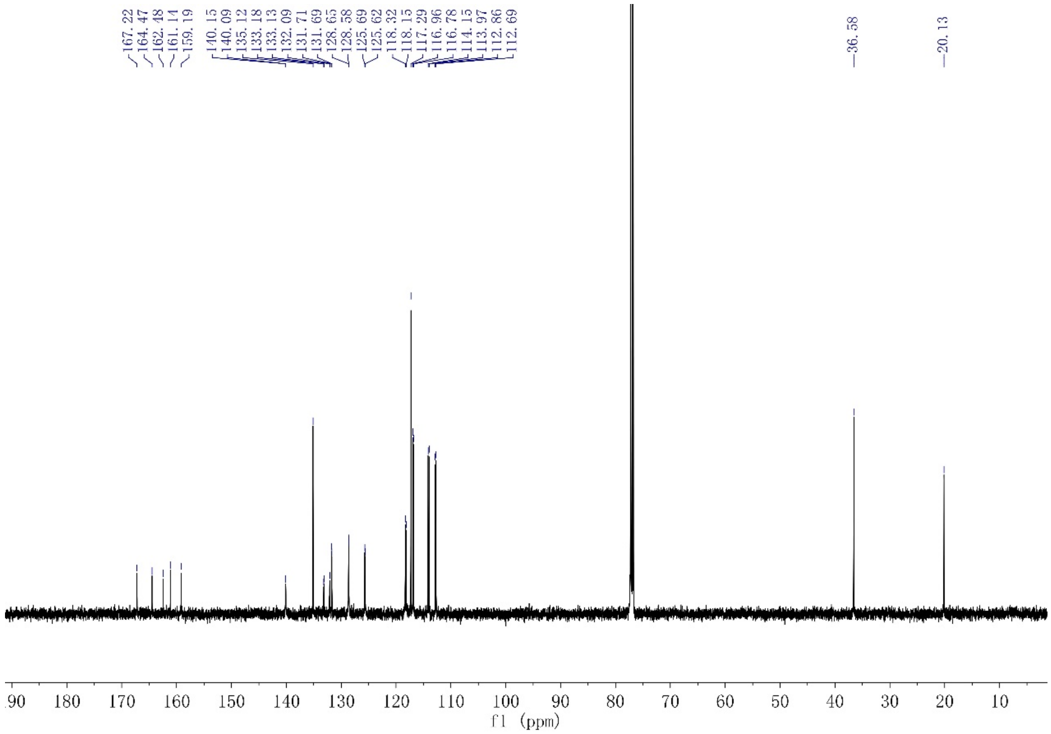

167.22
164.47
162.48
161.14
159.19
140.15
140.09
135.12
133.18
133.13
132.09
131.71
131.69
128.65
128.58
125.69
125.62
118.32
118.15
117.29
116.96
116.78
114.15
113.97
112.86
112.69
36.58
20.13
190 180 170 160 150 140 130 120 110 100 90 80 70 60 50 40 30 20 10
f1 (ppm)

1o

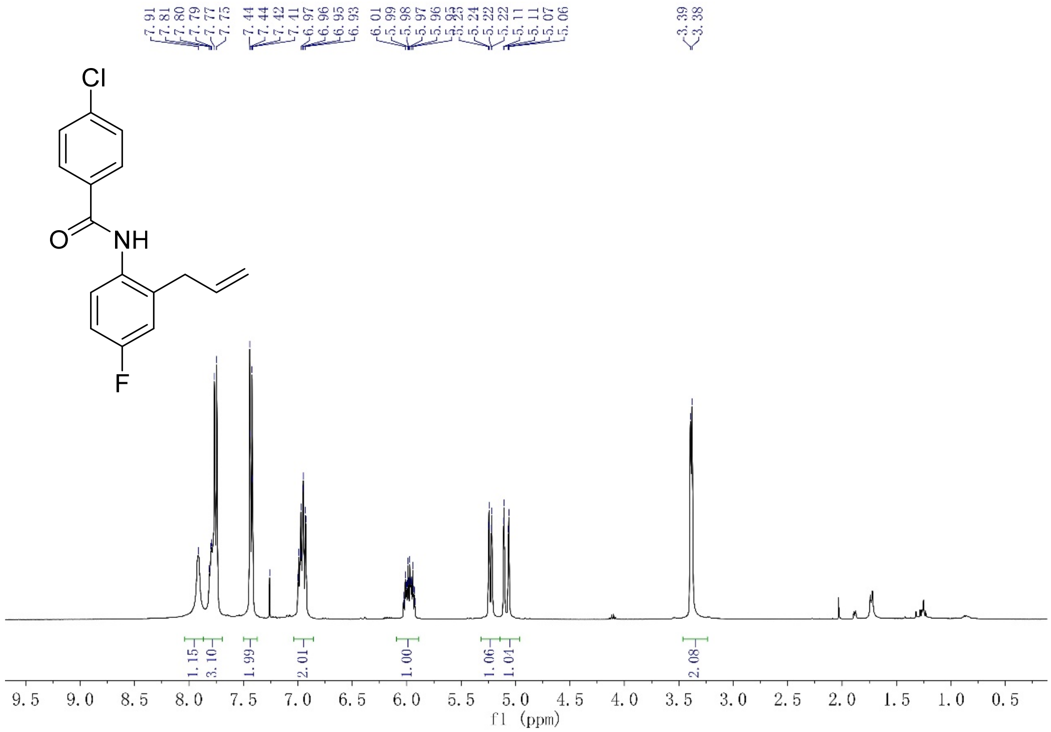

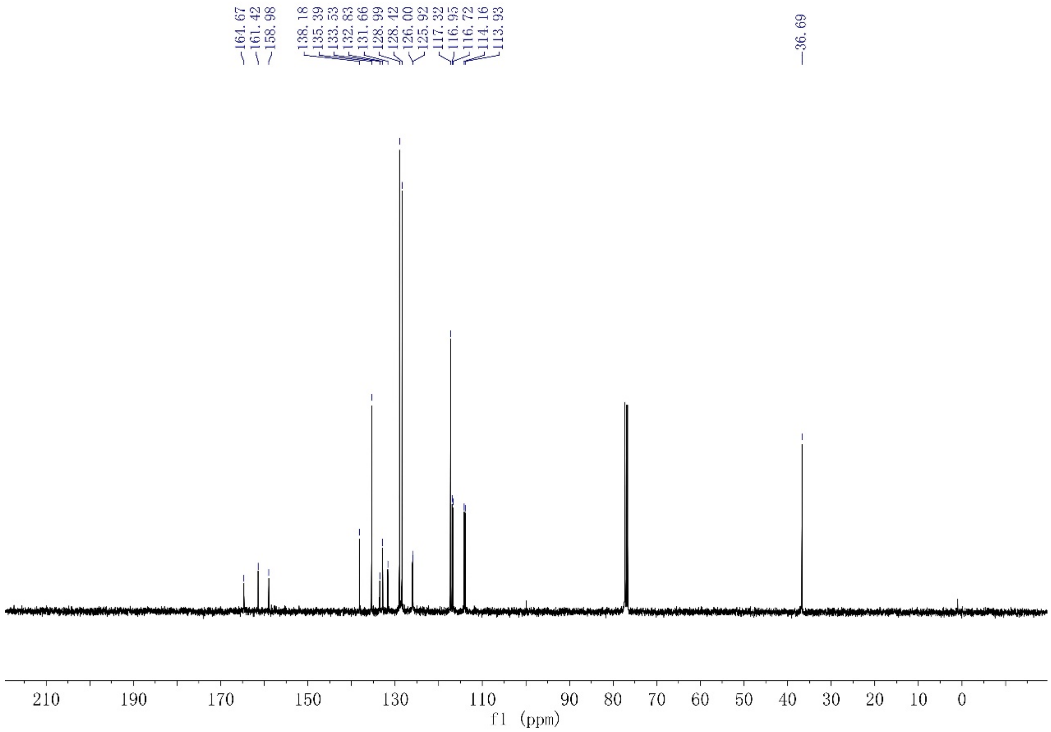

1p

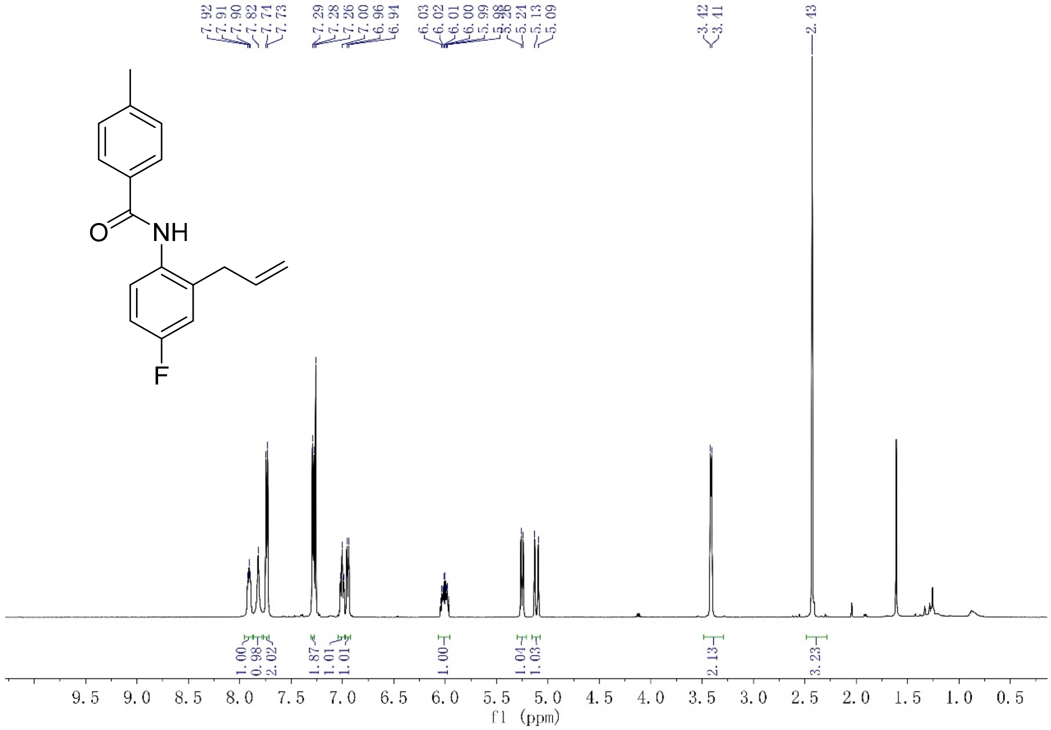

7.92
7.91
7.90
7.82
7.71
7.73
7.29
7.28
7.26
7.00
6.96
6.94
6.03
6.02
6.01
6.00
5.99
5.21
5.13
5.09
3.42
3.41
2.43
O
NH
F
1.00
0.98
2.02
1.87
1.01
1.01
1.00
1.04
1.03
2.13
3.23
9.5 9.0 8.5 8.0 7.5 7.0 6.5 6.0 5.5 5.0 4.5 4.0 3.5 3.0 2.5 2.0 1.5 1.0 0.5
f1 (ppm)

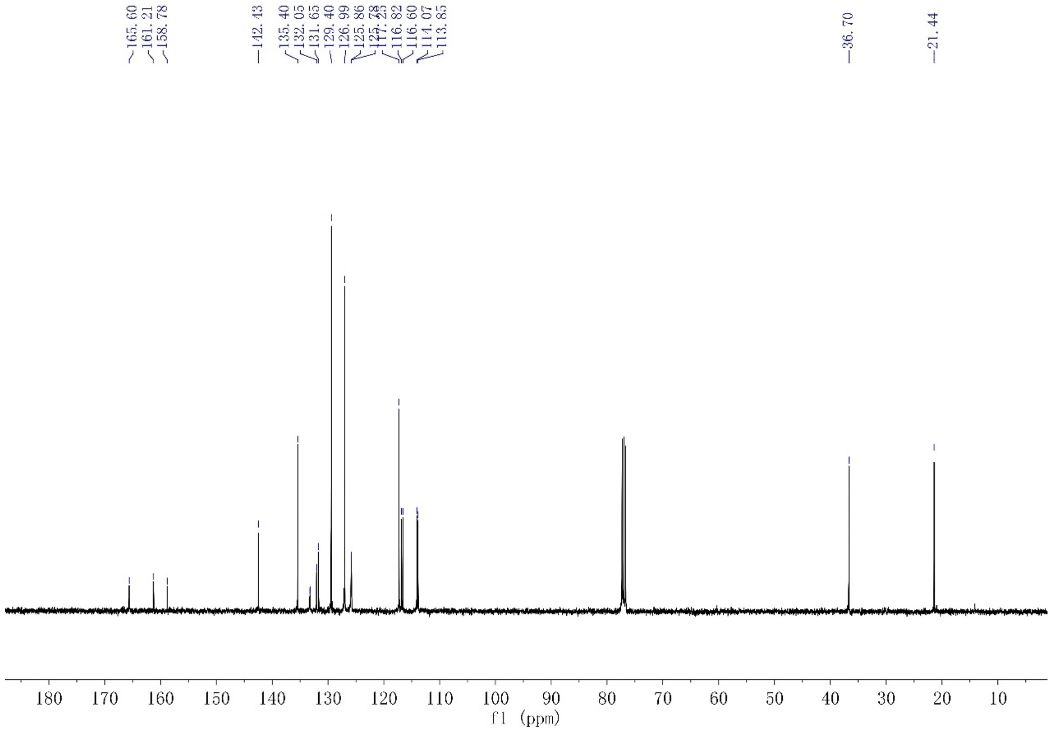

165.60
161.21
158.78
142.43
135.40
132.05
131.65
129.40
126.99
125.86
116.82
116.60
114.07
113.85
36.70
21.44
180 170 160 150 140 130 120 110 100 90 80 70 60 50 40 30 20 10
f1 (ppm)

1q

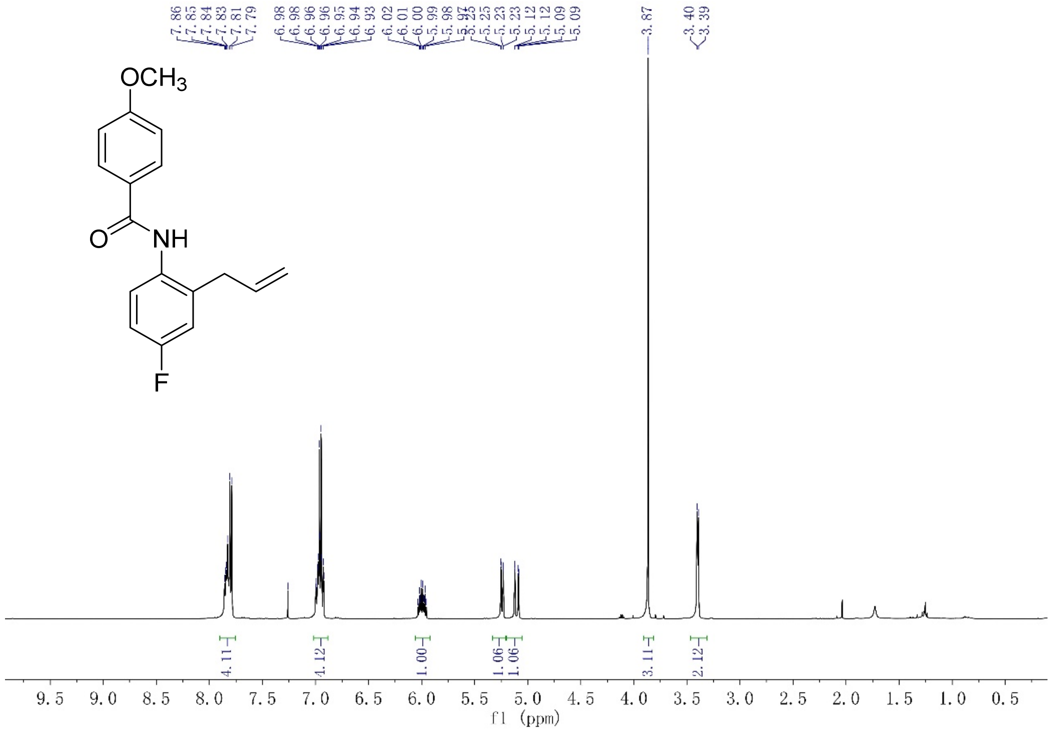

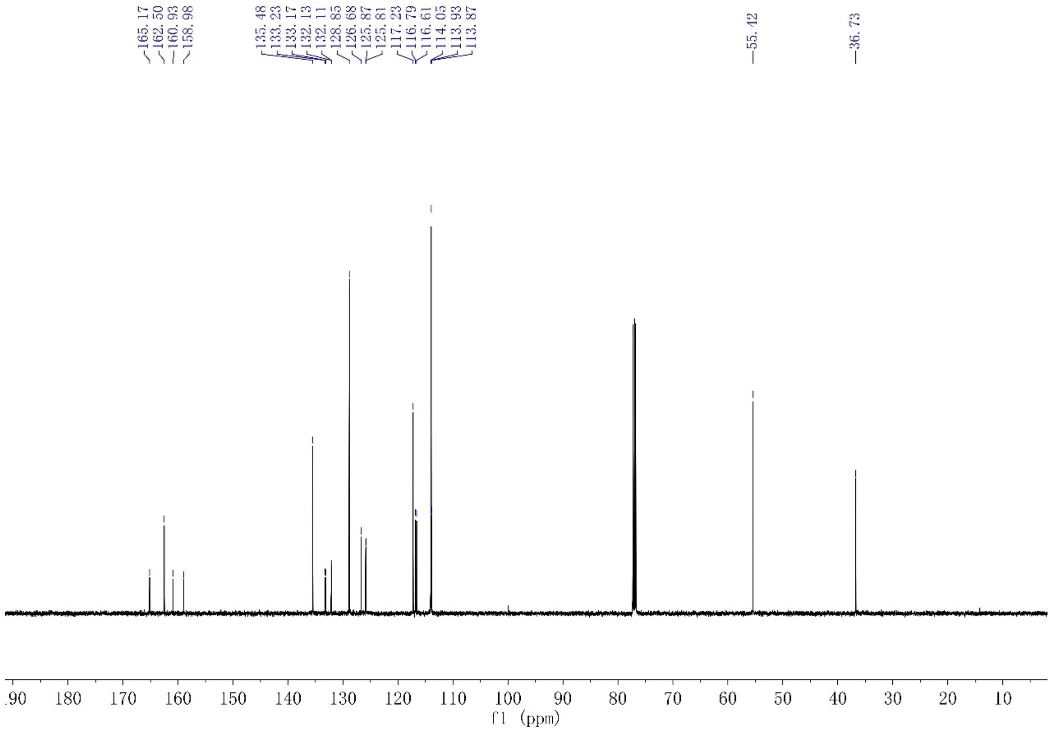

1r

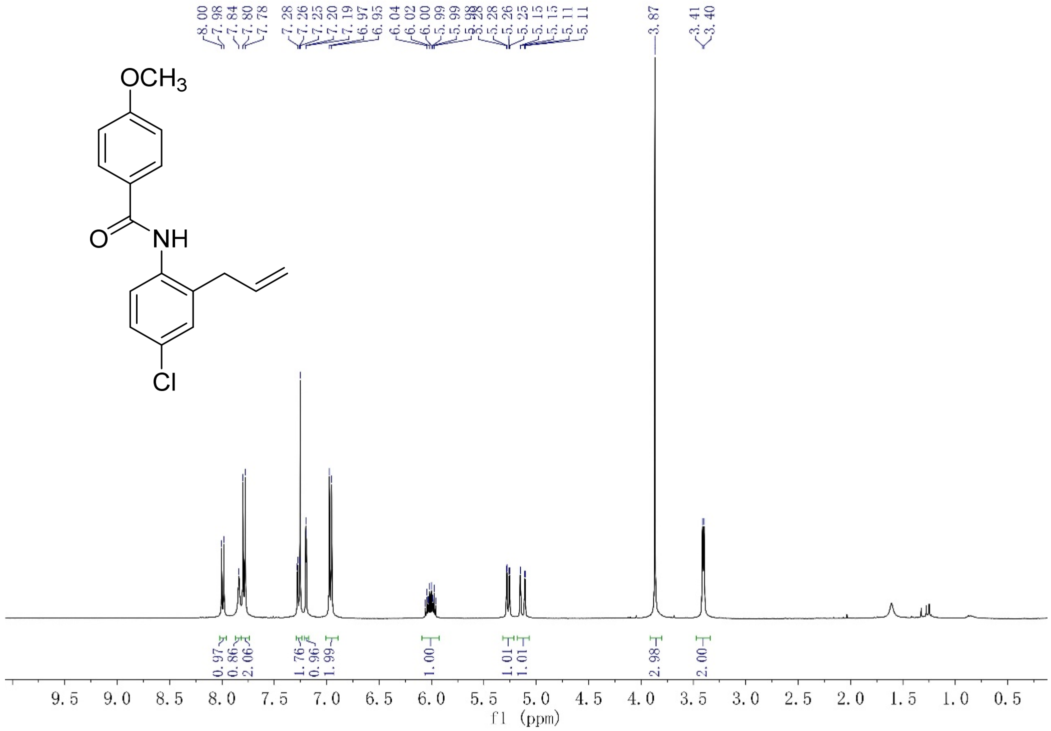
8.00
7.98
7.84
7.80
7.78
7.28
7.26
7.25
7.20
7.19
6.97
6.95
6.04
6.02
6.00
5.99
5.99
5.28
5.26
5.25
5.15
5.11
5.11
3.87
3.41
3.40
OCH3
O
NH
Cl
0.97
0.86
2.06
1.76
0.96
1.99
1.00
1.01
1.01
2.98
2.00
9.5 9.0 8.5 8.0 7.5 7.0 6.5 6.0 5.5 5.0 4.5 4.0 3.5 3.0 2.5 2.0 1.5 1.0 0.5
f1 (ppm)

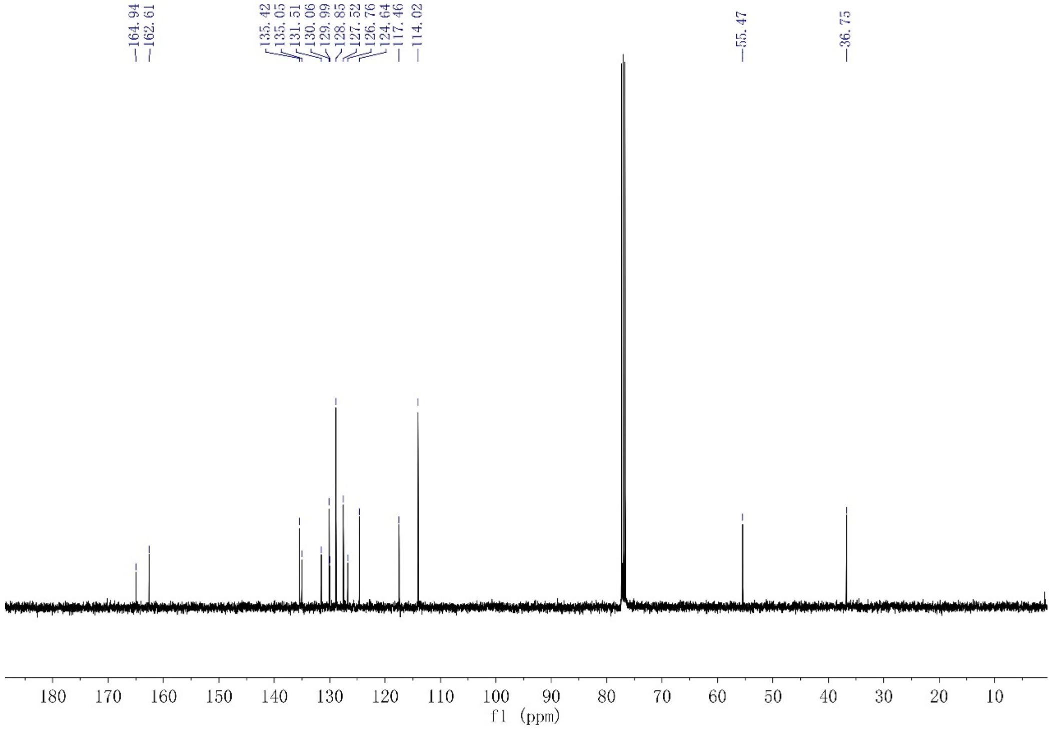
164.94
162.61
135.42
135.05
131.51
130.06
129.99
128.85
127.52
126.76
124.64
117.46
114.02
55.47
36.75
180 170 160 150 140 130 120 110 100 90 80 70 60 50 40 30 20 10
f1 (ppm)

1s

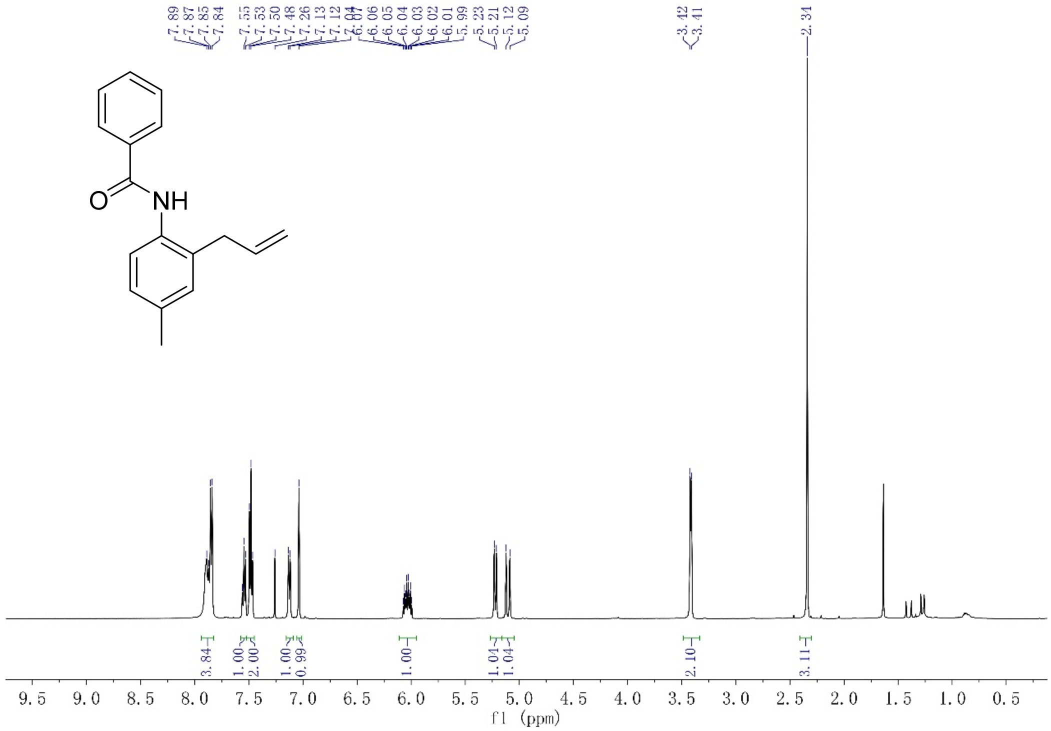
7.89
7.87
7.85
7.84
7.55
7.53
7.50
7.48
7.26
7.13
7.12
7.04
6.07
6.06
6.05
6.04
6.03
6.02
6.01
5.99
5.23
5.21
5.12
5.09
3.42
3.41
2.31
O
NH
3.84
1.00
2.00
1.00
0.99
1.00
1.04
1.04
2.10
3.11
f1 (ppm)

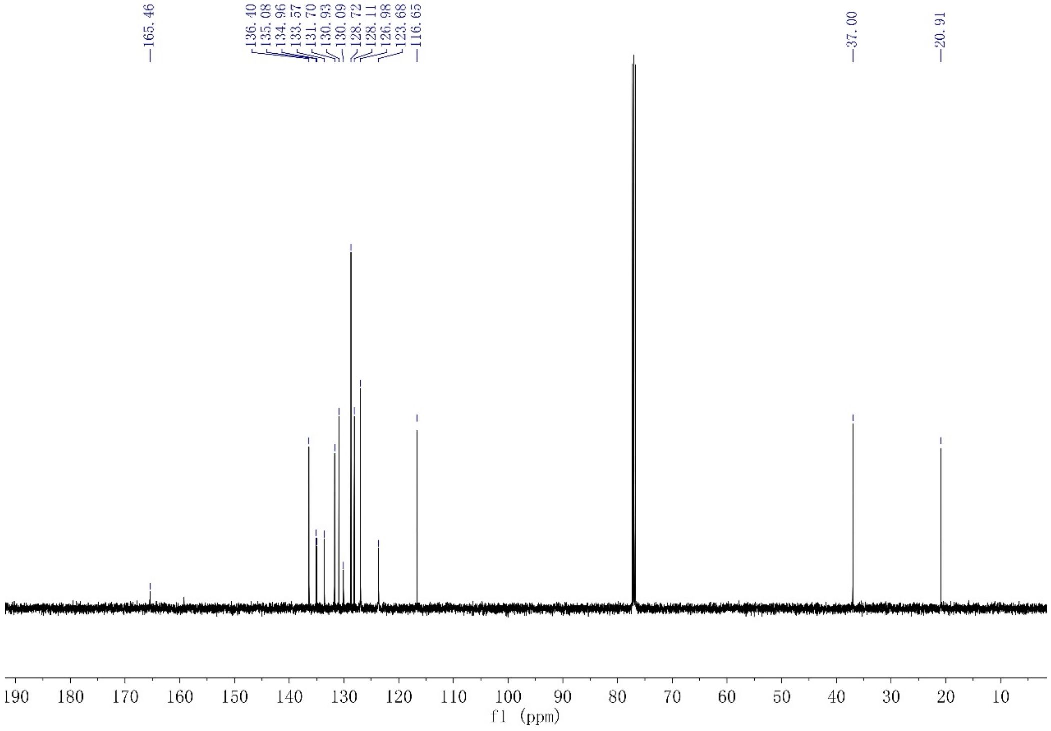
165.46
136.40
135.08
134.96
133.57
131.70
130.93
130.09
128.72
128.11
126.98
123.68
116.65
37.00
20.91
f1 (ppm)

1t

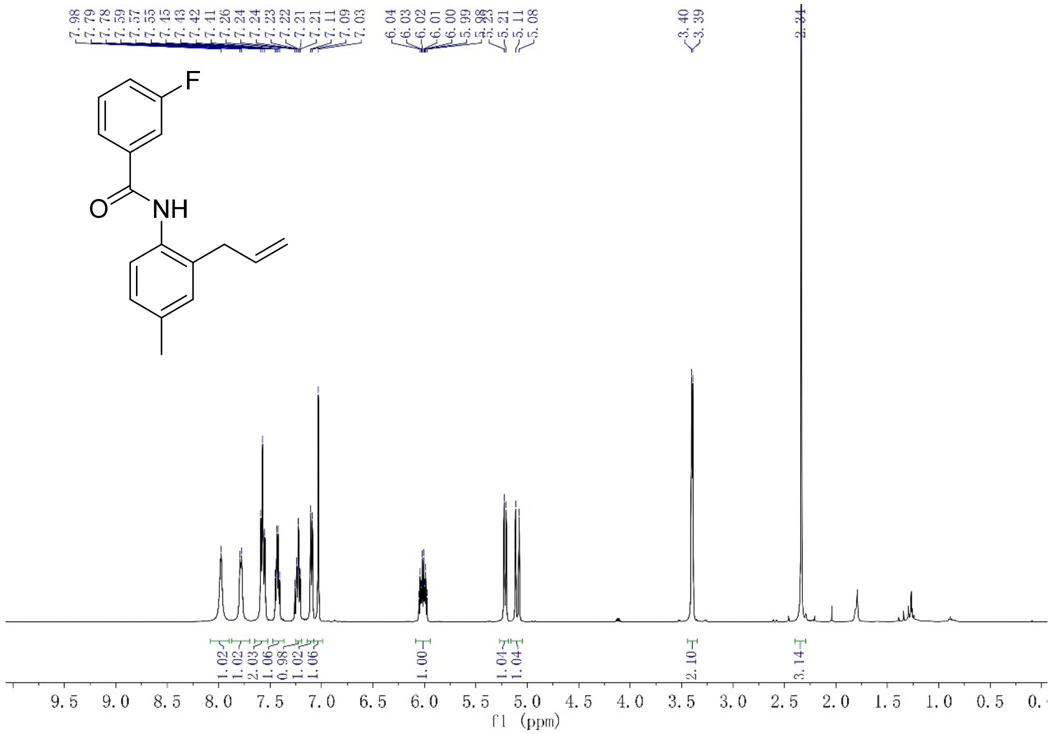

7.98
7.79
7.78
7.59
7.57
7.55
7.45
7.43
7.42
7.41
7.26
7.24
7.24
7.23
7.22
7.21
7.21
7.11
7.09
7.03
6.04
6.03
6.02
6.01
6.00
5.99
5.21
5.11
5.08
3.40
3.39
F
O
NH
1.02
1.02
2.03
1.06
0.98
1.02
1.06
1.00
1.04
1.04
2.10
3.14
9.5 9.0 8.5 8.0 7.5 7.0 6.5 6.0 5.5 5.0 4.5 4.0 3.5 3.0 2.5 2.0 1.5 1.0 0.5
f1 (ppm)

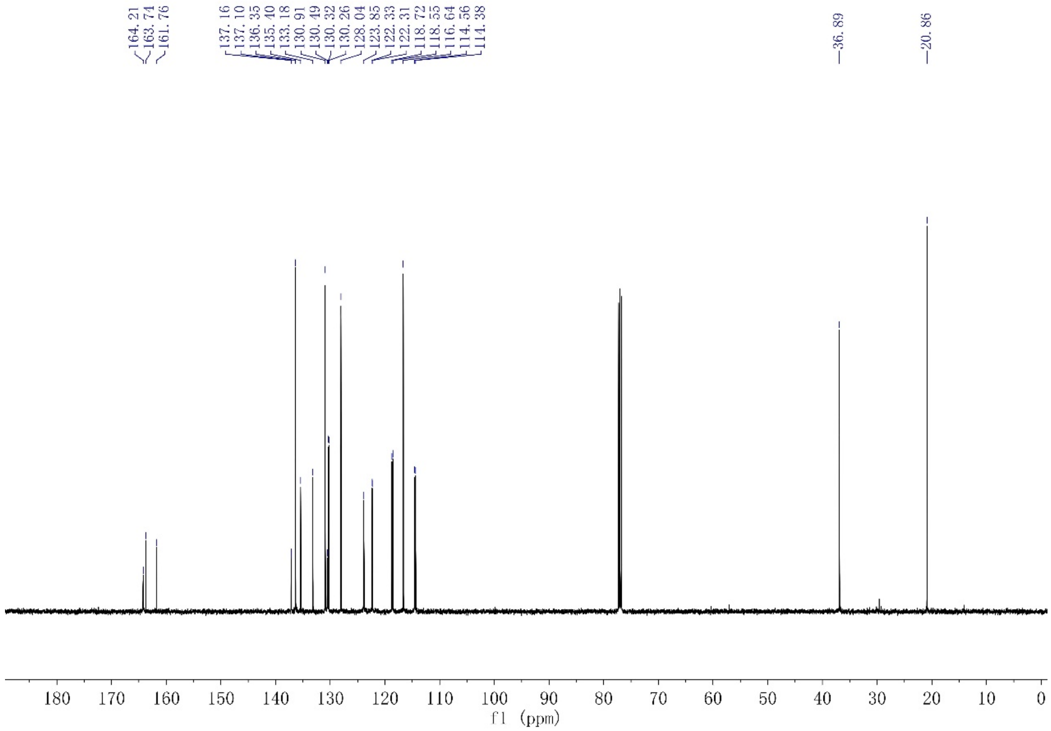

164.21
163.74
161.76
137.16
137.10
136.35
135.40
133.18
130.91
130.49
130.32
130.26
128.04
123.85
122.33
122.31
118.72
118.55
116.64
114.56
114.38
36.89
20.86
180 170 160 150 140 130 120 110 100 90 80 70 60 50 40 30 20 10 0
f1 (ppm)

1v

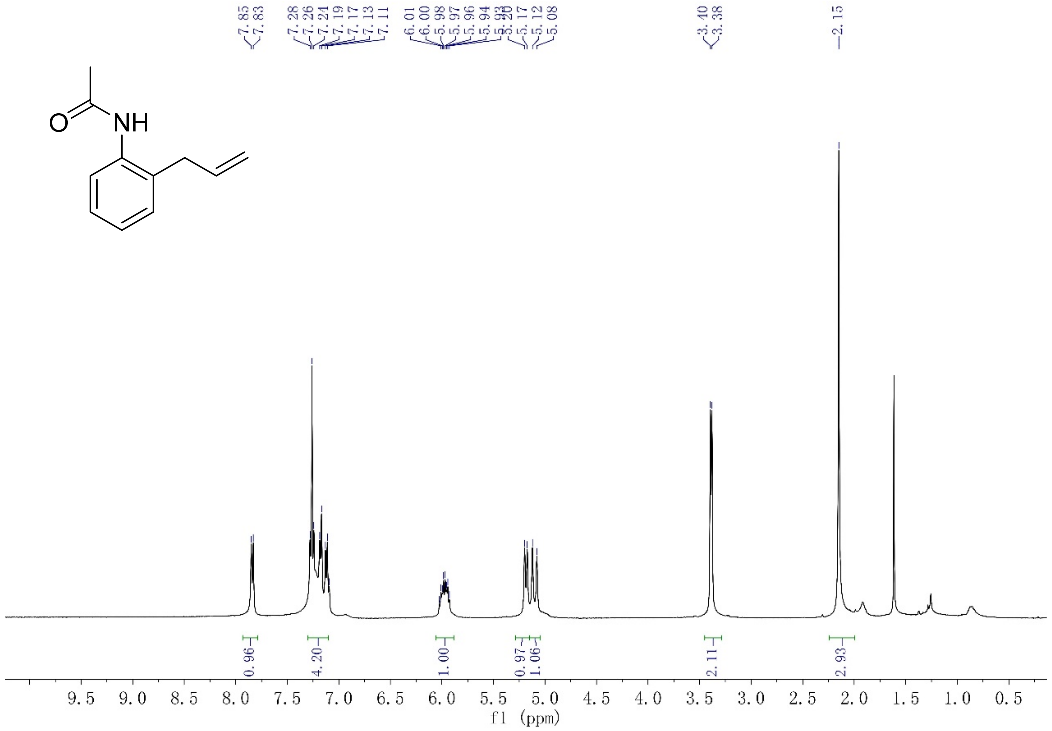
O
NH
7.85
7.83
7.28
7.26
7.21
7.19
7.17
7.13
7.11
6.01
6.00
5.98
5.97
5.96
5.94
3.40
3.38
2.15
0.96
4.20
1.00
0.97
1.06
2.11
2.93
9.5 9.0 8.5 8.0 7.5 7.0 6.5 6.0 5.5 5.0 4.5 4.0 3.5 3.0 2.5 2.0 1.5 1.0 0.5
f1 (ppm)

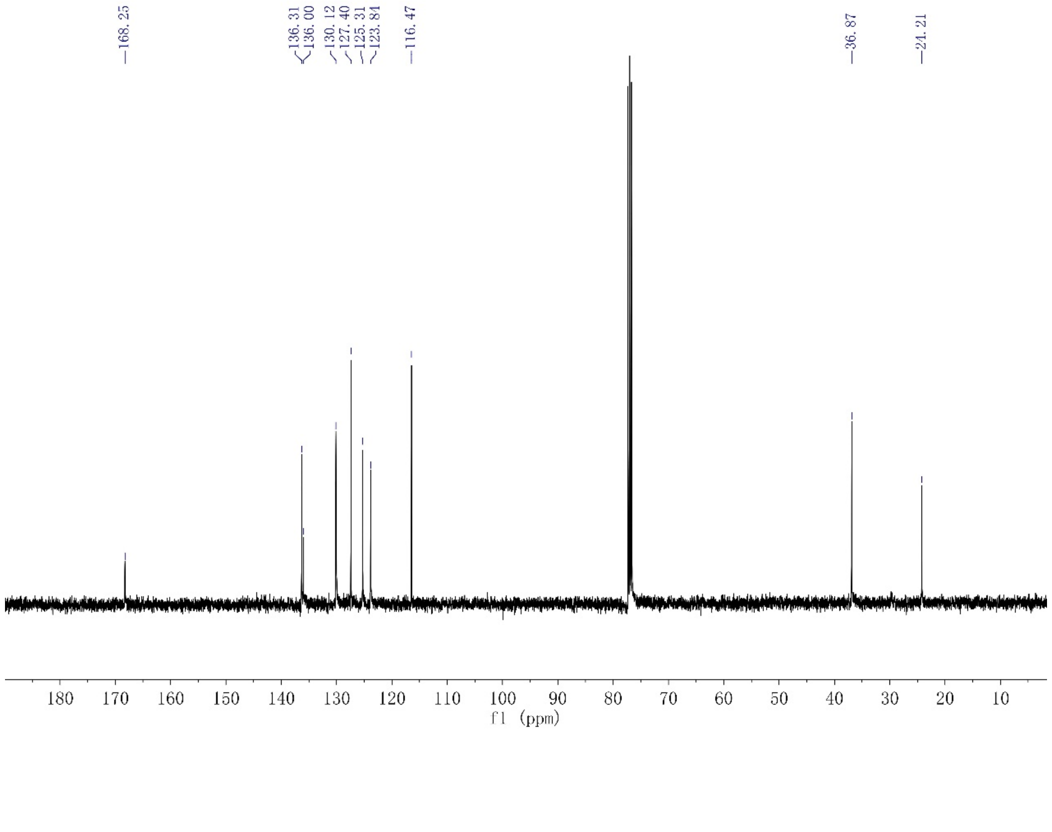
168.25
136.31
136.00
130.12
127.40
125.31
123.81
116.47
36.87
21.21
180 170 160 150 140 130 120 110 100 90 80 70 60 50 40 30 20 10
f1 (ppm)

1w

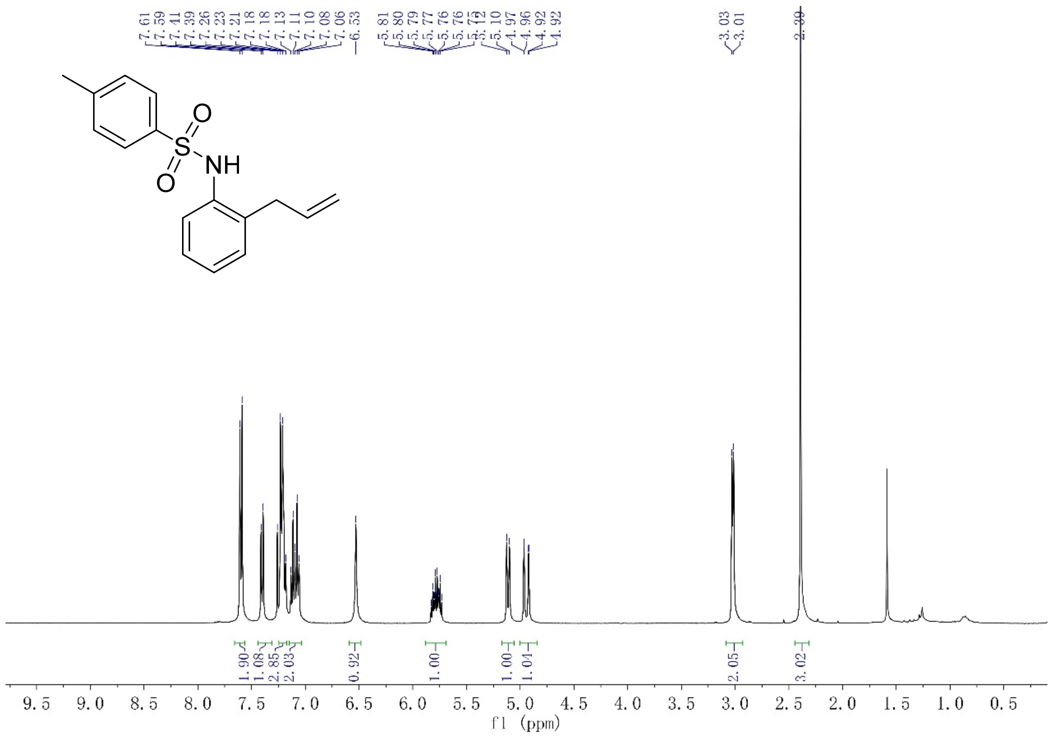
7.61
7.59
7.41
7.39
7.26
7.23
7.21
7.18
7.18
7.13
7.11
7.10
7.08
7.06
6.53
5.81
5.80
5.79
5.77
5.76
5.76
5.10
4.97
4.96
4.92
4.92
3.03
3.01
2.39
O
S
NH
O
1.90
1.08
2.85
2.03
0.92
1.00
1.00
1.04
2.05
3.02
9.5 9.0 8.5 8.0 7.5 7.0 6.5 6.0 5.5 5.0 4.5 4.0 3.5 3.0 2.5 2.0 1.5 1.0 0.5
f1 (ppm)

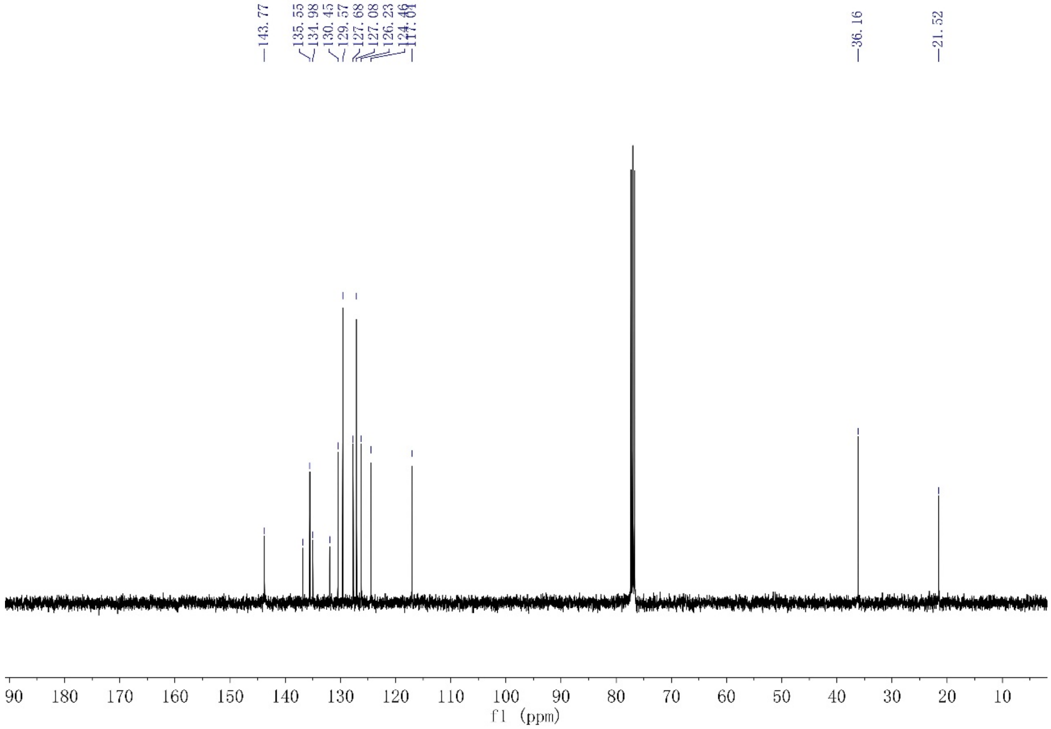
143.77
135.55
131.98
130.45
129.57
127.68
127.08
126.23
36.16
21.52
90 180 170 160 150 140 130 120 110 100 90 80 70 60 50 40 30 20 10
f1 (ppm)

4

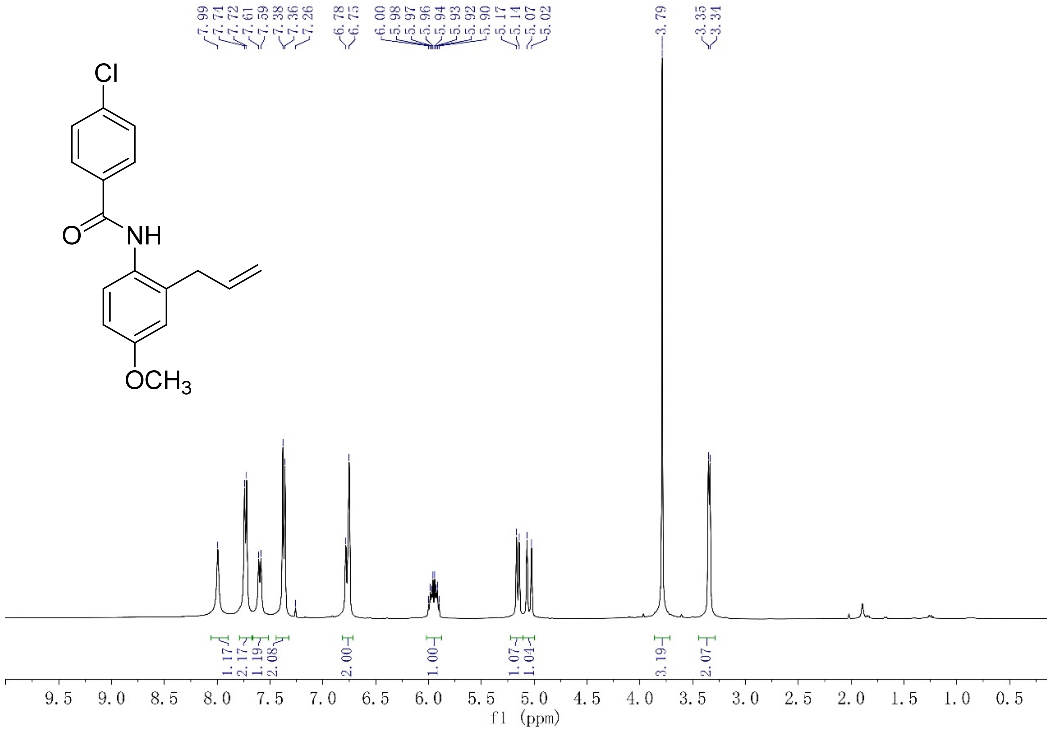
7.99
7.71
7.72
7.61
7.59
7.38
7.36
7.26
6.78
6.75
6.00
5.98
5.97
5.96
5.94
5.93
5.92
5.90
5.17
5.14
5.07
5.02
3.79
3.35
3.31
1.17
2.17
1.19
2.08
2.00
1.00
1.07
1.04
3.19
2.07
9.5 9.0 8.5 8.0 7.5 7.0 6.5 6.0 5.5 5.0 4.5 4.0 3.5 3.0 2.5 2.0 1.5 1.0 0.5
f1 (ppm)

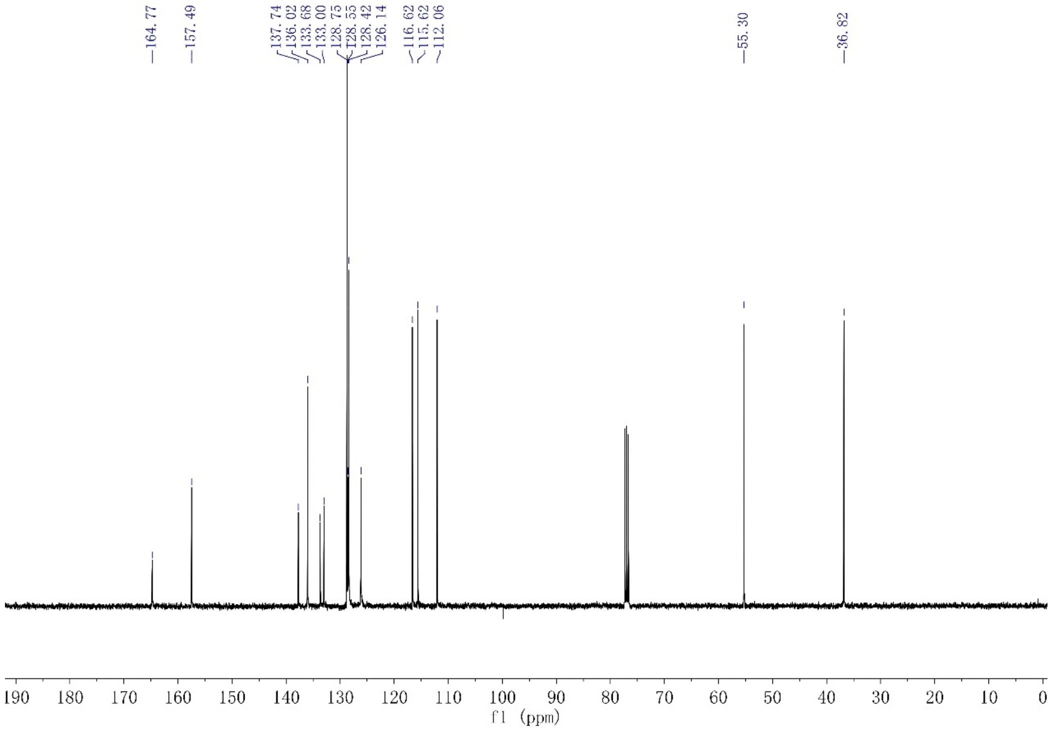
164.77
157.49
137.74
136.02
133.68
133.00
128.75
128.55
128.42
126.14
116.62
115.62
112.06
55.30
36.82
190 180 170 160 150 140 130 120 110 100 90 80 70 60 50 40 30 20 10 0
f1 (ppm)

2a

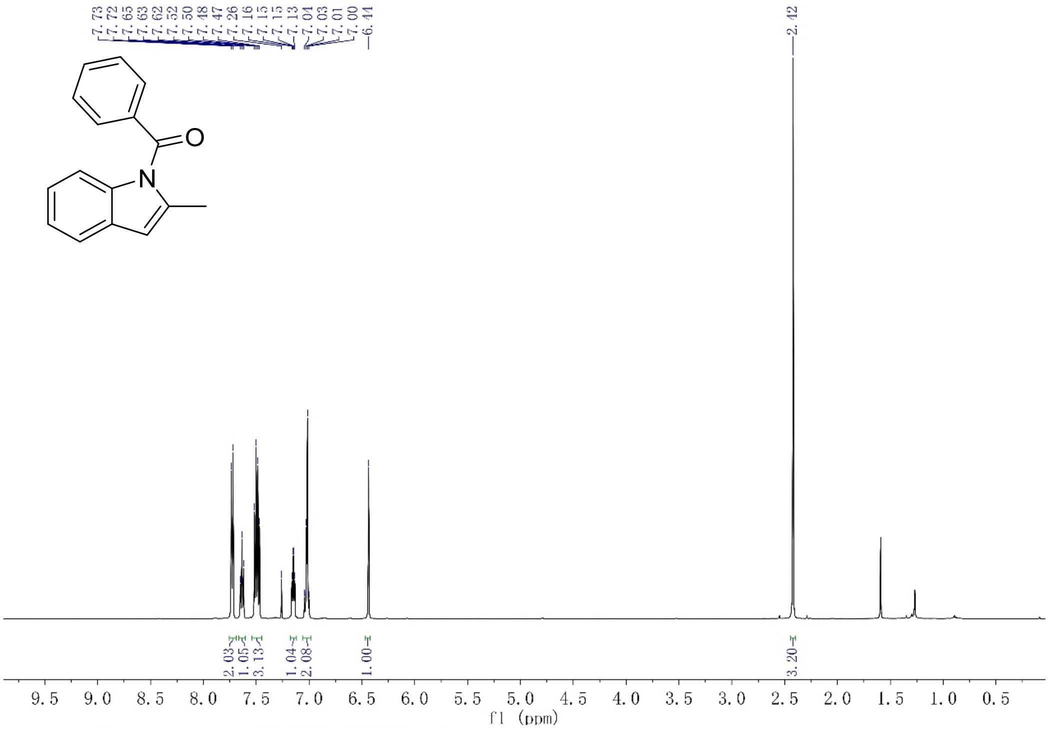
7.73
7.72
7.65
7.63
7.62
7.52
7.50
7.48
7.47
7.26
7.16
7.15
7.15
7.13
7.13
7.04
7.03
7.01
7.00
6.41
2.42
O
N
2.03
1.05
3.13
1.04
2.08
1.00
3.20
9.5 9.0 8.5 8.0 7.5 7.0 6.5 6.0 5.5 5.0 4.5 4.0 3.5 3.0 2.5 2.0 1.5 1.0 0.5
f1 (ppm)

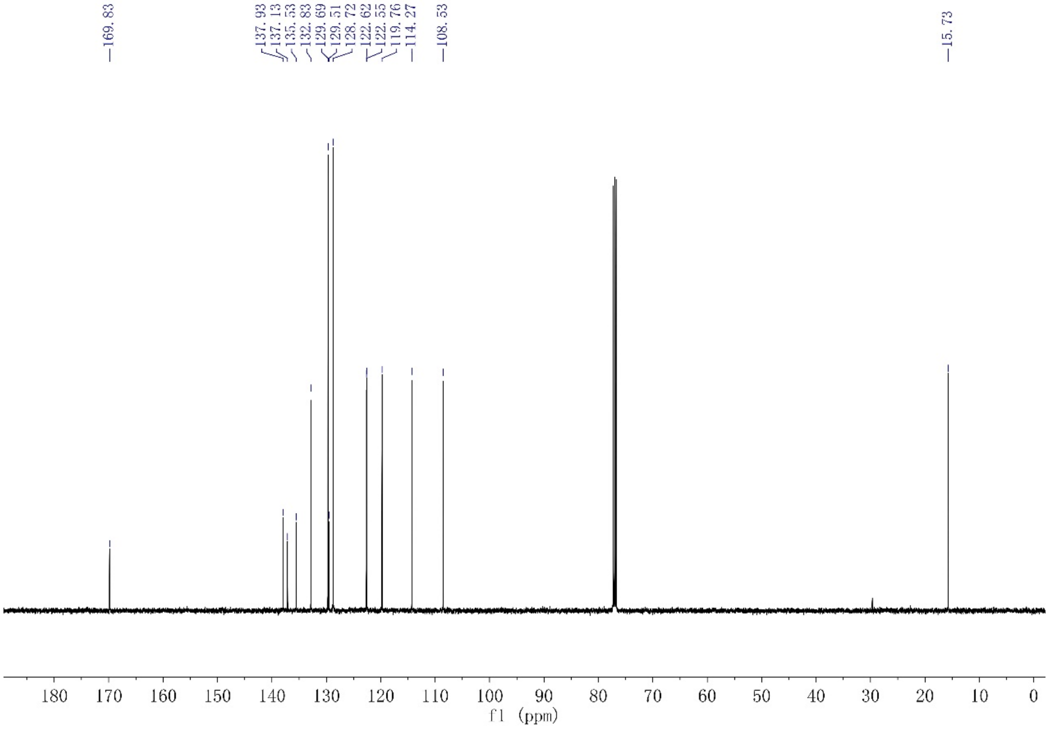
169.83
137.93
137.13
135.53
132.83
129.69
129.51
128.72
122.62
122.55
119.76
114.27
108.53
15.73
180 170 160 150 140 130 120 110 100 90 80 70 60 50 40 30 20 10 0
f1 (ppm)

2b

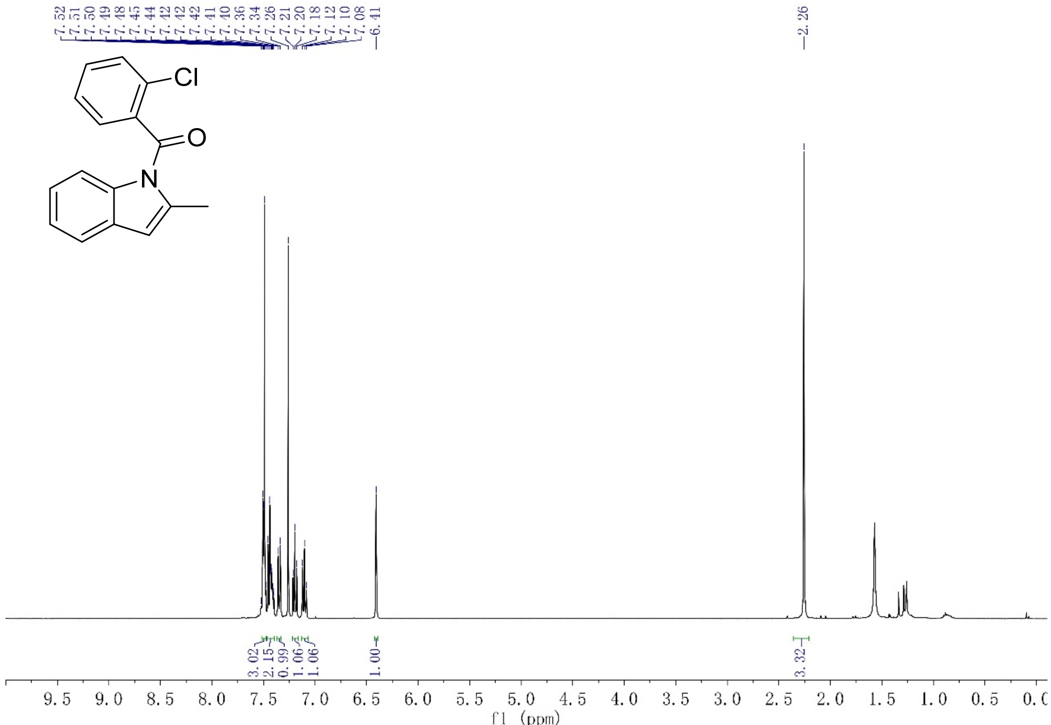

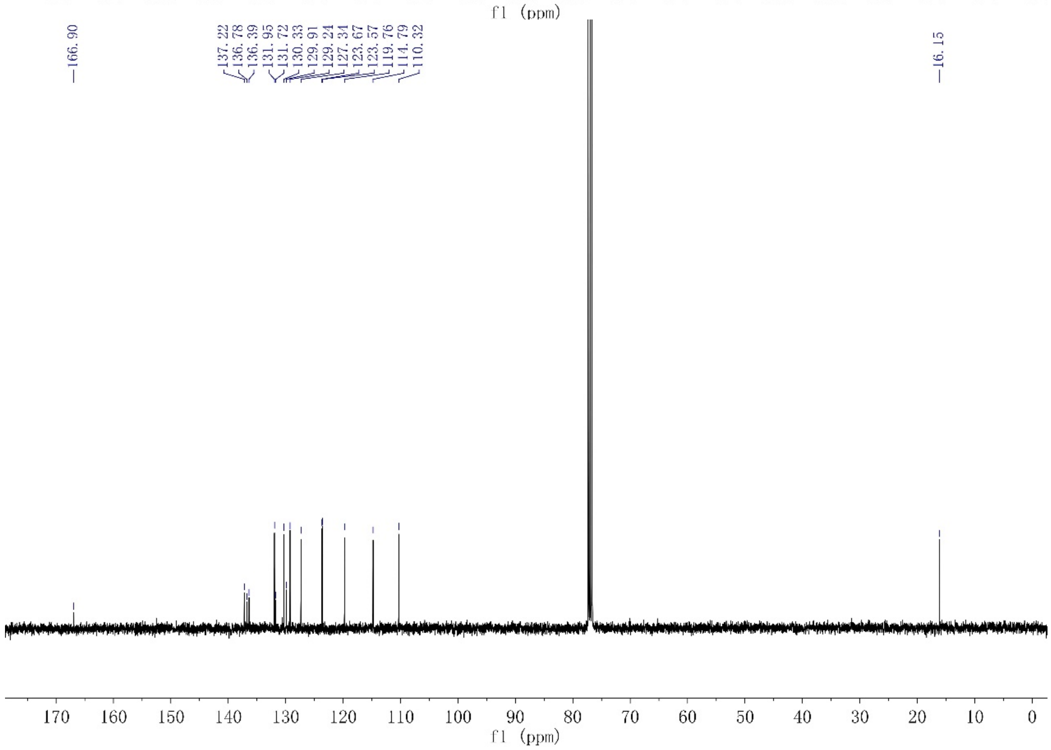

2c

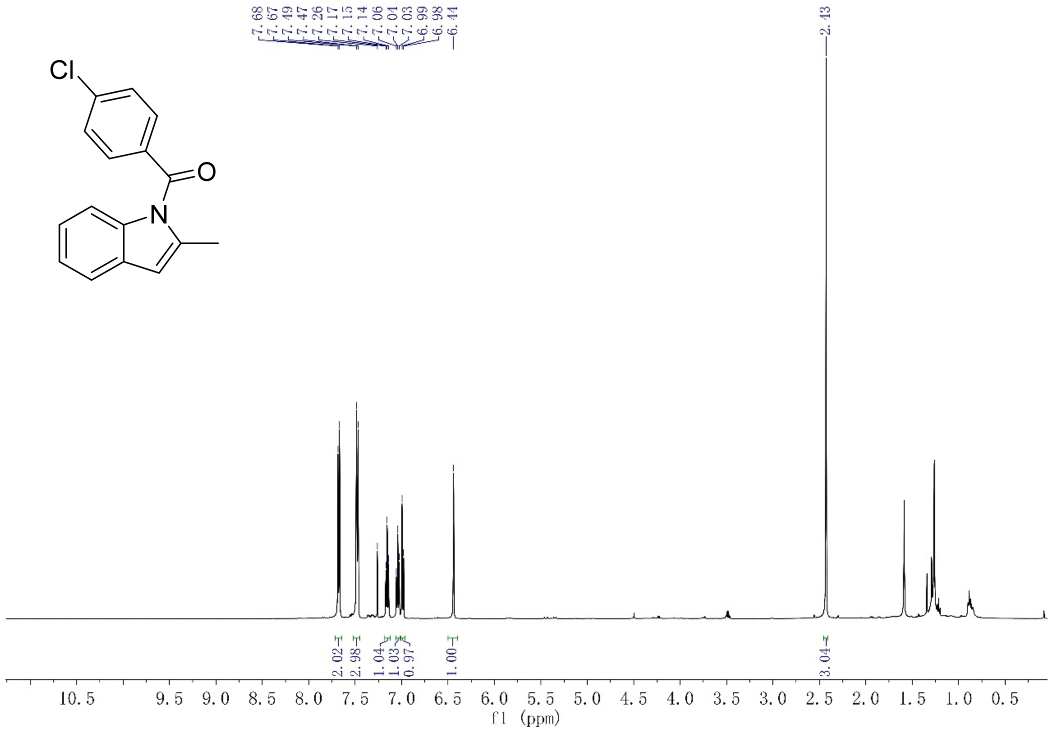
Cl
O
N
7.68
7.67
7.49
7.47
7.26
7.17
7.15
7.14
7.06
7.03
7.01
6.99
6.98
6.41
2.43
2.02
2.98
1.04
1.03
0.97
1.00
3.04
10.5 9.5 9.0 8.5 8.0 7.5 7.0 6.5 6.0 5.5 5.0 4.5 4.0 3.5 3.0 2.5 2.0 1.5 1.0 0.5
f1 (ppm)

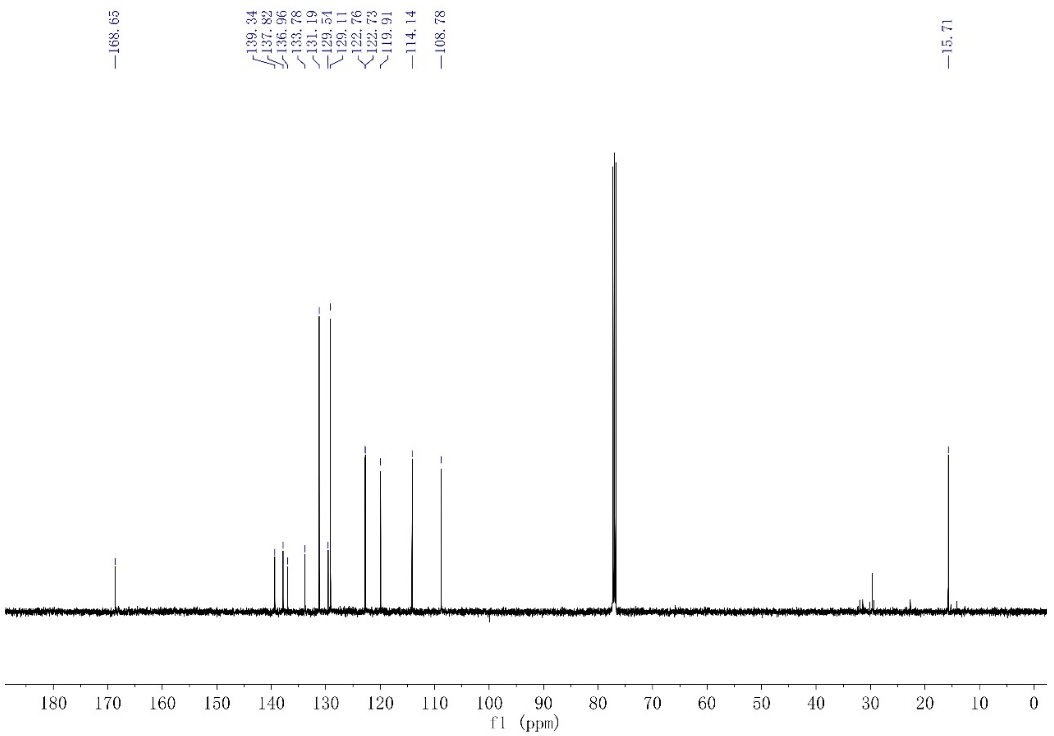
168.65
139.34
137.82
136.96
133.78
131.19
129.51
129.11
122.76
122.73
119.91
114.14
108.78
15.71
180 170 160 150 140 130 120 110 100 90 80 70 60 50 40 30 20 10 0
f1 (ppm)

2d

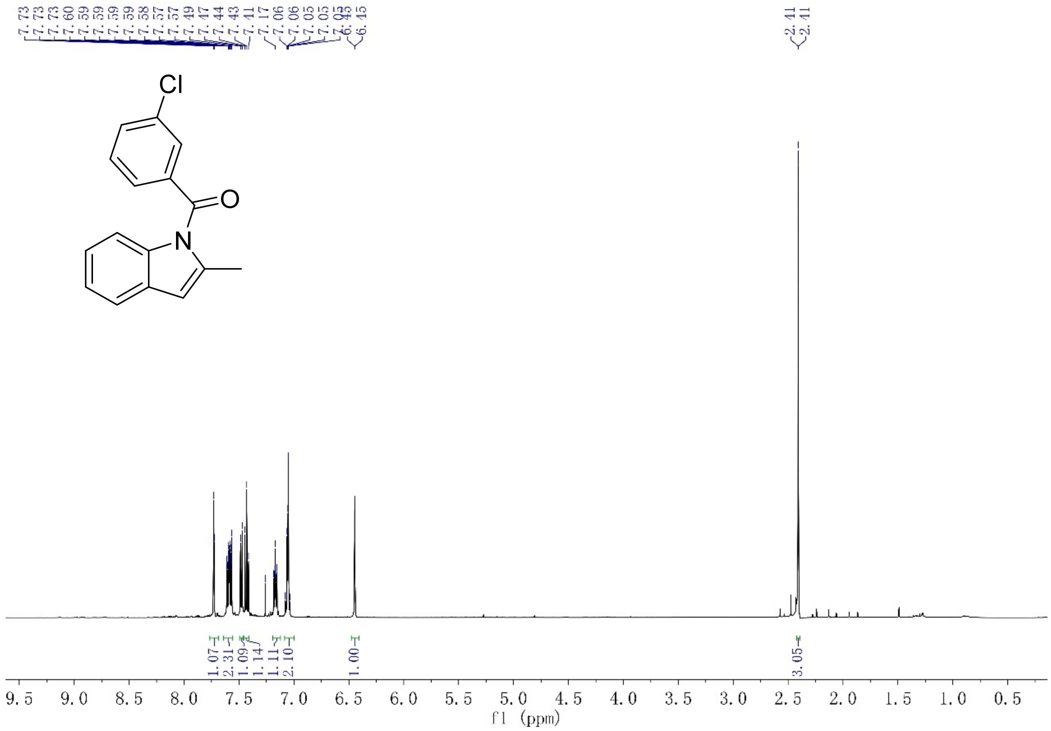

7.73
7.73
7.73
7.60
7.59
7.59
7.59
7.59
7.58
7.57
7.57
7.49
7.47
7.44
7.43
7.41
7.17
7.06
7.06
7.05
7.05
7.05
6.45
6.45
2.41
2.41
Cl
O
N
1.07
2.31
1.09
1.14
1.11
2.10
1.00
3.05
f1 (ppm)

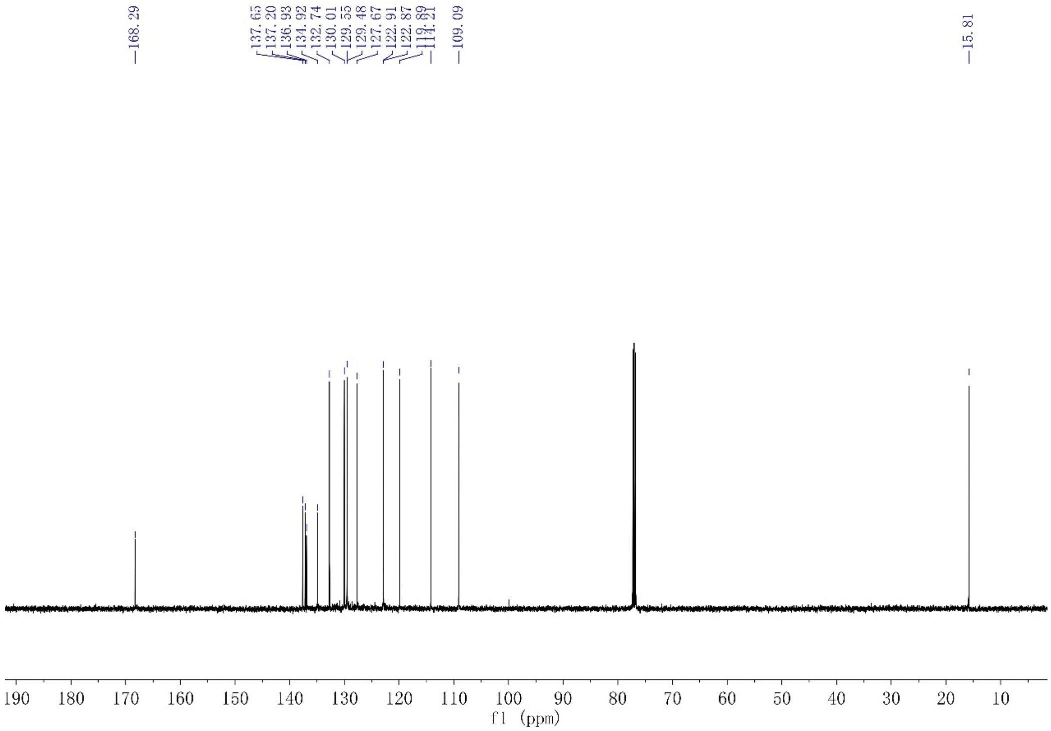

168.29
137.65
137.20
136.93
134.92
132.74
130.01
129.55
129.48
127.67
122.91
122.87
119.89
114.21
109.09
15.81
f1 (ppm)

2e

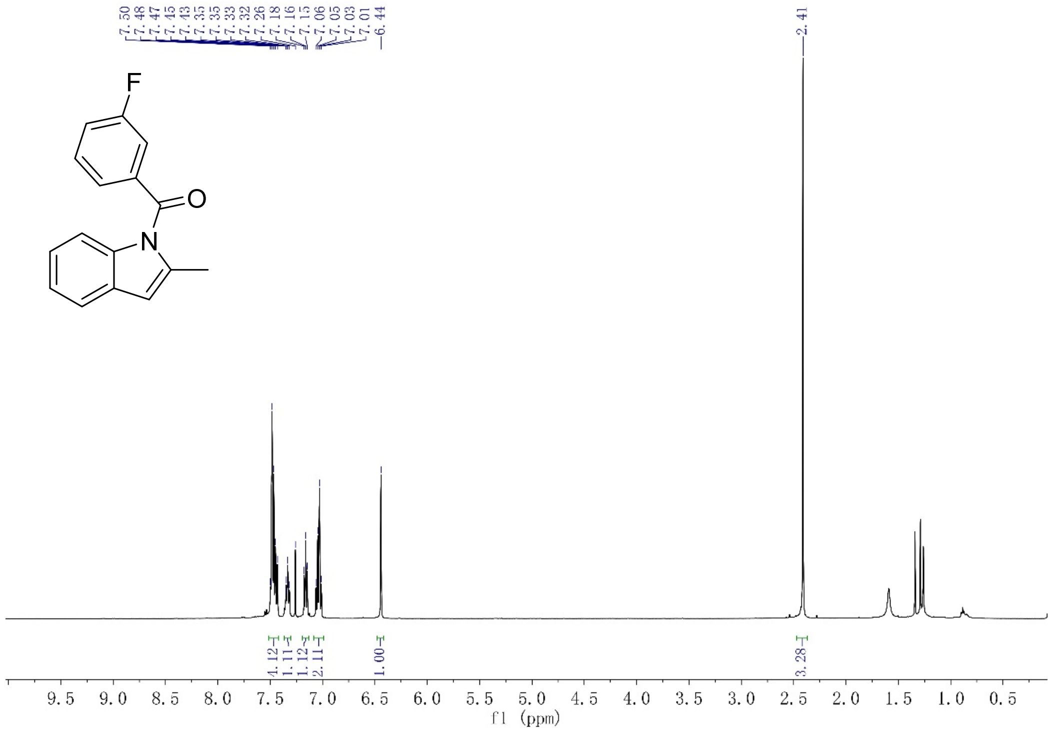
7.50
7.48
7.47
7.45
7.43
7.35
7.35
7.33
7.32
7.26
7.18
7.16
7.15
7.06
7.05
7.03
7.01
6.44
2.41
4.12
1.11
1.12
2.11
1.00
3.28
9.5 9.0 8.5 8.0 7.5 7.0 6.5 6.0 5.5 5.0 4.5 4.0 3.5 3.0 2.5 2.0 1.5 1.0 0.5
f1 (ppm)

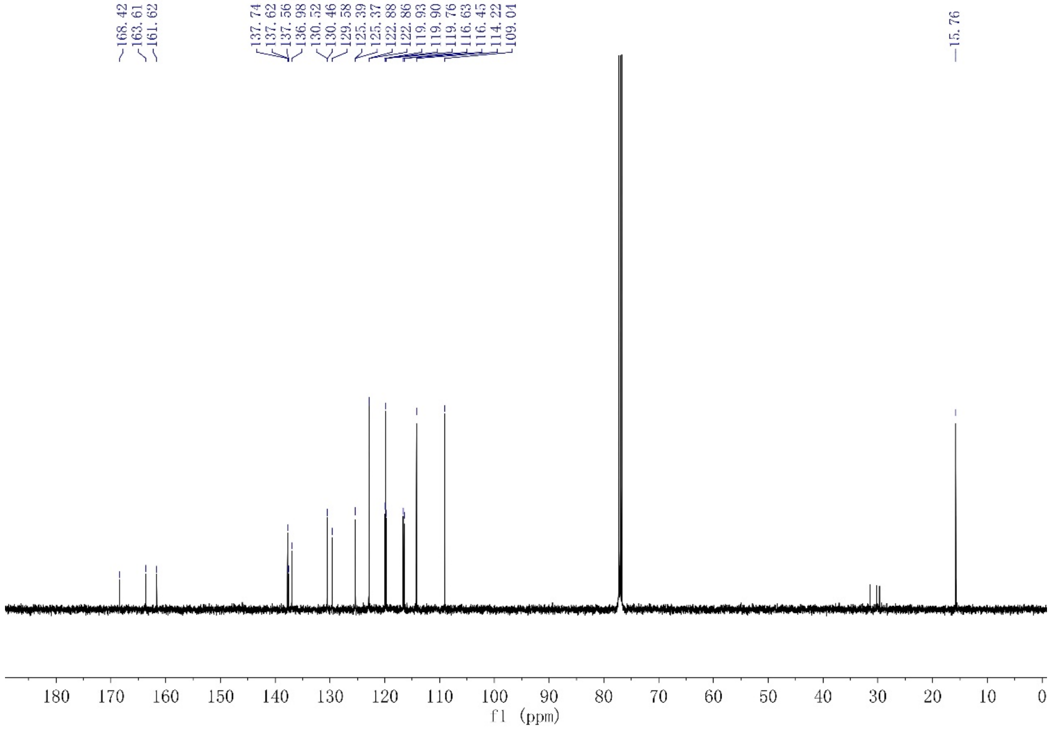
168.42
163.61
161.62
137.74
137.62
137.56
136.98
130.52
130.46
129.58
125.39
125.37
122.88
122.86
119.93
119.90
119.76
116.63
116.45
114.22
109.01
15.76
180 170 160 150 140 130 120 110 100 90 80 70 60 50 40 30 20 10 0
f1 (ppm)

2f

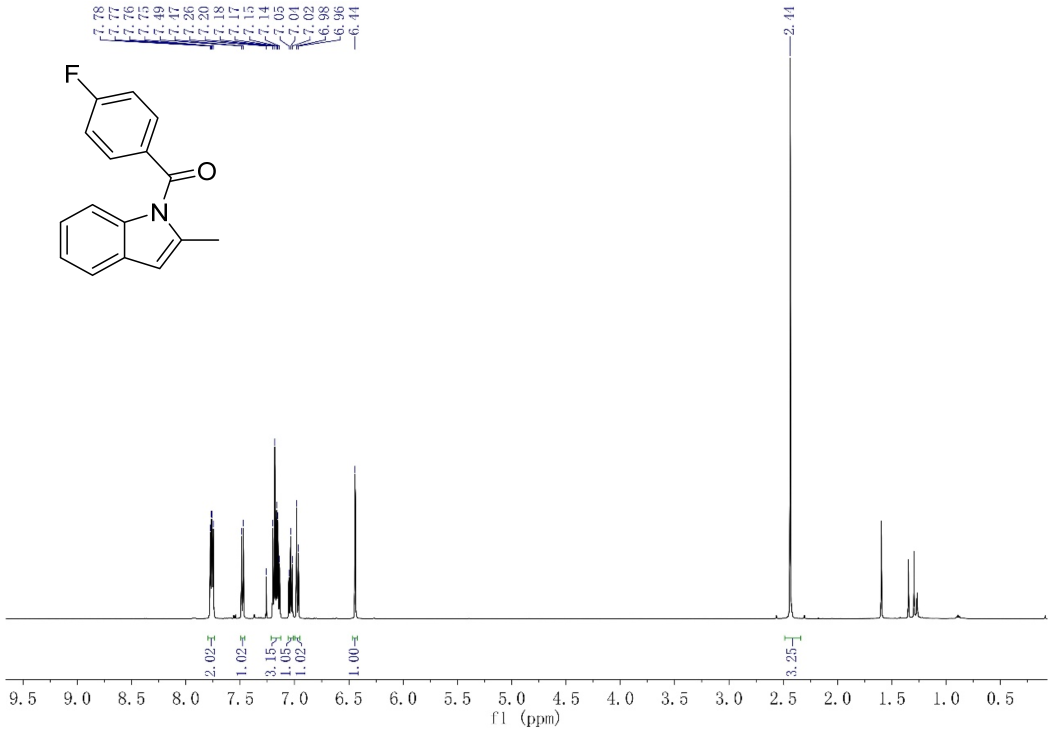
7.78
7.77
7.76
7.75
7.49
7.47
7.26
7.20
7.18
7.17
7.15
7.14
7.05
7.04
7.02
6.98
6.96
6.44
2.44
F
O
N
2.02
1.02
3.15
1.05
1.02
1.00
3.25
9.5 9.0 8.5 8.0 7.5 7.0 6.5 6.0 5.5 5.0 4.5 4.0 3.5 3.0 2.5 2.0 1.5 1.0 0.5
f1 (ppm)

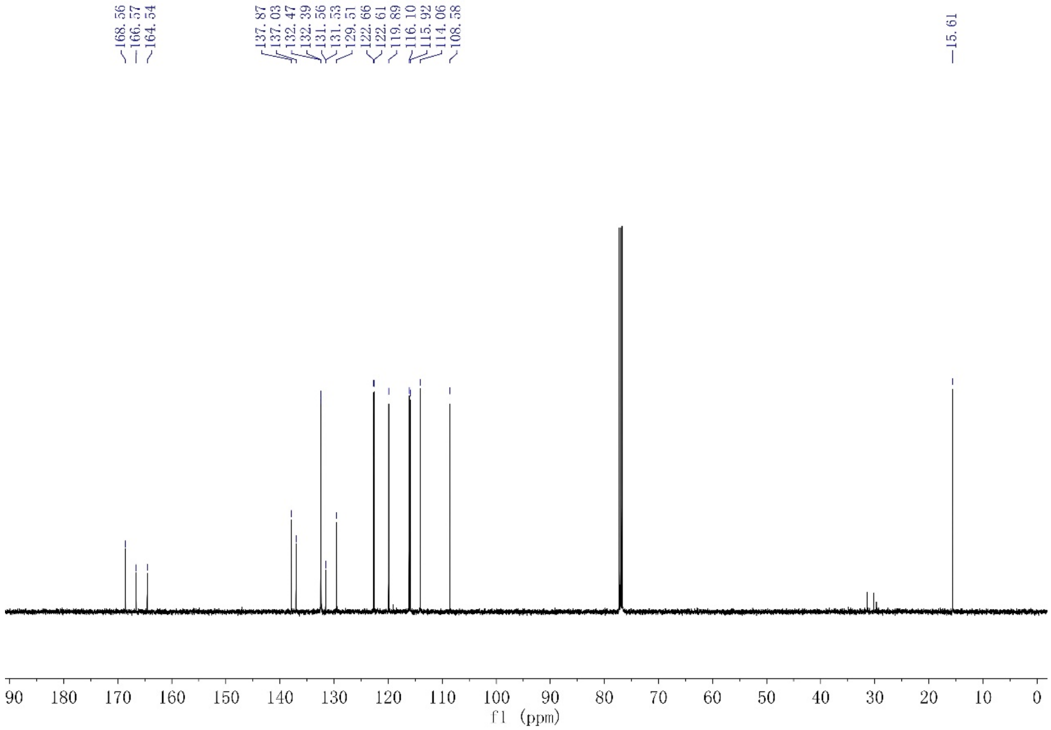
168.56
166.57
164.54
137.87
137.03
132.47
132.39
131.56
131.53
129.51
122.66
122.61
119.89
116.10
115.92
114.06
108.38
15.61
190 180 170 160 150 140 130 120 110 100 90 80 70 60 50 40 30 20 10 0
f1 (ppm)

2g

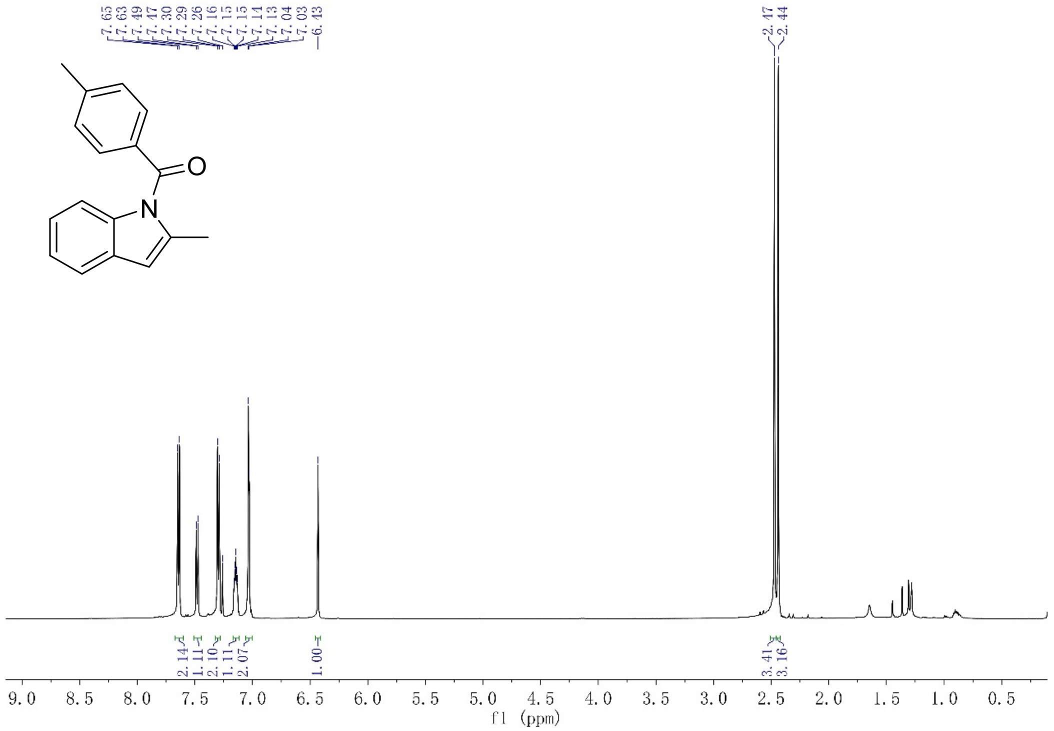

7.65
7.63
7.49
7.47
7.30
7.29
7.26
7.16
7.15
7.15
7.14
7.13
7.04
7.03
6.43
2.47
2.44
O
N
2.14
1.11
2.10
1.11
2.07
1.00
3.41
3.16
9.0 8.5 8.0 7.5 7.0 6.5 6.0 5.5 5.0 4.5 4.0 3.5 3.0 2.5 2.0 1.5 1.0 0.5
f1 (ppm)

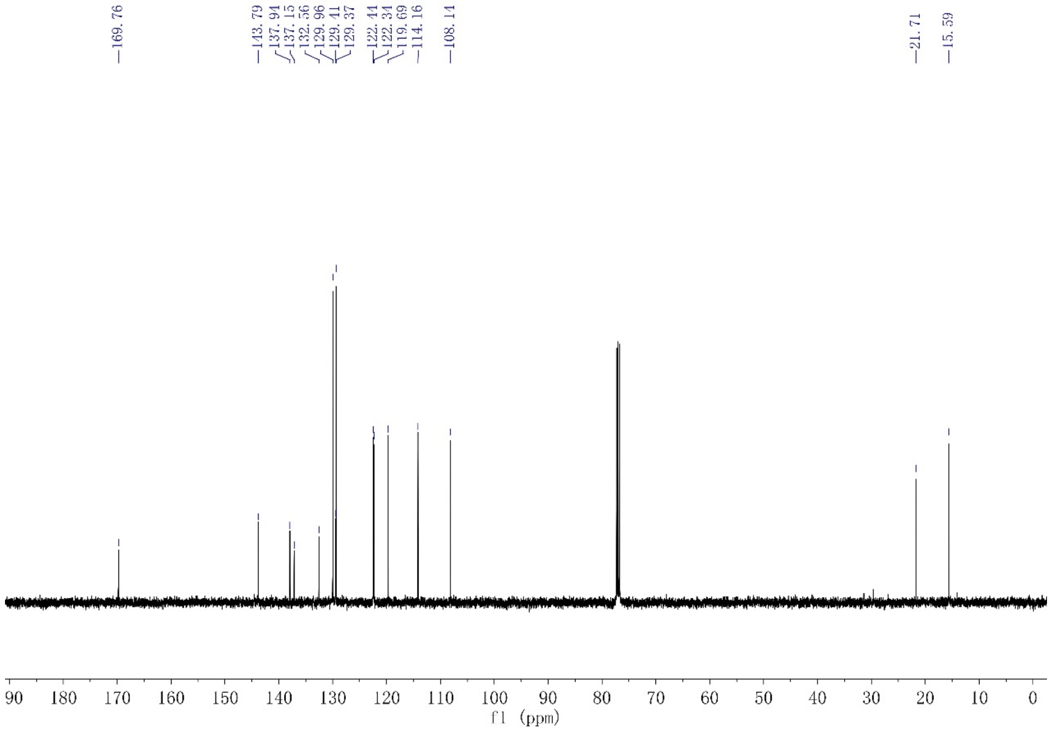

169.76
143.79
137.94
137.15
132.56
129.96
129.41
129.37
122.44
122.34
119.69
114.16
108.14
21.71
15.59
90 180 170 160 150 140 130 120 110 100 90 80 70 60 50 40 30 20 10 0
f1 (ppm)

2h

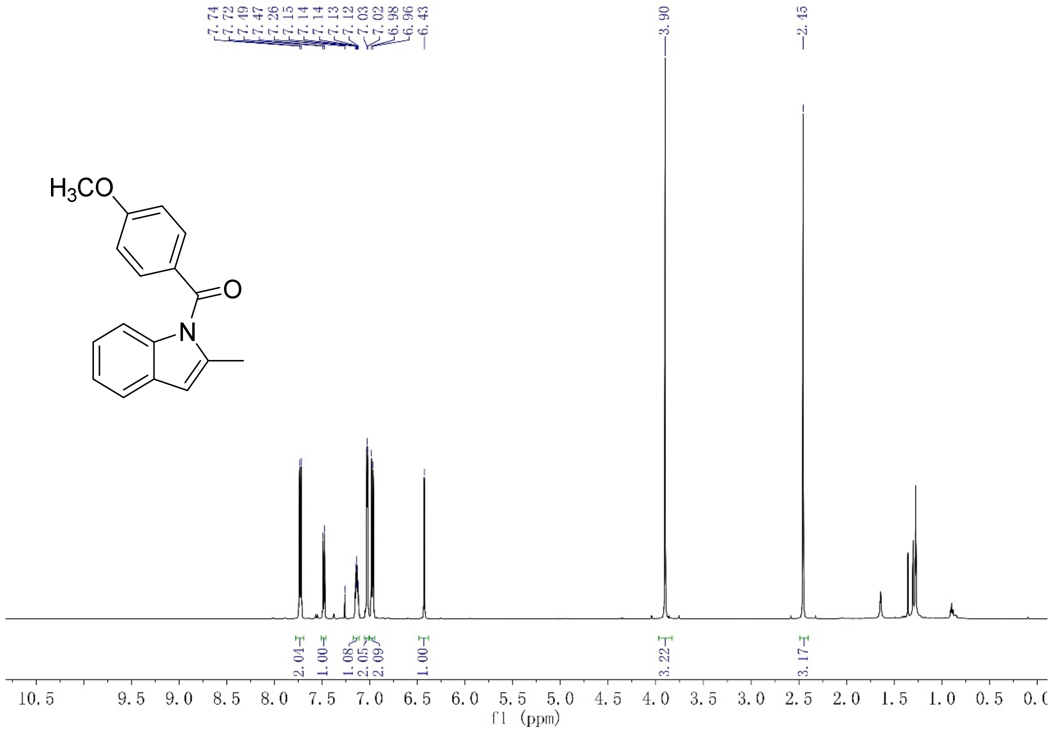
7.74
7.72
7.49
7.47
7.26
7.15
7.14
7.13
7.12
7.03
7.02
6.98
6.96
6.43
3.90
2.45
H_3CO
O
N
2.04
1.00
1.08
2.05
2.09
1.00
3.22
3.17
10.5 9.5 9.0 8.5 8.0 7.5 7.0 6.5 6.0 5.5 5.0 4.5 4.0 3.5 3.0 2.5 2.0 1.5 1.0 0.5 0.0
f1 (ppm)

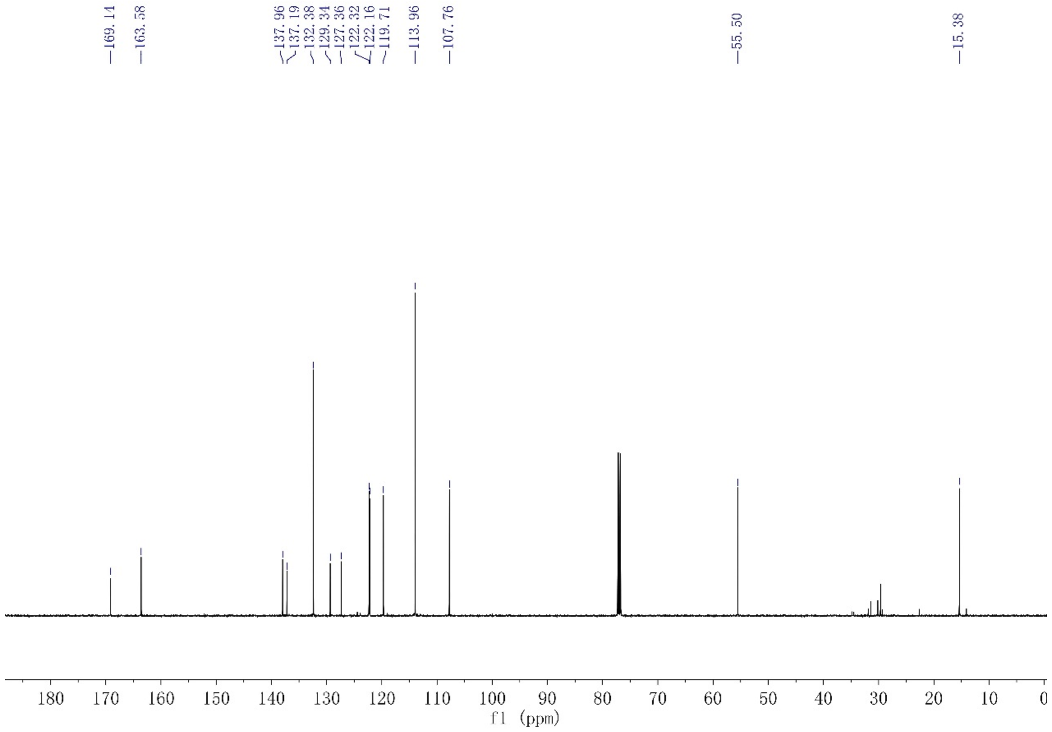
169.14
163.58
137.96
137.19
132.38
129.34
127.36
122.32
122.16
119.71
113.96
107.76
55.50
15.38
180 170 160 150 140 130 120 110 100 90 80 70 60 50 40 30 20 10 0
f1 (ppm)

2i

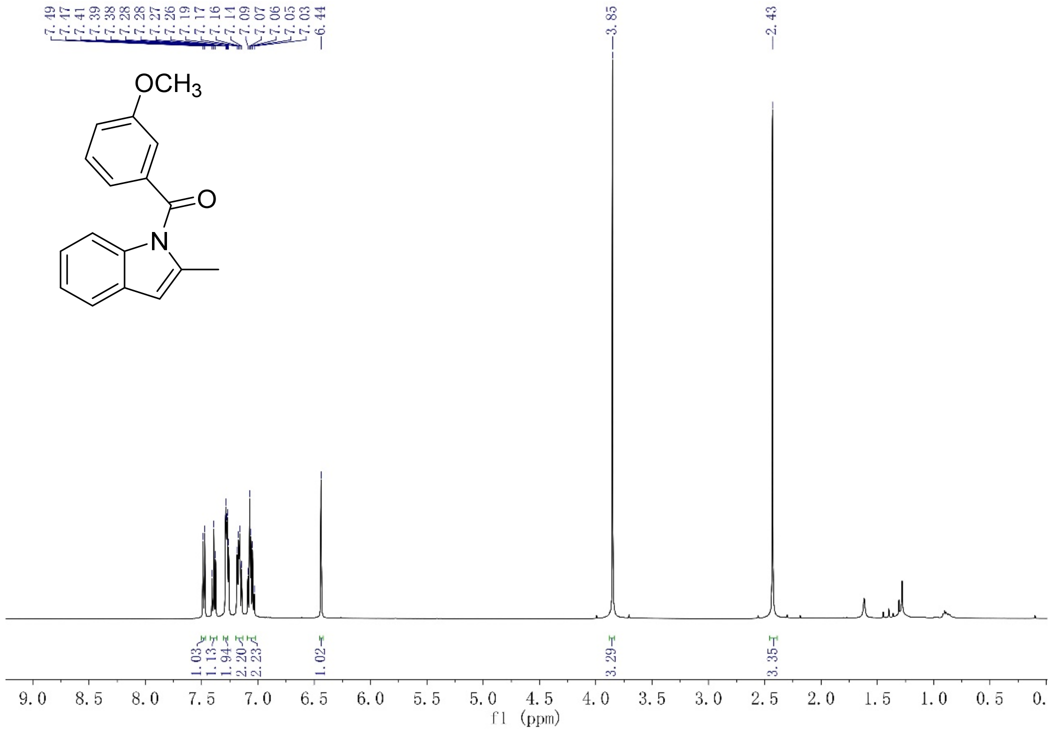

7.49
7.47
7.41
7.39
7.38
7.28
7.28
7.27
7.26
7.19
7.17
7.16
7.14
7.09
7.07
7.06
7.05
7.03
6.44
3.85
2.43
OCH3
O
N
1.03
1.13
1.94
2.20
2.23
1.02
3.29
3.35
9.0 8.5 8.0 7.5 7.0 6.5 6.0 5.5 5.0 4.5 4.0 3.5 3.0 2.5 2.0 1.5 1.0 0.5 0.
f1 (ppm)

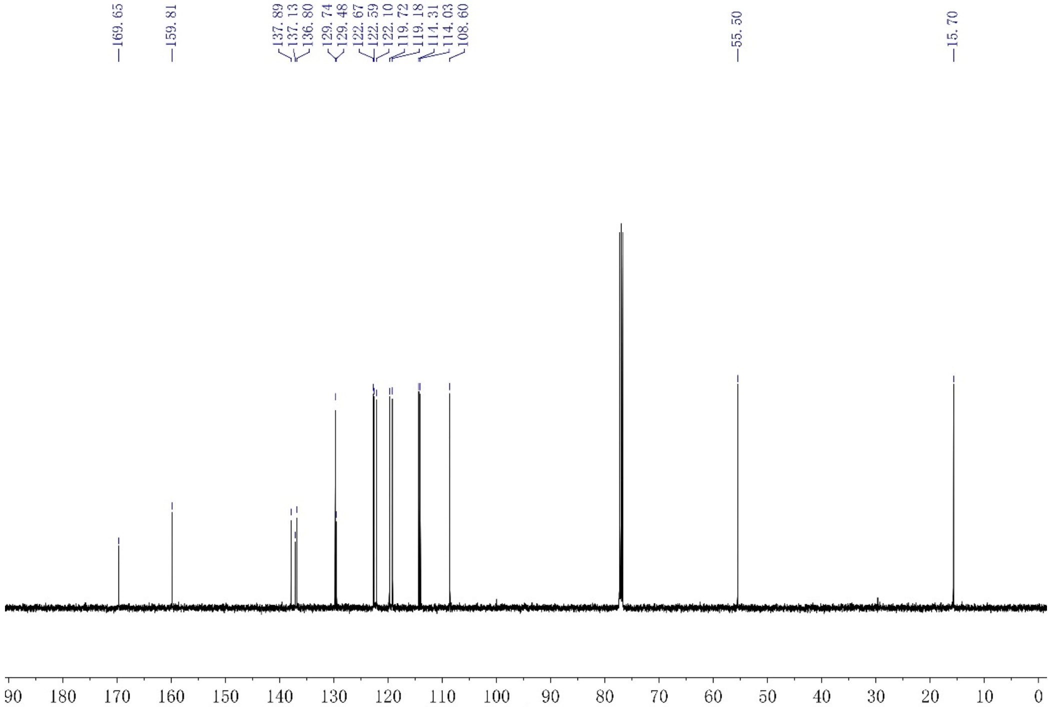

169.65
159.81
137.89
137.13
136.80
129.74
129.48
122.67
122.59
122.10
119.72
119.18
114.31
114.03
108.60
55.50
15.70
90 180 170 160 150 140 130 120 110 100 90 80 70 60 50 40 30 20 10 0
f1 (ppm)

2j

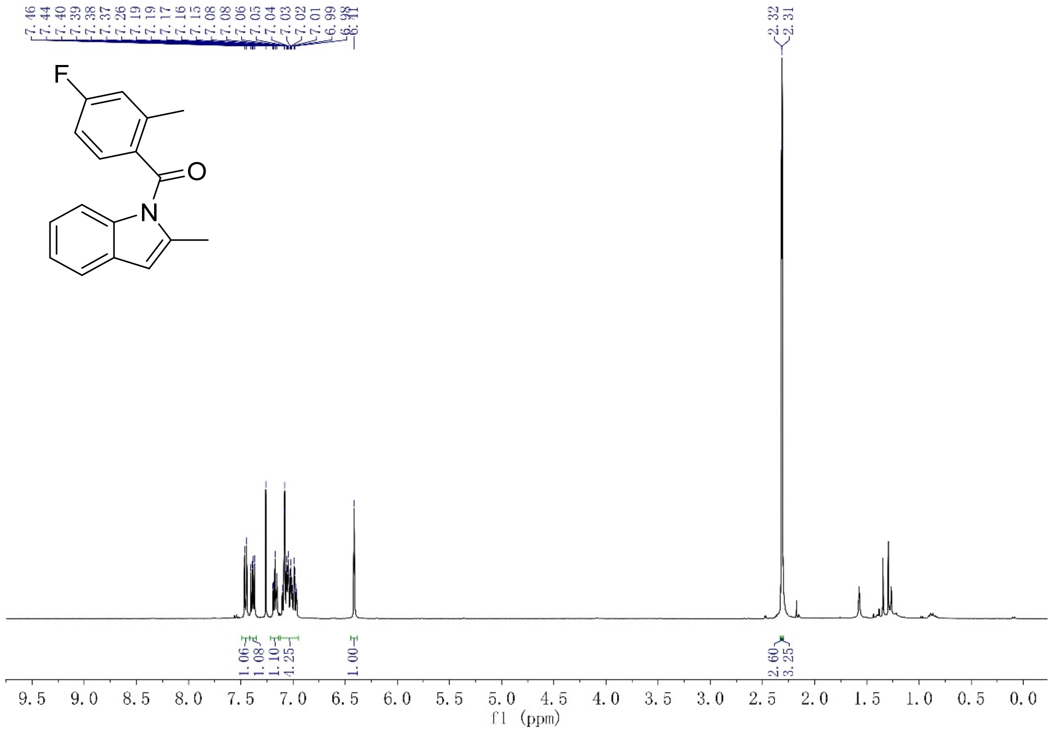
F
O
N
7.46 7.44 7.40 7.39 7.38 7.37 7.26 7.19 7.17 7.16 7.15 7.08 7.08 7.06 7.05 7.04 7.03 7.02 7.01 6.99 6.98 6.41
2.32 2.31
1.06 1.08 1.10 4.25 1.00 2.60 3.25
9.5 9.0 8.5 8.0 7.5 7.0 6.5 6.0 5.5 5.0 4.5 4.0 3.5 3.0 2.5 2.0 1.5 1.0 0.5 0.0
f1 (ppm)

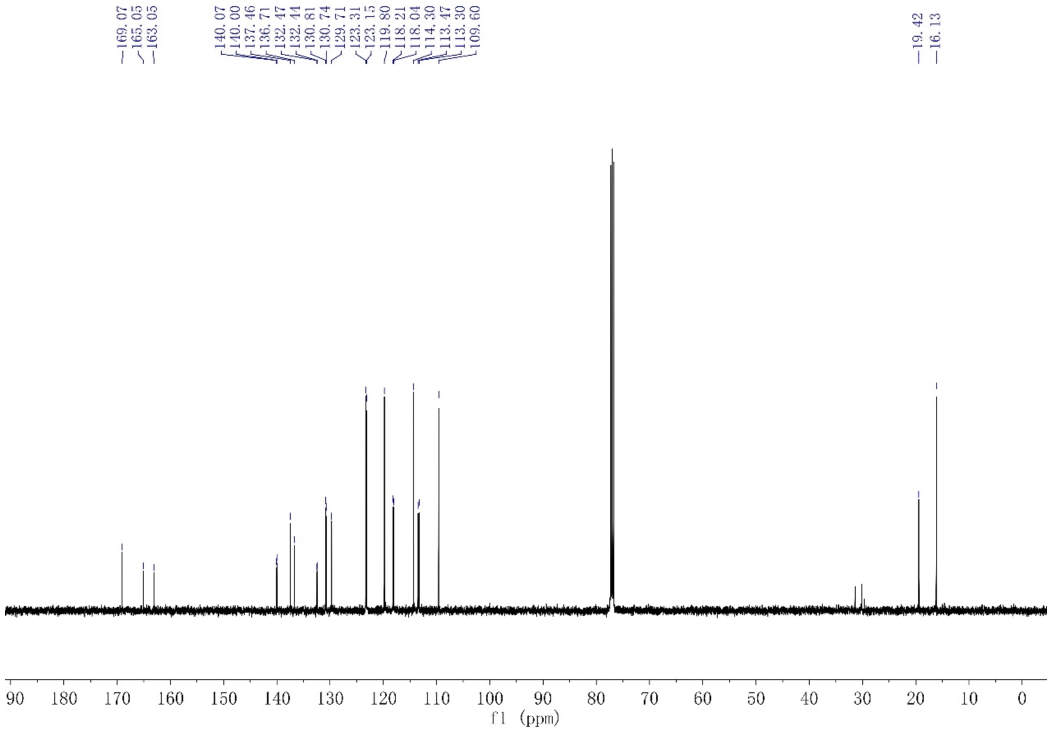
169.07 165.05 163.05
140.07 140.00 137.46 136.71 132.47 132.41 130.81 130.74 129.71 123.31 123.15 119.80 118.21 118.04 114.30 113.47 113.30 109.60
19.42 16.13
190 180 170 160 150 140 130 120 110 100 90 80 70 60 50 40 30 20 10 0
f1 (ppm)

2u

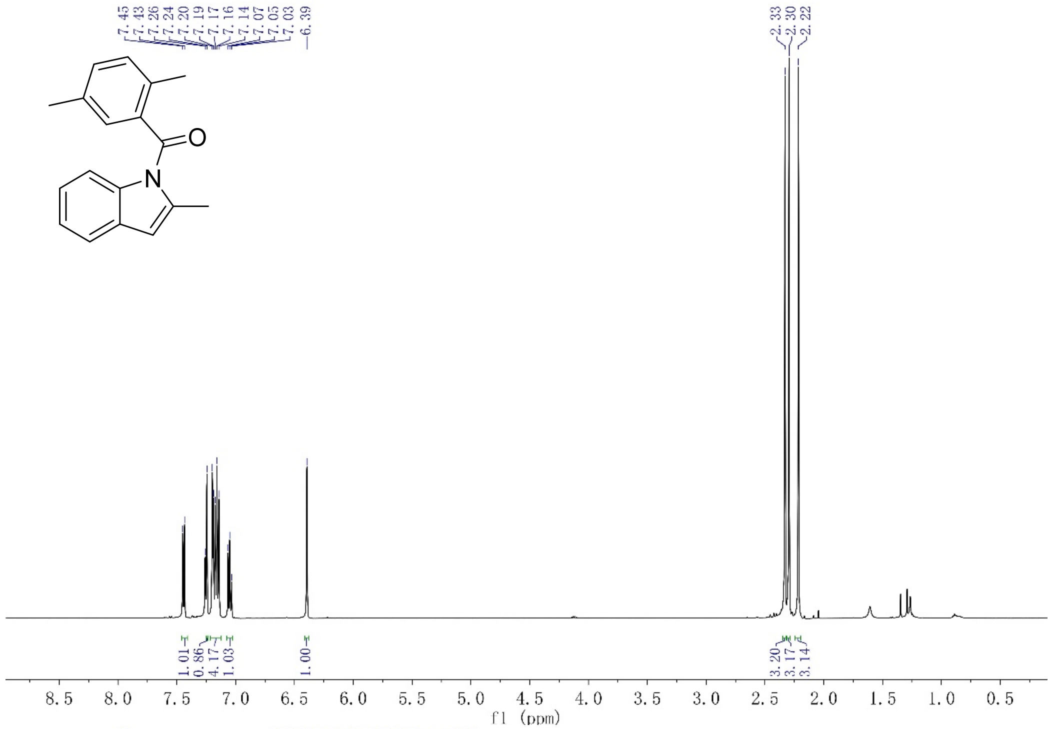
7.45
7.43
7.26
7.24
7.20
7.19
7.17
7.16
7.14
7.07
7.05
7.03
6.39
2.33
2.30
2.22
O
N
1.01
0.86
4.17
1.03
1.00
3.20
3.17
3.14
8.5 8.0 7.5 7.0 6.5 6.0 5.5 5.0 4.5 4.0 3.5 3.0 2.5 2.0 1.5 1.0 0.5
f1 (ppm)

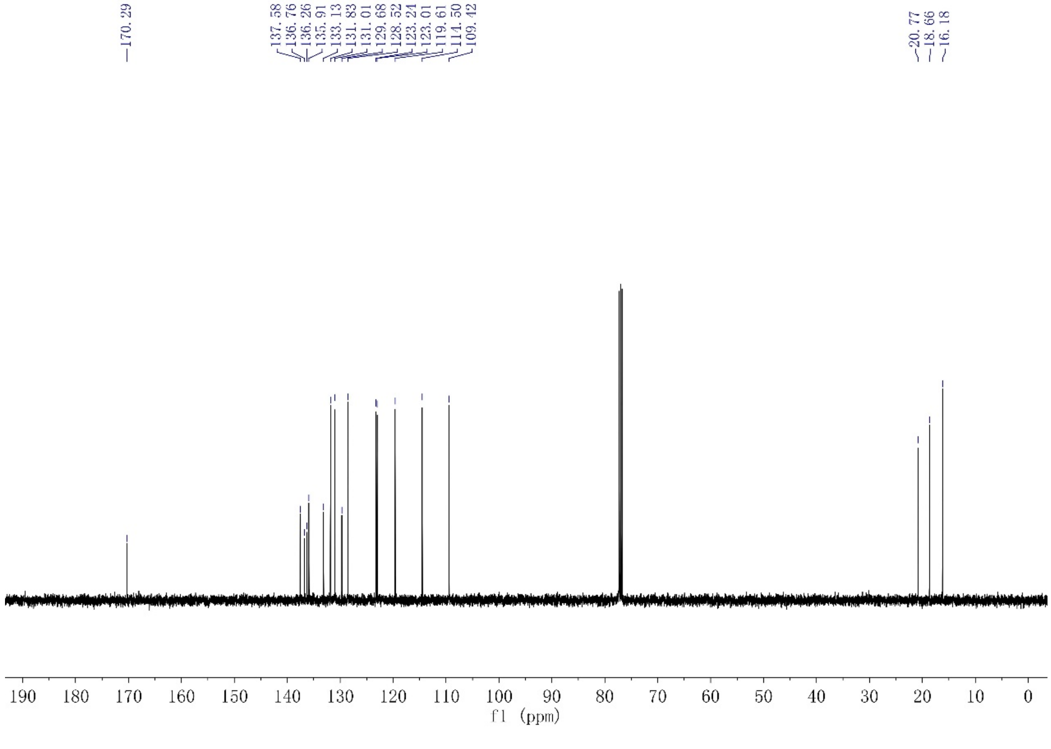
170.29
137.58
136.76
136.26
135.91
133.13
131.83
131.01
129.68
128.52
123.21
123.01
119.61
114.50
109.42
20.77
18.66
16.18
190 180 170 160 150 140 130 120 110 100 90 80 70 60 50 40 30 20 10 0
f1 (ppm)

2k

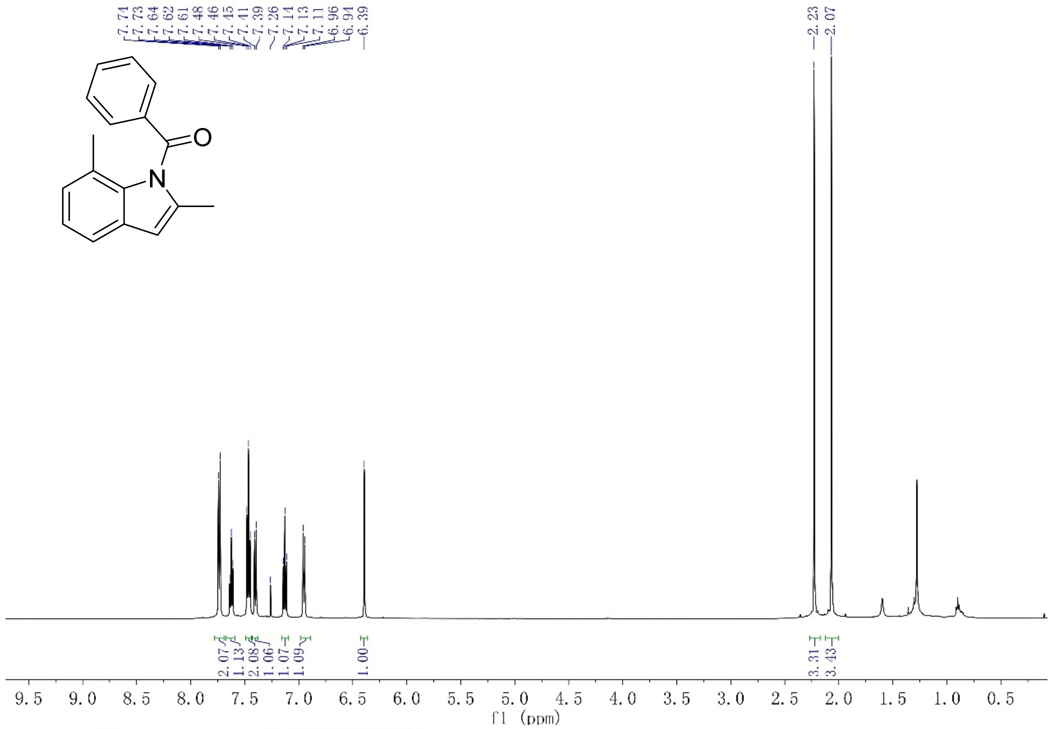
7.74 7.73 7.64 7.62 7.61 7.48 7.46 7.45 7.41 7.39 7.26 7.14 7.13 7.11 6.96 6.94 6.39
2.23 2.07
O
N
2.07 1.13 2.08 1.06 1.07 1.09 1.00 3.31 3.43
9.5 9.0 8.5 8.0 7.5 7.0 6.5 6.0 5.5 5.0 4.5 4.0 3.5 3.0 2.5 2.0 1.5 1.0 0.5
f1 (ppm)

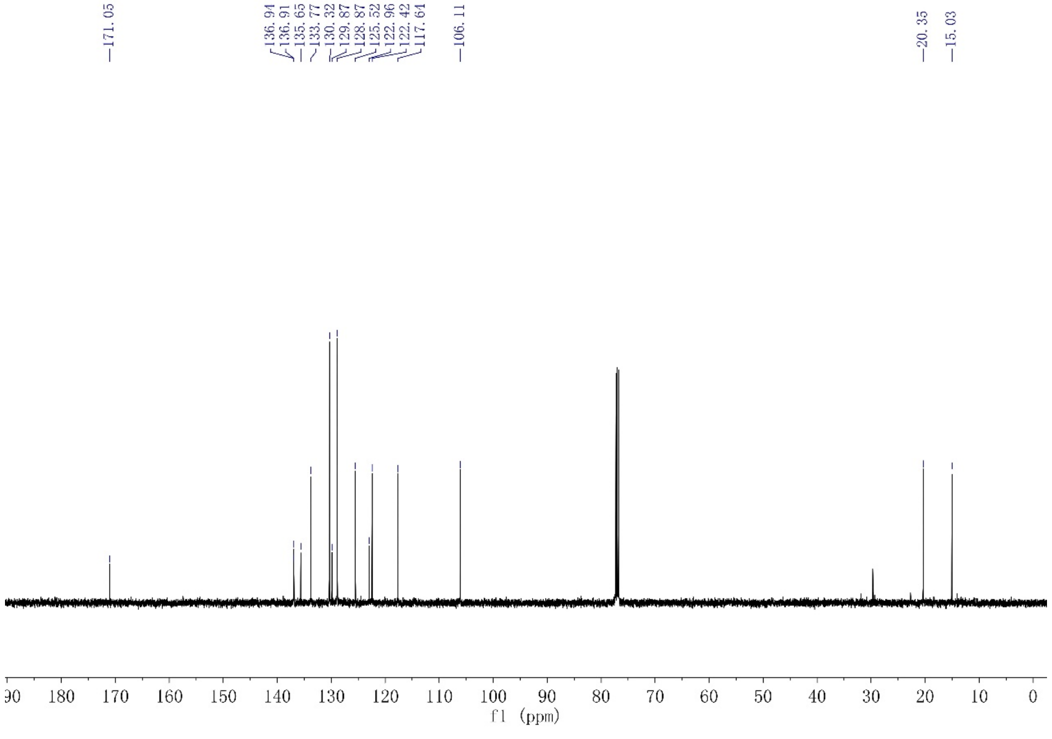
171.05
136.94 136.91 135.65 133.77 130.32 129.87 128.87 125.52 122.96 122.42 117.64
106.11
20.35 15.03
90 180 170 160 150 140 130 120 110 100 90 80 70 60 50 40 30 20 10 0
f1 (ppm)

2l

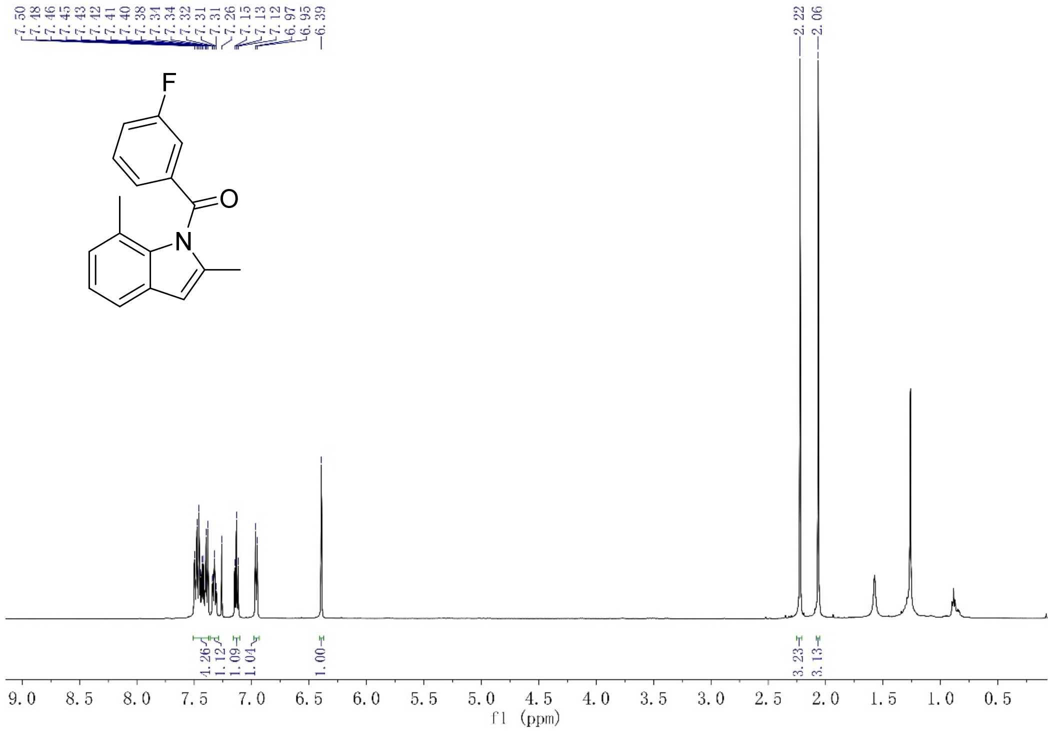
7.50
7.48
7.46
7.45
7.43
7.42
7.41
7.40
7.38
7.34
7.34
7.32
7.31
7.31
7.26
7.15
7.13
7.12
6.97
6.95
6.39
2.22
2.06
F
O
N
4.26
1.12
1.09
1.04
1.00
3.23
3.13
9.0 8.5 8.0 7.5 7.0 6.5 6.0 5.5 5.0 4.5 4.0 3.5 3.0 2.5 2.0 1.5 1.0 0.5
f1 (ppm)

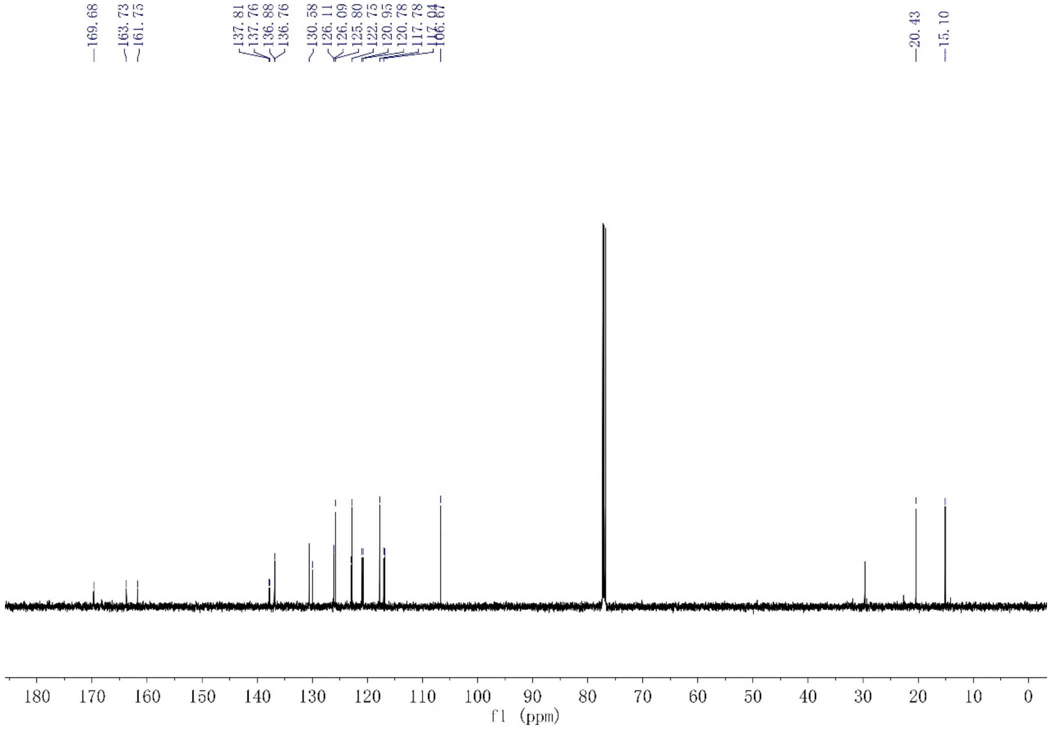
169.68
163.73
161.75
137.81
137.76
136.88
136.76
130.58
126.11
126.09
125.80
122.75
120.95
120.78
117.78
20.43
15.10
180 170 160 150 140 130 120 110 100 90 80 70 60 50 40 30 20 10 0
f1 (ppm)

2m

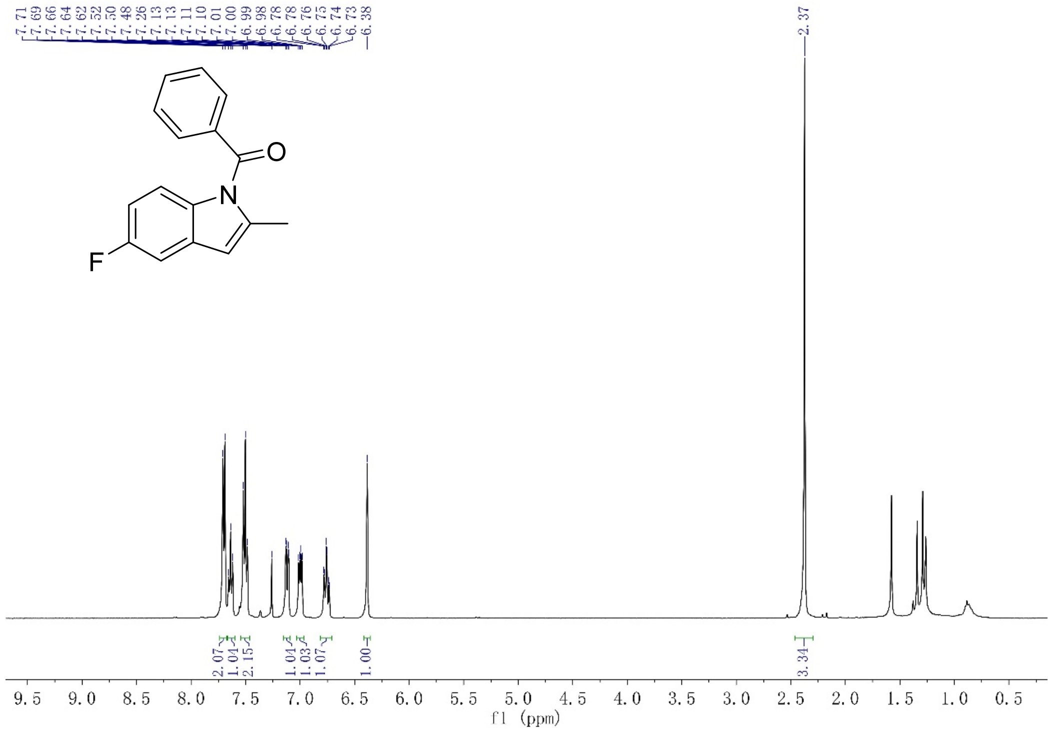
7.71
7.69
7.66
7.64
7.62
7.52
7.50
7.48
7.26
7.13
7.13
7.11
7.10
7.01
7.00
6.99
6.98
6.78
6.78
6.76
6.75
6.74
6.73
6.38
2.37
O
N
F
2.07
1.04
2.15
1.04
1.03
1.07
1.00
3.34
9.5 9.0 8.5 8.0 7.5 7.0 6.5 6.0 5.5 5.0 4.5 4.0 3.5 3.0 2.5 2.0 1.5 1.0 0.5
f1 (ppm)

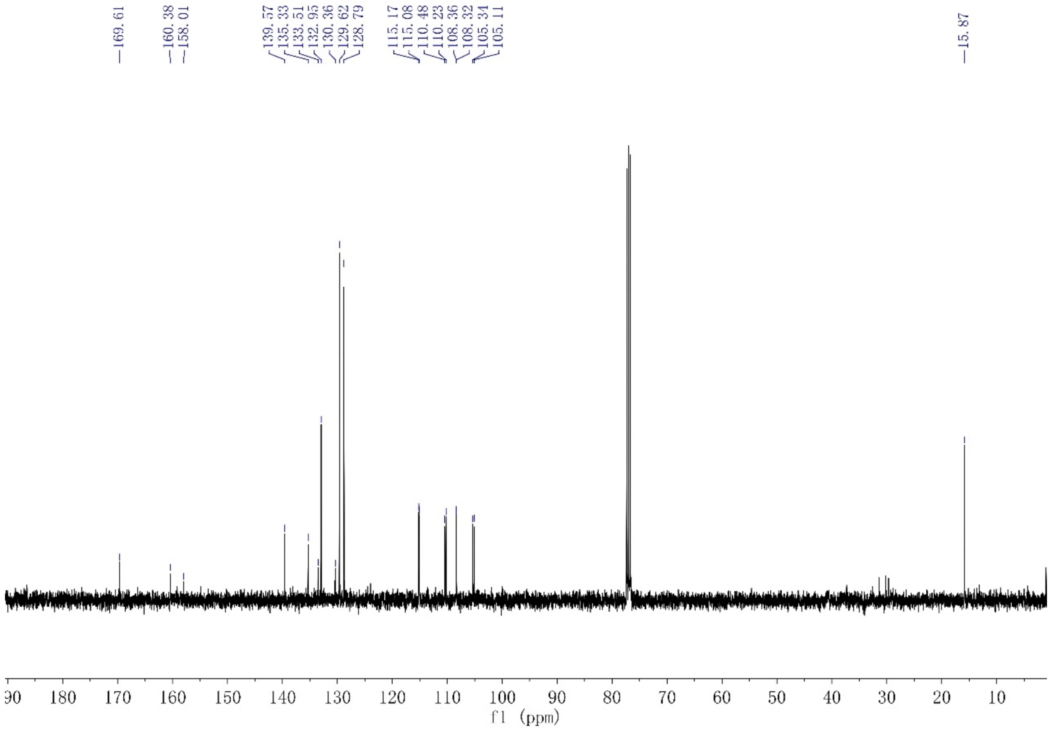
169.61
160.38
158.01
139.57
135.33
133.51
132.95
130.36
129.62
128.79
115.17
115.08
110.48
110.23
108.36
108.32
105.31
105.11
15.87
90 180 170 160 150 140 130 120 110 100 90 80 70 60 50 40 30 20 10
f1 (ppm)

2n

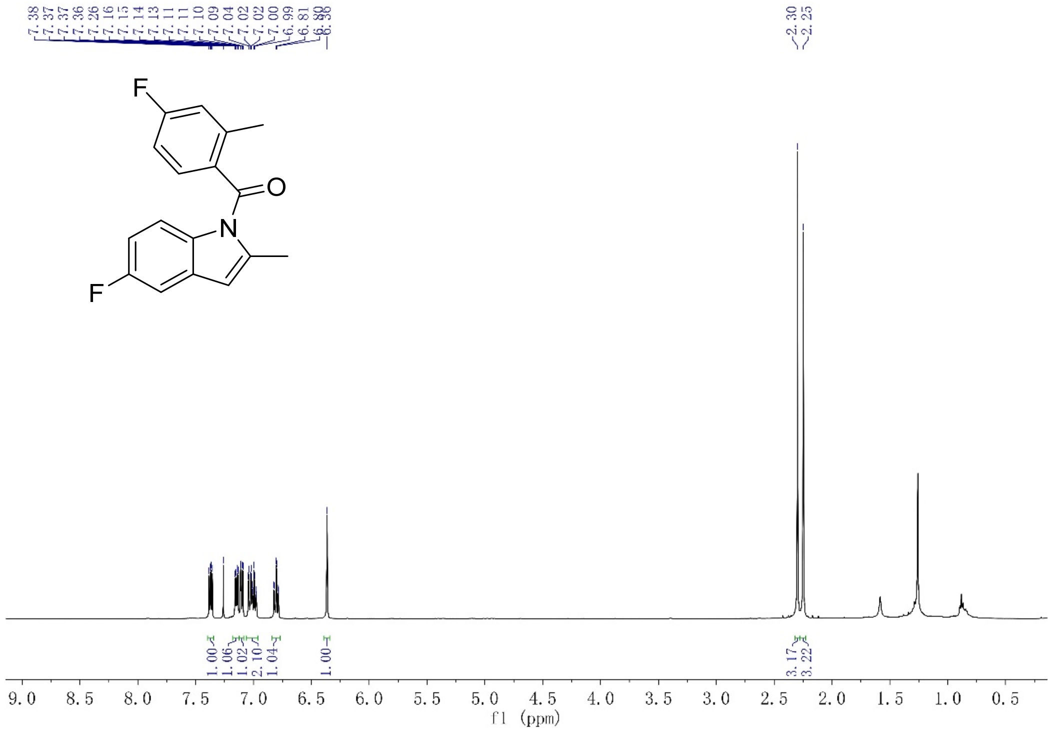

7.38
7.37
7.37
7.36
7.26
7.16
7.15
7.14
7.13
7.11
7.11
7.10
7.09
7.04
7.02
7.02
7.00
6.99
6.81
2.30
2.25
1.00
1.06
1.02
2.10
1.04
1.00
3.17
3.22
f1 (ppm)

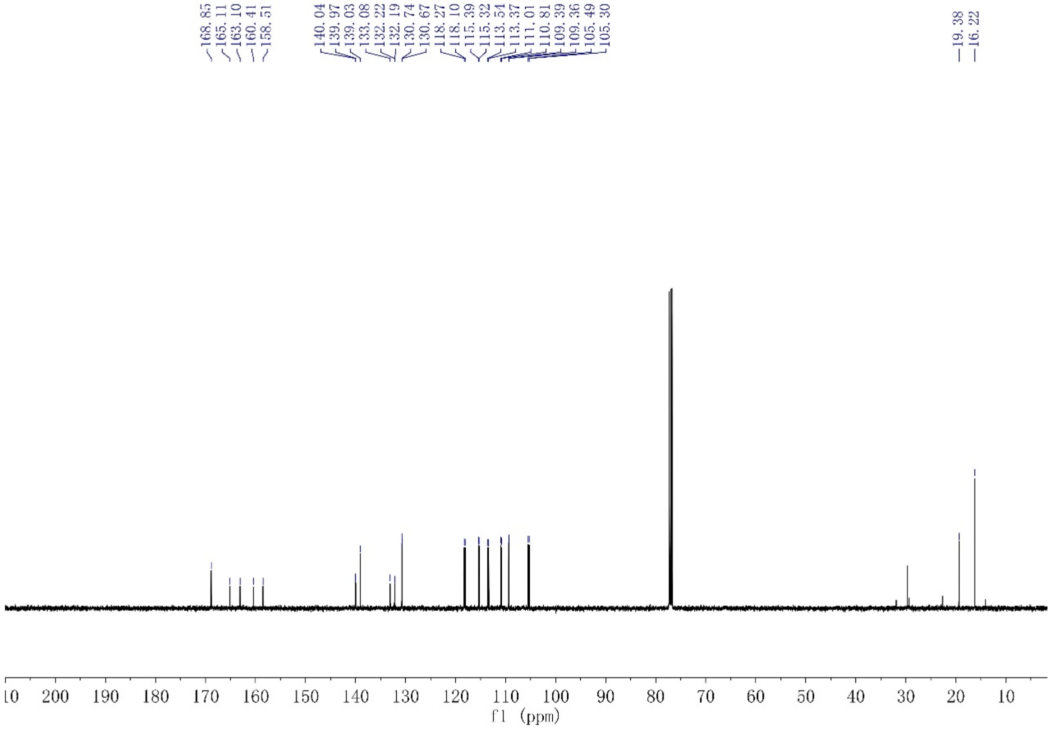

168.85
165.11
163.10
160.41
158.51
140.04
139.97
139.03
133.08
132.22
132.19
130.74
130.67
118.27
118.10
115.39
115.32
113.54
113.37
111.01
110.81
109.39
109.36
105.49
105.30
19.38
16.22
f1 (ppm)

2o

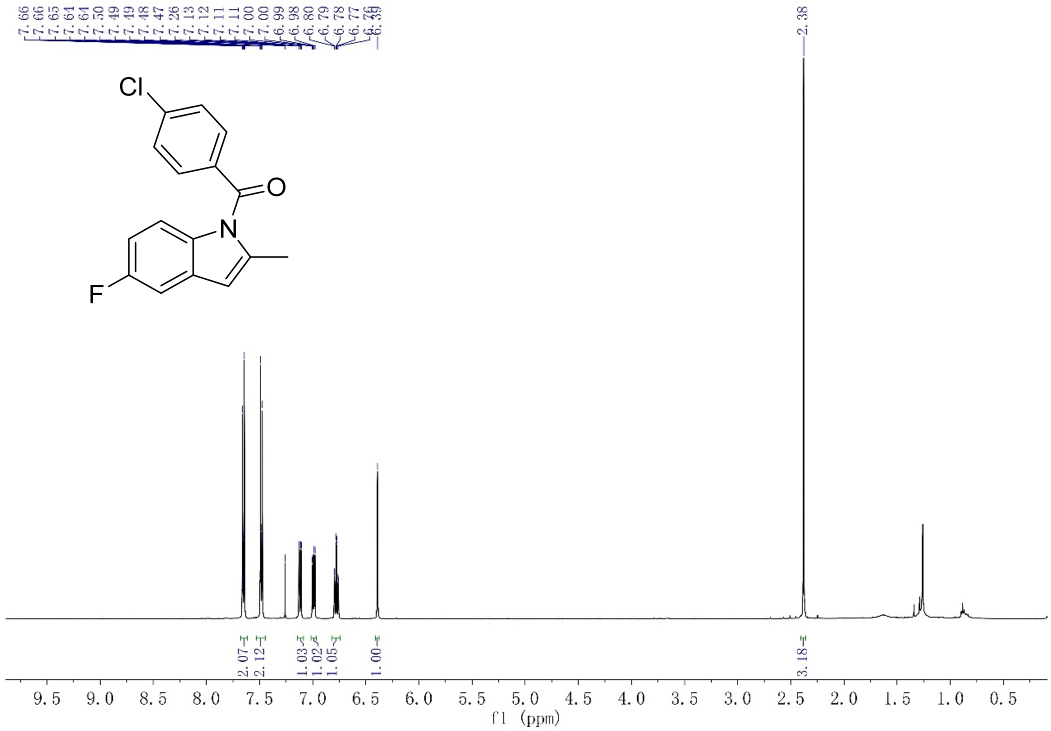
7.66
7.66
7.65
7.64
7.64
7.50
7.49
7.49
7.48
7.47
7.26
7.13
7.12
7.11
7.11
7.00
7.00
6.99
6.98
6.80
6.79
6.78
6.77
6.39
2.38
Cl
O
N
F
2.07
2.12
1.03
1.02
1.05
1.00
3.18
9.5 9.0 8.5 8.0 7.5 7.0 6.5 6.0 5.5 5.0 4.5 4.0 3.5 3.0 2.5 2.0 1.5 1.0 0.5
f1 (ppm)

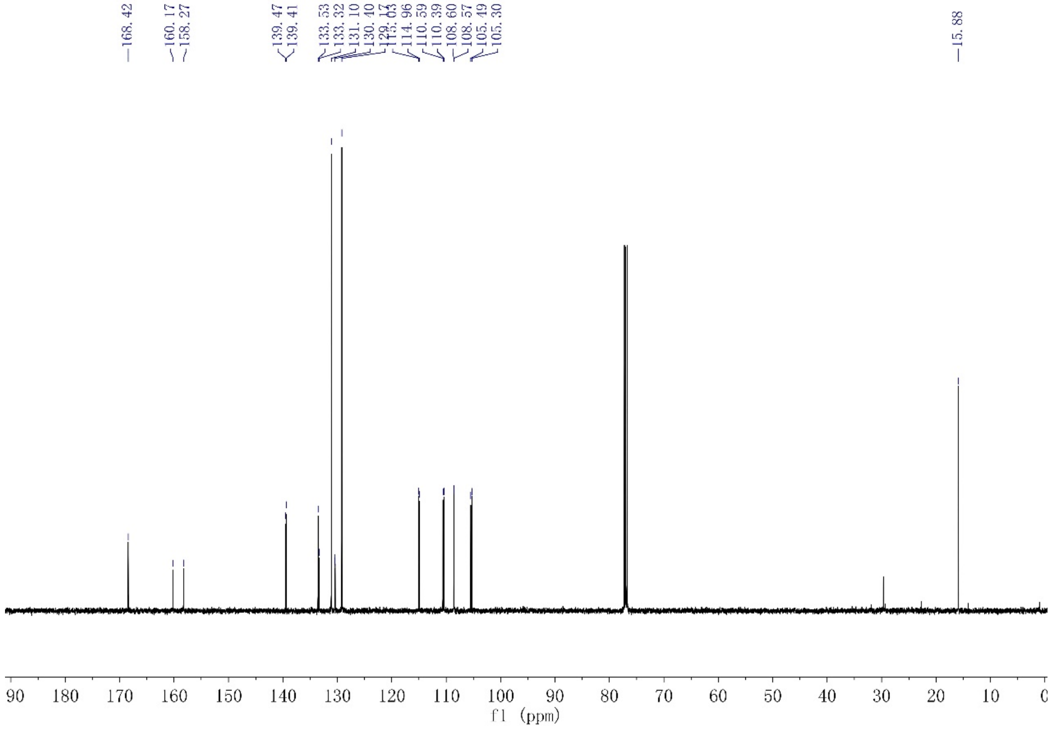
168.42
160.17
158.27
139.47
139.41
133.53
133.32
131.10
130.40
114.96
110.59
110.39
108.60
108.57
105.49
105.30
15.88
180 170 160 150 140 130 120 110 100 90 80 70 60 50 40 30 20 10
f1 (ppm)

2p

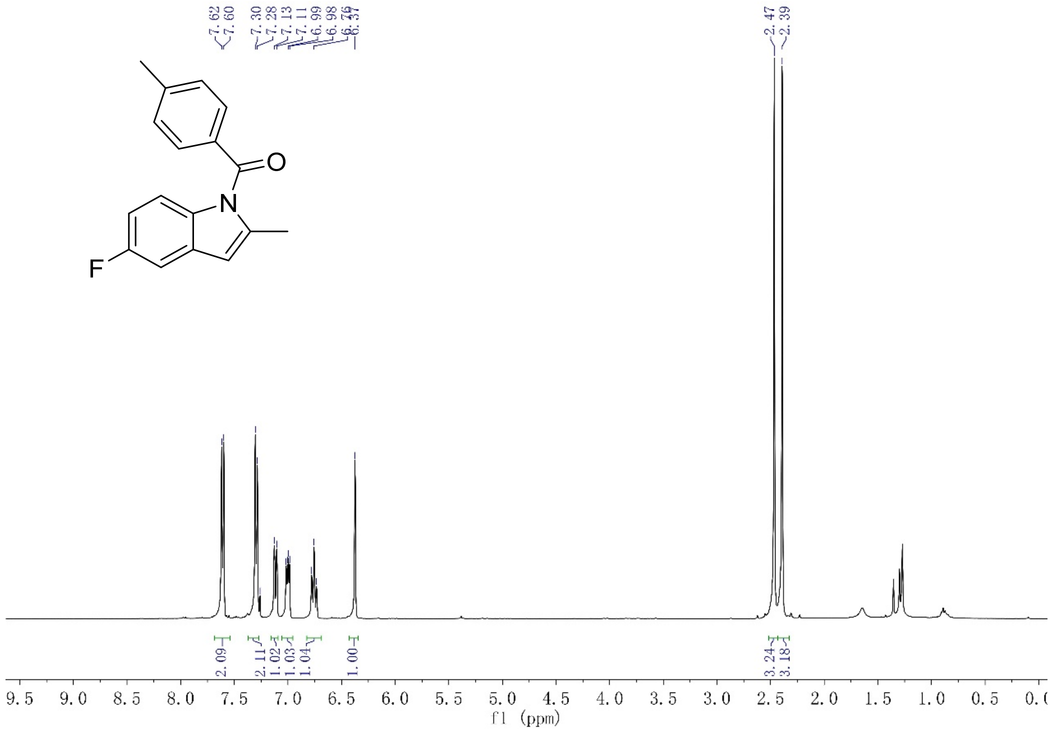
O
N
F
7.62
7.60
7.30
7.28
7.13
7.11
6.99
6.98
2.47
2.39
2.09
2.11
1.02
1.03
1.04
1.00
3.24
3.18
9.5 9.0 8.5 8.0 7.5 7.0 6.5 6.0 5.5 5.0 4.5 4.0 3.5 3.0 2.5 2.0 1.5 1.0 0.5
f1 (ppm)

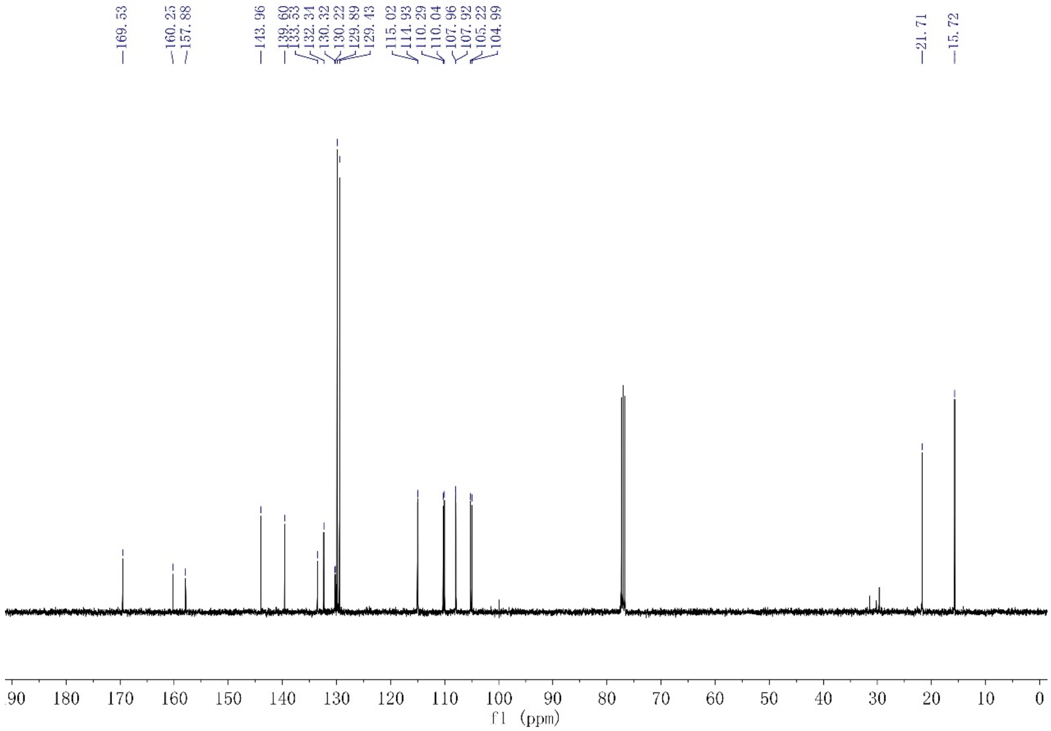
169.53
160.25
157.88
143.96
139.60
133.53
132.31
130.32
130.22
129.89
129.43
115.02
114.93
110.29
110.04
107.96
107.92
105.22
104.99
21.71
15.72
180 170 160 150 140 130 120 110 100 90 80 70 60 50 40 30 20 10 0
f1 (ppm)

2q

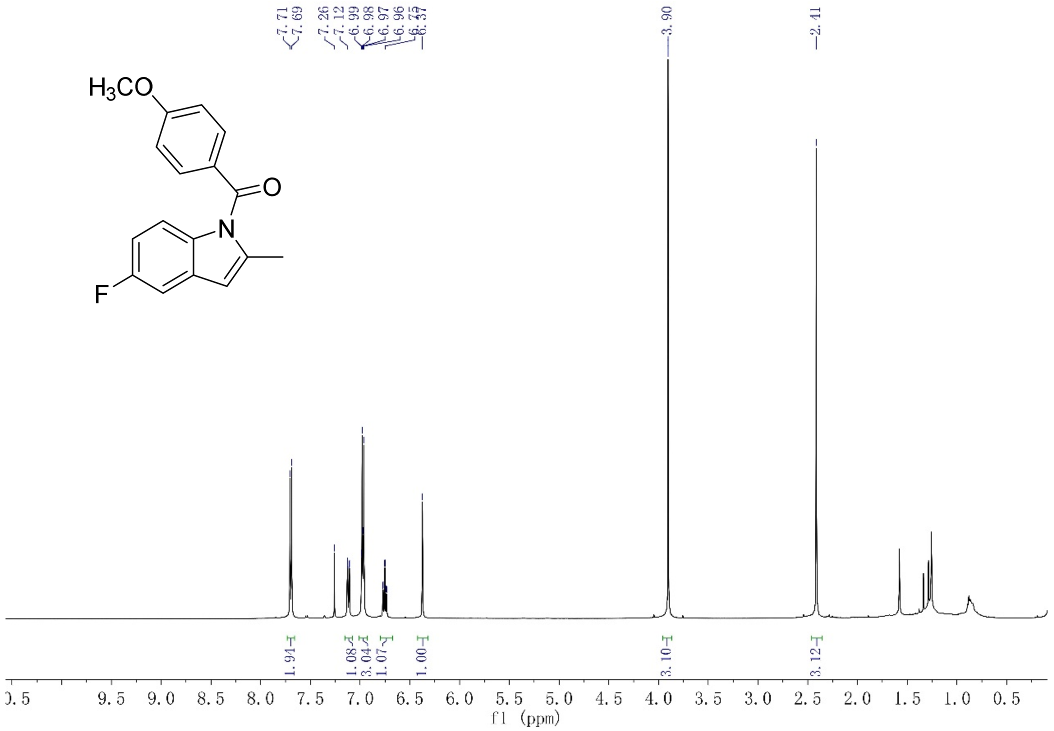

7.71
7.69
7.26
7.12
6.99
6.98
6.97
6.96
3.90
2.41
H_3CO
O
N
F
1.94
1.08
3.04
1.07
1.00
3.10
3.12
f1 (ppm)

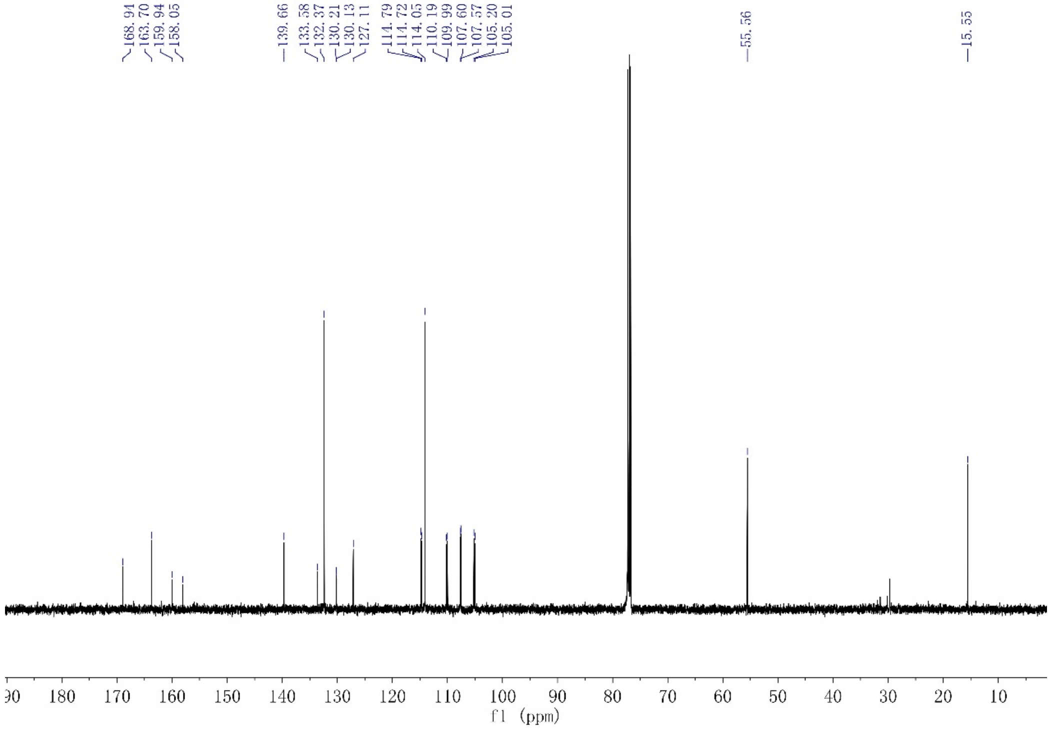

168.91
163.70
159.94
158.05
139.66
133.58
132.37
130.21
130.13
127.11
114.79
114.72
114.05
110.19
109.99
107.60
107.57
105.20
105.01
55.56
15.55
f1 (ppm)

2r

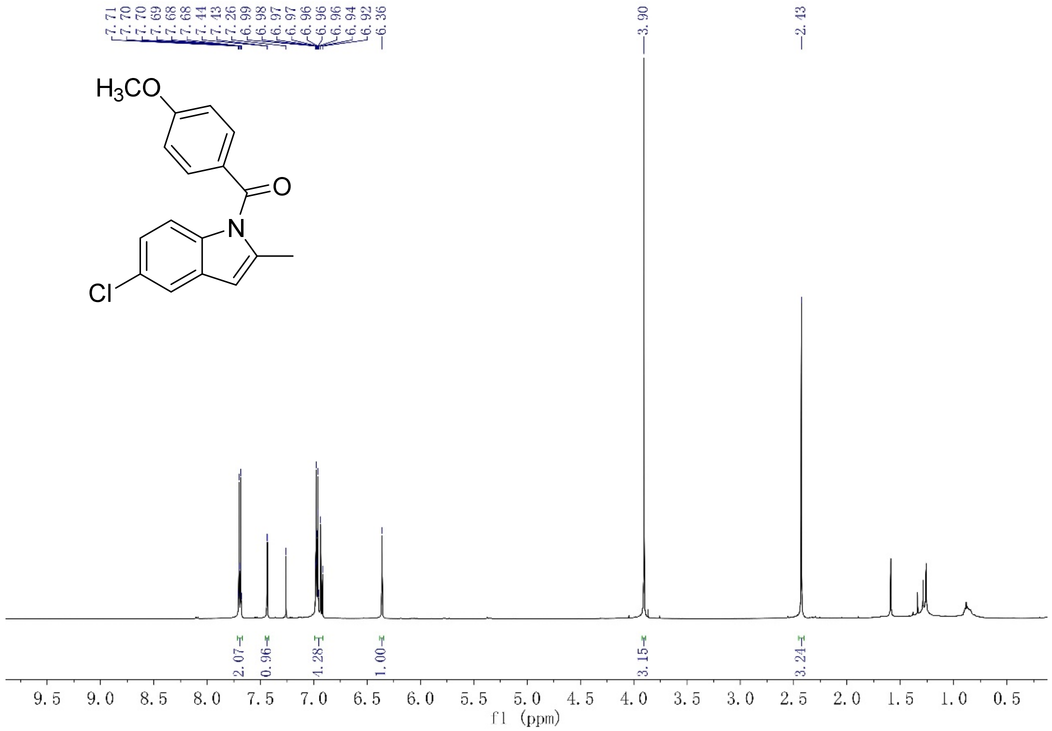
7.71
7.70
7.70
7.69
7.68
7.68
7.44
7.43
7.26
6.99
6.98
6.97
6.97
6.96
6.96
6.96
6.94
6.92
6.36
3.90
2.43
H3CO
O
N
Cl
2.07
0.96
4.28
1.00
3.15
3.24
9.5 9.0 8.5 8.0 7.5 7.0 6.5 6.0 5.5 5.0 4.5 4.0 3.5 3.0 2.5 2.0 1.5 1.0 0.5
f1 (ppm)

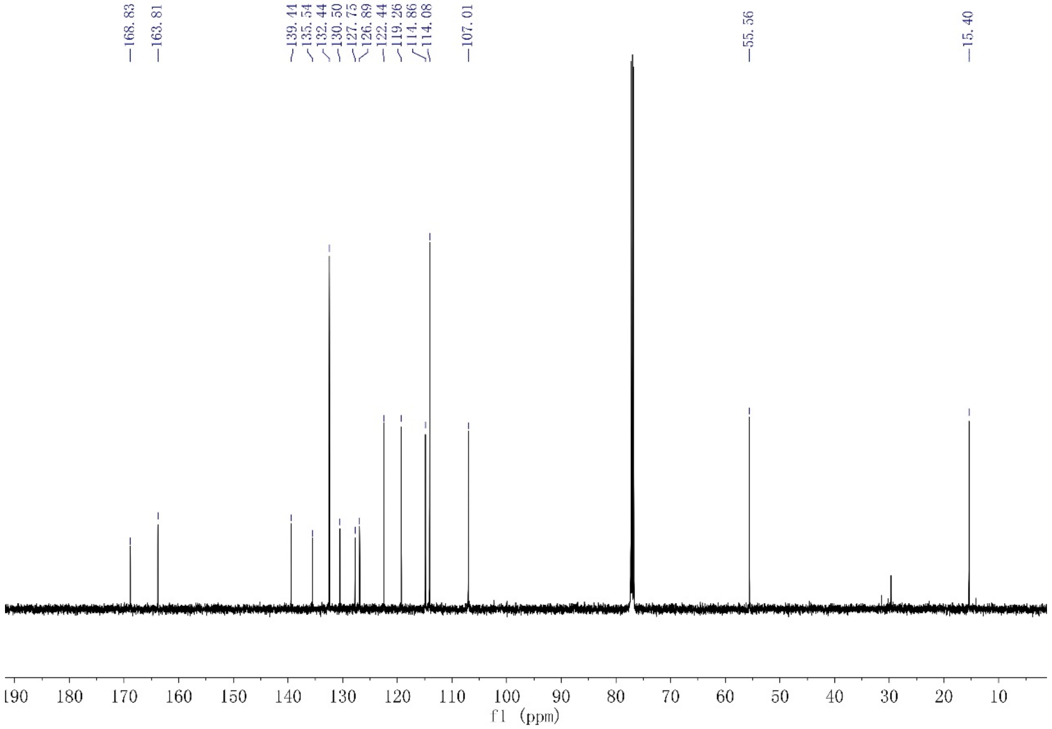
168.83
163.81
139.41
135.54
132.44
130.50
127.75
126.89
122.44
119.26
114.86
114.08
107.01
55.56
15.40
190 180 170 160 150 140 130 120 110 100 90 80 70 60 50 40 30 20 10
f1 (ppm)

2s

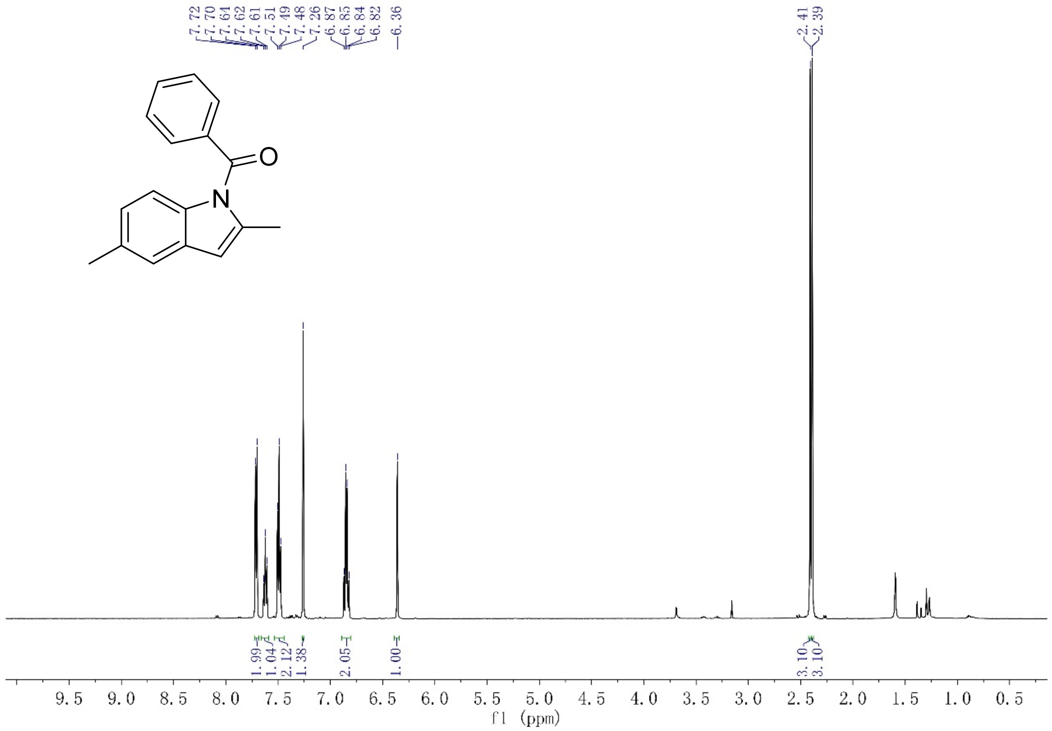
7.72
7.70
7.64
7.62
7.61
7.51
7.49
7.48
7.26
6.87
6.85
6.84
6.82
6.36
2.41
2.39
O
N
1.99
1.04
2.12
1.38
2.05
1.00
3.10
3.10
9.5 9.0 8.5 8.0 7.5 7.0 6.5 6.0 5.5 5.0 4.5 4.0 3.5 3.0 2.5 2.0 1.5 1.0 0.5
f1 (ppm)

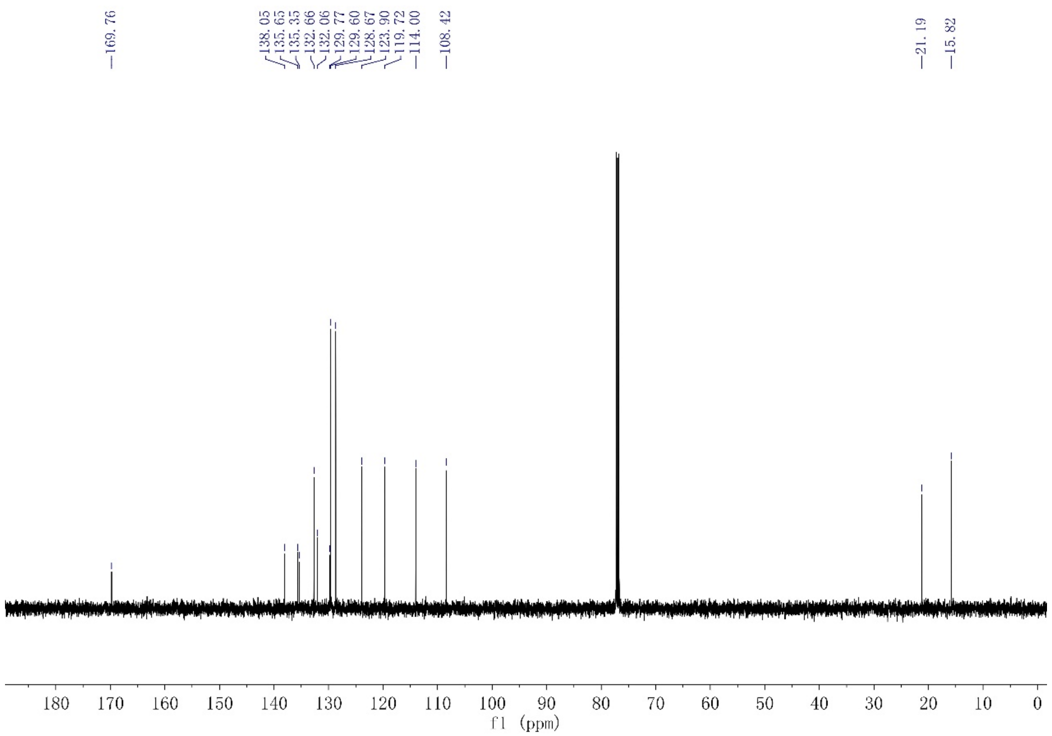
169.76
138.05
135.65
135.35
132.66
132.06
129.77
129.60
128.67
123.90
119.72
114.00
108.42
21.19
15.82
180 170 160 150 140 130 120 110 100 90 80 70 60 50 40 30 20 10 0
f1 (ppm)

2t

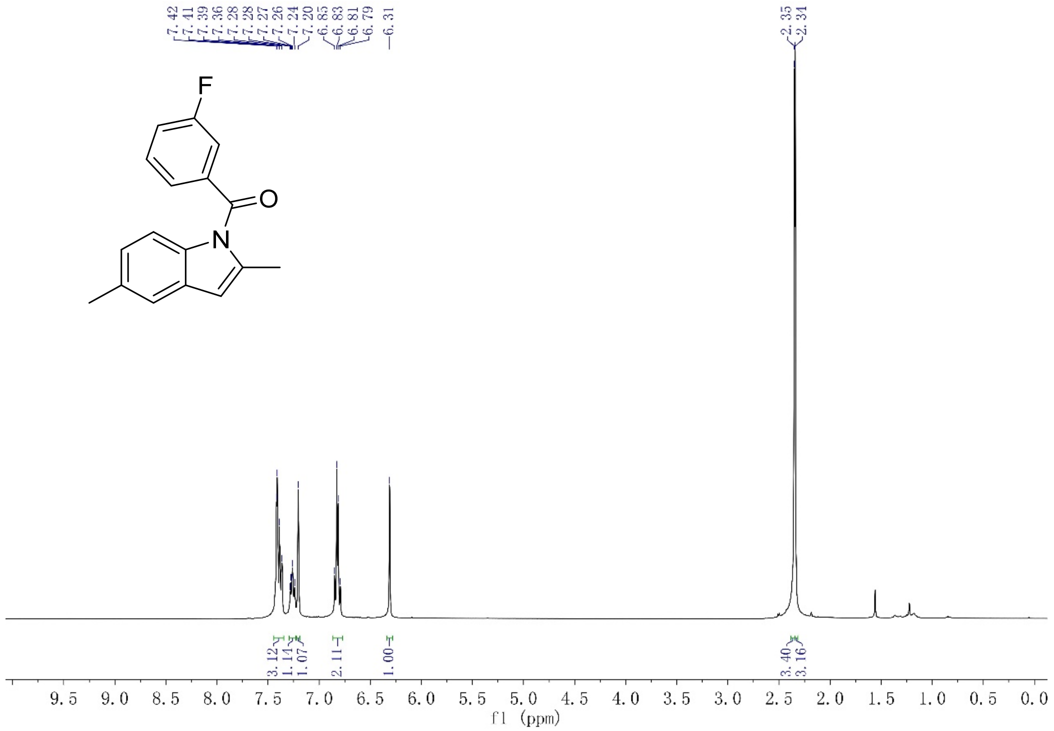
F
O
N
7.42
7.41
7.39
7.36
7.28
7.28
7.27
7.26
7.24
7.20
6.85
6.83
6.81
6.79
6.31
2.35
2.34
3.12
1.14
1.07
2.11
1.00
3.40
3.16
9.5 9.0 8.5 8.0 7.5 7.0 6.5 6.0 5.5 5.0 4.5 4.0 3.5 3.0 2.5 2.0 1.5 1.0 0.5 0.0
f1 (ppm)

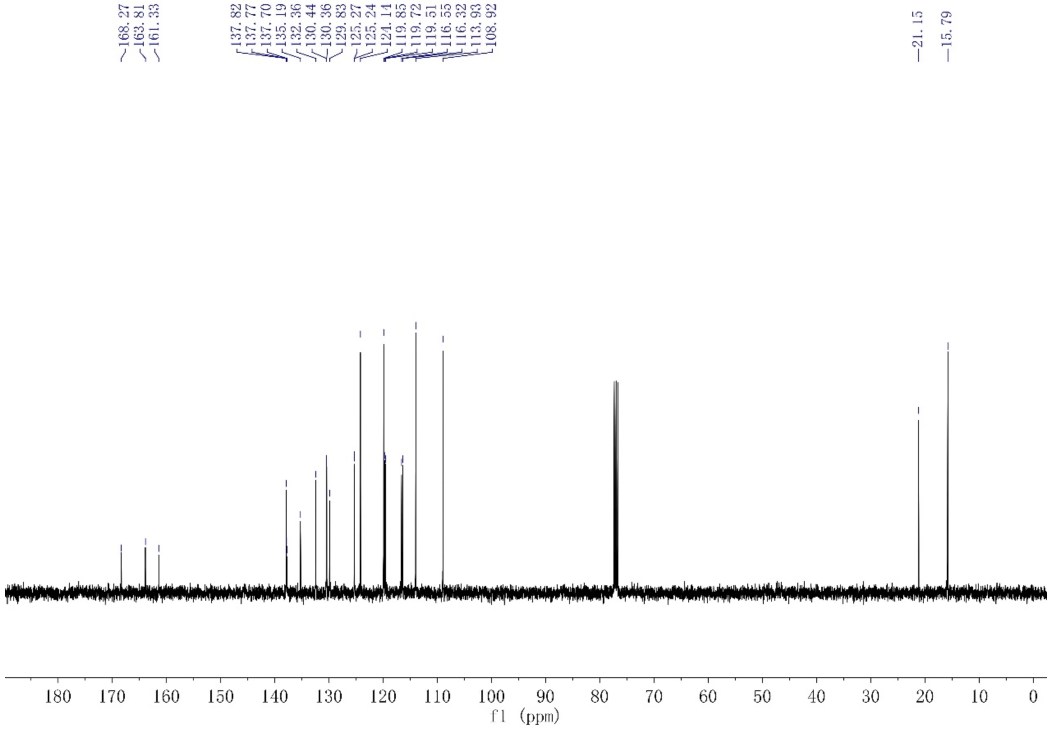
168.27
163.81
161.33
137.82
137.77
137.70
135.19
132.36
130.44
130.36
129.83
125.27
125.24
124.14
119.85
119.72
119.51
116.55
116.32
113.93
108.92
21.15
15.79
180 170 160 150 140 130 120 110 100 90 80 70 60 50 40 30 20 10 0
f1 (ppm)

2v

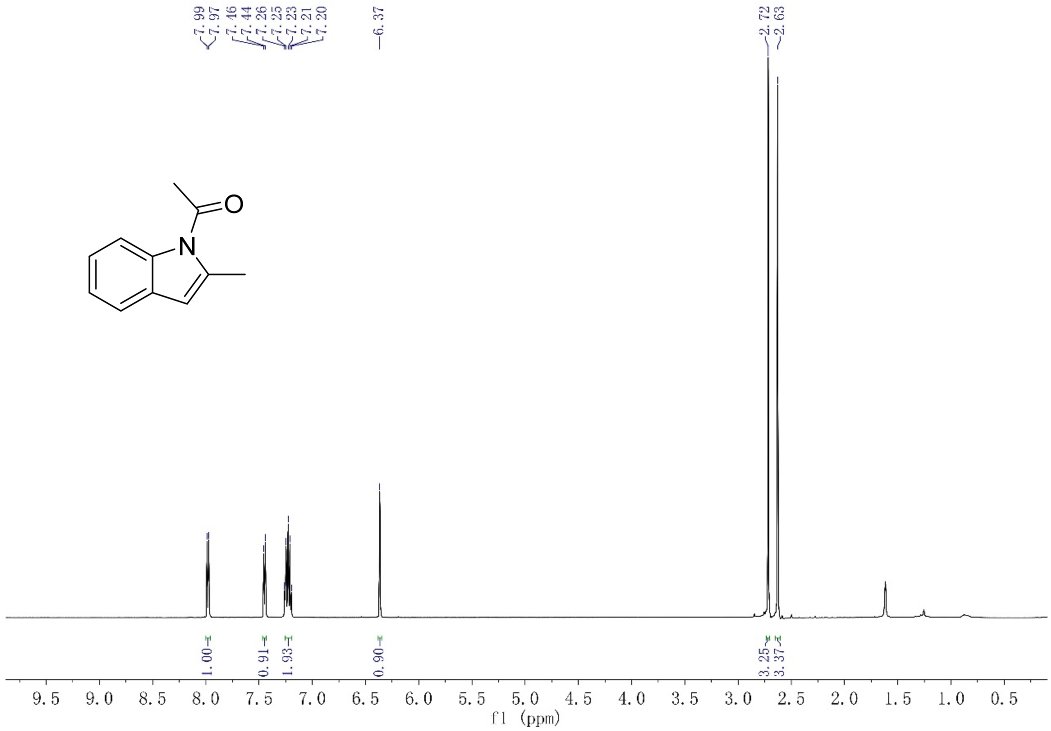
7.99
7.97
7.46
7.44
7.26
7.25
7.23
7.21
7.20
6.37
2.72
2.63
1.00
0.91
1.93
0.90
3.25
3.37
9.5 9.0 8.5 8.0 7.5 7.0 6.5 6.0 5.5 5.0 4.5 4.0 3.5 3.0 2.5 2.0 1.5 1.0 0.5
f1 (ppm)

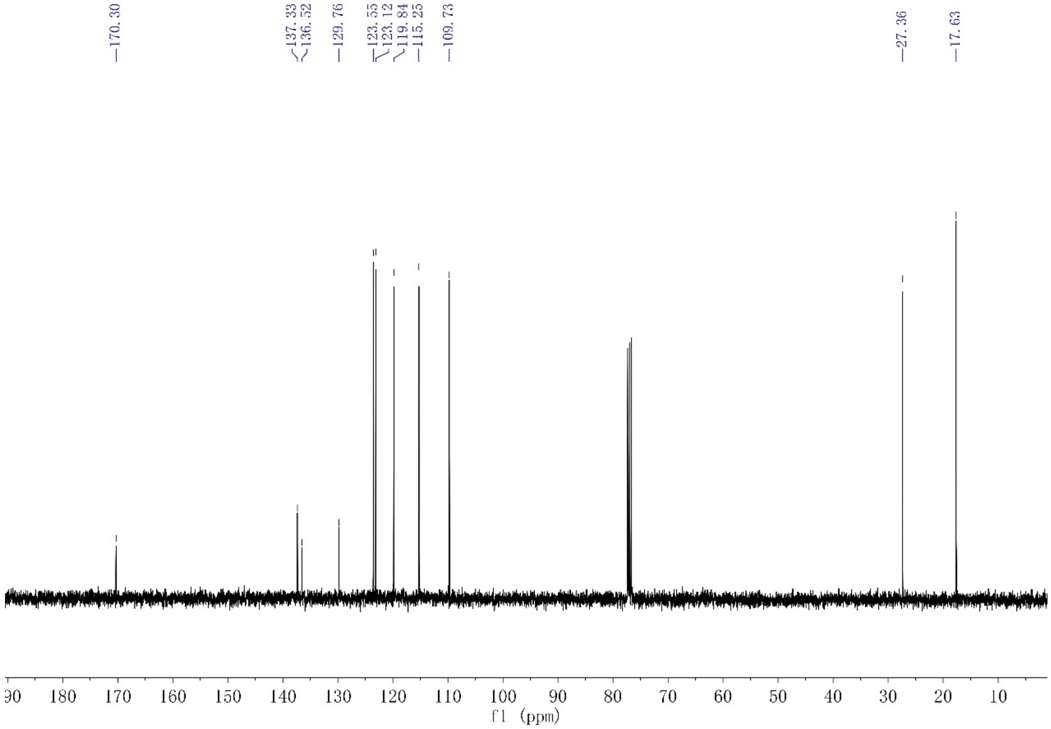
170.30
137.33
136.52
129.76
123.55
123.12
119.84
115.25
109.73
27.36
17.63
90 180 170 160 150 140 130 120 110 100 90 80 70 60 50 40 30 20 10
f1 (ppm)

2w

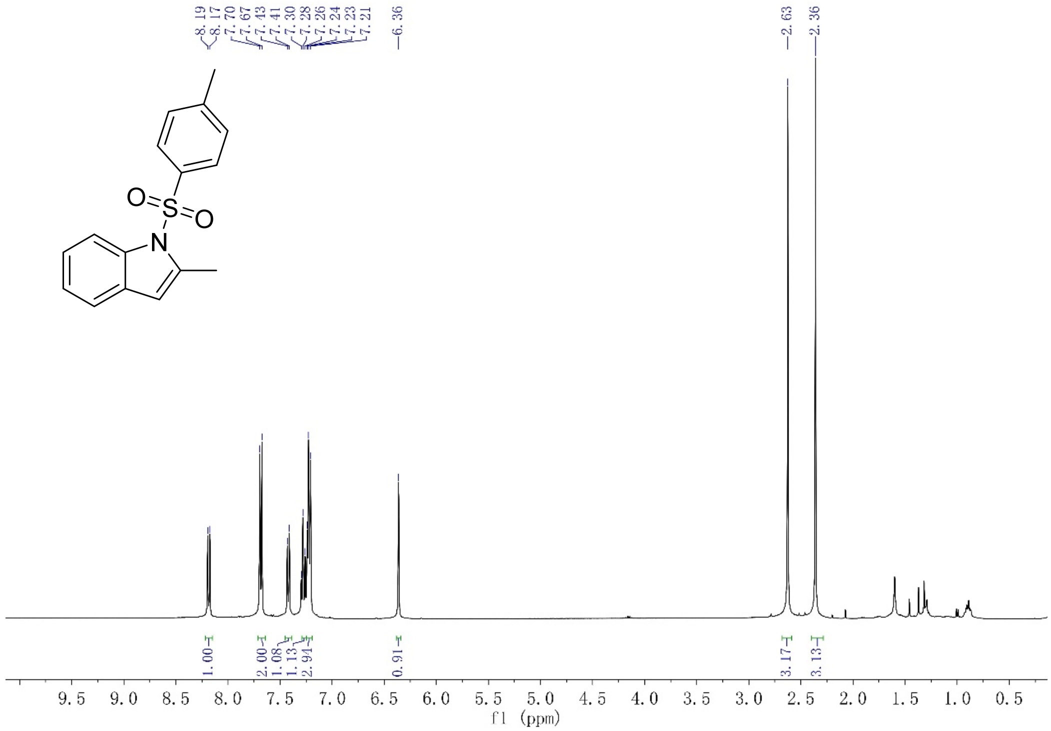
O
S
O
N
8.19
8.17
7.70
7.67
7.43
7.41
7.30
7.28
7.26
7.24
7.23
7.21
6.36
2.63
2.36
1.00
2.00
1.08
1.13
2.91
0.91
3.17
3.13
9.5 9.0 8.5 8.0 7.5 7.0 6.5 6.0 5.5 5.0 4.5 4.0 3.5 3.0 2.5 2.0 1.5 1.0 0.5
f1 (ppm)

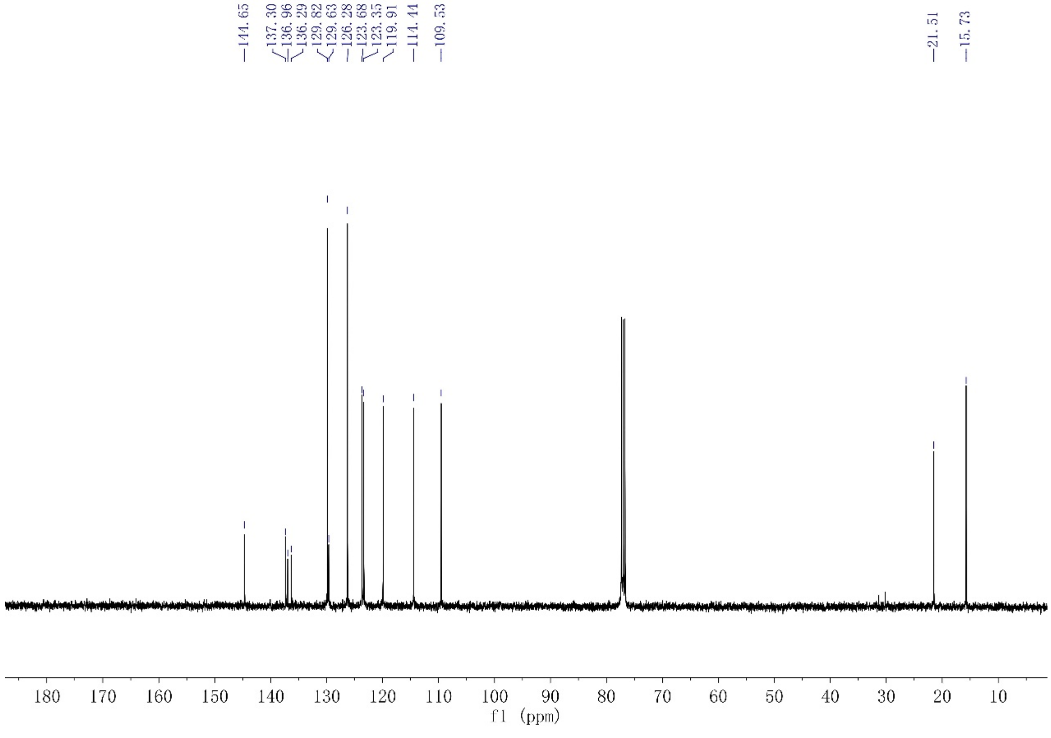
144.65
137.30
136.96
136.29
129.82
129.63
126.28
123.68
123.35
119.91
114.44
109.53
21.51
15.73
180 170 160 150 140 130 120 110 100 90 80 70 60 50 40 30 20 10
f1 (ppm)

5

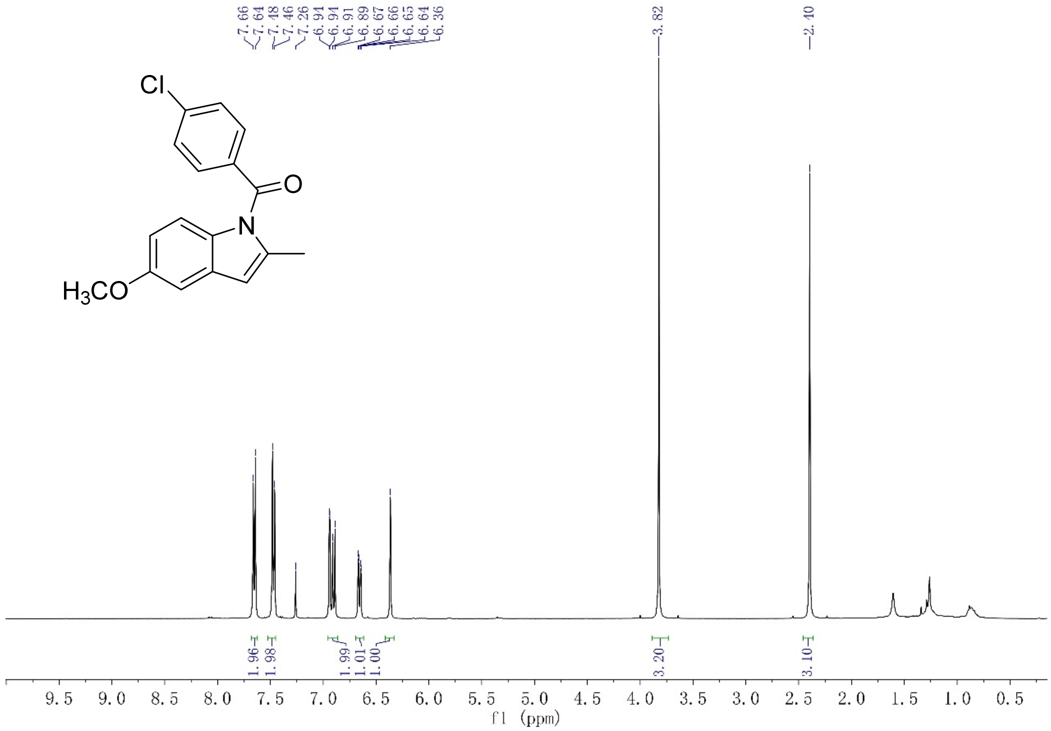
7.66
7.64
7.48
7.46
7.26
6.91
6.91
6.91
6.89
6.67
6.66
6.65
6.64
6.36
3.82
2.40
1.96
1.98
1.99
1.01
1.00
3.20
3.10
f1 (ppm)

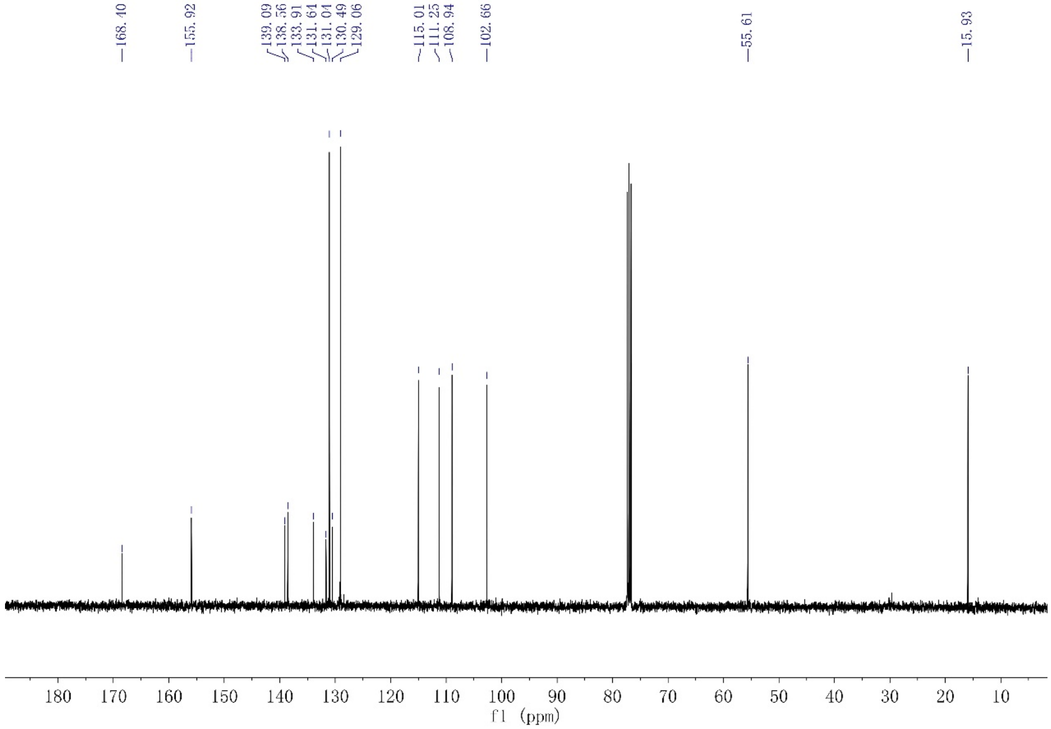
168.40
155.92
139.09
138.56
133.91
131.64
131.04
130.49
129.06
115.01
111.25
108.94
102.66
55.61
15.93
f1 (ppm)
